# Income gaps in self-rated poor health and its association with life expectancy in 245 districts of Korea

**DOI:** 10.4178/epih.e2017011

**Published:** 2017-03-15

**Authors:** Ikhan Kim, Jinwook Bahk, Sung-Cheol Yun, Young-Ho Khang

**Affiliations:** 1 Department of Health Policy and Management, Seoul National University College of Medicine, Seoul, Korea; 2 Institute of Health Policy and Management, Seoul National University Medical Research Center, Seoul, Korea; 3 Department of Public Health, Keimyung University, Daegu, Korea; 4 Department of Clinical Epidemiology and Biostatistics, Asan Medical Center, University of Ulsan College of Medicine, Seoul, Korea

**Keywords:** Geography, Income, Self-rated health, Socioeconomic factors, Korea

## Abstract

**OBJECTIVES:**

To examine the income gaps associated with self-rated poor health at the district level in Korea and to identify the geographical correlations between self-rated poor health, life expectancy, and the associated income gaps.

**METHODS:**

We analyzed data for 1,578,189 participants from the Community Health Survey of Korea collected between 2008 and 2014. The age-standardized prevalence of self-rated poor health and the associated income gaps were calculated. Previously released data on life expectancy and the associated income gaps were also used. We performed correlation and regression analyses for self-rated poor health, life expectancy, and associated income gaps.

**RESULTS:**

Across 245 districts, the median prevalence of self-rated poor health was 15.7% (95% confidence interval [CI], 14.6 to 16.8%), with interquartile range (IQR) of 3.1 percentage points (%p). The median interquintile gaps in the prevalence of self-rated poor health was 11.1%p (95% CI, 8.1 to 14.5%p), with IQR of 3.6%p. Pro-rich inequalities in self-rated health were observed across all 245 districts of Korea. The correlation coefficients for the association between self-rated poor health and the associated income gaps, self-rated poor health and life expectancy, and income gaps associated with self-rated poor health and life expectancy were 0.59, 0.78 and 0.55 respectively.

**CONCLUSIONS:**

Income gaps associated with self-rated poor health were evident across all districts in Korea. The magnitude of income gaps associated with self-rated poor health was larger in the districts with greater prevalence of self-rated poor health. A strong correlation between self-rated poor health and life expectancy was also observed.

## INTRODUCTION

Self-rated health is known to be a predictor of mortality [1]. Korean studies have also shown that it is an important predictor of mortality [2-4]. Self-rated health has been widely used as an indicator of subjective health status [5,6]. In measuring the magnitude of socioeconomic inequalities in health, it is necessary to use subjective health indicators such as self-rated health as well as objective health indicators such as mortality.

Interest in health equality has been growing in Korea over the last 10 years [7]. In the ‘Health Plan 2010’ established in 2005, achieving health equity according to income has been established as a major goal for the first time in Korea [8]. The third National Health Plan 2020 identified the reduction of health inequality according to geographical areas and income levels as the health equity targets [9]. This was continued in the most recent fourth National Health Plan 2020 established in 2015 [10]. Thus, awareness of health inequality between geographical areas and income levels is increasing, as is the demand to resolve the issue. Policies to reduce health inequality are most effective when local government (district authorities) makes efforts to resolve the problem in addition to interventions by the central government. For local government to pursue policies to achieve health equity, it is essential to monitor the socioeconomic differences in health at the district level. Thus far, socioeconomic health inequalities between local districts (si, gun, and gu) in Korea have been investigated by analyzing death certificate and the Korea Community Health Survey (KCHS) data, but the magnitude of health inequalities has not been sufficiently monitored within individual si, gun and gu (e.g., the income differences in health status or health behaviors in a single district).

Meanwhile, a study conducted by the National Health Insurance Service (NHIS) in 2015 showed the presence and magnitude of income gaps in life expectancy at the district level [11]. However, unlike mortality which is an objective health indicator, income gap in self-reported health which is a subjective health indicator has not been reported at the district level. Moreover, the correlation between these two indicators would be of great interest.

This study investigated income differences in self-rated poor health at the district level using the KCHS, and compared these results with previously published results for income gaps in life expectancy.

## MATERIALS AND METHODS

### Data and units of analysis

KCHS was conducted over 2.5 months annually from 2008, with approximately 900 residents aged 19 years and older. Stratified cluster sampling methods across the country have been used to recruit participants, based on resident registration data for each district. Of the 1,578,990 total respondents aged 20 years and older in the KCHS in 2008-2014, 801 respondents (0.05%) with missing responses to self-rated health or household size questions were excluded, and the remaining 1,578,189 respondents were included in the analysis. The 252 si, gun and gu according to the 2015 governmental administrative classification were reclassified to 245 units of analysis, to account for changes in local administrative district between 2008 and 2014. Further details about data and units of analysis were described elsewhere [12].

### Self-rated poor health

The prevalence of self-rated poor health was defined as the proportion of subjects responding “poor” or “very poor” to the question “How do you think your health is usually?” The reason for using this definition is because prior studies showed that the relative risk of death was similar among people who responded “very good,” “good,” or “average,” but increased significantly in people who responded “poor” or “very poor” [2-4].

### Income

We utilized equivalized annual household income in calculating the income quintiles. When calculating equivalized annual household income, self-reported annual or monthly household income and household size were used. Unlike 2008 through 2013, when household income was measured as a continuous variable, monthly household income was recorded as one of 8 categories since 2014 (less than 500,000 Korean won [KRW], 500,000-1,000,000 KRW, 1,000,000-2,000,000 KRW, 2,000,000-3,000,000 KRW, 3,000,000- 4,000,000 KRW, 4,000,000-5,000,000 KRW, 5,000,000-6,000,000 KRW and > 6,000,000 KRW). Therefore, we transformed the categorized monthly household income in 2014 into a continuous variable using the median value of the relevant category, and then converted it to an annual household income. Other details about calculating the income quintiles in each si, gun, and gu have been described elsewhere [12].

### Use of life expectancy data

This study used life expectancies that had been calculated according to income levels for individual si, gun, and gu in a previous study [11]. The NHIS released life expectancies for each district in Korea, using the National Health Information Database Eligibility Database which was constructed from the whole Korean population, including 294,010,120 subjects and 1,459,660 deaths. The NHIS study analyzed 252 si, gun, and gu based on the administrative districts in 2014, and so in the present study, we reclassified some districts and recalculated the life expectancies to ensure a consistent unit of analysis. More details on calculating life expectancies for each district have been described elsewhere [11].

### Statistical analysis

The income quintiles, age-standardized prevalence and its income gap between the highest 20% and the lowest 20% of income levels were calculated in the same manner as described elsewhere for smoking prevalence and its income gap [12].

We conducted correlation analysis of age-standardized prevalence of self-rated poor health with income gaps in prevalence of self-rated poor health and life expectancy as well as correlation analysis between the income gaps in self-rated poor health and the income gaps in life expectancy. We also performed a linear regression analysis. SAS version 9.4 (SAS Institute Inc., Cary, NC, USA) was used for all analyses.

### Ethics statement

The present study protocol was reviewed and approved by the Seoul National University Hospital Institutional Review Board (IRB no. E-1411-001-620).

## RESULTS

As Table 1 shows, the total number of subjects in the KCHS used in this study was 1,578,189. Of these, a total of 717,183 subjects (45.4%) were men and 861,006 (54.6%) were women. Most respondents were from metropolitan areas at 625,991 followed by rural areas at 511,699 and urban areas at 440,499. The mean age of all respondents was 51.3 years, and the standard deviation was 16.7 years. There was a difference in the mean age between the genders, with a mean age of 50.4 years for men and 52.0 years for women. A similar pattern was still present even after applying sample weights. The number of respondents with a self-rated health of “poor” or “very poor” was 339,708 (crude rate, 21.5%), with women showing a higher crude rate than men. After applying the weights, the age-standardized prevalence of self-rated poor health for all participants was 16.3% (95% confidence interval [CI], 16.3 to 16.4%), men was 13.7% (95% CI, 13.6 to 13.8%), and women was 18.2% (95% CI, 18.1 to 18.3%).

Table 2 displays the median and distribution for the age-standardized prevalence of self-rated poor health and its interquintile income gap in the 245 si, gun, and gu. The median age-standardized prevalence of self-rated poor health for all subjects was 15.7% (95% CI, 14.6 to 16.8%), with the minimum value of 8.1% (95% CI, 7.3 to 8.8%) in Bundang-gu, Seongnam-si, Gyeonggi-do, and the maximum value of 21.8% (95% CI, 20.7 to 22.9%) in Gochanggun, Jeollabuk-do. For the interquintile income gap in self-rated poor health, among all subjects, the median value was 11.1%p (95% CI, 8.1 to 14.5%p), the minimum value of 2.8%p (95% CI, -0.1% to 5.5%p) in Suji-gu, Yongin-si, Gyeonggi-do, and the maximum value of 20.1%p (95% CI, 18.0 to 24.0%p) in Goheung-gun, Jeollanam-do. The results for men and women are also presented in Table 2. The interquintile income gaps in self-rated poor health were over 0.0%p in all 245 districts in Korea, for both genders. Even accounting for the 95% CIs, 244 of the 245 districts (99.6%) for men and women combined, 242 districts (98.8%) for men, and 239 districts (97.6%) for women showed a significantly higher prevalence of self-rated poor health for the lowest income quintile compared to the highest income quintile. Appendix 1 shows the age-standardized prevalences of self-rated poor health in the 245 districts as well as the income gaps in self-rated poor health. Overall, the prevalence of self-rated poor health in women was higher than in men. However, the income gap in self-rated poor health was not significantly different between men and women. Appendics 2 and 3 show the 10 districts with the greatest and the least age-standardized prevalence of self-rated poor health and its interquintile income gaps.

**Figure 1.**
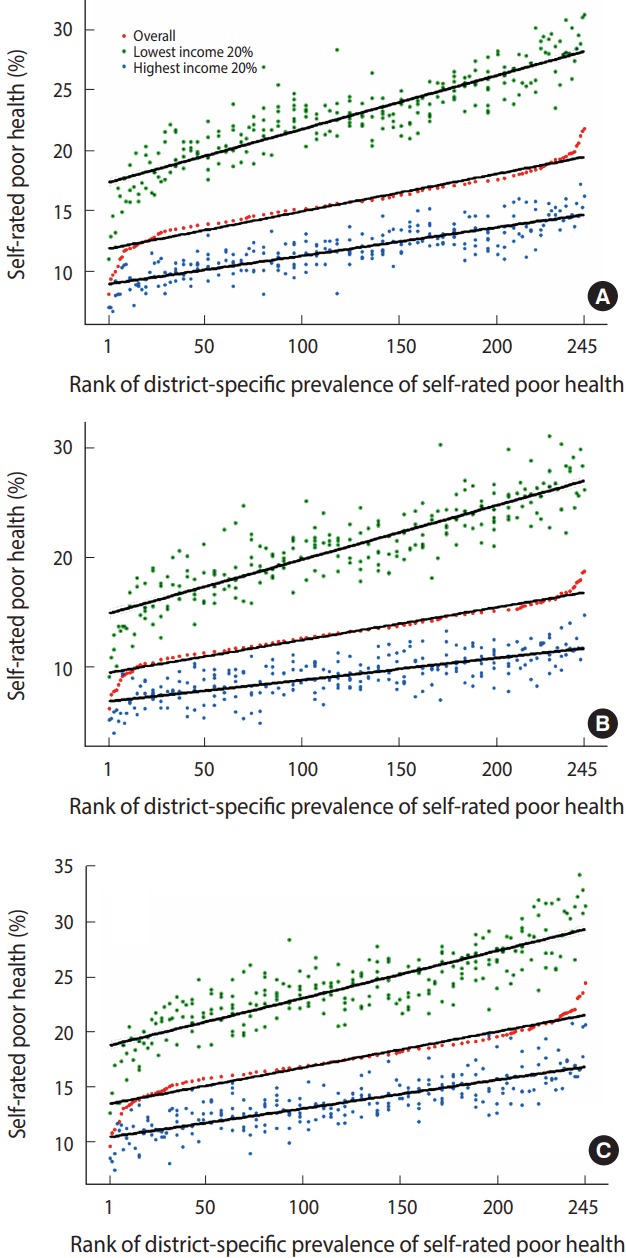
Scatter plots and regression lines of age-standardized prevalence of self-rated poor health in overall, lowest income quintiles (Q5) and highest income quintiles (Q1) on the rank of district-specific prevalence of self-rated poor health in (A) total, (B) men, and (C) women, findings from the Korea Community Health Survey, 2008-2014.

Figure 1 shows regression lines of self-rated health prevalences for the highest 20% and lowest 20% income levels, according to the overall rank of the prevalence of self-rated poor health in 245 si, gun, and gu. As shown in the Figure 1, the age-standardized prevalence of self-rated poor health of the highest and lowest income quintiles tended to increase as overall age-standardized prevalence of self-rated poor health increased. The slope of the regression line was steeper in the lowest income quintile than the highest income quintile. The *p*-value for interaction between the prevalence of self-rated poor health and income level was less than 0.001, with the interquintile income gap in self-rated poor health tending to increase with the increase in prevalence of self-rated poor health. Appendix 4 shows the age-standardized prevalences of self-rated poor health and its income gaps for both genders combined, men and women in all 245 si, gun, and gu.

**Figure 2.**
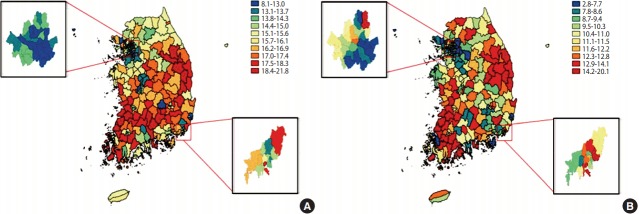
Choropleth maps for (A) the age-standardized prevalences of self-rated poor health and (B) the interquintile income gaps in selfrated poor health in Korea (both genders combined): findings from the Korea Community Health Survey in Korea, 2008-2014.

Figure 2A shows the map of the age-standardized prevalence of self-rated poor health of men and women combined. Age-standardized prevalence of self-rated poor health was high in parts of Gyeongsangnam-do, Gyeongsangbuk-do, and Gangwon-do, as well as areas of Jeollanam-do and Jeollabuk-do. The prevalence of self-rated poor health in Seoul was generally low, but there was a clear difference between the southern Han River (Gangnam) region and northern Han River (Gangbuk) region. In Busan, prevalence of self-rated poor health was high in Dong-gu, Gijang-gun, and Sasang-gu, and low in Geumjeong-gu, Dongnae-gu, and Suyeong-gu, showing a difference between eastern and western regions. Figure 2B presents the interquintile income gap in self-rated poor health of men and women combined. Gangwon-do, Gyeongsangbuk-do, Jeollanam-do, and Jeollabuk-do are shown in red, which indicates a large income gap in self-rated poor health. In Seoul, Nowon-gu, Jung-gu, and Jongno-gu showed large income gaps in self-rated poor health, whereas Songpa-gu, Seocho-gu, and Seongdong-gu showed small gaps. In Busan, Haeundae-gu, Yeongdo-gu, and Seo-gu showed large income gaps in self-rated poor health, while Sasang-gu, Gangseo-gu, and Suyeong-gu showed small gaps.

**Figure 3.**
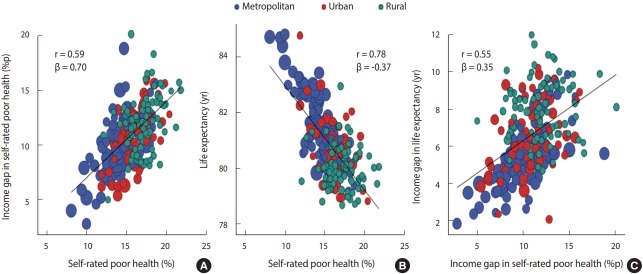
Scatter plots for correlations of age-standardized prevalences of self-rated poor health with (A) interquintile income gaps in self-rated poor health and (B) life expectancy and of (C) interquintile income gaps in self-rated poor health with interquintile income gaps in life expectancy in the 245 local districts of Korea (both genders combined): findings from the Korea Community Health Survey, 2008-2014, and analysis results on life expectancy from the National Health Insurance Service.

Figure 3 shows correlation and regression coefficients for (A) age-standardized prevalence of self-rated poor health with the income gap in self-rated poor health, and for (B) life expectancy with the age-standardized prevalence of self-rated poor health, and for (C) the income gaps in life expectancy with income gaps in self-rated poor health. Life expectancy also showed a clear income gap in all districts (si, gun, and gu) in Korea. The income gap in self-rated poor health showed an increasing pattern with increasing prevalence of self-rated poor health, with a correlation coefficient (r) of 0.59 and a regression coefficient of 0.70. In the relationship between life expectancy and self-rated poor health, life expectancy decreased with increasing prevalence of self-rated poor health. The correlation coefficient was relatively high at 0.78, while the regression coefficient was -0.37. Meanwhile, the income gap in life expectancy tended to be larger in districts with a large income gap in self-rated poor health, with a correlation coefficient of 0.55 and a regression coefficient of 0.35. The p-value was less than 0.001 for all analyses.

## DISCUSSION

This study aimed to examine the income gaps in self-rated poor health of 245 si, gun, and gu in Korea, and to investigate the correlation with the income gap in life expectancy reported in the previous study. Our results showed a clear income gap in self-rated poor health in all 245 si, gun, and gu. There were no districts where the income gap in self-rated poor health was reversed. Even accounting for 95% CIs, when the data for men and women were combined, 244 districts (99.6%) showed a significantly higher prevalence of self-rated poor health in the lowest income quintile compared to the highest income quintile. This pattern demonstrates that there is clear pro-rich inequality in self-rated poor health in Korea. Life expectancy also shows pro-rich inequality in all districts, meaning that there are inequalities in both objective and subjective health indicators (life expectancy and self-rated health) in all 245 si, gun, and gu. Prevalence of self-rated poor health also tended to be higher in districts with a large income gap in self-rated poor health, and this was more so in rural areas than in metropolitan and urban areas. There were strong correlations between self-rated poor health and life expectancy, and the income gaps in self-rated poor health and the income gaps in life expectancy.

According to our results, there were no districts where the age-standardized prevalence of self-rated poor health of women was lower than that of men. This is consistent with previous studies that reported a difference in self-rated health between the genders [13]. Although the gender differences in self-rated poor health might not fully explained, one possible explanation would be differences in the perception of suffering. Women are more sensitive to pain than men, and are known to show less tolerance to suffering [14,15]. For example, women are more vulnerable than men in musculoskeletal disorders and related pain [14,16].

Despite gender differences in reporting self-rated health, this study found pro-rich inequalities in self-rated poor health in all si, gun, and gu for both genders. This is consistent with the results of previous studies conducted at the national level, or at the metropolitan level. Mackenbach et al. [17] investigated seven European countries, and found that prevalence of self-rated poor health was lower in the high-income group than the low-income group. Hildebrand & Van Kerm [18] revealed income gaps in self-rated poor health in both genders in a study of 11 European countries. A prior Korean study employing social statistics surveys from 1989 to 1999 revealed that education-related inequality in self-rated health became more severe in the past 10 years [19]. Kim [20] reported that both men and women in Seoul showed self-rated health inequality related to socioeconomic status, including education, income, and occupation. A study combining data from the Korea National Health and Nutrition Examination Survey (KNHANES) in 1998 and 2001 revealed a close association of self-rated health with economic activities and household income [4]. Another study using data from KNHANES in 2005 showed inequality in self-rated poor health according to social class, education, and income, irrespective of gender [21].

In the present study, age-standardized prevalence of self-rated poor health was higher in rural areas than in urban areas, and the income gap in self-rated poor health was also larger in rural areas. In addition, we observed differences in the prevalence of self-rated poor health and the income gap in self-rated poor health between the capital area and the rest of the country, and between Gangnam and Gangbuk areas in Seoul. As Figure 1 shows, the reason for these differences is that, with increasing prevalence of self-rated poor health, the increase in the prevalence of the highest income quintile was not as large as that of the lowest income quintile. Districts with high prevalence of self-rated poor health (rural areas, Gangbuk in Seoul) had relatively low socioeconomic status, and the absolute income level of the lowest income quintile in these areas was lower than the absolute income level of the lowest income quintile in areas with lower prevalence of self-rated poor health (urban areas, Gangnam in Seoul). Similarly, the absolute income level of the highest income quintile was higher in districts with low prevalence of self-rated poor health, but the relative difference in the absolute income levels between districts among the highest income quintiles was smaller compared to the relative difference in the absolute income levels among the lowest income quintiles. This pattern can be seen in Appendix 5. Considering the close association between income and self-rated health, it seems that a compositional effect was partly responsible for the larger increase of self-rated poor health prevalence in the lowest 20% of incomes compared to the highest 20% with increasing overall self-rated poor health prevalence. A recent prior study showed that the lowest income group among districts with high prevalence of self-rated poor health in this analysis had a higher smoking rate than the lowest income group in districts with low prevalence of self-rated poor health [12]. It was suggested that, besides compositional effects, contextual effects might have also affected smoking rates in these districts, such as the ease of purchasing cigarettes in areas with poor socioeconomic indices [22], or the later implementation of indoor smoking bans [23]. Contextual effects are also expected to contribute to the high prevalence of self-rated poor health in low income groups in socioeconomically disadvantaged districts in terms of access to living environment [24], urban rest facilities [25], and healthcare services [26]. It is possible that compositional and contextual effects might have contributed together to the self-rated health of low-income groups residing in rural areas or Gangbuk area, where the prevalence of self-rated poor health was high.

Our study also showed a correlation between age-standardized prevalences of self-rated poor health and income gaps in self-rated poor health at the level of si, gun, and gu. A study conducted by the NHIS in 2015 reported a similar strong correlation between life expectancy and income gap in life expectancy [11]. This means that, at the level of si, gun, and gu in Korea, districts with less inequality in life expectancy and self-rated poor health also have longer life expectancy and lower prevalence of self-rated poor health. Our study also found strong correlations between age-standardized prevalence of self-rated poor health and life expectancy, and the income gaps in self-rated poor health and income gaps in life expectancy. In other words, among si, gun, and gu in Korea, areas with higher prevalence of self-rated poor health have higher mortality, and areas with greater self-rated poor health inequality have greater mortality inequality. These results indicate the possibility of improving overall objective and subjective health of the district by reducing health inequalities at the district level.

This study has limitations. Household income was measured as a continuous variable in KCHS from 2008 to 2013, but this changed to a categorical variable from 2014. This may limit accurate measurement of household income. Especially because people with a monthly household income of 6,000,000 KRW or over were classified in a single category of “over 6,000,000 KRW”, caution was required when calculating the highest income group. Nevertheless, this study divided the subjects into 14,700 numerator-denominator pairs, by age groups, gender, region, and income quintile; there were 290 pairs, representing 2.0% of the total, in which more than 20% of subjects reported a monthly household income of 6,000,000 KRW or over in the 2014 KCHS, and the number of subjects in these groups was 6,025 which was only 2.6% of the 228,712 respondents in the 2014 KCHS. Moreover, the overall pattern was similar even when the analysis was performed with-out the 2014 KCHS data. Given that the life expectancy data used in this study includes 2014 data, and the use of all available samples at the district level enabled the measurement of prevalence of self-rated poor health by income quintile more stable, we decided to include 2014 KCHS data in the final analysis. In addition, because we focused on the absolute differences in prevalence, we did not provide relative measures such as prevalence ratio. The prevalence ratio can be calculated using Appendix 4.

The present study revealed the magnitude of the income gaps in self-rated poor health for all 245 si, gun, and gu in Korea. We also demonstrated a strong correlation between the income gap in self-rated poor health and income gap in life expectancy in each district. This finding suggests the presence of health inequalities in both subjective and objective health indicators in all districts of Korea. The income gap in self-rated poor health might provide valuable information for monitoring the status of health inequalities in each district in Korea and could be used as evidence to develop local public health policies on equity in health. In the future, further research to explain the cause of variation in self-rated health inequalities by districts and socioeconomic status is needed.

## Figures and Tables

**Table 1.** Characteristics of study subjects in 245 districts of Korea^1^

	Total	Men	Women
No. of study subjects	1,578,189 (100.0)	717,183 (45.4)	861,006 (54.6)
Residential area			
Metropolitan	625,991 (100.0)	284,892 (45.5)	341,099 (54.5)
Urban	440,499 (100.0)	202,102 (45.9)	238,397 (54.1)
Rural	511,699 (100.0)	230,189 (45.0)	281,510 (55.0)
Age	51.3±16.7	50.4±16.1	52.0±17.1
Weighted age	48.2±18.7	46.6±17.8	49.5±19.1
Equivalized household income (10^4^ KRW)	1,808.1±1,910.5	1,892.9±1,975.0	1,737.6±1,852.1
Quintile I (lowest)	657.8±403.1	690.6±407.6	630.5±397.3
Quintile II	1,134.0±524.9	1,203.1±508.1	1,076.8±531.6
Quintile III	1,546.9±641.1	1,626.2±618.7	1,480.3±651.8
Quintile IV	2,093.5±786.6	2,193.7±759.5	2,009.8±799.1
Quintile V (highest)	3,622.2±3,443.0	3,766.8±3,589.3	3,502.3± 3,312.1
No. of subjects with self-rated poor health (crude prevalence of self-rated poor health)	339,708 (21.5)	122,060 (17.0)	217,648 (25.3)
			
Age-standardized prevalence of self-rated poor health^2^ (%, 95% Cl)	16.3 (16.3,16.4)	13.7 (13.6,13.8)	18.2 (18.1,18.3)

Values are presented as number (%) or mean ± standard deviation.

CI, confidence interval; KRW, Korean won.

1 Data from the Korea Community Health Survey, 2008-2014.

2 Age-standardized prevalence of self-rated poor health were estimated with standard population being 2010 mid-year resident population, after considering sample weights of the Korea Community Health Survey.

**Table 2.** Overall age-standardized prevalence of self-rated poor health, self-rated poor health prevalence by income quintiles, and interquintile income gaps in self-rated poor health stratified by gender among 245 local districts of Korea: findings from the Korea Community Health Survey, 2008-2014

Variable	Total					Men					Women				
	Median	SD	Min	Max	IQR	Median	SD	Min	Max	IQR	Median	SD	Min	Max	IQR
Overall	0.157	2.3%	8.1%	21.8%	3.1 %p	13.2%	2.2%	6.2%	18.7%	3.2%p	17.4%	2.4%	9.6%	24.4%	3.0%p
Income															
Q1 (lowest)	0.228	3.6%	11.0%	31.2%	4.6%p	21.1%	4.1%	9.1%	31.0%	5.4%p	23.9%	3.7%	12.6%	34.2%	4.3%p
Q2	0.166	2.6%	8.2%	24.2%	3.1 %p	13.7%	2.8%	5.1%	21.6%	3.5%p	18.5%	2.8%	10.5%	27.7%	3.3%p
Q3	0.145	2.3%	6.7%	22.2%	3.1 %p	11.7%	2.2%	6.4%	19.3%	3.1 %p	16.4%	2.7%	6.9%	27.3%	3.6%p
Q4	12.9%	2.0%	7.1%	20.8%	2.7%p	10.1%	1.9%	5.1%	16.2%	2.5%p	14.9%	2.5%	8.4%	23.8%	3.2%p
Q5 (highest)	11.7%	2.0%	6.7%	17.2%	2.7%p	9.4%	1.9%	4.0%	14.7%	2.6%p	13.4%	2.3%	7.4%	20.7%	2.7%p
Q1-Q5	11.1%p	2.7%p	2.8%p	20.1 %p	3.6%p	11.2%p	3.4%p	2.7%p	23.5%p	4.3%p	10.4%p	3.0%p	1.5%p	19.2%p	4.3%p

SD, standard deviation; Min, minimum; Max, maximum; IQR, interquartile range.

## Appendix 1.

Distribution of (A) age-standardized prevalences of self-rated poor health, and of (B) interquintile income gaps in self-rated poor health in the 245 local districts in South Korea, findings from the Korea Community Health Survey, 2008-2014.

## Appendix 2.

Ten top and bottom districts in terms of age-standardized prevalences of self-rated poor health in Korea: findings from the Korea Community Health Survey, 2008-2014

## Appendix 3.

Ten top and bottom districts in terms of income gaps in self-rated poor health between the lowest and highest income quintiles in South Korea: findings from the Korea Community Health Survey, 2008-2014

## Appendix 4.

Age-standardized prevalences of self-rated poor health, self-rated poor health prevalences according to income quintiles, and income gaps in self-rated poor health (between the highest [Q5] and the lowest [Q1] income quintiles) in 245 local districts of South Korea: findings from the Korea Community Health Survey, 2008-2014

## Appendix 5.

Mean equivalized household income and its relative ratios and absolute differences among metropolitan, urban, and rural areas: findings for total study population, those with the lowest and highest income quintiles using the Community Health Survey in South Korea, 2008-2014
