# Supplementary material for: Income gaps in self-rated poor health and its association with life expectancy in 245 districts of Korea
Source: Epidemiol Health. 2017 Mar 15;39:e2017011. doi: 10.4178/epih.e2017011 (PMC5543297; doi:10.4178/epih.e2017011)
Supplement: Supplementary file 1 [file epih-39-e2017011-supplementary.pdf]

Volume: 39, Article ID: e2017011,

https://doi.org/10.4178/epih.e2017011

우리나라 245 개 시군구 자가평가 불건강 유병률의 소득수준 간 격차 및 기대수명과의 상관성

김익한[1], 박진욱[2,3], 윤성철[4], 강영호[1,2*]

[1]서울대학교 의과대학 의료관리학교실; [2]서울대학교 의학연구원 의료관리학연구소; [3]계명대학교 공중보건학과; [4]울산대학교 의과대학 의학통계학교실

Income gaps in self-rated poor health and its association with life expectancy in 245 districts of Korea

Ikhan Kim[1], Jinwook Bahk[2,3], Sung-Cheol Yun4, Young-Ho Khang[1,2*]

[1]Department of Health Policy and Management, Seoul National University College of Medicine, Seoul, Korea; [2]Institute of Health Policy and Management, Seoul National University Medical Research Center, Seoul, Korea; [3]Department of Public Health, Keimyung University, Daegu, Korea; [4]Department of Clinical Epidemiology and Biostatistics, Asan Medical Center, University of Ulsan College of Medicine, Seoul, Korea

ABSTRACT

OBJECTIVES: To examine the income gaps associated with self-rated poor health at the district level in Korea and to identify the geographical correlations between self-rated poor health, life expectancy, and the associated income gaps.

METHODS: We analyzed data for 1,578,189 participants from the Community Health Survey of Korea collected between 2008 and 2014. The age-standardized prevalence of self-rated poor health and the associated income gaps were calculated. Previously released data on life expectancy and the associated income gaps were also used. We performed correlation and regression analyses for self-rated poor health, life expectancy, and associated income gaps.

RESULTS: Across 245 districts, the median prevalence of self-rated poor health was 15.7% (95% confidence interval [CI], 14.6 to 16.8%), with interquartile range (IQR) of 3.1

percentage points (%p). The median interquintile gaps in the prevalence of self-rated poor health was 11.1%p (95% CI, 8.1 to 14.5%p), with IQR of 3.6%p. Pro-rich inequalities in self-rated health were observed across all 245 districts of Korea. The correlation coefficients for the association between self-rated poor health and the associated income gaps, self-rated poor health and life expectancy, and income gaps associated with self-rated poor health and life expectancy were 0.59, 0.78 and 0.55 respectively.

CONCLUSIONS: Income gaps associated with self-rated poor health were evident across all districts in Korea. The magnitude of income gaps associated with self-rated poor health was larger in the districts with greater prevalence of self-rated poor health. A strong correlation between self-rated poor health and life expectancy was also observed.

KEY WORDS: Geography, Income, Self-rated health, Socioeconomic factors, Korea

서 론

자가평가 건강수준은 사망률의 예측변수로 잘 알려져 있다[1]. 우리나라에서 시행된 연구에서도 자가평가 건강수준은 사망률을 예측하는 중요한 인자로 밝혀졌다[2-4]. 또한 자가평가 건강수준은 주관적 건강상태를 반영하는 중요한 지표로도 활용되어 왔다[5, 6]. 건강 불평등 측정에 있어 사망률과 같은 객관적 건강수준에서의 지표와 함께 자가평가 건강수준과 같은 주관적 건강수준에 있어서의 불평등을 파악하는 것도 중요한 과제라고 할 수 있다.

우리나라에서는 지난 10여 년 간 건강 형평성에 대한 관심이 커져 왔다[7]. 지난 2005년 발간된 '새국민건강증진종합계획' 에서 처음으로 소득수준 간 건강 형평성 확보가 목표로 제시된 바 있고[8], 뒤이어 발표된 제3차 국민건강증진종합계획에서는

지역 간 건강 형평성 목표가 추가되었다 [9]. 최근 수립된 제4차 국민건강증진종합계획에서는 소득 및 지역에 따른 건강 형평성 제고가 목표로 설정된 바 있다 [10]. 이처럼 소득 및 지역간 건강 불평등에 대한 인식이 증가하고, 문제 해결에 대한 요구는 커지고 있다. 그러나 건강 형평성 확보 정책은 중앙정부 차원의 개입 뿐 아니라 지방정부 차원의 문제 해결 노력이 함께 이루어 질 때 가장 효과적일 수 있다. 지방정부별로 건강 불평등 감소 정책을 추진하는 데 있어서 해당 지역의 건강수준의 사회경제적 격차에 대한 정보는 필수적이다. 사망신고자료와 지역사회건강조사 등의 분석을 통해 시군구 간 건강수준에서의 차이에 대한 정보는 제공되어 왔지만, 개별 시군구 내에서 건강 불평등의 현황(예를 들어 한 시군구 내에서의 소득수준에 따른 건강수준이나 건강행태의 차이)에 대한 정보는 충분하게 제공되지 않고 있다.

한편, 2015년 국민건강보험공단에 의해 수행된 연구를 통해 우리나라 시군구 수준에서 기대수명의 소득수준에 따른 불평등의 존재와 그 크기가 밝혀진 바 있다 [11]. 그러나 객관적 건강 상태 지표인 사망지표와 달리 주관적 건강 상태 지표인 자가평가 건강에 있어서는 시군구 수준에서의 소득수준에 따른 격차가 아직 보고되지 못했다. 그리고 두 지표들 간의 상관성 또한 아직까지 규명되지 않았다.

본 연구에서는 지역사회 건강조사 자료를 이용하여 시군구 수준에서 자가평가 불건강 유병률의 소득수준 간 격차를 규명하고, 그 결과를 기존에 보고된 기대수명의 소득수준 간 격차의 결과와 비교해보고자 한다.

Volume: 39, Article ID: e2017011,

https://doi.org/10.4178/epih.e2017011

재료 및 방법

분석 자료 및 단위

지역사회 건강조사는 2008 년부터 매년 각 시군구의 주민등록인구자료를 토대로 층화와 집락을 고려하여 추출된 만 19 세 이상 900 여명의 지역주민들을 대상으로 약 2.5 개월 동안 조사가 이루어진다. 설문조사는 조사원들이 직접 응답자의 가구를 방문하여 노트북에 설치된 전자조사표를 이용하여 이루어진다. 본 연구에서는 2008-2014 년도 지역사회건강조사 자료의 20 세 이상 응답자 총 1,578,990 명 중 자가평가 건강수준 문항이나 가구원 수 문항이 결측인 응답자 801 명(0.05%)을 제외한 1,578,189 명을 분석하였다. 본 연구에서는 우리나라의 2015 년도 기준 252 개 시군구를 2008 년도부터 2014 년도까지의 지방행정구역의 변화를 고려해 245 개 시군구로 다시 분류하였다. 첫째, 2014 년 충청북도 청주시와 청원군이 통합 청주시로 통합되며 상당구, 서원구, 흥덕구, 청원구 등의 행정구로 구성되었는데 본 연구에서는 청주시로 재분류하였다. 둘째, 충청남도 천안시의 경우, 2008 년 6 월 현재의 동남구와 서북구가 설치되었는데 대상자의 지역 정보가 천안시, 동남구 및 서북구 세 가지 범주로 관측됨에 따라 천안시로 통합하였다. 셋째, 전라북도 전주시 또한 실제 행정구역 상으로는 완산구와 덕진구로 이루어져 있지만 위와 같은 이유로 전주시로 통합하여 분석하였다. 넷째, 충청남도 연기군은 2012 년 7 월 세종특별자치시로 편입이 되었다. 따라서 본 연구에서도 세종특별자치시로 포함시켜 분석하였다. 다섯째, 경상남도 통합창원시는 2010 년 창원시, 마산시, 진해시가 통합되어 만들어졌다. 통합창원시는 현재 의창구,

성산구, 마산회원구, 마산합포구, 진해구로 나뉘었는데, 본 연구에서는 기존 행정구역을 감안하여 의창구와 성산구, 마산회원구와 마산합포구 그리고 진해구 세 지역으로 나누어 분석하였다. 도시화 정도는 대도시(Metropolitan area), 도시(Urban area), 농촌(Rural area)을 행정구역에 따라 각각 "구", "시", "군" 지역으로 설정하였다.

자가평가 불건강

자가평가 불건강은 "평소에 본인의 건강은 어떻다고 생각하십니까?"라는 질문에 "나쁨" 혹은 "매우 나쁨"이라고 대답한 사람들의 분율로 정의하였다. 이와 같이 정의한 이유는 선행 연구들에 의해서 자가평가 건강수준을 "매우 좋음", "좋음" 혹은 "보통"이라고 응답한 사람들의 사망에 대한 상대위험도가 비슷하였고, "나쁨" 혹은 "매우 나쁨" 이라고 응답한 사람들에서 유의하게 증가하였기 때문이다 [2-4].

소득

2008 년도부터 2013 년도까지의 지역사회 건강조사는 연간 혹은 월간 가구소득을 연속형 변수로 파악하였다. 그러나 2014 년도부터는 월간 가구소득을 여덟 개의 범주 (50 만원 미만, 50-100 만원 미만, 100 만원-200 만원 미만, 200-300 만원 미만, 300-400 만원 미만, 400-500 만원 미만, 500-600 만원 미만, 600 만원 이상)로 파악하는 것으로

변경되었다. 이에 따라 본 연구에서는 각 범주의 중간값을 응답자의 월 가구소득으로 취해 연속형 변수로 변환한 뒤, 연간 가구소득을 산출하였다. 예를 들면, "100 만원-200 만원 미만"이라고 응답한 경우에는 월 가구소득을 150 만원으로 변환한 뒤, 연간 가구소득을 계산하였다. "600 만원 이상"으로 응답한 경우에는 650 만원으로 변환하였다. 2008 년도부터 2014 년도까지의 자료를 합산했으므로 각 연도에 따른 물가의 변동을 고려할 필요가 있었다. 본 연구에서는 2010 년도 기준 소비자물가지수를 이용하여 가구소득을 보정하였다. 예를 들면, 2008 년도의 가구소득은 2008 년도와 2010 년도 소비자물가지수의 비 100/94.52 를 곱했다. 따라서 모든 가구소득은 2010 년도의 물가를 기준으로 환산되었다. 2008 년도부터 2014 년도까지의 응답자 중 가구소득이 결측인 응답자는 110,579 명(7.0%)이었다. 시군구 수준에서 지역 대표성을 지닌 소득수준 간 건강불평등 지표를 산출하기 위해, 결측인 소득정보는 대치(imputation)하였다. 대치는 소비자물가지수가 반영된 가구소득 정보를 가지고 있는 응답자들의 성별, 5 세별 연령군, 가족 구성원 수, 직업, 교육수준을 활용한 단순 대치(single imputation) 방식으로 이루어졌다. 본 연구에서는 가구원 수의 효과를 보정하기 위하여 '가구원수 보정 연간 가구소득'을 산출하였다. 이 때, 균등화지수는 OECD 연구에서 이용하는 방식을 따라 0.5 을 이용하였다 [12]. 계산식은 아래와 같다.

$$\textit{가구원수 보정 연간 가구소득} = \frac{\textit{연간 가구소득}}{\textit{가구원 수}^{0.5}}$$

245 개의 개별 시군구 내에서 자가평가 불건강 유병률의 소득 5 분위 간 격차 산출을 위해 각 연도별, 시군구별, 성별, 10 세 단위 연령구간별 소득 5 분위를 산출하였다. 소득 5 분위는 특정 연령대의 집단을 소득수준에 따라 서열화 한 뒤, 5 분위 분율(20%, 40%, 60%, 80%)에서 가장 가까운 지점(the nearest quintile points)에서 구분하여 개별 시군구 내에서의 상대적인 소득 순위를 가진 5 분위 집단을 산출하였다.

기대수명 분석 결과의 활용

이 연구에서는 기존에 이루어진 시군구별 소득수준에 따른 기대수명 산출결과를 이용하였다 [11]. 국민건강보험공단(2015)에 의해 수행된 기대수명의 산출은 국민건강보험 자격DB를 활용하여 이루어졌다. 국민건강보험 자격DB는 우리나라 건강보험 가입자 전체를 대상으로 구축되었다. 이 연구에서는 2009년도부터 2014년도까지 6년간의 자료를 통합하여 294,010,120명의 대상자 정보와 1,459,660명의 사망자 정보를 이용하여 분석이 이루어졌다. 이 연구에서는 2014년도 행정구역을 기준으로 252개 시군구별 분석이 이루어졌는데, 본 연구의 시군구 분석단위와 일치시키기 위해 일부 행정구역을 재분류해 기대수명의 재산출이 이루어졌다. 기대수명 산출은 연령별 사망률(${}_nm_x$)을 통해 산출한 연령별 사망확률(${}_nq_x$)을 토대로 연령별 사망자 수와 생존자 수(l_x)를 구한 뒤, 연령별 정지인구(x 연령에서 x+n 연령까지의 기간 동안 생존이 기대되는 생존 연 수의 합계, ${}_nL_x$)를 계산하였다. 계산식은 아래와 같다.

$${}_nq_x = \frac{{}_nm_x}{1+\frac{1}{2}\,{}_nm_x},$$

$$ {}_nL_x = n\frac{(l_x + l_{n+x})}{2}, $$

그 후, 특정 연령 이후의 정지인구를 모두 합해 총 정지인구(T_x)를 계산한 뒤, 이를 생존자 수로 나누어 기대수명(e_x)을 산출하였다. 계산식은 아래와 같다.

$$ T_x = \Sigma_x^{\infty}\, {}_nL_x, $$

$$ e_x = \frac{T_x}{l_x} $$

소득 자료는 국민건강보험 보험료를 이용하였다. 앞선 연구들에 의해 국민건강보험 보험료가 기대수명의 소득에 따른 차이 산출에 유용함이 밝혀진 바 있다 [13, 14]. 6 년간의 국민건강보험 자격 DB 를 통합하여 산출된 대상자들을 연도별, 성별, 시군구별, 5 세 단위 연령별로 소득 5 분위로 나누어, 생성된 각 그룹의 연도별 연앙 인구수와 사망자수를 토대로 성별, 시군구별로 소득수준과 5 세 단위 연령군에 따른 사망확률을 산출한 후 생명표를 작성하여 시군구별 소득수준에 따른 기대수명을 산출하였다. 기대수명의 소득수준 간 격차는 소득수준 상위 20%와 하위 20%의 차로 정의하였다.

분석 방법

지역사회 건강조사 자료의 가구원수 보정 연간 가구소득을 성별과 10 세별 연령군을 고려하여 지역별로 5 분위를 산출해 총 14,700 개의 세부 그룹으로 나눴다. 이 때, 자가평가건강을 "나쁨" 혹은 "매우 나쁨"이라고 보고한 응답자가 한 명도 없는 그룹이 남자에서 273 개(전체 남성에서 3.7%), 여자에서는 186 개 그룹(전체 여성에서 2.5%)으로 나타났다. 이 때, 연령표준화 자가평가 불건강 유병률이 과소추정 될 수 있기 때문에 각 그룹의 분자 수가 0 인 경우 0.01 로 치환했다. 가중치는 지역사회 건강조사 자료에서 제공하는 원 가중치를 이용하여 성별과 연도별로 각 시군구의 응답자 수와 가중치의 총합이 일치하도록 새로운 가중치를 산출하였다. 연령표준화 자가평가 불건강 유병률은 2010 년도 주민등록연앙인구를 표준 인구로 사용하여 산출하였다. 소득 5 분위 간 차이는 소득수준 상위 20%와 하위 20%의 절대적 차이를 이용해서 산출하였다. 그 이유는 유병률의 절대적 차이가 상대비에 비해서 일반인들이 직관적으로 이해하기 쉽다는 장점 때문이었다. 다음 식을 이용해 소득 5 분위 간 격차의 95% 신뢰구간을 산출하였다.

$$\text{Lower CI} = \text{Gap} - (1.96 \text{ x } \sqrt{\frac{P1(1-P1)}{N1} + \frac{P2(1-P2)}{N2}})$$

$$\text{Upper CI} = \text{Gap} + (1.96 \text{ x } \sqrt{\frac{P1(1-P1)}{N1} + \frac{P2(1-P2)}{N2}})$$

Lower CI= 95% 신뢰구간의 하한값, Upper CI= 95% 신뢰구간의 상한값

Gap=소득수준 상, 하위 20% 간 연령표준화 자가평가 불건강 유병률 격차

P1=소득수준 상위 20% 연령표준화 자가평가 불건강 유병률,

Volume: 39, Article ID: e2017011,

https://doi.org/10.4178/epih.e2017011

P2=소득수준 하위 20% 연령표준화 자가평가 불건강 유병률

N1=소득수준 상위 20% 인구수, N2=소득수준 하위 20% 인구수

연령표준화 자가평가 불건강 유병률과 소득수준 간 격차 및 기대수명 그리고 연령표준화 자가평가 불건강 유병률 소득수준 간 격차와 기대수명의 소득수준 간 격차와의 상관분석 및 선형 회귀분석을 수행하였다. 모든 분석은 SAS 9.4 를 이용하여 이루어졌다.

결 과

<Table 1>에서 볼 수 있듯이 본 연구에 사용된 지역사회 건강조사의 대상자는 총 1,578,189명이었다. 그 중 남자는 717,183명(45.4%), 여자는 861,006명(54.6%)이었다. 대도시 지역에 사는 응답자가 625,991명으로 가장 많았고, 농촌 지역이 511,699명, 도시 지역이 440,499명이었다. 모든 응답자의 평균 나이는 51.3세, 표준편차는 16.7세였다. 평균 나이는 성별에 따라 차이가 있었는데 남성이 50.4세, 여성이 52.0세로 나타났다. 표본가중치를 적용한 후에도 유사한 양상이 나타났다. 자신의 건강수준을 '나쁨' 혹은 '매우 나쁨'이라고 평가한 사람은 339,708명(조율 = 21.5%)이었는데 남자보다는 여자에서 조율값이 더 높았다. 가중치를 적용한 연령표준화 자가평가 불건강 유병률은 남녀 전체에서는 16.3% (95% CI: 16.3%-16.4%), 남성은 13.7% (95% CI: 13.6%-13.8%), 여성은

18.2% (95% CI: 18.1%-18.3%)로 나타났다.

<Table 2>에는 245개 시군구별 연령표준화 자가평가 불건강 유병률과 소득수준별 격차의 대표값(중앙값)과 분포 양상이 제시되어 있다. 남녀 전체의 연령표준화 자가평가 불건강 유병률의 중앙값은 15.7% (95% CI: 14.6%-16.8%)이었고, 최소값은 경기도 성남시 분당구의 8.1% (95% CI: 7.3%-8.8%), 최대값은 전라북도 고창군의 21.8% (95% CI: 20.7%-22.9%)이었다. 남성에서 중앙값은 13.2% (95% CI: 11.8%-14.5%), 최소값은 경기도 성남시 분당구의 6.2% (95% CI: 5.2%-7.2%), 최대값은 강원도 태백시의 18.7% (95% CI: 17.2%-20.2%)이었다. 여성에서는 중앙값이 17.4% (95% CI: 15.8%-18.9%), 최소값은 경기도 성남시 분당구의 9.6% (95% CI: 8.5%-10.6%), 최대값은 전라북도 고창군의 24.4% (95% CI: 22.9%-26.0%)이었다. 남녀 전체 소득수준 상위 20%와 하위 20% 간 연령표준화 자가평가 불건강 유병률의 격차의 중앙값은 11.1%p (95% CI: 8.1%p-14.5%p)이었고 최소값은 경기도 용인시 수지구의 2.8%p (95% CI: -0.1%p-5.5%p), 최대값은 전라남도 고흥군의 20.1%p (95% CI: 18.0%p-24.0%p)로 나타났다. 남성 연령표준화 자가평가 불건강 유병률 격차의 중앙값은 11.2%p (95% CI: 7.0%p-15.4%p)이었고 최소값은 경기도 수원시 영통구와 경기도 성남시 분당구의 2.7%p (95 CI: -1.1%p-6.5%p), 최대값은 전라남도 고흥군의 23.5%p (18.9%p-28.1%p)이었다. 여성의 경우, 연령표준화 자가평가 불건강 유병률 격차의 중앙값은 10.4%p (95% CI: 5.9%p-14.5%p)로 나타났고, 최소값은 경기도 용인시 수지구의 1.5%p (95% CI: -2.2%p-5.2%p), 최대값은 부산광역시 해운대구의 19.2%p (95% CI: 14.8%p-23.6%p)가 기록했다. 소득수준 하위 20%와 상위 20% 간 연령표준화 자가평가 불건강 유병률의 격차는 성별에 상관없이 245개 전체 시군구

지역에서 0%p보다 컸다. 95% 신뢰구간을 고려하더라도 245개 시군구 중 남녀 전체에서는 244개 지역(99.6%), 남성에서는 242개 지역(98.8%), 여성은 239개 지역(97.6%)에서 소득수준 하위 20%의 자가평가 불건강 유병률이 상위 20%보다 통계적으로 유의하게 높은 것으로 나타났다.

<Appendix 1>은 우리나라 245개 시군구에서 자가평가 불건강 유병률과 이의 소득계층간 불평등 크기를 남녀별로 제시한 그림이다. 전반적으로 남성보다 여성에서의 자가평가 불건강 유병률이 높은 양상을 나타냈다. 하지만 자가평가 불건강 유병률의 소득5분위간 격차의 크기는 남녀별로 큰 차이를 보이지는 않았다.

<Figure 1>은 245개 시군구의 연령표준화 자가평가 불건강 유병률의 순위에 따라 최하위 소득군과 최상위 소득군의 연령표준화 자가평가 불건강 유병률과 회귀직선을 보여주고 있다. 그림에서 볼 수 있듯이 연령표준화 자가평가 불건강 유병률이 높아질수록 최하위 및 최상위 소득군의 연령표준화 자가평가 불건강 유병률 또한 높아지는 양상을 관찰할 수 있었다. 최하위 소득군의 회귀선 기울기는 최상위 소득군에 비해서 컸다. 지역의 자가평가 불평등 유병률과 소득수준의 교호작용은 통계적으로 유의하여 (P <0.001), 자가평가 불평등 유병률이 커질수록 최상위 및 최하위 소득군 간 유병률 격차의 크기는 커지는 양상이 나타났다.

<Appendix 2>과 <Appendix 3>에는 연령표준화 자가평가 불건강 유병률과 그것의 소득수준 간 격차가 가장 높거나 낮은 10개 지역들이 제시되어 있다. 남녀 전체 연령표준화 자가평가 불건강 유병률은 전라북도 고창군, 경상남도 남해군 등의 지역에서 높았고 경기도 성남시 분당구, 서울특별시 서초구 등의 지역에서 낮았다. 남자에서는

강원도 태백시, 경상남도 남해군 등에서 자가평가 불건강 유병률이 높았고 경기도 성남시 분당구, 서울특별시 서초구에서 낮았다. 여자 연령표준화 자가평가 불건강 유병률은 전라북도 고창군, 경상남도 남해군 등의 지역들에서 높은 것으로 나타났고, 경기도 성남시 분당구, 서울특별시 서초구 등에서 낮았다. 남녀 전체 연령표준화 자가평가 불건강 유병률의 소득수준 간 격차가 큰 지역들은 전라남도 고흥군, 부산광역시 해운대구 등으로 나타났고, 작은 지역들은 경기도 용인시 수지구, 경기도 성남시 분당구 등이었다. 남자에서는 전라남도 고흥군, 전라남도 완도군 등에서 컸고, 경기도 수원시 영통구, 경기도 성남시 분당구 등의 지역들에서 작은 것으로 나타났다. 여자의 경우, 부산광역시 해운대구, 전라남도 고흥군에서 연령표준화 자가평가 불건강 유병률 격차가 컸고 경기도 용인시 수지구, 경상북도 청도군 등의 지역들에서 그 격차가 작았다. <Appendix 4>에는 245개 전체 시군구 지역의 연령표준화 자가평가 불건강 유병률과 그 격차가 제시되어 있다.

우리나라 시군구별 남녀 전체 연령표준화 자가평가 불건강 유병률과 그것의 소득수준 간 격차를 지도로 표현한 것이 <Figure 2-A, B>에 나타나 있다. 연령표준화 자가평가 불건강 유병률은 경상남도와 경상북도 및 강원도의 일부 지역들과 전라남도와 전라북도 일부 지역 등에서 높은 양상을 보였다. 서울특별시의 경우, 전국의 자가평가 불건강 유병률 수준과 비교했을 때 모든 시군구에서 낮은 경향을 보였지만 강남과 강북 지역 간 뚜렷한 격차를 보였다. 부산광역시에서는 동구, 기장군, 사상구 등의 지역들에서 유병률이 높았고 금정구, 동래구, 수영구 등의 지역들에서는 낮은 것으로 나타나 동부와 서부 지역 간 유병률 격차가 나타났다. 연령표준화 자가평가 불건강 유병률 격차에서는

Volume: 39, Article ID: e2017011,

https://doi.org/10.4178/epih.e2017011

강원도, 경상북도 및 전라남도와 전라북도 지역이 큰 격차를 의미하는 붉은색으로 나타났다. 서울특별시의 경우, 노원구, 중구 및 종로구에서 연령표준화 자가평가 불건강 유병률 격차가 컸고 송파구, 서초구, 성동구에서는 그 격차가 작았다. 부산광역시에서는 해운대구, 영도구, 서구 등에서 큰 격차가 관찰되었고 사상구, 강서구, 수영구 등에서는 격차가 작았다.

<Figure 3-A, B, C>에는 245개 시군구별 남녀 전체 연령표준화 자가평가 불건강 유병률과 소득수준 간 격차(A), 기대수명과 연령표준화 자가평가 불건강 유병률 소득수준 간 격차(B), 그리고 기대수명의 소득수준 간 격차와 연령표준화 자가평가 불건강 유병률의 소득수준 간 격차(C)의 상관관계와 회귀계수가 나타나 있다. 기대수명에서도 우리나라 모든 시군구 지역에서 소득수준간 격차가 존재하는 것으로 나타났다(Figure 3-C 참조). 연령표준화 자가평가 불건강 유병률 및 소득수준 간 격차에서는 연령표준화 자가평가 불건강 유병률이 높을수록 소득수준 간 격차 또한 큰 양상을 보였고, 상관계수(r 값)는 0.59이었다. 회귀계수는 0.70이었다. 연령표준화 자가평가 불건강 유병률과 기대수명의 관계에서는 유병률이 높은 지역일수록 기대수명은 낮아지는 양상이 나타났다. 상관계수의 크기는 0.78로 상대적으로 컸고 회귀계수는 -0.37으로 나타났다. 또한 연령표준화 자가평가 불건강 유병률 소득수준 간 격차가 큰 지역일수록 기대수명의 소득수준 간 격차 또한 큰 양상을 보였는데 상관계수는 0.55, 회귀계수는 0.35였다. 모든 분석에서 P값은 0.001 미만이었다.

고 찰

본 연구는 우리나라의 245개 시군구별 자가평가 불건강 유병률의 소득수준 간 격차를 산출하고, 이를 기존에 보고된 기대수명의 소득수준 간 격차와의 상관성을 밝히는 것을 목적으로 하였다. 연구 결과, 우리나라 모든 시군구에서 연령표준화 자가평가 불건강 유병률의 소득수준 간 격차가 뚜렷하게 나타났다. 연령표준화 자가평가 불건강 유병률의 소득수준 간 격차가 역전되는 지역은 존재하지 않았다. 95% 신뢰구간을 고려할 경우에도 남녀 전체 244개 지역(99.6%)에서 소득 하위 20% 집단이 상위 20% 집단보다 유의하게 높은 자가평가 불건강 유병률을 보였다. 이와 같은 양상은 우리나라에서 자가평가 불건강 유병률에서의 불평등이 매우 명료하다는 점을 의미한다. 기대수명에서의 소득수준간 격차의 경우에도 모든 시군구에서 높은 소득군에 유리한 불평등이 존재하였으므로, 우리나라 245개 전체 시군구 내에서 객관적, 주관적 건강지표(기대수명과 주관적 건강수준) 모두 불평등이 존재한다고 볼 수 있다. 자가평가 불건강 유병률의 소득수준 간 격차가 큰 지역들은 자가평가 불건강 유병률 또한 높은 양상을 보였는데, 이는 도시보다는 농촌이 그러하였다. 자가평가 불건강 유병률과 기대수명, 자가평가 불건강 유병률 소득수준 간 격차와 기대수명의 소득수준 간 격차는 높은 상관성을 나타냈다.

연구 결과에 따르면, 여성의 연령표준화 자가평가 불건강 유병률이 남성의 유병률보다 낮은 지역은 한 군데도 없었다. 이는 자가평가 건강수준의 측정에서 성별에 따른 차이가 존재함을 보고한 선행 연구의 결과와 같다 [15]. 성별에 따른 자가평가 불건강 유

병률의 차이는 완전히 해명되지는 않았지만, 고통의 경험에 대한 차이가 하나의 설명 방법이 될 수 있다. 여성들은 고통에 대한 감수성이 남성들보다 더 크고, 고통에 대한 내성(tolerance)은 더 작은 것으로 알려져 있다 [16, 17]. 예를 들어, 여성은 남성보다 근골격계 질환에 따른 고통의 경험에 더 취약하다 [16, 18].

성별 자가평가 건강수준 보고의 차이에도 불구하고 본 연구에서는 연령표준화 자가평가 불건강 유병률의 소득수준 간 격차가 소득수준에 따른 역전 현상 없이 남녀 모두 저소득층에 불리한 불평등이 모든 시군구에서 나타났다. 이는 국가 혹은 광역시도 수준에서 시행한 선행 연구들의 결과와도 부합한다. Mackenbach 등(2004)에 의해 유럽의 7개 국가를 대상으로 시행된 연구에 의하면 소득이 높은 집단은 낮은 집단보다 자가평가 불건강 유병률이 낮았고 [19], Hildebrand와 Van Kerm (2009)에 의해 유럽의 11개 국가에서 이루어진 연구에서도 남녀 모두 소득에 따른 자가평가 불건강 유병률의 불평등을 발견할 수 있었다 [20]. 국내에서는 1989년도부터 1999년도까지 사회통계조사를 분석한 바에 따르면 교육수준에 따른 자가평가 건강수준 불평등이 10년 간 심화되었음이 밝혀진 바 있으며 [21], 김혜련(2005)이 서울 지역을 대상으로 시행한 연구에서도 남녀 모두 교육수준, 소득, 직업 등의 사회경제적 위치에 따른 자가평가 건강수준의 불평등이 보고된 바 있다 [22]. 1998년도와 2001년도 국민건강영양조사 자료를 합산하여 분석한 연구에서 자가평가 건강수준은 경제활동 상태, 가구소득수준과 깊은 관련성을 보였다 [4]. 2005년도 국민건강영양조사 자료를 이용한 연구에서도 성별에 상관없이 사회계층, 교육수준, 소득수

준에 따른 자가평가 불건강 유병률의 불평등이 관찰되었다 [23].

이 연구에서는 도시보다 농촌에서 연령표준화 자가평가 불건강 유병률이 더 높고 소득수준 간 격차는 큰 것으로 나타났다. 또한 수도권과 비수도권, 서울의 강남과 강북 지역에서도 자가평가 불건강 유병률의 격차와 함께 소득수준 간 격차의 차이 또한 관찰되었다. 이와 같은 현상이 나타난 이유는 <Figure 1>에서 볼 수 있듯이, 자가평가 불건강 유병률의 증가에 따라 소득 상위 20% 유병률의 상승폭은 크지 않았던 반면, 소득 하위 20%의 유병률 상승폭은 상대적으로 컸기 때문이다. 자가평가 불건강 유병률이 높은 지역(농촌 지역, 서울의 강북 지역)은 상대적으로 사회경제적 수준이 낮은 지역으로, 해당 지역에서의 소득 하위 20%의 절대 소득수준은 자가평가 불건강 유병률이 낮은 지역(도시 지역, 서울시에서 강남지역)의 소득 하위 20%의 절대 소득수준보다 낮다. 소득 상위 20%의 절대 소득수준 또한 자가평가 불건강 유병률이 낮은 지역에서 더 높았지만 소득 하위 20%에 비해서 지역 간 상대적 격차가 작았고, 가구원 수 보정 연간 가구소득액이 농촌 지역에서도 소득수준을 고려하지 않았을 때의 전체 평균액보다 큰 것으로 나타났다. 이는 <Appendix 5>에서 확인할 수 있다. 소득이 낮을수록 자가평가 불건강 유병률이 높은 점을 고려하면 자가평가 불건강 유병률이 높아짐에 따라 소득 하위 20% 유병률의 상승폭이 상위 20% 유병률의 상승폭보다 더 컸던 것은 구성적 효과(compositional effect)가 일부 원인으로 작용했기 때문이라고 할 수 있다. 건강행태에서도 자가평가 불건강 유병률이 높은 지역의 최하위 소득군 흡연율은 자가평가 불건강 유병률이 낮은 지역의 최하위 소득군에 비해서 더 높은 흡연율을 보였다 [24]. 이러한 차이에는 구성적 효과 뿐만 아니라 사회경제적 지표가 좋지 않은 지역들에서 담배 구매가 더 용이하거

나 [25] 실내 금연 정책의 시행이 늦게 이루어지는 등 [26] 흡연 행태에 영향을 미치는 맥락 효과(contextual effect)가 해당 지역에 작용하기 때문으로 여겨진다. 사회경제적으로 취약한 지역의 저소득계층에서 보이는 높은 자가평가 불건강 유병률에는 주거 환경 [27], 도심 내 휴식시설 [28] 및 보건의료 서비스 접근성 [29] 등의 맥락적 요인도 기여할 것으로 생각된다. 이처럼 구성적 및 맥락적 요인들은 동시에 작용하며, 자가평가 불건강 유병률이 높은 농촌지역이나 강북 지역에 거주하는 낮은 소득계층의 주관적 건강수준에 영향을 미쳤을 것으로 생각된다.

연구 결과에 따르면, 우리나라 시군구 수준에서 연령표준화 자가평가 불건강 유병률과 그 격차의 크기는 상관성을 보였다. 2015년 국민건강보험공단에 의해 이루어진 연구에서 기대수명과 기대수명의 소득수준 간 격차가 높은 상관성을 보인 결과와 유사한 양상이라고 할 수 있다. 우리나라 시군구 수준에서 기대수명과 자가평가 불건강 유병 지표 모두 불평등의 크기가 작은 지역일수록 기대수명과 자가평가 불건강 유병률 또한 낮게 나타남을 알 수 있다. 또한 이 연구에서는 연령표준화 자가평가 불건강 유병률과 기대수명, 그리고 자가평가 불건강 유병률과 기대수명의 소득수준 간 격차가 높은 상관성을 나타냈다. 즉, 우리나라 시군구 수준에서 자가평가 불건강 유병률이 높은 지역일수록 사망률 또한 높고 유병률의 불평등 크기가 큰 지역일수록 사망률에 있어서의 불평등의 크기 또한 큰 경향을 보였다. 이들 결과는 지역 단위에서 건강 불평등의 감소를 통하여 지역 전체의 객관적, 주관적 건강수준이 함께 향상될 가능성이 존재한다는 점을 알려준다.

이 연구는 다음과 같은 제한점을 지닌다. 2008년도부터 2013년도까지 연속형 변수로 측정되던 지역사회 건강조사의 가구소득 측정이 2014년도부터 월 가구소득을 범주형으로 측정하는 것으로 바뀌었다. 이에 따라 가구소득의 정확한 측정에 제한이 있을 수 있다. 특히 600만원 이상의 월 가구소득을 가진 사람들은 "650만원 이상"이라는 단일한 범주로 분류되기 때문에 상위 소득군의 산출에 주의를 기울여야 했다. 그러나 본 연구에서는 성별과 연령을 고려하여 지역별로 소득 5분위를 나눠 각 연도별로 14,700개의 그룹을 나누는 분석 방법을 적용했는데 2014년도 지역사회 건강조사 자료에서 월 가구소득을 '600만원 이상'이라고 응답한 사람의 분율이 20%가 넘는 그룹은 290개 그룹으로 전체의 2.0%를 차지했고 이 290개 그룹에 속한 응답자들의 수는 6,025명으로 2014년도 지역사회 건강조사 응답자 228,712명의 2.6%에 불과하였다. 또한 2014년도 자료를 제외하여 분석하였을 경우에도 전반적인 양상은 비슷하게 나타났다. 하지만 본 연구에 사용한 기대수명 자료가 2014년도까지를 포함하고 있고, 가능한 시군구 단위에서 많은 표본수를 사용하는 것이 보다 안정적인 소득5분위별 유병률을 산출할 수 있기 때문에, 최종 분석에는 2014년도 자료를 포함하였다. 또한 본 연구에서는 유병률의 절대 차이값에 초점을 두고 기술하였기 때문에, 유병률 비라는 상대 지표를 별도로 제시하지는 않았다. 상대지표는 <Appendix Table 3>을 이용하여 계산이 가능하다.

본 연구는 우리나라의 245개 모든 시군구에 존재하는 자가평가 불건강 유병률의 소득수준 간 격차의 크기를 밝혔다. 또한 자가평가 불건강 유병률의 격차와 시군구별 기대수명의 소득수준 간 격차는 높은 상관성을 보였다. 즉, 주관적 건강 상태 지표와 객관적 건강 상태 지표 모두에서 우리나라의 시군구 지역 내에 건강 불평등이 존재한다는 점

을 제시하였다는 점에서 이 연구의 의의를 찾을 수 있다. 자가평가 불건강 유병률의 소득수준 간 격차는 향후 개별 시군구 내 건강 형평성 현황에 관해서 유의미한 정보를 제공하고 이를 지역별 보건정책 수립의 근거로 활용할 수 있을 것으로 보인다. 앞으로는 지역과 사회경제적 위치에 따른 자가평가 건강 불평등의 크기 변이의 원인에 대해서 규명하는 연구가 필요할 것이다.

Volume: 39, Article ID: e2017011,

https://doi.org/10.4178/epih.e2017011

References

1.Idler EL, Benyamini Y. Self-rated health and mortality: a review of twenty-seven community studies. J Health Soc Behav 1997;38:21-37.

2.Jee SH, Ohrr HC, Kim IS. Self-rated health and mortality in elderly: Kangwha cohort, 8-year follow up. Korean J Epidemiol 1994 (Korean);16:172-180.

3.Khang YH, Kim HR. Gender differences in self-rated health and mortality association: role of pain-inducing musculoskeletal disorders. J Womens Health (Larchmt) 2010;19:109-116.

4.Khang YH, Kim HR. Self-rated health and mortality: gender- and age-specific contributions of explanatory factors in South Korea. Int J Public Health 2010;55:279-289.

5.Kaplan GA, Goldberg DE, Everson SA, Cohen RD, Salonen R, Tuomilehto J, et al. Perceived health status and morbidity and mortality: evidence from the Kuopio ischaemic heart disease risk factor study. Int J Epidemiol 1996;25:259-265.

6.Wu S, Wang R, Zhao Y, Ma X, Wu M, Yan X, et al. The relationship between self-rated health and objective health status: a population-based study. BMC Public Health 2013;13

7.Khang YH, Lee SI. Health inequalities policy in Korea: current status and future challenges. J Korean Med Sci 2012;27 Suppl:S33-40.

8.Ministry of Health and Welfare. Health Plan 2010. Seoul: Ministry of Health and Welfare; 2005 (Korean) p. 211

9.Ministry of Health and Welfare. Health Plan 2020. Seoul: Ministry of Health and Welfare; 2011 (Korean) p. 96

10.Ministry of Health and Welfare. Health Plan 2020. Seoul: Ministry of Health and Welfare; 2015 (Korean) p. 36

11.Khang YH, Bahk J, Kang H, Park JH. Income differences in life expectancy at birth in provinces and local districts in South Korea: Findings from National Health Insurance Data, 2009-2014. Seoul: National Health Insurance Service; 2015. (Korean).

12.Khang YH, Yun SC, Cho HJ, Jung-Choi K. The impact of governmental antismoking policy on socioeconomic disparities in cigarette smoking in South Korea. Nicotine Tob Res 2009;11:262-269.

13.Khang YH, Bahk J, Yi N, Yun SC. Age- and cause-specific contributions to income difference in life expectancy at birth: findings from nationally representative data on one million South Koreans. Eur J Public Health 2016;26:242-248.

14.Khang YH, Yang S, Cho HJ, Jung-Choi K, Yun SC. Decomposition of socio-economic differences

in life expectancy at birth by age and cause of death among 4 million South Korean public servants and their dependents. Int J Epidemiol 2010;39:1656-1666.

15.Benyamini Y, Leventhal EA, Leventhal H. Gender differences in processing information for making self-assessments of health. Psychosom Med 2000;62:354-364.

16.Unruh AM. Gender variations in clinical pain experience. Pain 1996;65:123-167.

17.Keogh E. Sex Differences in Pain. Rev Pain 2008;2:4-7.

18.Rollman GB, Lautenbacher S. Sex differences in musculoskeletal pain. Clin J Pain 2001;17:20-24.

19.Mackenbach JP, Martikainen P, Looman CW, Dalstra JA, Kunst AE, Lahelma E, et al. The shape of the relationship between income and self-assessed health: an international study. Int J Epidemiol 2005;34:286-293.

20.Hildebrand V, Van Kerm P. Income inequality and self-rated health status: evidence from the European Community Household Panel. Demography 2009;46:805-825.

21.Khang YH, Lynch JW, Yun S, Lee SI. Trends in socioeconomic health inequalities in Korea: use of mortality and morbidity measures. J Epidemiol Community Health 2004;58:308-314.

22.Kim HR. The relationship of socioeconomic position and health behaviors with morbidity in Seoul, Korea. Health and Social Welfare Review 2005;25:3-35 (Korean).

23.Kim M, Chung W, Lim S, Yoon S, Lee J, Kim E, et al. [Socioeconomic inequity in self-rated health status and contribution of health behavioral factors in Korea]. J Prev Med Public Health 2010;43:50-61.

24.Kim I, Bahk J, Yoon T-H, Khang YH. Income differences in smoking prevalences in 245 districts of South Korea: Patterns by area deprivation and urbanity, 2008-2014. Korean J Prev Med 2017;0

25.Rodriguez D, Carlos HA, Adachi-Mejia AM, Berke EM, Sargent JD. Predictors of tobacco outlet density nationwide: a geographic analysis. Tob Control 2013;22:349-355.

26.National health promotion act. Article 9. 2014. Available from: http://www.law.go.kr/lsInfoP.do?lsiSeq=181391&efYd=20160903#0000.

27.Oh S. Disparities of residential well-being for urban and rural autonomies: Application of homeownership and minimum standard (dissertation). Seoul: Seoul National University 2002 (Korean)

28.Lee KH, Seo JS. A Study on the Regional Disparity between the Major 3 Gangnam Districts and the Major 3 Gangbuk Districts in Seoul. Korean Public Management Review 2009;23:357-381 (Korean).

29.Cho HJ. Equity in health care: current situation in South Korea. J Korean Med Assoc 2013;56:184-194.

Volume: 39, Article ID: e2017011,

https://doi.org/10.4178/epih.e2017011

Volume: 39, Article ID: e2017011,

https://doi.org/10.4178/epih.e2017011

Table 1. Characteristics of study subjects in 245 districts of South Korea: Data from the Community Health Survey, 2008-2014

	Total	Men	Women
No. of study subjects (%)	1,578,189 (100.0)	717,183 (45.4)	861,006 (54.6)
Residential area (%)			
Metropolitan	625,991 (100.0)	284,892 (45.5)	341,099 (54.5)
Urban	440,499 (100.0)	202,102 (45.9)	238,397 (54.1)
Rural	511,699 (100.0)	230,189 (45.0)	281,510 (55.0)
Mean age (SD)	51.3 (16.7)	50.4 (16.1)	52.0 (17.1)
Weighted mean age (SD)	48.2 (18.7)	46.6 (17.8)	49.5 (19.1)
Mean equivalized household income (SD) (10,000 won)	1808.1 (1910.5)	1892.9 (1975.0)	1737.6 (1852.1)
Quintile I (Lowest)	657.8 (403.1)	690.6 (407.6)	630.5 (397.3)
Quintile II	1134.0 (524.9)	1203.1 (508.1)	1076.8 (531.6)
Quintile III	1546.9 (641.1)	1626.2 (618.7)	1480.3 (651.8)
Quintile IV	2093.5 (786.6)	2193.7 (759.5)	2009.8 (799.1)
Quintile V (Highest)	3622.2 (3443.0)	3766.8 (3589.3)	3502.3 (3312.1)
No. of subjects with self-rated poor health (crude prevalences of self-rated poor health, %)	339,708 (21.5)	122,060 (17.0)	217,648 (25.3)
Age-standardized prevalences of self-rated poor health[1] (%) (95% CI)	16.3 (16.3-16.4)	13.7 (13.6-13.8)	18.2 (18.1-18.3)

CI, confidence interval; SD, standard deviation.

[1]Age-standardized prevalences of self-rated poor health were estimated with standard population being 2010 mid-year resident population, after taking into account sample weights of the Community Health Survey.

Volume: 39, Article ID: e2017011,

https://doi.org/10.4178/epih.e2017011

Table 2. Overall age-standardized prevalences of self-rated poor health, self-rated poor health prevalences by income quintiles, and interquintile income gaps in the prevalences of self-rated poor health stratified by gender among 245 local districts of South Korea: Findings from the Community Health Survey , 2008-2014

	Total					Men					Women				
Variable	Median	SD	Min	Max	IQR	Median	SD	Min	Max	IQR	Median	SD	Min	Max	IQR
Overall	15.7%	2.3%	8.1%	21.8%	3.1%p	13.2%	2.2%	6.2%	18.7%	3.2%p	17.4%	2.4%	9.6%	24.4%	3.0%p
Income Q1 (lowest)	22.8%	3.6%	11.0%	31.2%	4.6%p	21.1%	4.1%	9.1%	31.0%	5.4%p	23.9%	3.7%	12.6%	34.2%	4.3%p
Income Q2	16.6%	2.6%	8.2%	24.2%	3.1%p	13.7%	2.8%	5.1%	21.6%	3.5%p	18.5%	2.8%	10.5%	27.7%	3.3%p
Income Q3	14.5%	2.3%	6.7%	22.2%	3.1%p	11.7%	2.2%	6.4%	19.3%	3.1%p	16.4%	2.7%	6.9%	27.3%	3.6%p
Income Q4	12.9%	2.0%	7.1%	20.8%	2.7%p	10.1%	1.9%	5.1%	16.2%	2.5%p	14.9%	2.5%	8.4%	23.8%	3.2%p
Income Q5 (highest)	11.7%	2.0%	6.7%	17.2%	2.7%p	9.4%	1.9%	4.0%	14.7%	2.6%p	13.4%	2.3%	7.4%	20.7%	2.7%p
Q1-Q5	11.1%p	2.7%p	2.8%p	20.1%p	3.6%p	11.2%p	3.4%p	2.7%p	23.5%p	4.3%p	10.4%p	3.0%p	1.5%p	19.2%p	4.3%p

SD, standard deviation; Min, minimum; Max, maximum; IQR, interquartile range

Volume: 39, Article ID: e2017011,

https://doi.org/10.4178/epih.e2017011

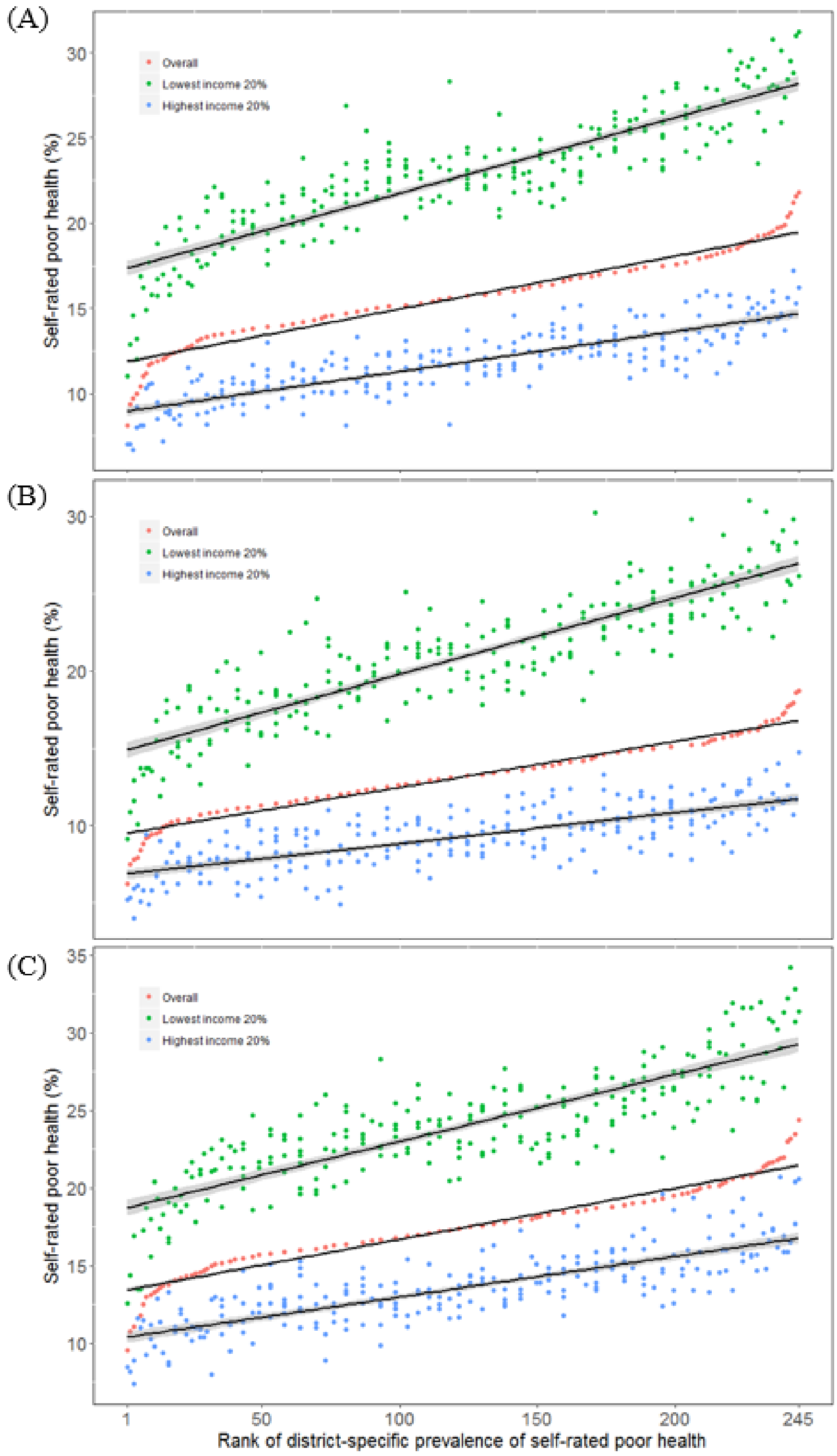

Figure 1. Scatter plots and regression lines of age-standardized prevalence of self-rated poor health in overall, the lowest [Q5] and the highest [Q1] income quintiles on the rank of district-specific

prevalence of self-rated poor health in (A) total, (B) men, and (C) women: Findings from the Community Health Survey, 2008-2014

Volume: 39, Article ID: e2017011,

https://doi.org/10.4178/epih.e2017011

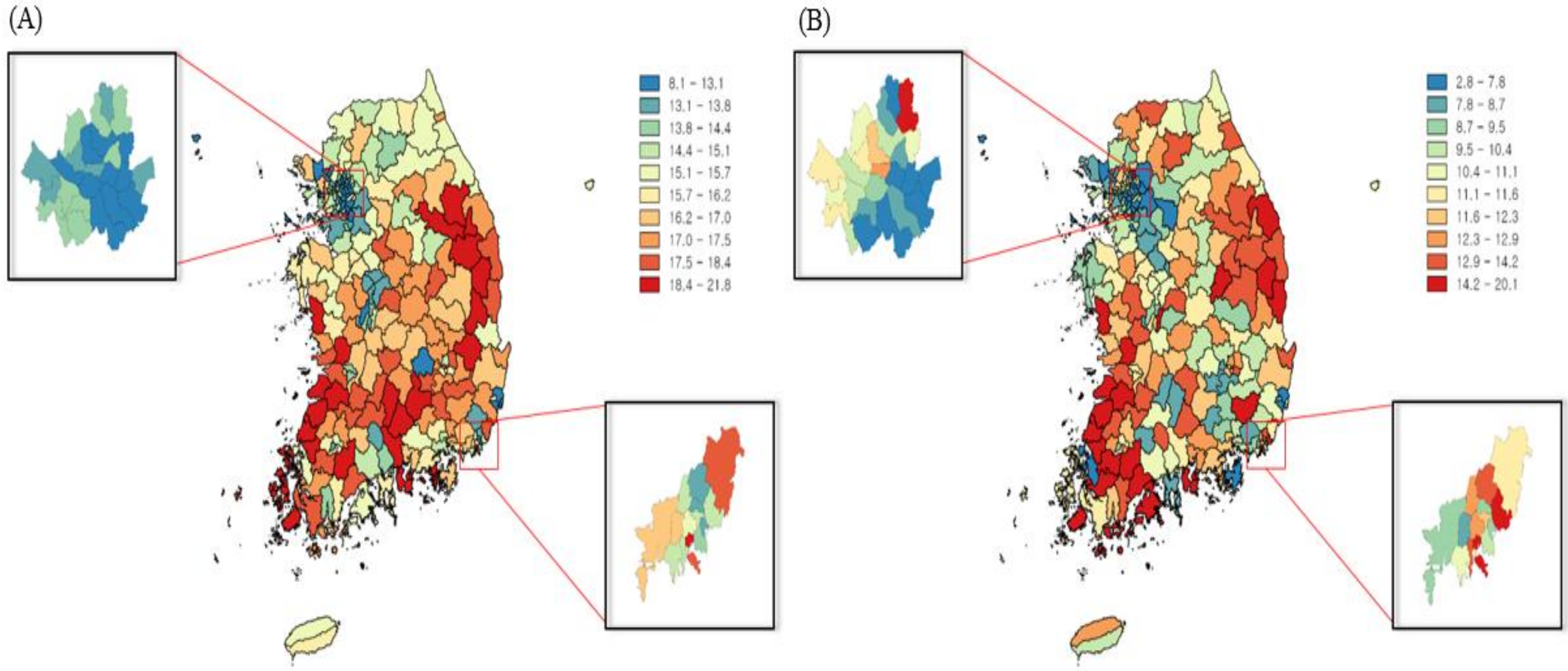

Figure 2. Choropleth maps for (A) the age-standardized prevalences of self-rated poor health and (B) the interquintile income gaps in self-rated poor health in Korea (both genders combined): Findings from the Korea Community Health Survey in Korea, 2008-2014.

Volume: 39, Article ID: e2017011,

https://doi.org/10.4178/epih.e2017011

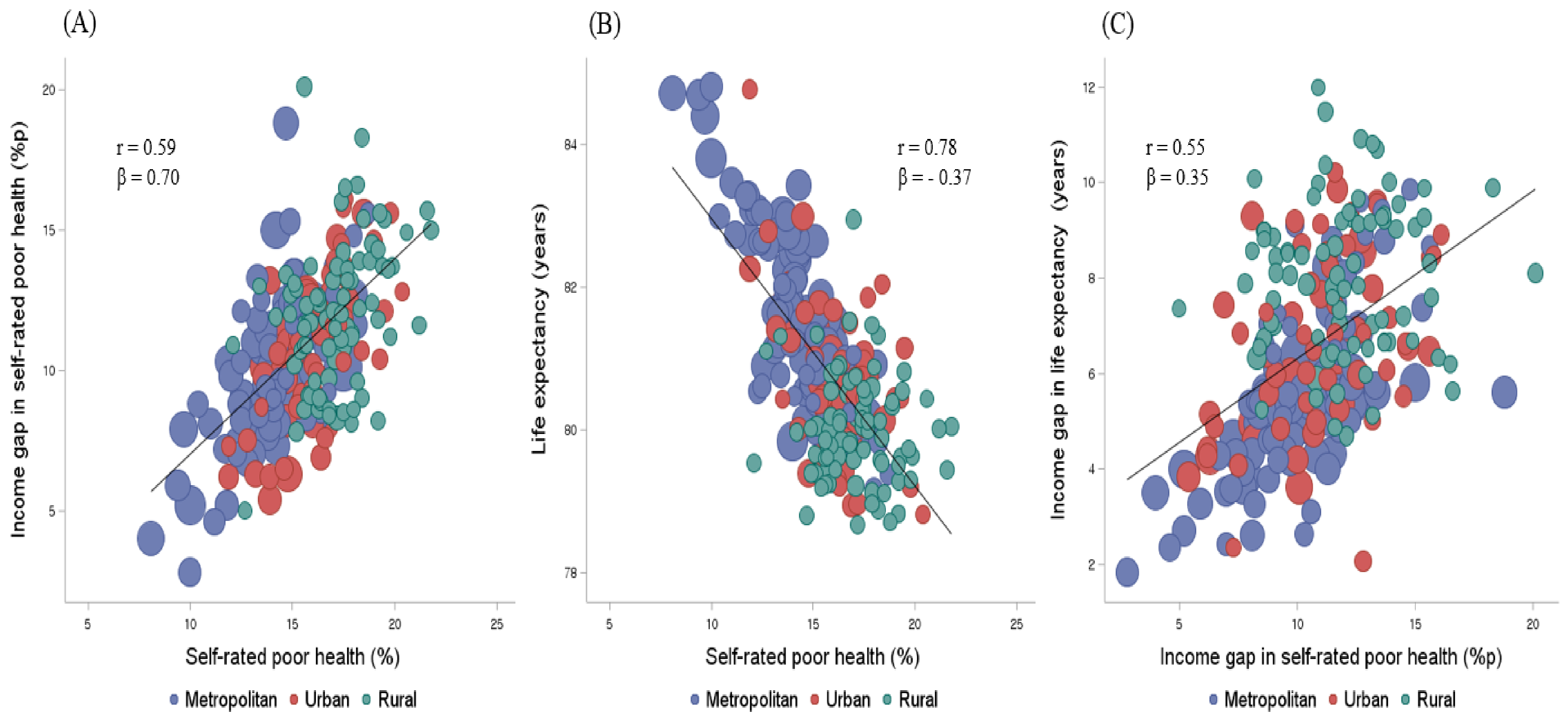

Figure 3. Scatter plots for correlations of age-standardized prevalences of self-rated poor health with (A) interquintile income gaps of self-rated poor health prevalence and (B) life expectancy and of (C) interquintile income gaps in self-rated poor health with life expectancy gap by income in the 245 local

Volume: 39, Article ID: e2017011,

https://doi.org/10.4178/epih.e2017011

districts of South Korea (both sexes combined): Findings from the Community Health Survey in Korea, 2008-2014, and analysis results on life expectancy from the National Health Insurance Service

Volume: 39, Article ID: e2017011,

https://doi.org/10.4178/epih.e2017011

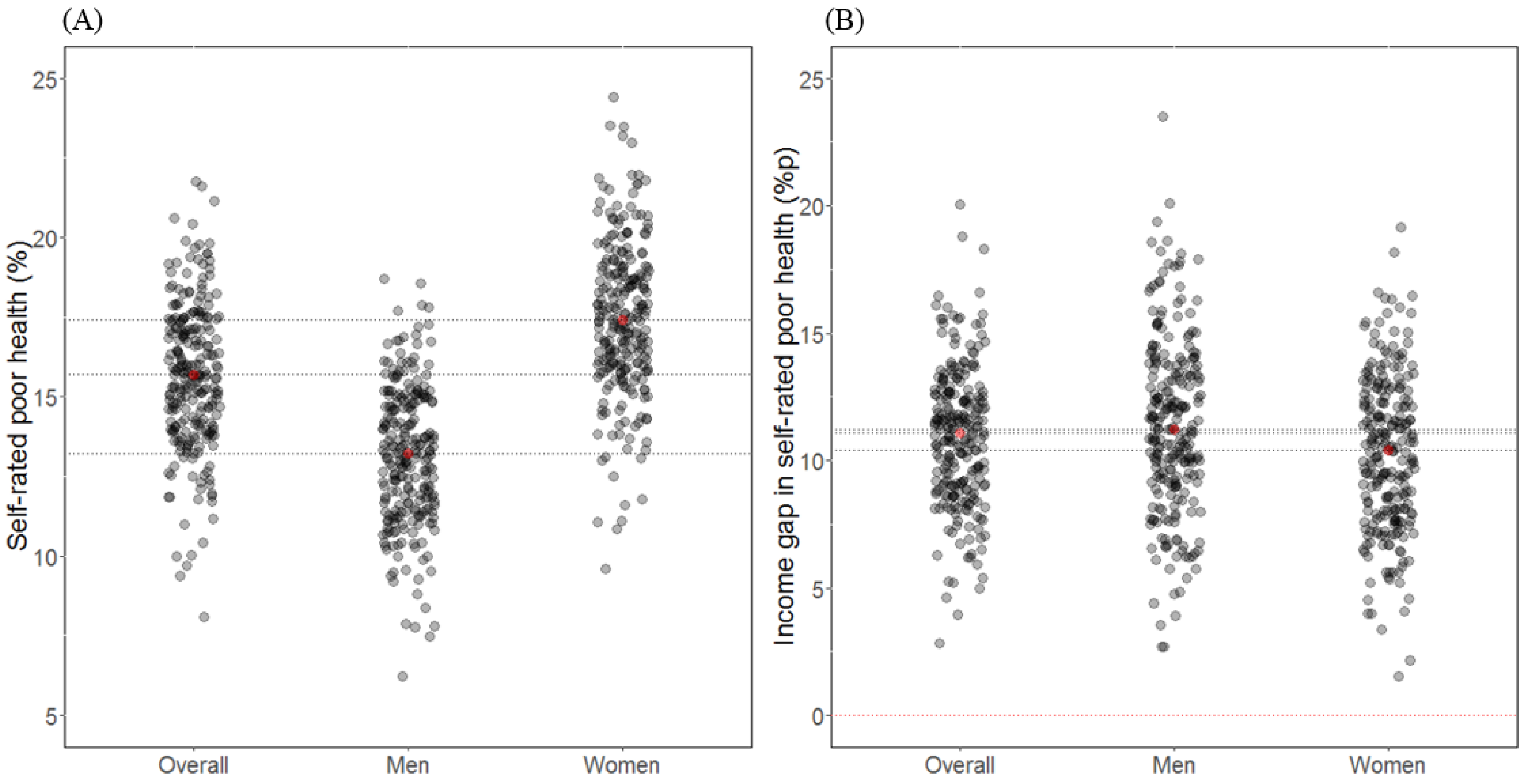

Appendix 2. Distribution of (A) age-standardized prevalences of self-rated poor health, and of (B) interquintile income gaps in self-rated poor health in the 245 local districts in South Korea, Findings from the Community Health Survey, 2008-2014

Volume: 39, Article ID: e2017011,

https://doi.org/10.4178/epih.e2017011

Appendix 2. Ten top and bottom districts in terms of age-standardized prevalences of self-rated poor health in South Korea: Findings from the Community Health Survey, 2008-2014

	Top 10			Bottom 10		
	Province	District	Self-rated poor health (%)	Province	District	Self-rated poor health (%)
Total	Jeollabuk-do	Gochang-gun	21.8	Gyeonggi-do	Bundang-gu	8.1
	Gyeongsangnam-do	Namhae-gun	21.6	Seoul	Seocho-gu	9.4
	Gyeongsangnam-do	Hadong-gun	21.2	Seoul	Gangnam-gu	9.7
	Gyeongsangbuk-do	Yeongyang-gun	20.6	Seoul	Songpa-gu	10.0
	Gangwon-do	Taebaek-si	20.4	Gyeonggi-do	Suji-gu	10.0
	Gangwon-do	Jeongseon-gun	19.9	Seoul	Yongsan-gu	10.4
	Chungcheongnam-do	Boryeong-si	19.8	Gyeonggi-do	Giheung-gu	11.0
	Jeollanam-do	Sinan-gun	19.8	Gyeonggi-do	Yeongtong-gu	11.2
	Gyeongsangbuk-do	Bonghwa-gun	19.7	Gyeonggi-do	Ilsanseo-gu	11.7
	Jeollabuk-do	Buan-gun	19.5	Gyeonggi-do	Dongan-gu	11.8
Men	Gangwon-do	Taebaek-si	18.7	Gyeonggi-do	Bundang-gu	6.2
	Gyeongsangnam-do	Namhae-gun	18.6	Seoul	Seocho-gu	7.5
	Jeollabuk-do	Gochang-gun	17.9	Seoul	Gangnam-gu	7.8
	Gangwon-do	Jeongseon-gun	17.8	Seoul	Songpa-gu	7.8
	Gyeongsangnam-do	Hadong-gun	17.7	Gyeonggi-do	Suji-gu	7.9
	Gyeongsangbuk-do	Yeongyang-gun	17.3	Gyeonggi-do	Giheung-gu	8.4
	Jeollabuk-do	Buan-gun	17.2	Gyeonggi-do	Gwacheon-si	8.8
	Gyeongsangbuk-do	Cheongsong-gun	16.9	Gyeonggi-do	Dongan-gu	9.2
	Gyeongsangnam-do	Sancheong-gun	16.9	Seoul	Mapo-gu	9.3
	Chungcheongnam-do	Boryeong-si	16.8	Gyeonggi-do	Ilsandong-gu	9.4
Women	Jeollabuk-do	Gochang-gun	24.4	Gyeonggi-do	Bundang-gu	9.6
	Gyeongsangnam-do	Namhae-gun	23.5	Seoul	Seocho-gu	10.8
	Gyeongsangnam-do	Hadong-gun	23.5	Seoul	Yongsan-gu	11.1
	Gyeongsangbuk-do	Yeongyang-gun	23.2	Seoul	Gangnam-gu	11.1
	Gyeongsangbuk-do	Bonghwa-gun	23.0	Gyeonggi-do	Suji-gu	11.6
	Chungcheongnam-do	Boryeong-si	22.0	Seoul	Songpa-gu	11.8
	Jeollanam-do	Sinan-gun	22.0	Gyeonggi-do	Yeongtong-gu	12.5
	Gyeongsangnam-do	Tongyeong-si	21.9	Seoul	Seongbuk-gu	13.0
	Gangwon-do	Taebaek-si	21.8	Gyeonggi-do	Giheung-gu	13.1
	Jeollanam-do	Hampyeong-gun	21.7	Gyeonggi-do	Gimpo-si	13.1

Volume: 39, Article ID: e2017011,

https://doi.org/10.4178/epih.e2017011

Volume: 39, Article ID: e2017011,

https://doi.org/10.4178/epih.e2017011

Appendix 3. Ten top and bottom districts in terms of income gaps in self-rated poor health between the lowest and highest income quintiles in South Korea: Findings from the Community Health Survey in South Korea, 2008-2014

	Top 10			Bottom 10		
	Province	District	Interquintile income gap in self-rated poor health (%p)	Province	District	Interquintile income gap in self-rated poor health (%p)
Total	Jeollanam-do	Goheung-gun	20.1	Gyeonggi-do	Suji-gu	2.8
	Busan	Haeundae-gu	18.8	Gyeonggi-do	Bundang-gu	4.0
	Gyeongsangbuk-do	Yecheon-gun	18.3	Gyeonggi-do	Yeongtong-gu	4.6
	Jeollanam-do	Boseong-gun	16.6	Incheon	Ongjin-gun	5.0
	Gyeongsangbuk-do	Yeongdeok-gun	16.5	Seoul	Songpa-gu	5.2
	Jeollanam-do	Naju-si	16.1	Gyeonggi-do	Dongan-gu	5.2
	Jeollanam-do	Wando-gun	16.0	Gyeonggi-do	Gwangmyeong-si	5.4
	Gangwon-do	Samcheok-si	15.8	Seoul	Seocho-gu	5.9
	Gyeongsangnam-do	Namhae-gun	15.7	Gyeonggi-do	Gimpo-si	6.2
	Chungcheongnam-do	Boryeong-si	15.6	Gyeonggi-do	Osan-si	6.2
Men	Jeollanam-do	Goheung-gun	23.5	Gyeonggi-do	Yeongtong-gu	2.7
	Jeollanam-do	Wando-gun	20.1	Gyeonggi-do	Bundang-gu	2.7
	Jeollanam-do	Yeonggwang-gun	19.4	Seoul	Seongdong-gu	3.6
	Jeollabuk-do	Gochang-gun	18.6	Gyeonggi-do	Dongan-gu	3.9
	Jeollanam-do	Boseong-gun	18.6	Gyeonggi-do	Cheoin-gu	4.4
	Gyeongsangnam-do	Miryang-si	18.2	Incheon	Yeonsu-gu	4.8
	Jeollabuk-do	Imsil-gun	18.1	Gyeonggi-do	Suji-gu	4.8
	Busan	Haeundae-gu	17.9	Busan	Sasang-gu	5.4
	Jeollanam-do	Naju-si	17.8	Gyeonggi-do	Yeoju-gun	5.7
	Gyeongsangbuk-do	Yecheon-gun	17.7	Gyeonggi-do	Gunpo-si	5.7
Women	Busan	Haeundae-gu	19.2	Gyeonggi-do	Suji-gu	1.5
	Jeollanam-do	Goheung-gun	18.2	Gyeongsangbuk-do	Cheongdo-gun	2.2
	Gyeongsangbuk-do	Uljin-gun	16.6	Gyeonggi-do	Gimpo-si	3.4
	Jeollanam-do	Naju-si	16.5	Gyeonggi-do	Ilsanseo-gu	4.0
	Jeollanam-do	Boseong-gun	16.4	Gyeonggi-do	Bundang-gu	4.0
	Jeollanam-do	Hwasun-gun	16.3	Gyeonggi-do	Osan-si	4.1
	Jeollanam-do	Hampyeong-gun	16.0	Gyeongsangnam-do	Changnyeong-gun	4.5
	Gyeongsangbuk-do	Bonghwa-gun	15.8	Seoul	Songpa-gu	4.6
	Jeollabuk-do	Iksan-si	15.8	Gyeonggi-do	Dongan-gu	5.2
	Jeollanam-do	Yeonggwang-gun	15.4	Gyeonggi-do	Yeongtong-gu	5.2

Volume: 39, Article ID: e2017011,

https://doi.org/10.4178/epih.e2017011

Volume: 39, Article ID: e2017011,

https://doi.org/10.4178/epih.e2017011

Appendix 4. Age-standardized prevalences of self-rated poor health, self-rated poor health prevalences according to income quintiles, and income gaps in self-rated poor health (between the highest [Q5] and the lowest [Q1] income quintiles) in 245 local districts of South Korea: Findings from the Community Health Survey in South Korea, 2008-2014

Gender	Provinces	Districts	Overall	Q1 (Lowest)	Q2	Q3	Q4	Q5 (Highest)	Q1-Q5	Urbanity
Total	Seoul	Jongno-gu	12.5 (11.7,13.4)	20.3 (17.8,22.7)	12.6 (10.8,14.5)	12.2 (10.3,14.1)	9.8 (8.1,11.4)	8.2 (6.7,9.7)	12.1 (7.1,13.1)	Metropolitan
Total	Seoul	Jung-gu	13.5 (12.6,14.4)	21.7 (19.1,24.2)	14.5 (12.5,16.6)	12.7 (10.8,14.5)	10.2 (8.5,11.9)	9.2 (7.5,10.8)	12.5 (9.2,15.4)	Metropolitan
Total	Seoul	Yongsan-gu	10.4 (9.7,11.2)	16.9 (14.6,19.2)	9.8 (8.1,11.5)	8.8 (7.2,10.4)	8.7 (7.1,10.3)	8.1 (6.6,9.6)	8.8 (8.2,14.4)	Metropolitan
Total	Seoul	Seongdong-gu	12.8 (11.9,13.8)	16.3 (14.0,18.6)	14.3 (12.2,16.3)	12.9 (10.8,14.9)	11.2 (9.3,13.0)	9.6 (7.8,11.4)	6.7 (2.4,7.8)	Metropolitan
Total	Seoul	Gwangjin-gu	12.3 (11.3,13.2)	16.4 (14.0,18.8)	13.5 (11.3,15.6)	11.5 (9.5,13.5)	11.2 (9.3,13.0)	9.0 (7.2,10.8)	7.4 (5.8,11.4)	Metropolitan
Total	Seoul	Dongdaemun-gu	14.0 (13.1,14.9)	18.9 (16.5,21.4)	15.0 (13.0,17.1)	13.3 (11.3,15.2)	12.5 (10.5,14.4)	10.3 (8.5,12.1)	8.6 (5.5,11.3)	Metropolitan
Total	Seoul	Jungnang-gu	13.1 (12.2,14.0)	19.8 (17.3,22.4)	14.2 (12.1,16.2)	11.6 (9.7,13.4)	11.3 (9.4,13.2)	8.8 (7.1,10.5)	11.0 (8.1,13.9)	Metropolitan
Total	Seoul	Seongbuk-gu	11.9 (11.1,12.8)	18.8 (16.4,21.2)	11.1 (9.3,12.9)	11.0 (9.2,12.7)	10.4 (8.6,12.2)	8.5 (6.9,10.1)	10.3 (7.1,12.9)	Metropolitan
Total	Seoul	Gangbuk-gu	13.9 (13.0,14.8)	18.4 (16.0,20.8)	16.4 (14.2,18.5)	12.9 (11.0,14.8)	11.2 (9.4,12.9)	10.4 (8.7,12.1)	8.0 (4.7,10.7)	Metropolitan
Total	Seoul	Dobong-gu	13.8 (12.9,14.7)	19.4 (17.0,21.9)	14.5 (12.5,16.6)	13.0 (11.0,14.9)	10.8 (9.0,12.5)	11.7 (9.8,13.6)	7.7 (4.9,11.1)	Metropolitan
Total	Seoul	Nowon-gu	14.2 (13.2,15.1)	23.8 (21.0,26.5)	15.8 (13.7,18.0)	12.3 (10.4,14.3)	10.1 (8.3,11.8)	8.8 (7.1,10.5)	15.0 (10.0,16.0)	Metropolitan
Total	Seoul	Eunpyeong-gu	14.3 (13.3,15.2)	20.7 (18.2,23.3)	15.7 (13.5,17.9)	13.6 (11.6,15.6)	11.4 (9.6,13.2)	10.1 (8.3,11.8)	10.6 (7.1,13.1)	Metropolitan
Total	Seoul	Seodaemun-gu	13.7 (12.8,14.6)	20.2 (17.7,22.7)	14.9 (12.8,17.0)	12.0 (10.1,13.9)	12.8 (10.9,14.7)	8.8 (7.2,10.4)	11.4 (8.1,13.9)	Metropolitan
Total	Seoul	Mapo-gu	12.0 (11.1,12.9)	17.0 (14.7,19.4)	15.0 (12.8,17.2)	11.8 (9.9,13.8)	9.1 (7.4,10.8)	7.2 (5.7,8.8)	9.8 (6.9,13.1)	Metropolitan
Total	Seoul	Yangcheon-gu	13.5 (12.6,14.5)	21.5 (18.7,24.2)	13.5 (11.4,15.6)	11.6 (9.6,13.5)	11.0 (9.0,12.9)	10.7 (8.8,12.6)	10.8 (6.0,12.4)	Metropolitan
Total	Seoul	Gangseo-gu	13.7 (12.7,14.6)	20.7 (18.1,23.4)	15.8 (13.6,18.0)	12.6 (10.6,14.6)	10.1 (8.3,11.9)	9.4 (7.7,11.2)	11.3 (7.5,13.3)	Metropolitan
Total	Seoul	Guro-gu	14.2 (13.3,15.2)	22.0 (19.2,24.7)	13.5 (11.4,15.6)	14.1 (12.1,16.2)	11.2 (9.3,13.1)	10.7 (8.8,12.6)	11.3 (7.6,13.8)	Metropolitan
Total	Seoul	Geumcheon-gu	14.4 (13.5,15.3)	21.0 (18.4,23.6)	15.3 (13.1,17.4)	13.8 (11.7,15.8)	10.8 (9.0,12.6)	11.4 (9.5,13.3)	9.6 (4.5,10.5)	Metropolitan
Total	Seoul	Yeongdeungpo-gu	13.9 (13.0,14.9)	21.1 (18.5,23.7)	14.8 (12.7,17.0)	12.7 (10.7,14.6)	10.1 (8.3,11.8)	11.0 (9.1,12.9)	10.1 (7.4,13.4)	Metropolitan
Total	Seoul	Dongjak-gu	14.3 (13.3,15.2)	19.0 (16.5,21.5)	15.1 (12.9,17.3)	12.6 (10.6,14.6)	14.0 (12.0,16.1)	10.9 (9.1,12.7)	8.1 (3.9,9.9)	Metropolitan
Total	Seoul	Gwanak-gu	14.2 (13.3,15.2)	18.7 (16.1,21.3)	15.7 (13.4,18.0)	14.3 (12.1,16.4)	11.4 (9.4,13.4)	11.4 (9.4,13.4)	7.3 (5.4,11.2)	Metropolitan
Total	Seoul	Seocho-gu	9.4 (8.6,10.2)	12.9 (10.8,15.0)	11.6 (9.6,13.6)	8.8 (7.1,10.5)	7.1 (5.6,8.6)	7.0 (5.4,8.6)	5.9 (3.7,8.9)	Metropolitan
Total	Seoul	Gangnam-gu	9.7 (8.8,10.5)	14.6 (12.3,17.0)	10.5 (8.5,12.4)	8.8 (7.1,10.6)	8.0 (6.4,9.7)	6.7 (5.2,8.3)	7.9 (5.8,11.2)	Metropolitan
Total	Seoul	Songpa-gu	10.0 (9.2,10.9)	13.2 (11.0,15.4)	9.4 (7.6,11.2)	10.0 (8.1,11.9)	9.6 (7.8,11.3)	8.0 (6.3,9.7)	5.2 (4.0,9.2)	Metropolitan
Total	Seoul	Gangdong-gu	13.3 (12.3,14.2)	17.6 (15.1,20.0)	16.0 (13.8,18.3)	12.1 (10.1,14.1)	10.7 (8.8,12.7)	9.9 (8.0,11.9)	7.7 (5.2,11.6)	Metropolitan
Total	Busan	Jung-gu	15.0 (14.1,15.9)	21.4 (18.9,23.9)	16.8 (14.7,18.8)	15.5 (13.4,17.5)	12.0 (10.3,13.8)	9.8 (8.2,11.4)	11.6 (7.9,14.1)	Metropolitan
Total	Busan	Seo-gu	15.1 (14.2,16.0)	24.2 (21.6,26.8)	14.9 (13.0,16.9)	13.6 (11.7,15.5)	12.3 (10.6,14.1)	10.6 (8.9,12.3)	13.6 (8.8,15.2)	Metropolitan
Total	Busan	Dong-gu	18.7 (17.7,19.7)	29.4 (26.5,32.3)	18.6 (16.3,20.8)	16.6 (14.5,18.6)	15.2 (13.2,17.2)	13.8 (11.9,15.7)	15.6 (11.3,18.1)	Metropolitan
Total	Busan	Yeongdo-gu	18.0 (17.0,18.9)	27.9 (25.1,30.8)	18.8 (16.6,21.1)	16.4 (14.3,18.4)	14.1 (12.2,16.0)	13.1 (11.3,14.9)	14.8 (10.6,17.4)	Metropolitan
Total	Busan	Busanjin-gu	15.2 (14.2,16.1)	23.2 (20.6,25.8)	16.4 (14.2,18.6)	13.5 (11.5,15.5)	12.2 (10.4,14.0)	10.6 (8.8,12.3)	12.6 (10.0,16.2)	Metropolitan
Total	Busan	Dongnae-gu	13.5 (12.6,14.4)	19.2 (16.9,21.5)	14.2 (12.1,16.2)	11.8 (10.0,13.6)	12.0 (10.2,13.8)	10.2 (8.4,12.0)	9.0 (6.9,12.7)	Metropolitan
Total	Busan	Nam-gu	14.4 (13.5,15.3)	19.9 (17.4,22.3)	15.7 (13.6,17.8)	14.2 (12.2,16.2)	12.5 (10.7,14.4)	10.1 (8.4,11.9)	9.8 (7.5,13.5)	Metropolitan

Volume: 39, Article ID: e2017011,

https://doi.org/10.4178/epih.e2017011

Total	Busan	Buk-gu	14.9 (13.9,15.9)	23.6 (20.8,26.4)	14.8 (12.6,17.0)	14.8 (12.6,17.0)	10.4 (8.6,12.2)	11.2 (9.2,13.2)	12.4 (10.9,17.1)	Metropolitan
Total	Busan	Haeundae-gu	14.7 (13.8,15.7)	26.9 (24.1,29.8)	14.4 (12.3,16.5)	12.3 (10.4,14.2)	12.3 (10.3,14.2)	8.1 (6.5,9.6)	18.8 (15.3,21.5)	Metropolitan
Total	Busan	Saha-gu	14.6 (13.6,15.5)	22.8 (20.1,25.5)	14.7 (12.6,16.8)	12.5 (10.5,14.4)	11.5 (9.6,13.4)	11.7 (9.8,13.6)	11.1 (8.1,14.5)	Metropolitan
Total	Busan	Geumjeong-gu	13.3 (12.4,14.2)	21.5 (18.9,24.1)	14.5 (12.5,16.5)	12.5 (10.6,14.4)	10.5 (8.8,12.3)	8.2 (6.6,9.7)	13.3 (10.6,16.4)	Metropolitan
Total	Busan	Gangseo-gu	16.8 (15.9,17.8)	23.0 (20.4,25.5)	18.1 (15.9,20.3)	15.3 (13.2,17.3)	14.2 (12.2,16.1)	13.8 (11.8,15.7)	9.2 (5.7,12.1)	Metropolitan
Total	Busan	Yeonje-gu	15.1 (14.1,16.0)	24.7 (21.9,27.4)	14.7 (12.7,16.8)	12.6 (10.6,14.5)	11.2 (9.5,13.0)	12.4 (10.4,14.3)	12.3 (8.6,15.0)	Metropolitan
Total	Busan	Suyeong-gu	13.7 (12.9,14.6)	20.0 (17.6,22.3)	15.0 (12.9,17.0)	11.2 (9.5,13.0)	11.9 (10.1,13.7)	10.6 (8.9,12.3)	9.4 (6.2,12.4)	Metropolitan
Total	Busan	Sasang-gu	16.4 (15.4,17.4)	22.3 (19.6,25.1)	17.0 (14.7,19.4)	15.1 (12.9,17.3)	14.0 (11.9,16.2)	13.7 (11.5,15.9)	8.6 (4.8,11.2)	Metropolitan
Total	Busan	Gijang-gun	17.9 (16.9,18.9)	23.8 (21.1,26.4)	20.6 (18.2,23.1)	17.3 (15.1,19.4)	15.6 (13.5,17.6)	12.5 (10.6,14.3)	11.3 (7.7,14.3)	Rural
Total	Daegu	Jung-gu	17.0 (16.0,18.0)	25.5 (22.8,28.3)	17.9 (15.8,20.1)	15.4 (13.4,17.5)	13.4 (11.6,15.3)	12.8 (10.9,14.7)	12.7 (9.2,15.8)	Metropolitan
Total	Daegu	Dong-gu	18.1 (17.1,19.1)	25.6 (22.8,28.4)	18.3 (16.0,20.6)	17.8 (15.6,20.0)	16.2 (14.0,18.4)	12.9 (10.9,14.8)	12.7 (9.4,15.8)	Metropolitan
Total	Daegu	Seo-gu	18.2 (17.2,19.2)	25.3 (22.5,28.1)	18.4 (16.1,20.8)	16.1 (14.0,18.3)	17.3 (15.1,19.6)	13.7 (11.7,15.7)	11.6 (5.6,12.2)	Metropolitan
Total	Daegu	Nam-gu	15.8 (14.9,16.8)	22.8 (20.2,25.4)	17.5 (15.3,19.8)	15.0 (13.0,17.0)	12.9 (11.0,14.8)	11.1 (9.3,12.9)	11.7 (9.5,15.7)	Metropolitan
Total	Daegu	Buk-gu	14.7 (13.7,15.7)	22.5 (19.7,25.3)	15.6 (13.3,17.8)	14.9 (12.7,17.1)	10.8 (9.0,12.7)	10.1 (8.3,12.0)	12.4 (10.5,16.7)	Metropolitan
Total	Daegu	Suseong-gu	15.1 (14.2,16.1)	23.7 (21.0,26.5)	16.1 (13.8,18.3)	14.5 (12.4,16.5)	11.5 (9.6,13.4)	10.3 (8.5,12.1)	13.4 (9.1,15.3)	Metropolitan
Total	Daegu	Dalseo-gu	16.0 (14.9,17.0)	23.6 (20.8,26.3)	18.3 (15.8,20.9)	13.9 (11.8,16.1)	12.9 (10.9,15.0)	11.4 (9.4,13.4)	12.2 (8.9,15.1)	Metropolitan
Total	Daegu	Dalseong-gun	17.2 (16.2,18.2)	23.2 (20.5,25.9)	18.3 (15.9,20.6)	16.1 (13.9,18.3)	13.8 (11.7,15.8)	14.9 (12.7,17.0)	8.3 (6.9,13.3)	Rural
Total	Incheon	Jung-gu	14.7 (13.8,15.6)	21.2 (18.7,23.8)	14.5 (12.5,16.5)	14.7 (12.6,16.7)	11.7 (9.9,13.5)	11.5 (9.6,13.3)	9.7 (7.4,14.6)	Metropolitan
Total	Incheon	Dong-gu	14.1 (13.3,15.0)	19.9 (17.4,22.3)	14.9 (12.9,17.0)	14.3 (12.3,16.3)	11.1 (9.4,12.8)	10.9 (9.1,12.6)	9.0 (5.3,11.3)	Metropolitan
Total	Incheon	Nam-gu	12.6 (11.7,13.4)	19.0 (16.5,21.5)	12.4 (10.4,14.3)	11.2 (9.3,13.0)	10.9 (9.1,12.7)	9.9 (8.2,11.7)	9.1 (5.6,11.4)	Metropolitan
Total	Incheon	Yeonsu-gu	13.9 (12.9,15.0)	21.1 (18.2,23.9)	14.5 (12.1,16.8)	12.8 (10.6,14.9)	11.6 (9.6,13.7)	10.3 (8.3,12.3)	10.8 (6.9,13.3)	Metropolitan
Total	Incheon	Namdong-gu	15.0 (14.0,16.0)	21.5 (18.7,24.3)	15.4 (13.1,17.8)	15.1 (12.8,17.4)	10.6 (8.7,12.5)	12.3 (10.3,14.4)	9.2 (7.7,14.3)	Metropolitan
Total	Incheon	Bupyeong-gu	16.0 (14.9,17.0)	24.3 (21.4,27.3)	16.0 (13.7,18.4)	15.5 (13.2,17.7)	12.9 (10.9,14.9)	11.8 (9.8,13.8)	12.5 (9.2,16.2)	Metropolitan
Total	Incheon	Gyeyang-gu	15.7 (14.6,16.8)	22.3 (19.3,25.3)	17.5 (14.8,20.1)	13.7 (11.5,15.9)	12.9 (10.7,15.1)	12.6 (10.4,14.8)	9.7 (6.3,13.1)	Metropolitan
Total	Incheon	Seo-gu	16.2 (15.1,17.3)	21.9 (19.0,24.9)	16.2 (13.7,18.6)	15.5 (13.0,17.9)	14.3 (12.0,16.6)	13.5 (11.2,15.8)	8.4 (5.5,12.7)	Metropolitan
Total	Incheon	Ganghwa-gun	16.3 (15.3,17.2)	22.0 (19.5,24.6)	17.7 (15.5,19.9)	15.4 (13.5,17.4)	12.7 (10.9,14.4)	13.4 (11.5,15.4)	8.6 (4.3,10.7)	Rural
Total	Incheon	Ongjin-gun	12.7 (11.9,13.6)	16.5 (14.3,18.7)	12.7 (10.9,14.6)	11.7 (9.9,13.5)	11.2 (9.5,12.9)	11.5 (9.7,13.3)	5.0 (3.0,9.8)	Rural
Total	Gwangju	Dong-gu	15.1 (14.2,16.0)	23.4 (20.7,26.0)	16.9 (14.8,19.1)	13.5 (11.6,15.4)	11.8 (10.1,13.6)	10.5 (8.8,12.2)	12.9 (10.3,16.5)	Metropolitan
Total	Gwangju	Seo-gu	13.8 (12.8,14.7)	19.7 (17.2,22.3)	15.5 (13.2,17.8)	11.9 (9.9,13.9)	11.7 (9.8,13.7)	10.5 (8.6,12.3)	9.2 (6.4,12.2)	Metropolitan
Total	Gwangju	Nam-gu	15.3 (14.4,16.3)	23.0 (20.4,25.7)	16.5 (14.3,18.7)	14.2 (12.3,16.2)	12.1 (10.2,14.0)	11.4 (9.6,13.2)	11.6 (8.9,15.1)	Metropolitan
Total	Gwangju	Buk-gu	15.7 (14.7,16.7)	23.1 (20.4,25.8)	15.3 (13.1,17.5)	15.3 (13.1,17.5)	13.5 (11.5,15.6)	11.5 (9.5,13.4)	11.6 (7.2,13.2)	Metropolitan
Total	Gwangju	Gwangsan-gu	15.8 (14.7,16.8)	23.9 (20.9,27.0)	17.2 (14.7,19.7)	14.7 (12.4,17.0)	11.7 (9.6,13.8)	11.6 (9.5,13.8)	12.3 (8.7,15.5)	Metropolitan
Total	Daejeon	Dong-gu	14.9 (14.0,15.8)	25.4 (22.6,28.3)	16.0 (13.9,18.1)	12.0 (10.1,13.8)	11.5 (9.7,13.4)	10.1 (8.4,11.8)	15.3 (10.7,16.9)	Metropolitan
Total	Daejeon	Jung-gu	13.3 (12.4,14.2)	20.7 (18.2,23.3)	13.3 (11.4,15.3)	12.6 (10.7,14.6)	11.5 (9.7,13.3)	8.9 (7.3,10.5)	11.8 (7.8,13.8)	Metropolitan
Total	Daejeon	Seo-gu	14.0 (13.0,15.0)	21.2 (18.3,24.0)	15.1 (12.8,17.3)	12.8 (10.6,15.0)	11.7 (9.7,13.7)	9.7 (7.8,11.6)	11.5 (9.8,15.8)	Metropolitan
Total	Daejeon	Yuseong-gu	12.4 (11.4,13.4)	17.3 (14.7,19.8)	15.0 (12.6,17.4)	9.9 (8.0,11.8)	10.8 (8.7,12.9)	8.5 (6.5,10.4)	8.8 (5.9,11.7)	Metropolitan
Total	Daejeon	Daedeok-gu	13.9 (13.0,14.9)	19.4 (16.8,22.0)	15.7 (13.5,17.9)	12.0 (10.0,14.0)	12.3 (10.2,14.3)	10.4 (8.5,12.3)	9.0 (5.7,13.1)	Metropolitan

Total	Ulsan	Jung-gu	15.7 (14.7,16.8)	22.0 (19.3,24.7)	15.4 (13.1,17.6)	13.9 (11.7,16.2)	13.7 (11.6,15.8)	13.7 (11.5,15.9)	8.3 (5.7,12.1)	Metropolitan
Total	Ulsan	Nam-gu	13.1 (12.1,14.1)	17.8 (14.9,20.7)	13.6 (11.3,15.9)	12.3 (10.0,14.5)	11.5 (9.4,13.6)	10.8 (8.7,13.0)	7.0 (4.9,10.7)	Metropolitan
Total	Ulsan	Dong-gu	12.5 (11.5,13.5)	19.6 (16.7,22.5)	14.4 (11.9,16.9)	11.0 (8.9,13.1)	8.9 (7.0,10.8)	9.3 (7.2,11.4)	10.3 (7.8,13.4)	Metropolitan
Total	Ulsan	Buk-gu	12.3 (11.3,13.4)	15.8 (13.1,18.5)	14.2 (11.8,16.6)	12.6 (10.3,15.0)	10.1 (8.0,12.2)	8.8 (6.8,10.9)	7.0 (4.3,10.3)	Metropolitan
Total	Ulsan	Ulju-gun	17.4 (16.4,18.5)	25.4 (22.6,28.3)	18.7 (16.3,21.1)	15.3 (13.2,17.5)	13.8 (11.8,15.8)	14.6 (12.5,16.7)	10.8 (7.8,14.4)	Rural
Total	Sejong	Sejong	17.0 (16.1,18.0)	24.0 (21.4,26.7)	18.2 (16.0,20.4)	16.9 (14.8,19.1)	14.0 (12.1,15.9)	12.4 (10.6,14.3)	11.6 (8.1,14.5)	Metropolitan
Total	Gyeonggi-do	Jangan-gu	13.6 (12.7,14.6)	18.5 (16.0,21.0)	15.8 (13.5,18.0)	12.4 (10.3,14.4)	11.7 (9.7,13.7)	9.7 (7.8,11.5)	8.8 (6.1,12.1)	Metropolitan
Total	Gyeonggi-do	Gwonseon-gu	14.0 (12.9,15.0)	20.2 (17.4,22.9)	14.1 (11.8,16.4)	13.1 (11.0,15.3)	11.8 (9.7,13.9)	10.7 (8.7,12.8)	9.5 (5.7,11.7)	Metropolitan
Total	Gyeonggi-do	Paldal-gu	14.1 (13.2,15.1)	20.4 (17.7,23.0)	14.7 (12.5,16.9)	12.1 (10.1,14.1)	11.9 (9.9,13.9)	11.8 (9.7,13.8)	8.6 (5.5,12.5)	Metropolitan
Total	Gyeonggi-do	Yeongtong-gu	11.2 (10.1,12.2)	14.9 (12.1,17.6)	10.5 (8.2,12.7)	9.6 (7.4,11.7)	10.8 (8.5,13.2)	10.3 (8.0,12.7)	4.6 (2.3,10.1)	Metropolitan
Total	Gyeonggi-do	Sujeong-gu	15.7 (14.6,16.7)	22.0 (19.3,24.8)	16.8 (14.5,19.1)	14.1 (11.9,16.3)	13.6 (11.6,15.7)	11.8 (9.8,13.9)	10.2 (7.8,14.6)	Metropolitan
Total	Gyeonggi-do	Jungwon-gu	16.6 (15.5,17.6)	24.1 (21.2,26.9)	17.3 (15.0,19.7)	14.6 (12.4,16.8)	13.8 (11.6,15.9)	13.3 (11.2,15.4)	10.8 (7.7,14.3)	Metropolitan
Total	Gyeonggi-do	Bundang-gu	8.1 (7.3,8.8)	11.0 (9.1,13.0)	8.2 (6.5,9.8)	6.7 (5.2,8.1)	7.5 (5.9,9.1)	7.0 (5.4,8.6)	4.0 (0.9,6.1)	Metropolitan
Total	Gyeonggi-do	Uijeongbu-si	15.2 (14.2,16.1)	23.4 (20.6,26.1)	17.0 (14.7,19.3)	12.7 (10.7,14.7)	11.9 (10.0,13.8)	11.5 (9.6,13.3)	11.9 (8.2,14.6)	Urban
Total	Gyeonggi-do	Manan-gu	13.6 (12.7,14.6)	20.1 (17.5,22.7)	14.8 (12.6,17.0)	12.0 (10.1,13.9)	11.2 (9.3,13.1)	10.6 (8.7,12.4)	9.5 (7.4,13.4)	Metropolitan
Total	Gyeonggi-do	Dongan-gu	11.8 (10.9,12.7)	15.8 (13.4,18.3)	12.1 (10.0,14.3)	11.3 (9.4,13.2)	9.2 (7.4,11.0)	10.6 (8.7,12.6)	5.2 (0.6,6.4)	Metropolitan
Total	Gyeonggi-do	Wonmi-gu	14.1 (13.0,15.1)	19.9 (17.1,22.6)	15.1 (12.7,17.6)	12.2 (10.2,14.3)	12.0 (9.9,14.1)	11.6 (9.5,13.6)	8.3 (4.6,10.8)	Metropolitan
Total	Gyeonggi-do	Sosa-gu	14.2 (13.2,15.2)	20.0 (17.3,22.7)	14.8 (12.6,17.0)	13.3 (11.2,15.4)	12.7 (10.6,14.7)	10.8 (8.9,12.7)	9.2 (7.4,13.6)	Metropolitan
Total	Gyeonggi-do	Ojeong-gu	15.2 (14.2,16.2)	23.7 (20.8,26.5)	15.6 (13.3,17.8)	11.7 (9.7,13.6)	12.1 (10.1,14.1)	13.1 (10.9,15.2)	10.6 (6.2,12.8)	Metropolitan
Total	Gyeonggi-do	Gwangmyeong-si	13.9 (12.9,14.8)	18.4 (15.8,20.9)	15.2 (13.0,17.4)	12.2 (10.2,14.3)	10.7 (8.8,12.6)	13.0 (10.8,15.1)	5.4 (5.3,13.1)	Urban
Total	Gyeonggi-do	Pyeongtaek-si	14.9 (14.2,15.5)	22.0 (20.1,23.8)	15.1 (13.5,16.6)	13.4 (11.9,14.8)	12.6 (11.2,14.0)	11.5 (10.2,12.8)	10.5 (9.2,13.6)	Urban
Total	Gyeonggi-do	Dongducheon-si	16.3 (15.4,17.3)	23.8 (21.1,26.5)	17.3 (15.0,19.6)	15.4 (13.3,17.5)	14.0 (12.0,16.0)	11.5 (9.7,13.4)	12.3 (9.0,15.4)	Urban
Total	Gyeonggi-do	Sangnok-gu	13.8 (12.7,14.8)	19.9 (17.2,22.7)	14.5 (12.1,16.8)	12.5 (10.3,14.7)	11.4 (9.4,13.4)	10.7 (8.7,12.7)	9.2 (8.6,14.6)	Metropolitan
Total	Gyeonggi-do	Danwon-gu	15.3 (14.2,16.4)	22.6 (19.5,25.7)	15.6 (13.1,18.0)	14.0 (11.6,16.3)	12.6 (10.3,14.9)	12.1 (9.8,14.3)	10.5 (7.0,13.4)	Metropolitan
Total	Gyeonggi-do	Deogyang-gu	15.4 (14.4,16.4)	23.7 (20.9,26.4)	16.4 (14.2,18.7)	13.1 (11.0,15.1)	12.0 (10.1,13.9)	11.9 (9.9,13.9)	11.8 (6.3,12.5)	Metropolitan
Total	Gyeonggi-do	Ilsandong-gu	12.5 (11.6,13.5)	16.9 (14.4,19.4)	13.4 (11.2,15.5)	12.9 (10.8,14.9)	11.1 (9.2,13.0)	8.7 (6.9,10.4)	8.2 (5.6,11.8)	Metropolitan
Total	Gyeonggi-do	Ilsanseo-gu	11.7 (10.8,12.6)	17.7 (15.2,20.2)	11.2 (9.3,13.2)	10.1 (8.3,12.0)	9.4 (7.7,11.1)	10.5 (8.6,12.4)	7.2 (2.3,8.1)	Metropolitan
Total	Gyeonggi-do	Gwacheon-si	11.9 (11.0,12.8)	16.7 (14.3,19.1)	11.6 (9.7,13.5)	11.1 (9.2,13.0)	10.9 (9.0,12.8)	9.4 (7.6,11.1)	7.3 (3.9,9.7)	Urban
Total	Gyeonggi-do	Guri-si	15.3 (14.3,16.4)	21.4 (18.7,24.2)	15.6 (13.3,17.9)	14.5 (12.3,16.8)	14.5 (12.3,16.6)	10.6 (8.7,12.6)	10.8 (5.4,11.6)	Urban
Total	Gyeonggi-do	Namyangju-si	14.8 (13.8,15.8)	19.6 (17.0,22.1)	15.6 (13.4,17.9)	13.3 (11.3,15.3)	12.5 (10.5,14.5)	13.3 (11.2,15.4)	6.3 (3.7,10.1)	Urban
Total	Gyeonggi-do	Osan-si	13.9 (12.9,15.0)	17.6 (14.9,20.3)	13.2 (10.9,15.6)	13.3 (11.1,15.5)	14.3 (11.9,16.7)	11.4 (9.2,13.6)	6.2 (1.9,8.3)	Urban
Total	Gyeonggi-do	Siheung-si	14.7 (13.6,15.8)	19.7 (16.9,22.5)	15.8 (13.3,18.2)	12.8 (10.6,14.9)	13.8 (11.5,16.1)	11.5 (9.3,13.6)	8.2 (5.7,13.3)	Urban
Total	Gyeonggi-do	Gunpo-si	14.5 (13.5,15.5)	21.9 (19.1,24.7)	13.5 (11.4,15.6)	12.2 (10.2,14.3)	13.4 (11.2,15.5)	11.9 (9.8,13.9)	10.0 (5.6,11.8)	Urban
Total	Gyeonggi-do	Uiwang-si	12.8 (11.9,13.7)	18.2 (15.7,20.6)	13.8 (11.6,16.0)	10.4 (8.6,12.3)	11.2 (9.4,13.1)	10.7 (8.8,12.6)	7.5 (5.1,11.1)	Urban
Total	Gyeonggi-do	Hanam-si	14.6 (13.7,15.6)	18.9 (16.4,21.4)	15.8 (13.6,18.1)	13.6 (11.5,15.7)	12.9 (11.0,14.9)	12.4 (10.4,14.4)	6.5 (3.7,10.3)	Urban
Total	Gyeonggi-do	Cheoin-gu	13.4 (12.5,14.3)	18.2 (15.8,20.6)	14.4 (12.3,16.5)	12.6 (10.7,14.5)	12.2 (10.2,14.1)	9.8 (8.0,11.5)	8.4 (1.7,8.9)	Metropolitan
Total	Gyeonggi-do	Giheung-gu	11.0 (10.2,11.9)	16.2 (13.8,18.6)	10.9 (9.0,12.8)	10.2 (8.4,12.1)	10.0 (8.1,11.8)	8.1 (6.4,9.7)	8.1 (4.5,10.1)	Metropolitan

Total	Gyeonggi-do	Suji-gu	10.0 (9.1,10.8)	12.0 (9.9,14.0)	10.1 (8.2,11.9)	10.4 (8.5,12.2)	8.4 (6.7,10.0)	9.2 (7.5,11.0)	2.8 (-0.1,5.5)	Metropolitan
Total	Gyeonggi-do	Paju-si	14.7 (13.8,15.7)	21.7 (19.0,24.4)	15.4 (13.3,17.6)	13.4 (11.4,15.4)	11.6 (9.7,13.4)	12.0 (10.1,13.9)	9.7 (5.4,11.6)	Urban
Total	Gyeonggi-do	Icheon-si	15.4 (14.4,16.3)	21.3 (18.7,23.9)	16.1 (13.9,18.3)	13.4 (11.4,15.5)	13.5 (11.4,15.5)	12.5 (10.5,14.5)	8.8 (6.4,12.6)	Urban
Total	Gyeonggi-do	Anseong-si	15.3 (14.3,16.2)	20.9 (18.4,23.3)	15.7 (13.6,17.9)	13.5 (11.5,15.5)	13.8 (11.8,15.8)	12.2 (10.4,14.1)	8.7 (4.0,11.4)	Urban
Total	Gyeonggi-do	Gimpo-si	11.9 (11.1,12.8)	15.7 (13.4,17.9)	12.6 (10.6,14.6)	11.2 (9.4,13.1)	10.8 (9.0,12.6)	9.5 (7.8,11.3)	6.2 (2.9,8.5)	Urban
Total	Gyeonggi-do	Hwaseongsi	13.6 (12.7,14.6)	19.5 (16.9,22.1)	14.8 (12.5,17.1)	12.8 (10.7,14.9)	11.9 (9.9,13.9)	9.4 (7.5,11.2)	10.1 (8.0,13.8)	Urban
Total	Gyeonggi-do	Gwangju-si	13.2 (12.3,14.1)	17.4 (15.0,19.8)	14.3 (12.2,16.4)	12.5 (10.5,14.4)	10.6 (8.7,12.4)	11.1 (9.2,13.0)	6.3 (4.2,11.2)	Urban
Total	Gyeonggi-do	Yangju-si	15.5 (14.5,16.5)	21.6 (19.0,24.3)	15.8 (13.6,18.0)	13.3 (11.3,15.3)	14.7 (12.6,16.7)	12.5 (10.5,14.5)	9.1 (6.1,12.3)	Urban
Total	Gyeonggi-do	Pocheon-si	16.6 (15.6,17.6)	23.8 (21.2,26.5)	17.7 (15.5,19.9)	15.2 (13.2,17.3)	14.1 (12.2,16.0)	12.4 (10.6,14.3)	11.4 (6.9,13.3)	Urban
Total	Gyeonggi-do	Yeoju-gun	16.6 (15.6,17.5)	21.4 (19.0,23.9)	18.4 (16.2,20.6)	15.1 (13.1,17.1)	14.4 (12.5,16.4)	13.8 (11.8,15.7)	7.6 (2.9,9.3)	Urban
Total	Gyeonggi-do	Yeoncheon-gun	15.7 (14.8,16.6)	22.9 (20.4,25.5)	16.5 (14.5,18.5)	15.1 (13.1,17.0)	13.5 (11.6,15.3)	10.6 (9.0,12.2)	12.3 (8.2,14.4)	Rural
Total	Gyeonggi-do	Gapyeong-gun	14.9 (14.0,15.8)	23.2 (20.6,25.8)	16.6 (14.6,18.7)	13.5 (11.7,15.4)	10.8 (9.2,12.5)	10.5 (8.8,12.1)	12.7 (9.4,15.6)	Rural
Total	Gyeonggi-do	Yangpyeong-gun	15.9 (15.0,16.8)	20.4 (18.0,22.8)	18.0 (15.8,20.1)	14.7 (12.7,16.6)	15.0 (13.1,17.0)	11.4 (9.6,13.1)	9.0 (5.8,12.0)	Rural
Total	Gangwon-do	Chuncheon-si	13.9 (13.0,14.8)	22.4 (19.8,25.0)	15.4 (13.3,17.5)	11.7 (9.9,13.5)	11.3 (9.5,13.1)	9.2 (7.6,10.8)	13.2 (9.0,15.8)	Urban
Total	Gangwon-do	Wonju-si	14.6 (13.7,15.6)	21.7 (19.1,24.4)	17.4 (15.1,19.7)	11.5 (9.7,13.4)	12.7 (10.7,14.6)	10.0 (8.2,11.8)	11.7 (7.9,14.1)	Urban
Total	Gangwon-do	Gangneung-si	15.3 (14.4,16.2)	22.6 (20.0,25.2)	16.3 (14.2,18.3)	13.6 (11.7,15.4)	13.2 (11.3,15.1)	11.3 (9.5,13.1)	11.3 (9.3,15.5)	Urban
Total	Gangwon-do	Donghae-si	15.3 (14.3,16.2)	22.6 (20.0,25.2)	16.5 (14.4,18.6)	14.1 (12.1,16.1)	11.8 (10.0,13.6)	11.6 (9.7,13.4)	11.0 (8.6,14.8)	Urban
Total	Gangwon-do	Taebaek-si	20.4 (19.4,21.5)	28.4 (25.5,31.2)	22.4 (20.0,24.9)	19.9 (17.7,22.2)	15.9 (13.9,18.0)	15.6 (13.5,17.6)	12.8 (10.0,17.0)	Urban
Total	Gangwon-do	Sokcho-si	17.3 (16.3,18.3)	26.1 (23.3,28.9)	17.8 (15.6,20.0)	15.2 (13.1,17.2)	13.3 (11.4,15.2)	14.5 (12.4,16.6)	11.6 (8.0,14.6)	Urban
Total	Gangwon-do	Samcheok-si	17.5 (16.5,18.4)	27.7 (24.8,30.6)	18.3 (16.1,20.5)	16.5 (14.5,18.5)	13.5 (11.6,15.4)	11.9 (10.2,13.7)	15.8 (10.8,17.4)	Urban
Total	Gangwon-do	Hongcheon-gun	15.1 (14.2,16.0)	22.4 (19.9,24.9)	16.5 (14.4,18.5)	13.6 (11.8,15.4)	11.3 (9.7,13.0)	12.2 (10.4,14.0)	10.2 (7.9,14.5)	Rural
Total	Gangwon-do	Hoengseong-gun	17.4 (16.5,18.4)	23.7 (21.0,26.3)	18.8 (16.6,21.0)	17.0 (14.9,19.1)	15.1 (13.1,17.1)	12.6 (10.7,14.5)	11.1 (7.6,14.0)	Rural
Total	Gangwon-do	Yeongwol-gun	18.8 (17.8,19.8)	28.1 (25.1,31.0)	20.6 (18.3,22.9)	15.9 (13.9,17.8)	15.6 (13.7,17.6)	14.2 (12.3,16.1)	13.9 (10.3,17.1)	Rural
Total	Gangwon-do	Pyeongchang-gun	16.3 (15.3,17.2)	24.2 (21.6,26.8)	17.4 (15.4,19.5)	13.9 (12.1,15.8)	14.4 (12.5,16.3)	11.6 (9.8,13.3)	12.6 (7.9,14.3)	Rural
Total	Gangwon-do	Jeongseon-gun	19.9 (18.9,20.9)	27.4 (24.6,30.2)	20.7 (18.4,22.9)	20.0 (17.7,22.2)	18.0 (15.9,20.2)	13.7 (11.8,15.6)	13.7 (11.7,18.5)	Rural
Total	Gangwon-do	Cheorwon-gun	14.7 (13.8,15.6)	23.5 (20.9,26.1)	15.3 (13.4,17.3)	13.3 (11.4,15.1)	11.5 (9.7,13.2)	10.1 (8.4,11.7)	13.4 (11.3,17.7)	Rural
Total	Gangwon-do	Hwacheon-gun	15.6 (14.6,16.5)	22.5 (19.8,25.2)	17.0 (14.8,19.2)	13.6 (11.7,15.5)	13.5 (11.5,15.4)	11.6 (9.9,13.4)	10.9 (8.9,15.3)	Rural
Total	Gangwon-do	Yanggu-gun	16.1 (15.2,17.1)	22.9 (20.2,25.5)	16.8 (14.6,19.0)	14.7 (12.8,16.7)	13.2 (11.3,15.1)	13.3 (11.4,15.2)	9.6 (5.9,12.5)	Rural
Total	Gangwon-do	Inje-gun	15.6 (14.7,16.6)	23.1 (20.5,25.7)	15.5 (13.5,17.6)	14.6 (12.7,16.6)	13.1 (11.2,15.0)	11.6 (9.8,13.5)	11.5 (8.6,15.0)	Rural
Total	Gangwon-do	Goseong-gun	15.4 (14.5,16.3)	21.3 (18.7,23.8)	17.8 (15.5,20.1)	14.8 (12.8,16.8)	12.7 (10.9,14.5)	10.6 (8.9,12.3)	10.7 (7.9,14.1)	Rural
Total	Gangwon-do	Yangyang-gun	15.1 (14.2,16.0)	22.6 (20.0,25.2)	17.2 (15.1,19.3)	13.3 (11.4,15.2)	12.9 (11.1,14.7)	9.5 (8.0,11.0)	13.1 (9.7,15.9)	Rural
Total	Chungcheongbuk-do	Cheongju-si	13.8 (13.2,14.3)	20.7 (19.1,22.2)	15.4 (14.1,16.8)	12.1 (11.0,13.2)	11.2 (10.2,12.3)	10.2 (9.2,11.2)	10.5 (8.2,11.8)	Metropolitan
Total	Chungcheongbuk-do	Chungju-si	17.5 (16.5,18.5)	25.0 (22.3,27.7)	20.3 (18.0,22.6)	15.4 (13.4,17.5)	13.8 (11.9,15.7)	13.2 (11.3,15.2)	11.8 (9.1,15.5)	Urban
Total	Chungcheongbuk-do	Jecheon-si	15.1 (14.2,16.0)	21.9 (19.4,24.4)	15.2 (13.2,17.1)	14.6 (12.7,16.6)	12.6 (10.8,14.4)	11.5 (9.7,13.3)	10.4 (7.7,14.1)	Urban
Total	Chungcheongbuk-do	Boeun-gun	17.9 (17.0,18.9)	26.8 (23.9,29.8)	20.6 (18.3,23.0)	15.2 (13.4,17.1)	14.5 (12.5,16.4)	13.6 (11.7,15.5)	13.2 (10.6,17.4)	Rural
Total	Chungcheongbuk-do	Okcheon-gun	16.3 (15.4,17.2)	21.2 (18.8,23.7)	18.5 (16.4,20.7)	15.3 (13.4,17.3)	14.3 (12.4,16.1)	12.2 (10.3,14.0)	9.0 (5.4,11.8)	Rural
Total	Chungcheongbuk-do	Yeongdong-gun	16.3 (15.4,17.2)	24.9 (22.2,27.5)	16.9 (14.8,18.9)	14.3 (12.5,16.1)	13.1 (11.4,14.9)	12.5 (10.7,14.3)	12.4 (8.4,14.8)	Rural

Total	Chungcheongbuk-do	Jincheon-gun	16.8 (15.9,17.8)	23.8 (21.1,26.5)	15.1 (13.0,17.2)	16.8 (14.6,18.9)	13.5 (11.6,15.4)	15.2 (13.1,17.2)	8.6 (4.6,11.4)	Rural
Total	Chungcheongbuk-do	Goesan-gun	15.6 (14.7,16.5)	21.2 (18.7,23.6)	15.4 (13.4,17.5)	14.9 (12.9,16.9)	14.1 (12.1,16.0)	12.6 (10.8,14.5)	8.6 (6.7,12.9)	Rural
Total	Chungcheongbuk-do	Eumseong-gun	17.5 (16.5,18.4)	25.2 (22.4,28.0)	19.5 (17.1,21.8)	16.8 (14.8,18.8)	13.5 (11.7,15.2)	12.6 (10.8,14.4)	12.6 (9.8,16.4)	Rural
Total	Chungcheongbuk-do	Danyang-gun	17.7 (16.7,18.6)	26.9 (24.1,29.7)	16.6 (14.4,18.7)	15.7 (13.7,17.7)	14.3 (12.5,16.2)	15.4 (13.3,17.5)	11.5 (6.2,13.0)	Rural
Total	Chungcheongbuk-do	Jeungpyeong-gun	14.9 (14.1,15.8)	22.4 (19.9,24.9)	14.8 (13.0,16.6)	14.1 (12.3,15.8)	13.1 (11.3,14.8)	10.4 (8.8,12.0)	12.0 (9.3,15.5)	Rural
Total	Chungcheongnam-do	Cheonan-si	16.0 (14.9,17.0)	23.0 (20.0,26.0)	17.9 (15.4,20.4)	14.5 (12.3,16.8)	13.5 (11.3,15.7)	11.6 (9.6,13.7)	11.4 (8.3,14.7)	Metropolitan
Total	Chungcheongnam-do	Gongju-si	17.2 (16.2,18.1)	25.3 (22.6,28.0)	18.0 (15.8,20.1)	16.6 (14.5,18.6)	14.3 (12.4,16.1)	11.9 (10.1,13.7)	13.4 (10.3,16.7)	Urban
Total	Chungcheongnam-do	Boryeong-si	19.8 (18.8,20.8)	30.1 (27.1,33.1)	22.5 (20.0,24.9)	15.7 (13.8,17.6)	16.9 (14.8,19.0)	14.5 (12.5,16.4)	15.6 (11.9,18.9)	Urban
Total	Chungcheongnam-do	Asan-si	15.9 (14.9,16.9)	24.3 (21.4,27.2)	16.7 (14.4,18.9)	14.2 (12.1,16.3)	12.8 (10.8,14.8)	11.7 (9.8,13.6)	12.6 (9.3,15.7)	Urban
Total	Chungcheongnam-do	Seosan-si	16.0 (15.1,17.0)	21.8 (19.3,24.3)	17.2 (15.1,19.4)	14.5 (12.5,16.5)	14.3 (12.3,16.3)	12.5 (10.6,14.3)	9.3 (6.2,12.4)	Urban
Total	Chungcheongnam-do	Nonsan-si	17.1 (16.1,18.0)	26.1 (23.3,28.8)	17.6 (15.6,19.7)	15.2 (13.3,17.2)	14.1 (12.2,16.0)	12.2 (10.4,14.0)	13.9 (10.9,17.7)	Urban
Total	Chungcheongnam-do	Gyeryong-si	13.5 (12.5,14.5)	18.7 (16.0,21.4)	15.7 (13.3,18.1)	11.3 (9.2,13.4)	12.0 (9.9,14.1)	10.0 (8.0,11.9)	8.7 (4.7,10.7)	Urban
Total	Chungcheongnam-do	Dangjin-si	16.4 (15.4,17.3)	23.6 (21.0,26.2)	16.4 (14.3,18.5)	16.2 (14.1,18.4)	13.8 (11.9,15.7)	12.3 (10.4,14.1)	11.3 (6.3,12.7)	Rural
Total	Chungcheongnam-do	Geumsan-gun	16.8 (15.9,17.7)	22.9 (20.4,25.5)	19.7 (17.5,21.9)	15.2 (13.4,17.1)	13.9 (12.1,15.7)	12.1 (10.4,13.7)	10.8 (6.9,13.3)	Rural
Total	Chungcheongnam-do	Buyeo-gun	15.8 (14.9,16.7)	22.9 (20.3,25.6)	17.7 (15.6,19.8)	13.6 (11.9,15.3)	14.0 (12.2,15.9)	11.2 (9.6,12.9)	11.7 (7.6,13.8)	Rural
Total	Chungcheongnam-do	Seocheon-gun	17.0 (16.1,18.0)	25.2 (22.4,28.0)	17.0 (14.9,19.1)	15.2 (13.3,17.1)	15.1 (13.1,17.0)	13.1 (11.2,15.0)	12.1 (7.4,14.0)	Rural
Total	Chungcheongnam-do	Cheongyang-gun	15.2 (14.4,16.1)	22.8 (20.1,25.4)	16.6 (14.6,18.6)	15.3 (13.4,17.2)	11.9 (10.1,13.6)	9.9 (8.4,11.3)	12.9 (10.8,17.4)	Rural
Total	Chungcheongnam-do	Hongseong-gun	16.8 (15.9,17.7)	22.8 (20.3,25.4)	18.1 (15.9,20.2)	15.6 (13.6,17.5)	14.5 (12.6,16.5)	13.1 (11.3,14.9)	9.7 (8.0,14.4)	Rural
Total	Chungcheongnam-do	Yesan-gun	15.6 (14.8,16.5)	23.9 (21.3,26.6)	16.9 (14.8,18.9)	13.3 (11.5,15.0)	12.5 (10.8,14.2)	12.3 (10.5,14.1)	11.6 (9.0,15.6)	Rural
Total	Chungcheongnam-do	Taean-gun	15.9 (15.0,16.8)	20.8 (18.4,23.2)	17.9 (15.8,20.1)	15.6 (13.7,17.6)	13.4 (11.6,15.3)	11.7 (10.0,13.5)	9.1 (5.4,11.6)	Urban
Total	Jeollabuk-do	Jeonju-si	14.0 (13.1,15.0)	21.6 (19.0,24.3)	14.9 (12.8,17.1)	12.5 (10.5,14.4)	11.5 (9.7,13.4)	10.3 (8.5,12.1)	11.3 (9.9,16.7)	Metropolitan
Total	Jeollabuk-do	Gunsan-si	17.7 (16.7,18.7)	26.2 (23.4,29.0)	17.5 (15.3,19.7)	15.6 (13.5,17.7)	15.9 (13.8,18.1)	13.7 (11.8,15.7)	12.5 (10.6,18.6)	Urban
Total	Jeollabuk-do	Iksan-si	18.5 (17.4,19.5)	29.0 (26.1,31.9)	18.4 (16.2,20.7)	17.1 (14.9,19.2)	14.5 (12.6,16.5)	13.4 (11.5,15.3)	15.6 (12.9,19.7)	Urban
Total	Jeollabuk-do	Jeongeup-si	19.0 (18.0,20.0)	29.6 (26.6,32.5)	19.0 (16.8,21.2)	17.9 (15.8,20.0)	13.9 (12.0,15.7)	15.0 (13.0,17.0)	14.6 (10.5,17.3)	Urban
Total	Jeollabuk-do	Namwon-si	18.4 (17.5,19.4)	25.8 (23.1,28.5)	20.9 (18.6,23.2)	17.1 (15.0,19.2)	13.5 (11.8,15.3)	15.1 (13.2,17.0)	10.7 (10.5,18.1)	Urban
Total	Jeollabuk-do	Gimje-si	17.7 (16.7,18.7)	26.4 (23.6,29.2)	17.9 (15.8,20.0)	15.7 (13.7,17.6)	15.7 (13.7,17.7)	13.2 (11.3,15.1)	13.2 (6.3,14.7)	Urban
Total	Jeollabuk-do	Wanju-gun	16.7 (15.8,17.6)	24.3 (21.7,27.0)	19.4 (17.2,21.6)	15.4 (13.5,17.3)	12.2 (10.4,13.9)	12.5 (10.8,14.2)	11.8 (8.7,17.3)	Rural
Total	Jeollabuk-do	Jinan-gun	16.2 (15.2,17.1)	23.0 (20.3,25.8)	19.6 (17.3,21.9)	13.7 (12.0,15.4)	14.3 (12.4,16.2)	10.4 (8.8,12.0)	12.6 (11.8,21.6)	Rural
Total	Jeollabuk-do	Muju-gun	18.3 (17.3,19.3)	25.6 (22.8,28.3)	20.7 (18.3,23.0)	16.7 (14.7,18.7)	14.9 (13.0,16.9)	13.4 (11.5,15.3)	12.2 (7.9,14.7)	Rural
Total	Jeollabuk-do	Jangsu-gun	18.0 (17.0,19.0)	24.6 (21.8,27.4)	19.7 (17.4,21.9)	16.2 (14.3,18.2)	13.9 (12.1,15.8)	16.0 (13.9,18.0)	8.6 (5.7,13.5)	Rural
Total	Jeollabuk-do	Imsil-gun	17.7 (16.7,18.7)	24.2 (21.3,27.1)	17.2 (15.2,19.2)	22.2 (19.7,24.6)	13.8 (11.9,15.7)	11.0 (9.5,12.6)	13.2 (11.1,18.3)	Rural
Total	Jeollabuk-do	Sunchang-gun	17.7 (16.7,18.7)	25.6 (22.8,28.3)	21.3 (18.8,23.8)	15.6 (13.8,17.5)	13.9 (12.0,15.9)	12.0 (10.3,13.8)	13.6 (9.7,16.3)	Rural
Total	Jeollabuk-do	Gochang-gun	21.8 (20.7,22.9)	31.2 (28.0,34.3)	21.9 (19.6,24.2)	19.5 (17.2,21.7)	20.8 (18.4,23.2)	16.2 (14.2,18.3)	15.0 (11.3,18.3)	Rural
Total	Jeollabuk-do	Buan-gun	19.5 (18.5,20.5)	30.8 (27.7,33.9)	20.2 (17.9,22.5)	18.3 (16.1,20.5)	12.9 (11.2,14.7)	15.4 (13.3,17.4)	15.4 (11.7,19.1)	Rural
Total	Jeollanam-do	Mokpo-si	17.2 (16.2,18.2)	25.6 (22.8,28.4)	19.5 (17.1,21.9)	16.6 (14.4,18.7)	13.7 (11.7,15.6)	10.9 (9.1,12.7)	14.7 (8.9,15.3)	Urban
Total	Jeollanam-do	Yeosu-si	16.0 (15.1,16.9)	22.7 (20.1,25.2)	15.7 (13.7,17.7)	14.7 (12.7,16.7)	12.5 (10.6,14.3)	14.6 (12.6,16.6)	8.1 (4.8,11.2)	Urban
Total	Jeollanam-do	Suncheon-si	14.8 (13.9,15.8)	21.7 (19.1,24.3)	17.0 (14.9,19.2)	12.4 (10.5,14.3)	12.7 (10.8,14.6)	10.7 (9.0,12.4)	11.0 (10.1,16.3)	Urban

Volume: 39, Article ID: e2017011,

https://doi.org/10.4178/epih.e2017011

Total	Jeollanam-do	Naju-si	17.6 (16.6,18.5)	28.2 (25.4,31.1)	18.4 (16.3,20.6)	15.4 (13.5,17.4)	13.6 (11.8,15.3)	12.1 (10.4,13.7)	16.1 (13.6,20.2)	Urban
Total	Jeollanam-do	Gwangyang-si	14.3 (13.3,15.3)	21.3 (18.6,24.0)	15.6 (13.4,17.8)	13.0 (11.0,15.1)	11.2 (9.3,13.1)	10.7 (8.8,12.5)	10.6 (6.8,13.0)	Urban
Total	Jeollanam-do	Damyang-gun	17.4 (16.4,18.3)	25.6 (22.9,28.4)	18.8 (16.6,21.0)	15.2 (13.4,17.0)	13.9 (12.1,15.7)	13.6 (11.7,15.5)	12.0 (10.2,16.6)	Rural
Total	Jeollanam-do	Gokseong-gun	16.8 (15.9,17.7)	22.4 (19.8,25.0)	17.3 (15.3,19.3)	17.1 (15.0,19.2)	13.3 (11.6,15.0)	14.0 (12.1,15.9)	8.4 (5.9,12.5)	Rural
Total	Jeollanam-do	Gurye-gun	13.4 (12.6,14.2)	22.1 (19.6,24.7)	15.7 (13.7,17.7)	10.4 (9.0,11.9)	10.1 (8.6,11.6)	9.1 (7.6,10.7)	13.0 (10.3,16.5)	Rural
Total	Jeollanam-do	Goheung-gun	15.6 (14.7,16.5)	28.3 (25.1,31.6)	15.4 (13.5,17.3)	14.8 (12.9,16.6)	11.8 (10.2,13.5)	8.2 (7.0,9.5)	20.1 (18.0,24.0)	Rural
Total	Jeollanam-do	Boseong-gun	18.2 (17.2,19.1)	27.8 (24.7,30.8)	21.0 (18.6,23.3)	17.3 (15.2,19.3)	13.7 (11.9,15.5)	11.2 (9.8,12.7)	16.6 (14.1,20.9)	Rural
Total	Jeollanam-do	Hwasun-gun	18.9 (17.9,19.9)	29.2 (26.3,32.1)	18.4 (16.3,20.5)	18.1 (16.0,20.3)	14.3 (12.5,16.2)	14.7 (12.8,16.7)	14.5 (11.0,18.0)	Rural
Total	Jeollanam-do	Jangheung-gun	15.9 (15.0,16.8)	26.4 (23.5,29.3)	16.0 (14.1,18.0)	13.7 (11.8,15.5)	11.3 (9.8,12.8)	12.7 (10.9,14.6)	13.7 (9.8,18.0)	Rural
Total	Jeollanam-do	Gangjin-gun	14.2 (13.3,15.1)	21.7 (19.1,24.3)	15.1 (13.1,17.0)	12.6 (10.8,14.4)	12.2 (10.4,13.9)	9.8 (8.2,11.3)	11.9 (10.8,18.2)	Rural
Total	Jeollanam-do	Haenam-gun	17.9 (16.9,18.8)	25.8 (23.0,28.7)	17.8 (15.7,19.8)	16.4 (14.5,18.3)	15.4 (13.4,17.4)	14.6 (12.7,16.5)	11.2 (8.0,15.4)	Rural
Total	Jeollanam-do	Yeongam-gun	17.5 (16.6,18.5)	25.9 (23.2,28.6)	19.2 (17.0,21.4)	16.1 (14.1,18.1)	15.0 (13.0,16.9)	11.7 (10.1,13.4)	14.2 (10.8,17.2)	Rural
Total	Jeollanam-do	Muan-gun	15.2 (14.3,16.1)	20.3 (17.9,22.7)	15.7 (13.7,17.6)	16.0 (14.0,18.0)	11.8 (10.2,13.5)	12.5 (10.6,14.3)	7.8 (6.0,12.2)	Rural
Total	Jeollanam-do	Hampyeong-gun	19.2 (18.2,20.2)	28.6 (25.6,31.6)	19.7 (17.5,22.0)	17.1 (15.0,19.3)	16.3 (14.3,18.4)	14.3 (12.3,16.2)	14.3 (11.6,18.4)	Rural
Total	Jeollanam-do	Yeonggwang-gun	19.3 (18.3,20.3)	29.0 (26.1,31.8)	21.7 (19.4,24.0)	17.7 (15.6,19.8)	15.1 (13.3,16.9)	13.4 (11.6,15.3)	15.6 (13.8,20.6)	Rural
Total	Jeollanam-do	Jangseong-gun	17.2 (16.3,18.2)	26.0 (23.2,28.8)	18.2 (16.1,20.4)	15.0 (13.1,16.9)	15.0 (13.1,16.9)	12.3 (10.6,14.1)	13.7 (6.3,12.9)	Rural
Total	Jeollanam-do	Wando-gun	17.4 (16.5,18.4)	27.4 (24.5,30.2)	19.3 (17.2,21.4)	14.3 (12.5,16.2)	14.8 (13.0,16.6)	11.4 (9.7,13.0)	16.0 (13.8,20.2)	Rural
Total	Jeollanam-do	Jindo-gun	18.5 (17.6,19.5)	28.4 (25.4,31.4)	20.1 (17.8,22.3)	16.7 (14.8,18.7)	14.6 (12.8,16.4)	13.0 (11.2,14.9)	15.4 (12.7,19.3)	Rural
Total	Jeollanam-do	Sinan-gun	19.8 (18.7,20.8)	25.9 (23.2,28.6)	23.3 (20.8,25.9)	17.2 (15.1,19.3)	17.5 (15.4,19.5)	14.7 (12.7,16.6)	11.2 (11.8,19.4)	Rural
Total	Gyeongsangbuk-do	Nam-gu	17.1 (16.1,18.1)	26.5 (23.7,29.3)	17.7 (15.4,19.9)	15.9 (13.7,18.1)	12.6 (10.7,14.6)	12.8 (10.8,14.7)	13.7 (10.1,16.7)	Metropolitan
Total	Gyeongsangbuk-do	Buk-gu	15.5 (14.6,16.5)	23.3 (20.6,26.0)	15.7 (13.6,17.8)	14.2 (12.2,16.2)	12.7 (10.8,14.7)	11.8 (9.9,13.7)	11.5 (8.1,14.5)	Metropolitan
Total	Gyeongsangbuk-do	Gyeongju-si	16.9 (16.0,17.9)	24.4 (21.8,27.0)	17.8 (15.6,20.0)	16.3 (14.2,18.3)	13.9 (12.1,15.8)	12.7 (10.9,14.5)	11.7 (8.5,14.9)	Urban
Total	Gyeongsangbuk-do	Gimcheon-si	16.2 (15.3,17.2)	22.4 (19.9,24.8)	17.3 (15.3,19.4)	16.1 (14.0,18.1)	12.7 (10.9,14.6)	12.5 (10.6,14.3)	9.9 (6.2,12.6)	Urban
Total	Gyeongsangbuk-do	Andong-si	16.9 (15.9,17.8)	25.6 (22.9,28.3)	18.7 (16.5,20.9)	15.8 (13.8,17.7)	12.6 (10.8,14.4)	12.2 (10.5,14.0)	13.4 (8.9,15.3)	Urban
Total	Gyeongsangbuk-do	Gumi-si	17.1 (16.2,17.9)	25.7 (23.3,28.0)	18.2 (16.2,20.1)	15.2 (13.4,17.0)	14.3 (12.6,16.1)	13.3 (11.8,14.9)	12.4 (8.5,13.1)	Urban
Total	Gyeongsangbuk-do	Yeongju-si	17.5 (16.6,18.5)	26.5 (23.7,29.3)	18.0 (15.9,20.1)	16.9 (14.9,19.0)	14.2 (12.3,16.0)	12.7 (10.9,14.4)	13.8 (9.9,16.5)	Urban
Total	Gyeongsangbuk-do	Yeongcheon-si	19.3 (18.3,20.3)	25.4 (22.7,28.1)	22.3 (19.9,24.6)	17.6 (15.5,19.7)	16.3 (14.3,18.4)	15.0 (13.0,17.0)	10.4 (6.7,13.5)	Urban
Total	Gyeongsangbuk-do	Sangju-si	17.1 (16.2,18.0)	24.9 (22.3,27.6)	16.7 (14.7,18.6)	15.9 (13.9,17.8)	14.7 (12.8,16.5)	13.3 (11.5,15.2)	11.6 (7.5,13.9)	Urban
Total	Gyeongsangbuk-do	Mungyeong-si	17.5 (16.6,18.5)	23.7 (21.2,26.3)	17.3 (15.3,19.4)	17.9 (15.8,19.9)	15.5 (13.6,17.5)	13.4 (11.5,15.2)	10.3 (5.3,12.7)	Urban
Total	Gyeongsangbuk-do	Gyeongsan-si	17.1 (16.1,18.1)	24.2 (21.5,27.0)	18.2 (15.9,20.4)	14.1 (12.1,16.1)	15.8 (13.7,18.0)	13.5 (11.5,15.4)	10.7 (6.4,12.8)	Urban
Total	Gyeongsangbuk-do	Gunwi-gun	17.1 (16.1,18.0)	24.4 (21.6,27.3)	18.1 (16.1,20.2)	15.6 (13.7,17.5)	14.8 (12.8,16.7)	12.9 (11.1,14.7)	11.5 (5.9,12.7)	Rural
Total	Gyeongsangbuk-do	Uiseong-gun	16.4 (15.4,17.3)	22.2 (19.6,24.9)	17.1 (15.0,19.1)	14.2 (12.4,16.1)	15.3 (13.2,17.3)	13.3 (11.4,15.3)	8.9 (4.4,10.8)	Rural
Total	Gyeongsangbuk-do	Cheongsong-gun	18.9 (17.9,19.9)	28.0 (24.9,31.0)	19.1 (17.0,21.2)	16.5 (14.5,18.5)	15.5 (13.6,17.5)	15.6 (13.5,17.7)	12.4 (9.6,16.4)	Rural
Total	Gyeongsangbuk-do	Yeongyang-gun	20.6 (19.5,21.6)	29.5 (26.4,32.5)	22.9 (20.4,25.4)	19.1 (16.8,21.3)	17.1 (15.0,19.2)	14.6 (12.7,16.6)	14.9 (11.2,18.2)	Rural
Total	Gyeongsangbuk-do	Yeongdeok-gun	17.6 (16.6,18.6)	27.9 (24.8,31.0)	20.8 (18.4,23.3)	14.8 (13.0,16.6)	13.0 (11.2,14.8)	11.4 (9.7,13.1)	16.5 (11.9,18.3)	Rural
Total	Gyeongsangbuk-do	Cheongdo-gun	18.2 (17.3,19.2)	25.3 (22.5,28.0)	17.6 (15.5,19.7)	16.0 (14.1,17.9)	16.9 (14.8,19.0)	15.7 (13.7,17.8)	9.6 (4.4,11.2)	Rural
Total	Gyeongsangbuk-do	Goryeong-gun	17.9 (16.9,18.8)	23.8 (21.1,26.4)	17.4 (15.3,19.4)	17.2 (15.1,19.4)	15.3 (13.3,17.2)	15.7 (13.6,17.8)	8.1 (4.1,10.9)	Rural

Total	Gyeongsangbuk-do	Seongju-gun	12.1 (11.3,12.9)	19.8 (17.4,22.2)	13.0 (11.2,14.8)	9.9 (8.3,11.4)	9.0 (7.5,10.4)	8.9 (7.4,10.4)	10.9 (7.5,13.3)	Rural
Total	Gyeongsangbuk-do	Chilgok-gun	17.1 (16.1,18.1)	24.3 (21.5,27.2)	17.4 (15.0,19.8)	17.2 (14.9,19.5)	13.1 (11.1,15.1)	13.9 (11.9,16.0)	10.4 (6.5,13.1)	Rural
Total	Gyeongsangbuk-do	Yecheon-gun	18.4 (17.3,19.4)	30.1 (26.9,33.3)	18.6 (16.4,20.7)	17.7 (15.4,19.9)	13.8 (12.0,15.6)	11.8 (10.1,13.5)	18.3 (11.4,18.2)	Rural
Total	Gyeongsangbuk-do	Bonghwa-gun	19.7 (18.7,20.8)	28.0 (25.0,31.0)	19.8 (17.5,22.1)	19.6 (17.3,21.9)	17.0 (15.0,19.1)	14.4 (12.4,16.4)	13.6 (9.4,16.4)	Rural
Total	Gyeongsangbuk-do	Uljin-gun	18.3 (17.3,19.3)	27.2 (24.4,30.0)	19.6 (17.5,21.8)	16.4 (14.4,18.3)	15.5 (13.5,17.5)	13.3 (11.5,15.0)	13.9 (10.3,17.1)	Rural
Total	Gyeongsangbuk-do	Ulleung-gun	15.7 (14.8,16.7)	22.6 (19.9,25.2)	17.7 (15.5,19.9)	14.3 (12.3,16.4)	12.3 (10.4,14.1)	11.7 (9.8,13.6)	10.9 (8.8,15.6)	Rural
Total	Gyeongsangnam-do	Changwon-si	17.5 (16.3,18.7)	23.3 (20.1,26.4)	18.6 (15.9,21.4)	16.9 (14.4,19.4)	15.9 (13.4,18.4)	13.2 (10.9,15.5)	10.1 (6.7,13.3)	Metropolitan
Total	Gyeongsangnam-do	Masan-si	14.6 (13.7,15.5)	22.1 (19.6,24.7)	14.0 (12.0,15.9)	13.4 (11.4,15.3)	13.7 (11.7,15.7)	10.1 (8.4,11.8)	12.0 (7.4,13.6)	Metropolitan
Total	Gyeongsangnam-do	Jinju-si	15.7 (14.7,16.6)	24.1 (21.4,26.7)	15.1 (13.0,17.1)	15.1 (13.0,17.1)	13.0 (11.1,14.9)	11.4 (9.6,13.2)	12.7 (8.8,15.2)	Urban
Total	Gyeongsangnam-do	Jinhae-si	15.8 (14.8,16.8)	22.0 (19.3,24.8)	16.8 (14.5,19.0)	14.9 (12.7,17.1)	13.4 (11.4,15.5)	12.1 (10.2,14.1)	9.9 (6.4,12.8)	Metropolitan
Total	Gyeongsangnam-do	Tongyeong-si	19.5 (18.5,20.6)	28.1 (25.2,31.0)	20.8 (18.4,23.2)	17.7 (15.5,20.0)	15.3 (13.3,17.4)	16.0 (13.9,18.2)	12.1 (8.5,15.3)	Urban
Total	Gyeongsangnam-do	Sacheon-si	16.8 (15.9,17.8)	25.7 (22.9,28.4)	18.6 (16.4,20.9)	13.7 (11.8,15.5)	13.6 (11.8,15.5)	12.9 (11.1,14.8)	12.8 (8.5,15.1)	Urban
Total	Gyeongsangnam-do	Gimhae-si	16.6 (15.5,17.7)	23.1 (20.1,26.1)	16.6 (14.3,19.0)	15.2 (12.9,17.5)	13.8 (11.6,16.0)	15.0 (12.7,17.2)	8.1 (4.3,10.9)	Urban
Total	Gyeongsangnam-do	Miryang-si	17.4 (16.5,18.4)	26.6 (23.9,29.2)	19.0 (16.8,21.2)	16.8 (14.8,18.8)	12.9 (11.2,14.6)	12.1 (10.4,13.8)	14.5 (12.7,19.1)	Urban
Total	Gyeongsangnam-do	Geoje-si	16.4 (15.3,17.5)	21.0 (18.3,23.8)	17.3 (14.8,19.8)	15.8 (13.4,18.2)	13.9 (11.7,16.1)	14.1 (11.9,16.4)	6.9 (3.3,9.7)	Urban
Total	Gyeongsangnam-do	Yangsan-si	13.6 (12.7,14.6)	20.7 (18.1,23.2)	14.1 (11.9,16.3)	11.9 (10.0,13.8)	11.0 (9.1,12.8)	10.9 (9.0,12.7)	9.8 (5.1,11.3)	Urban
Total	Gyeongsangnam-do	Uiryeong-gun	17.5 (16.5,18.5)	23.1 (20.4,25.8)	19.4 (17.1,21.7)	15.0 (13.1,16.8)	15.9 (13.8,18.0)	14.6 (12.5,16.6)	8.5 (7.6,14.8)	Rural
Total	Gyeongsangnam-do	Haman-gun	15.8 (14.9,16.7)	22.2 (19.7,24.6)	15.9 (13.9,17.9)	15.0 (13.2,16.9)	12.9 (11.1,14.6)	13.1 (11.3,14.9)	9.1 (6.3,12.9)	Rural
Total	Gyeongsangnam-do	Changnyeong-gun	18.4 (17.5,19.4)	24.8 (22.1,27.5)	19.7 (17.5,21.9)	16.7 (14.7,18.7)	15.4 (13.5,17.3)	15.8 (13.7,17.8)	9.0 (5.2,12.0)	Rural
Total	Gyeongsangnam-do	Goseong-gun	15.9 (15.0,16.8)	22.8 (20.2,25.4)	17.0 (15.0,19.1)	15.1 (13.2,17.0)	13.7 (11.8,15.5)	11.1 (9.5,12.8)	11.7 (8.0,14.2)	Rural
Total	Gyeongsangnam-do	Namhae-gun	21.6 (20.5,22.7)	31.0 (27.8,34.2)	24.2 (21.6,26.8)	19.9 (17.6,22.2)	18.1 (15.9,20.3)	15.3 (13.2,17.4)	15.7 (11.7,18.9)	Rural
Total	Gyeongsangnam-do	Hadong-gun	21.2 (20.1,22.3)	28.8 (25.8,31.8)	22.7 (20.3,25.2)	20.4 (18.1,22.7)	17.3 (15.2,19.3)	17.2 (15.0,19.4)	11.6 (5.5,12.7)	Rural
Total	Gyeongsangnam-do	Sancheong-gun	19.4 (18.3,20.4)	27.9 (24.9,30.9)	21.2 (18.9,23.6)	17.1 (15.1,19.0)	16.7 (14.6,18.7)	14.2 (12.3,16.2)	13.7 (10.2,17.0)	Rural
Total	Gyeongsangnam-do	Hamyang-gun	19.2 (18.2,20.2)	23.5 (20.9,26.2)	22.3 (19.8,24.8)	18.0 (15.8,20.1)	17.1 (15.0,19.2)	15.3 (13.3,17.3)	8.2 (5.5,13.7)	Rural
Total	Gyeongsangnam-do	Geochang-gun	17.0 (16.0,17.9)	25.5 (22.8,28.1)	17.3 (15.3,19.4)	16.3 (14.3,18.4)	13.6 (11.8,15.4)	12.3 (10.5,14.0)	13.2 (8.4,15.0)	Rural
Total	Gyeongsangnam-do	Hapcheon-gun	19.2 (18.1,20.2)	26.5 (23.6,29.5)	21.8 (19.3,24.3)	18.6 (16.5,20.7)	14.8 (13.0,16.7)	14.7 (12.7,16.7)	11.8 (5.9,13.3)	Rural
Total	Jeju-do	Jeju-si	15.7 (15.1,16.2)	24.4 (22.7,26.0)	17.6 (16.2,19.1)	13.7 (12.5,15.0)	11.7 (10.6,12.8)	11.6 (10.6,12.7)	12.8 (10.9,14.7)	Urban
Total	Jeju-do	Seogwipo-si	16.0 (15.5,16.6)	23.2 (21.6,24.8)	16.7 (15.4,18.0)	14.5 (13.3,15.7)	13.2 (12.1,14.3)	13.0 (11.9,14.2)	10.2 (7.0,11.0)	Urban
Men	Seoul	Jongno-gu	10.0 (8.9,11.2)	18.1 (14.6,21.5)	10.7 (8.1,13.4)	9.6 (7.0,12.1)	6.4 (4.4,8.5)	5.7 (3.8,7.6)	12.4 (8.5,16.5)	Metropolitan
Men	Seoul	Jung-gu	11.1 (9.9,12.3)	20.1 (16.3,23.8)	11.2 (8.5,13.9)	9.7 (7.2,12.2)	8.6 (6.3,10.9)	6.3 (4.3,8.3)	13.8 (8.8,17.4)	Metropolitan
Men	Seoul	Yongsan-gu	9.5 (8.3,10.7)	16.8 (13.3,20.2)	6.8 (4.6,8.9)	9.1 (6.6,11.6)	7.9 (5.5,10.3)	6.7 (4.6,8.9)	10.1 (8.1,16.7)	Metropolitan
Men	Seoul	Seongdong-gu	10.7 (9.4,12.0)	12.7 (9.6,15.8)	10.6 (7.9,13.2)	11.6 (8.5,14.7)	10.3 (7.7,13.0)	8.2 (5.7,10.6)	4.5 (-0.1,7.3)	Metropolitan
Men	Seoul	Gwangjin-gu	9.9 (8.6,11.1)	13.0 (9.7,16.2)	12.1 (9.1,15.1)	9.3 (6.5,12.0)	8.8 (6.2,11.4)	6.3 (4.0,8.5)	6.7 (5.9,12.9)	Metropolitan
Men	Seoul	Dongdaemun-gu	11.0 (9.8,12.3)	17.4 (13.8,20.9)	11.9 (9.1,14.7)	9.0 (6.5,11.5)	8.1 (5.7,10.6)	8.7 (6.1,11.3)	8.7 (3.7,11.9)	Metropolitan
Men	Seoul	Jungnang-gu	11.1 (9.9,12.4)	18.8 (15.0,22.5)	13.2 (10.0,16.3)	8.6 (6.1,11.1)	9.9 (7.2,12.7)	5.5 (3.3,7.6)	13.3 (10.3,18.7)	Metropolitan
Men	Seoul	Seongbuk-gu	10.4 (9.2,11.6)	19.0 (15.3,22.7)	9.8 (7.2,12.4)	7.3 (5.1,9.5)	9.0 (6.4,11.6)	7.5 (5.2,9.8)	11.5 (8.1,16.7)	Metropolitan
Men	Seoul	Gangbuk-gu	12.0 (10.8,13.3)	17.7 (14.2,21.3)	13.8 (10.7,16.8)	12.4 (9.5,15.3)	8.4 (6.0,10.7)	7.7 (5.4,10.0)	10.0 (6.2,15.0)	Metropolitan

Volume: 39, Article ID: e2017011,

https://doi.org/10.4178/epih.e2017011

Men	Seoul	Dobong-gu	11.5 (10.2,12.8)	18.4 (14.8,22.1)	11.2 (8.4,14.1)	11.7 (8.8,14.5)	9.0 (6.5,11.4)	7.7 (5.3,10.0)	10.7 (6.7,15.7)	Metropolitan
Men	Seoul	Nowon-gu	11.3 (9.9,12.6)	21.2 (17.1,25.4)	11.5 (8.6,14.3)	10.6 (7.6,13.6)	7.9 (5.4,10.5)	5.4 (3.4,7.5)	15.8 (9.6,18.0)	Metropolitan
Men	Seoul	Eunpyeong-gu	12.0 (10.7,13.3)	18.8 (15.1,22.6)	13.4 (10.3,16.4)	11.4 (8.6,14.1)	10.3 (7.6,13.0)	6.0 (3.9,8.1)	12.8 (7.8,16.2)	Metropolitan
Men	Seoul	Seodaemun-gu	11.2 (9.9,12.4)	16.6 (13.2,20.1)	12.9 (9.9,15.9)	10.6 (7.8,13.4)	9.6 (7.1,12.1)	6.1 (4.0,8.2)	10.5 (4.9,13.1)	Metropolitan
Men	Seoul	Mapo-gu	9.3 (8.1,10.5)	13.7 (10.4,17.0)	11.8 (8.8,14.9)	10.2 (7.5,13.0)	6.3 (4.1,8.4)	4.9 (3.0,6.9)	8.8 (2.6,10.8)	Metropolitan
Men	Seoul	Yangcheon-gu	11.0 (9.6,12.3)	20.6 (16.4,24.8)	12.3 (9.3,15.3)	8.0 (5.3,10.6)	7.2 (4.7,9.7)	7.2 (4.7,9.7)	13.4 (6.6,15.4)	Metropolitan
Men	Seoul	Gangseo-gu	12.4 (11.0,13.8)	20.4 (16.3,24.5)	14.0 (10.8,17.2)	10.5 (7.6,13.4)	9.9 (7.1,12.7)	7.5 (5.1,10.0)	12.9 (5.1,13.5)	Metropolitan
Men	Seoul	Guro-gu	11.9 (10.5,13.2)	21.6 (17.6,25.7)	9.9 (7.2,12.6)	11.0 (8.1,13.9)	9.3 (6.7,11.8)	7.7 (5.2,10.2)	13.9 (9.1,17.7)	Metropolitan
Men	Seoul	Geumcheon-gu	11.6 (10.3,12.9)	18.0 (14.4,21.6)	11.4 (8.6,14.2)	11.5 (8.7,14.3)	7.7 (5.4,10.0)	9.7 (7.1,12.4)	8.3 (2.7,11.1)	Metropolitan
Men	Seoul	Yeongdeungpo-gu	11.9 (10.6,13.2)	19.3 (15.6,23.1)	12.0 (9.0,14.9)	11.0 (8.2,13.8)	8.9 (6.4,11.5)	8.3 (5.8,10.8)	11.0 (5.9,14.3)	Metropolitan
Men	Seoul	Dongjak-gu	11.6 (10.3,12.9)	17.4 (13.7,21.0)	11.1 (8.2,14.0)	10.1 (7.5,12.7)	10.4 (7.7,13.1)	9.4 (6.8,12.1)	8.0 (2.0,10.4)	Metropolitan
Men	Seoul	Gwanak-gu	11.7 (10.3,13.0)	16.3 (12.7,19.9)	13.4 (10.1,16.7)	11.1 (8.3,13.9)	8.4 (5.8,11.0)	9.8 (6.9,12.6)	6.5 (5.0,13.2)	Metropolitan
Men	Seoul	Seocho-gu	7.5 (6.4,8.6)	10.9 (8.0,13.9)	9.2 (6.5,11.8)	7.0 (4.7,9.3)	5.1 (3.2,7.1)	5.3 (3.1,7.5)	5.6 (4.2,11.2)	Metropolitan
Men	Seoul	Gangnam-gu	7.8 (6.6,8.9)	11.6 (8.3,15.0)	7.4 (4.8,9.9)	7.7 (5.2,10.2)	6.5 (4.1,8.9)	5.9 (3.5,8.3)	5.7 (3.0,10.6)	Metropolitan
Men	Seoul	Songpa-gu	7.8 (6.6,8.9)	12.9 (9.6,16.3)	8.0 (5.3,10.7)	6.8 (4.3,9.2)	7.2 (4.8,9.6)	4.0 (2.2,5.7)	8.9 (5.9,13.1)	Metropolitan
Men	Seoul	Gangdong-gu	11.5 (10.1,12.9)	17.1 (13.4,20.8)	13.5 (10.4,16.7)	9.8 (6.9,12.6)	8.8 (6.1,11.5)	8.2 (5.4,11.0)	8.9 (5.9,14.3)	Metropolitan
Men	Busan	Jung-gu	12.8 (11.5,14.1)	21.5 (17.7,25.4)	13.3 (10.5,16.2)	13.2 (10.2,16.1)	8.8 (6.4,11.1)	7.7 (5.5,9.9)	13.8 (10.0,19.0)	Metropolitan
Men	Busan	Seo-gu	13.5 (12.2,14.8)	23.0 (19.1,26.8)	14.5 (11.5,17.4)	10.2 (7.7,12.7)	9.8 (7.4,12.3)	10.0 (7.5,12.5)	13.0 (7.6,16.8)	Metropolitan
Men	Busan	Dong-gu	16.0 (14.6,17.5)	26.7 (22.4,30.9)	15.3 (12.1,18.5)	13.2 (10.3,16.1)	13.1 (10.2,16.0)	12.2 (9.4,15.1)	14.5 (8.8,18.6)	Metropolitan
Men	Busan	Yeongdo-gu	16.1 (14.7,17.6)	27.8 (23.5,32.1)	17.3 (14.1,20.5)	13.6 (10.6,16.5)	11.6 (9.0,14.2)	11.1 (8.5,13.7)	16.7 (10.4,20.2)	Metropolitan
Men	Busan	Busanjin-gu	11.9 (10.6,13.1)	20.0 (16.3,23.7)	14.2 (11.2,17.3)	10.4 (7.6,13.2)	8.6 (6.1,11.0)	5.8 (3.8,7.9)	14.2 (9.6,17.8)	Metropolitan
Men	Busan	Dongnae-gu	11.3 (10.0,12.5)	15.8 (12.6,19.0)	11.6 (8.6,14.5)	9.7 (7.2,12.3)	9.3 (6.8,11.8)	9.7 (7.0,12.3)	6.1 (4.7,12.7)	Metropolitan
Men	Busan	Nam-gu	11.2 (9.9,12.4)	17.0 (13.6,20.4)	11.8 (9.0,14.6)	10.2 (7.5,12.8)	9.0 (6.6,11.5)	7.9 (5.5,10.3)	9.1 (6.7,14.7)	Metropolitan
Men	Busan	Buk-gu	12.1 (10.7,13.5)	20.8 (16.9,24.8)	10.6 (7.5,13.6)	10.9 (8.0,13.7)	8.2 (5.7,10.7)	10.1 (7.1,13.0)	10.7 (9.0,17.8)	Metropolitan
Men	Busan	Haeundae-gu	11.8 (10.5,13.1)	24.7 (20.4,29.0)	10.9 (8.1,13.7)	10.1 (7.4,12.8)	8.6 (6.1,11.2)	5.3 (3.2,7.3)	19.4 (13.6,22.2)	Metropolitan
Men	Busan	Saha-gu	12.7 (11.4,14.1)	21.5 (17.5,25.5)	12.7 (9.6,15.8)	9.7 (7.1,12.4)	9.8 (7.1,12.5)	10.1 (7.3,13.0)	11.4 (6.7,15.7)	Metropolitan
Men	Busan	Geumjeong-gu	10.9 (9.6,12.1)	20.0 (16.2,23.9)	11.5 (8.6,14.3)	8.4 (6.1,10.7)	7.4 (5.2,9.7)	7.9 (5.5,10.3)	12.1 (10.2,18.4)	Metropolitan
Men	Busan	Gangseo-gu	14.0 (12.7,15.3)	21.1 (17.4,24.8)	13.4 (10.5,16.2)	13.7 (10.7,16.6)	11.4 (8.7,14.2)	10.5 (7.9,13.2)	10.6 (4.5,13.5)	Metropolitan
Men	Busan	Yeonje-gu	12.5 (11.1,13.8)	21.7 (17.8,25.6)	11.1 (8.4,13.8)	10.3 (7.6,13.0)	8.5 (6.2,10.9)	11.1 (8.2,14.0)	10.6 (5.2,14.2)	Metropolitan
Men	Busan	Suyeong-gu	10.9 (9.8,12.1)	17.5 (14.1,20.8)	11.1 (8.4,13.9)	8.2 (6.0,10.5)	10.2 (7.6,12.8)	7.8 (5.4,10.1)	9.7 (5.9,14.3)	Metropolitan
Men	Busan	Sasang-gu	13.5 (12.0,14.9)	18.8 (15.1,22.6)	12.2 (9.2,15.3)	12.5 (9.4,15.6)	12.2 (9.2,15.2)	11.9 (8.8,15.0)	6.9 (1.0,9.8)	Metropolitan
Men	Busan	Gijang-gun	15.0 (13.5,16.4)	21.3 (17.4,25.1)	15.7 (12.3,19.1)	15.2 (12.0,18.3)	13.9 (10.8,17.0)	9.2 (6.6,11.7)	12.1 (5.2,14.2)	Rural
Men	Daegu	Jung-gu	15.8 (14.4,17.3)	24.0 (19.8,28.1)	17.7 (14.5,21.0)	14.8 (11.7,18.0)	12.0 (9.2,14.7)	10.8 (8.0,13.5)	13.2 (7.1,16.3)	Metropolitan
Men	Daegu	Dong-gu	15.7 (14.3,17.2)	22.8 (18.7,26.9)	18.2 (14.8,21.6)	13.9 (10.9,16.8)	13.5 (10.5,16.6)	10.6 (7.9,13.3)	12.2 (7.9,16.9)	Metropolitan
Men	Daegu	Seo-gu	15.1 (13.7,16.6)	21.0 (17.1,24.8)	16.6 (13.0,20.1)	13.6 (10.5,16.6)	14.0 (10.8,17.2)	10.4 (7.6,13.1)	10.6 (7.0,15.6)	Metropolitan
Men	Daegu	Nam-gu	13.1 (11.7,14.4)	21.1 (17.2,25.0)	13.7 (10.7,16.8)	11.6 (8.8,14.4)	10.4 (7.8,12.9)	9.0 (6.5,11.5)	12.1 (7.7,16.5)	Metropolitan
Men	Daegu	Buk-gu	12.5 (11.2,13.9)	20.8 (16.7,24.9)	13.6 (10.3,16.8)	11.8 (8.9,14.7)	8.7 (6.2,11.3)	8.4 (5.8,10.9)	12.4 (10.1,18.5)	Metropolitan

Men	Daegu	Suseong-gu	11.7 (10.4,13.0)	19.1 (15.4,22.8)	12.1 (9.2,15.1)	11.0 (8.3,13.7)	9.0 (6.5,11.6)	7.3 (5.0,9.7)	11.8 (6.1,14.3)	Metropolitan
Men	Daegu	Dalseo-gu	13.1 (11.7,14.5)	21.7 (17.6,25.7)	16.0 (12.3,19.7)	10.1 (7.4,12.9)	9.4 (6.7,12.2)	8.4 (5.8,10.9)	13.3 (7.7,16.5)	Metropolitan
Men	Daegu	Dalseong-gun	14.2 (12.8,15.6)	18.1 (14.4,21.7)	16.1 (12.6,19.6)	14.7 (11.6,17.8)	12.2 (9.1,15.2)	9.9 (7.2,12.7)	8.2 (7.9,16.3)	Rural
Men	Incheon	Jung-gu	12.2 (10.9,13.5)	18.3 (14.7,22.0)	13.5 (10.6,16.4)	12.0 (9.1,14.9)	9.6 (7.1,12.2)	7.7 (5.4,10.1)	10.6 (5.7,14.1)	Metropolitan
Men	Incheon	Dong-gu	12.1 (10.9,13.4)	18.6 (15.0,22.2)	12.9 (9.9,16.0)	13.2 (10.3,16.0)	8.2 (5.9,10.5)	8.2 (5.8,10.5)	10.4 (5.9,14.1)	Metropolitan
Men	Incheon	Nam-gu	10.3 (9.1,11.5)	17.6 (13.9,21.2)	8.7 (6.1,11.2)	8.7 (6.3,11.1)	8.4 (5.9,10.8)	8.7 (6.2,11.2)	8.9 (2.4,10.6)	Metropolitan
Men	Incheon	Yeonsu-gu	11.9 (10.4,13.4)	15.8 (12.0,19.7)	12.4 (8.9,15.9)	10.5 (7.4,13.5)	10.0 (6.9,13.1)	11.0 (7.6,14.3)	4.8 (0.4,9.2)	Metropolitan
Men	Incheon	Namdong-gu	12.2 (10.7,13.7)	18.9 (14.8,23.1)	12.3 (8.8,15.7)	11.7 (8.4,14.9)	7.5 (4.9,10.1)	10.7 (7.6,13.8)	8.2 (7.8,16.8)	Metropolitan
Men	Incheon	Bupyeong-gu	14.3 (12.8,15.9)	24.2 (19.7,28.8)	13.8 (10.4,17.2)	13.7 (10.5,17.0)	10.7 (7.7,13.6)	9.8 (6.8,12.7)	14.4 (10.1,19.5)	Metropolitan
Men	Incheon	Gyeyang-gu	14.1 (12.4,15.8)	21.2 (16.5,25.9)	14.6 (10.7,18.6)	13.4 (9.9,16.9)	11.1 (7.7,14.5)	10.8 (7.4,14.1)	10.4 (5.2,14.8)	Metropolitan
Men	Incheon	Seo-gu	12.8 (11.3,14.4)	18.8 (14.5,23.0)	11.4 (8.2,14.5)	12.1 (8.8,15.4)	11.6 (8.3,14.9)	10.5 (7.4,13.6)	8.3 (5.7,14.3)	Metropolitan
Men	Incheon	Ganghwa-gun	13.5 (12.2,14.8)	20.5 (16.8,24.2)	15.9 (12.8,18.9)	10.9 (8.5,13.3)	9.4 (7.1,11.7)	10.9 (8.3,13.4)	9.6 (3.0,12.2)	Rural
Men	Incheon	Ongjin-gun	10.8 (9.7,12.0)	15.5 (12.3,18.8)	9.8 (7.4,12.2)	10.2 (7.6,12.8)	9.5 (7.1,12.0)	9.0 (6.6,11.4)	6.5 (3.6,11.8)	Rural
Men	Gwangju	Dong-gu	13.0 (11.7,14.3)	21.1 (17.3,24.9)	15.2 (12.1,18.4)	9.9 (7.5,12.3)	10.4 (7.8,13.0)	8.8 (6.3,11.2)	12.3 (8.5,17.3)	Metropolitan
Men	Gwangju	Seo-gu	10.8 (9.4,12.1)	15.3 (11.9,18.8)	12.6 (9.2,15.9)	11.1 (8.1,14.0)	7.6 (5.2,10.0)	7.8 (5.2,10.4)	7.5 (5.6,13.0)	Metropolitan
Men	Gwangju	Nam-gu	12.9 (11.6,14.2)	21.3 (17.4,25.1)	15.1 (12.0,18.2)	9.7 (7.1,12.3)	9.0 (6.6,11.4)	9.8 (7.3,12.4)	11.5 (7.0,15.8)	Metropolitan
Men	Gwangju	Buk-gu	13.5 (12.1,14.9)	19.3 (15.4,23.1)	13.7 (10.6,16.9)	11.6 (8.6,14.6)	12.0 (8.9,15.0)	10.9 (8.0,13.8)	8.4 (5.0,13.6)	Metropolitan
Men	Gwangju	Gwangsan-gu	12.8 (11.2,14.3)	21.1 (16.6,25.5)	14.9 (11.2,18.6)	11.0 (7.8,14.2)	8.2 (5.4,11.0)	8.8 (5.8,11.8)	12.3 (9.1,17.7)	Metropolitan
Men	Daejeon	Dong-gu	12.7 (11.3,14.0)	25.1 (20.9,29.3)	12.2 (9.3,15.0)	9.2 (6.7,11.7)	8.1 (5.6,10.5)	8.9 (6.4,11.4)	16.2 (9.2,18.2)	Metropolitan
Men	Daejeon	Jung-gu	11.3 (10.0,12.5)	18.6 (14.9,22.3)	12.0 (9.2,14.9)	10.1 (7.4,12.9)	9.5 (7.0,11.9)	6.5 (4.4,8.7)	12.1 (6.5,15.1)	Metropolitan
Men	Daejeon	Seo-gu	11.4 (10.0,12.8)	18.7 (14.5,22.8)	13.4 (10.3,16.5)	10.8 (7.7,13.9)	8.5 (5.9,11.2)	6.5 (4.2,8.8)	12.2 (8.9,16.9)	Metropolitan
Men	Daejeon	Yuseong-gu	10.4 (9.0,11.8)	15.5 (11.9,19.1)	12.4 (8.9,15.8)	8.9 (6.1,11.7)	7.2 (4.5,9.8)	7.1 (4.3,9.8)	8.4 (4.0,12.0)	Metropolitan
Men	Daejeon	Daedeok-gu	11.0 (9.7,12.4)	16.7 (13.0,20.5)	13.0 (9.9,16.1)	8.8 (6.1,11.6)	9.4 (6.5,12.2)	7.2 (4.6,9.8)	9.5 (5.9,14.1)	Metropolitan
Men	Ulsan	Jung-gu	13.9 (12.4,15.4)	20.8 (16.8,24.7)	13.0 (9.7,16.4)	11.7 (8.5,14.9)	11.9 (8.9,14.8)	11.9 (8.8,15.0)	8.9 (7.1,16.1)	Metropolitan
Men	Ulsan	Nam-gu	10.4 (9.0,11.8)	13.9 (10.1,17.8)	10.8 (7.6,13.9)	11.1 (7.9,14.2)	8.9 (5.9,11.9)	7.9 (5.3,10.4)	6.0 (2.5,9.9)	Metropolitan
Men	Ulsan	Dong-gu	10.0 (8.6,11.4)	17.3 (13.1,21.4)	10.5 (7.2,13.8)	8.8 (6.0,11.5)	6.6 (4.1,9.0)	7.6 (4.7,10.5)	9.7 (6.6,14.0)	Metropolitan
Men	Ulsan	Buk-gu	10.3 (8.8,11.8)	14.4 (10.5,18.4)	11.9 (8.3,15.4)	9.3 (6.0,12.5)	7.5 (4.6,10.4)	8.8 (5.5,12.0)	5.6 (2.6,10.2)	Metropolitan
Men	Ulsan	Ulju-gun	14.9 (13.4,16.3)	23.9 (19.6,28.2)	16.1 (12.7,19.6)	12.6 (9.6,15.6)	10.0 (7.3,12.8)	12.2 (9.2,15.2)	11.7 (5.5,14.9)	Rural
Men	Sejong	Sejong	15.1 (13.7,16.5)	24.3 (20.2,28.4)	14.8 (11.8,17.8)	15.0 (11.9,18.1)	11.6 (8.9,14.2)	9.9 (7.3,12.5)	14.4 (9.3,18.5)	Metropolitan
Men	Gyeonggi-do	Jangan-gu	10.8 (9.5,12.2)	18.2 (14.2,22.2)	12.5 (9.3,15.7)	10.0 (7.3,12.6)	7.2 (4.8,9.7)	6.3 (3.9,8.6)	11.9 (8.1,16.5)	Metropolitan
Men	Gyeonggi-do	Gwonseon-gu	10.7 (9.3,12.1)	17.4 (13.4,21.5)	9.9 (6.9,13.0)	10.7 (7.7,13.7)	8.6 (5.9,11.3)	6.9 (4.3,9.5)	10.5 (7.0,14.8)	Metropolitan
Men	Gyeonggi-do	Paldal-gu	11.0 (9.7,12.4)	16.2 (12.6,19.9)	11.3 (8.4,14.2)	8.5 (5.9,11.2)	9.6 (6.9,12.3)	9.6 (6.8,12.3)	6.6 (1.2,11.0)	Metropolitan
Men	Gyeonggi-do	Yeongtong-gu	9.6 (8.1,11.2)	11.9 (8.1,15.7)	9.9 (6.3,13.5)	8.2 (5.2,11.1)	8.9 (5.6,12.1)	9.0 (5.3,12.6)	2.9 (-1.1,6.5)	Metropolitan
Men	Gyeonggi-do	Sujeong-gu	13.5 (12.0,14.9)	20.4 (16.4,24.3)	14.3 (11.0,17.5)	12.7 (9.6,15.9)	10.0 (7.3,12.6)	10.1 (7.3,12.9)	10.3 (5.9,14.5)	Metropolitan
Men	Gyeonggi-do	Jungwon-gu	13.5 (12.0,14.9)	22.9 (18.7,27.0)	13.2 (10.1,16.4)	11.7 (8.7,14.8)	10.1 (7.3,12.9)	9.7 (7.0,12.4)	13.2 (7.1,16.1)	Metropolitan
Men	Gyeonggi-do	Bundang-gu	6.2 (5.2,7.2)	9.1 (6.4,11.8)	5.1 (3.1,7.1)	6.4 (4.2,8.6)	5.3 (3.3,7.4)	5.2 (3.1,7.3)	3.9 (-0.3,5.7)	Metropolitan
Men	Gyeonggi-do	Uijeongbu-si	12.6 (11.2,14.0)	20.0 (16.0,24.0)	13.0 (9.8,16.2)	10.9 (8.0,13.7)	9.7 (7.1,12.4)	9.7 (7.0,12.5)	10.3 (4.6,13.6)	Urban

Volume: 39, Article ID: e2017011,

https://doi.org/10.4178/epih.e2017011

Men	Gyeonggi-do	Manan-gu	10.7 (9.4,11.9)	17.6 (14.0,21.3)	13.2 (10.1,16.3)	9.1 (6.5,11.7)	7.2 (4.8,9.5)	6.3 (4.1,8.6)	11.3 (7.3,15.3)	Metropolitan
Men	Gyeonggi-do	Dongan-gu	9.2 (7.9,10.5)	13.7 (10.0,17.4)	8.3 (5.4,11.1)	8.6 (6.0,11.3)	6.2 (3.9,8.5)	9.4 (6.5,12.3)	4.3 (0.1,7.7)	Metropolitan
Men	Gyeonggi-do	Wonmi-gu	11.2 (9.8,12.7)	16.2 (12.3,20.0)	11.5 (8.3,14.7)	8.5 (5.7,11.4)	9.8 (6.9,12.7)	10.3 (7.1,13.4)	5.9 (2.2,10.8)	Metropolitan
Men	Gyeonggi-do	Sosa-gu	11.7 (10.3,13.1)	17.2 (13.3,21.1)	12.7 (9.6,15.8)	9.6 (6.9,12.4)	10.2 (7.4,13.1)	9.2 (6.4,12.1)	8.0 (5.8,14.4)	Metropolitan
Men	Gyeonggi-do	Ojeong-gu	12.5 (11.1,13.9)	21.8 (17.7,26.0)	11.4 (8.4,14.5)	9.7 (6.9,12.4)	9.8 (7.0,12.6)	9.8 (6.8,12.8)	12.0 (6.5,15.3)	Metropolitan
Men	Gyeonggi-do	Gwangmyeong-si	11.2 (9.9,12.5)	13.9 (10.4,17.3)	11.9 (8.9,14.9)	11.7 (8.6,14.7)	8.9 (6.2,11.6)	10.0 (7.1,12.9)	3.9 (2.0,10.4)	Urban
Men	Gyeonggi-do	Pyeongtaek-si	12.0 (11.1,12.9)	19.2 (16.6,21.8)	12.5 (10.3,14.6)	10.0 (8.1,11.9)	9.8 (7.9,11.7)	8.7 (6.9,10.4)	10.5 (8.5,14.5)	Urban
Men	Gyeonggi-do	Dongducheon-si	13.2 (11.8,14.5)	19.5 (15.8,23.2)	15.0 (11.8,18.2)	13.6 (10.7,16.5)	9.2 (6.7,11.8)	8.8 (6.4,11.2)	10.7 (6.9,15.7)	Urban
Men	Gyeonggi-do	Sangnok-gu	11.1 (9.7,12.5)	18.2 (14.1,22.2)	10.0 (6.9,13.1)	9.0 (6.0,11.9)	9.5 (6.6,12.3)	8.6 (5.8,11.4)	9.6 (7.9,16.5)	Metropolitan
Men	Gyeonggi-do	Danwon-gu	11.7 (10.2,13.2)	17.9 (13.6,22.2)	12.4 (9.1,15.8)	10.2 (7.1,13.3)	9.6 (6.5,12.6)	8.7 (5.7,11.6)	9.2 (3.5,11.7)	Metropolitan
Men	Gyeonggi-do	Deogyang-gu	12.8 (11.4,14.1)	21.8 (17.7,25.9)	13.2 (10.2,16.3)	9.5 (6.9,12.2)	9.2 (6.6,11.8)	10.3 (7.5,13.2)	11.5 (5.1,13.9)	Metropolitan
Men	Gyeonggi-do	Ilsandong-gu	9.4 (8.2,10.7)	13.5 (10.0,17.1)	10.1 (7.3,13.0)	10.1 (7.3,12.9)	8.0 (5.5,10.5)	5.8 (3.5,8.2)	7.7 (4.3,12.1)	Metropolitan
Men	Gyeonggi-do	Ilsanseo-gu	9.5 (8.3,10.8)	15.5 (11.9,19.1)	8.7 (5.9,11.4)	9.5 (6.9,12.1)	7.5 (5.1,9.9)	6.8 (4.5,9.1)	8.7 (2.6,10.6)	Metropolitan
Men	Gyeonggi-do	Gwacheon-si	8.8 (7.6,9.9)	13.3 (9.9,16.7)	8.4 (5.9,10.9)	7.6 (5.1,10.0)	8.9 (6.3,11.4)	5.8 (3.7,7.9)	7.5 (3.2,10.6)	Urban
Men	Gyeonggi-do	Guri-si	13.2 (11.7,14.7)	19.9 (15.8,23.9)	13.8 (10.5,17.1)	10.7 (7.7,13.6)	12.5 (9.4,15.7)	9.0 (6.2,11.8)	10.9 (4.4,13.4)	Urban
Men	Gyeonggi-do	Namyangju-si	13.1 (11.7,14.5)	18.7 (14.9,22.5)	13.0 (9.9,16.1)	11.8 (8.8,14.8)	11.6 (8.5,14.6)	10.5 (7.5,13.4)	8.2 (4.0,12.8)	Urban
Men	Gyeonggi-do	Osan-si	11.4 (9.9,12.9)	15.8 (11.8,19.8)	10.4 (7.4,13.5)	10.5 (7.4,13.6)	12.5 (9.0,16.0)	8.2 (5.4,10.9)	7.6 (4.1,12.1)	Urban
Men	Gyeonggi-do	Siheung-si	11.4 (9.9,12.9)	18.6 (14.4,22.8)	11.9 (8.6,15.2)	7.6 (4.9,10.3)	9.2 (6.1,12.4)	9.8 (6.7,12.8)	8.8 (4.8,13.2)	Urban
Men	Gyeonggi-do	Gunpo-si	11.8 (10.4,13.2)	18.3 (14.3,22.3)	11.0 (8.1,13.9)	9.2 (6.3,12.0)	11.4 (8.4,14.5)	9.5 (6.7,12.3)	8.8 (1.4,10.0)	Urban
Men	Gyeonggi-do	Uiwang-si	10.2 (8.9,11.4)	14.7 (11.3,18.1)	11.6 (8.5,14.8)	7.2 (4.8,9.6)	9.6 (6.9,12.2)	8.3 (5.8,10.9)	6.4 (3.5,11.5)	Urban
Men	Gyeonggi-do	Hanam-si	12.7 (11.3,14.1)	17.9 (14.2,21.6)	13.5 (10.2,16.8)	11.2 (8.3,14.1)	11.4 (8.5,14.3)	10.1 (7.2,13.0)	7.8 (3.7,12.3)	Urban
Men	Gyeonggi-do	Cheoin-gu	10.7 (9.5,12.0)	14.6 (11.3,17.8)	13.7 (10.5,16.8)	7.8 (5.5,10.0)	10.7 (7.9,13.5)	7.5 (5.2,9.8)	7.1 (0.5,8.3)	Metropolitan
Men	Gyeonggi-do	Giheung-gu	8.4 (7.3,9.6)	13.7 (10.3,17.0)	8.1 (5.6,10.6)	7.7 (5.3,10.0)	7.8 (5.3,10.4)	5.1 (3.2,7.0)	8.6 (3.4,10.4)	Metropolitan
Men	Gyeonggi-do	Suji-gu	7.9 (6.8,8.9)	10.1 (7.3,12.8)	9.1 (6.5,11.8)	7.3 (5.1,9.6)	6.7 (4.5,8.9)	6.1 (3.9,8.2)	4.0 (1.3,8.3)	Metropolitan
Men	Gyeonggi-do	Paju-si	12.3 (11.0,13.7)	19.3 (15.5,23.1)	13.2 (10.1,16.2)	10.6 (7.9,13.4)	11.0 (8.2,13.8)	8.1 (5.7,10.5)	11.2 (5.1,13.7)	Urban
Men	Gyeonggi-do	Icheon-si	12.5 (11.2,13.9)	16.8 (13.3,20.2)	12.0 (9.0,15.0)	12.4 (9.4,15.3)	11.0 (8.3,13.7)	10.5 (7.7,13.3)	6.3 (3.4,11.8)	Urban
Men	Gyeonggi-do	Anseong-si	12.2 (10.9,13.4)	18.1 (14.7,21.5)	12.7 (9.8,15.7)	10.0 (7.4,12.6)	10.3 (7.7,12.9)	9.4 (6.9,11.9)	8.7 (5.0,13.4)	Urban
Men	Gyeonggi-do	Gimpo-si	10.3 (9.1,11.5)	15.4 (12.1,18.8)	10.3 (7.7,13.0)	10.0 (7.3,12.6)	8.6 (6.2,11.0)	7.6 (5.2,9.9)	7.8 (4.3,11.9)	Urban
Men	Gyeonggi-do	Hwaseongsi	11.4 (10.0,12.7)	16.6 (13.0,20.2)	13.1 (9.8,16.5)	10.3 (7.4,13.1)	8.2 (5.6,10.7)	9.0 (6.2,11.9)	7.6 (5.2,13.2)	Urban
Men	Gyeonggi-do	Gwangju-si	11.8 (10.5,13.1)	16.0 (12.7,19.4)	12.3 (9.3,15.2)	10.9 (8.2,13.6)	10.0 (7.3,12.7)	9.6 (7.0,12.3)	6.4 (2.5,10.7)	Urban
Men	Gyeonggi-do	Yangju-si	13.1 (11.7,14.4)	20.1 (16.3,24.0)	12.0 (9.1,14.9)	10.3 (7.5,13.0)	12.0 (9.2,14.9)	11.3 (8.3,14.2)	8.8 (3.6,12.2)	Urban
Men	Gyeonggi-do	Pocheon-si	14.0 (12.7,15.3)	23.0 (18.9,27.1)	13.3 (10.5,16.1)	13.2 (10.3,16.1)	10.5 (7.9,13.1)	10.6 (8.0,13.3)	12.4 (6.3,15.3)	Urban
Men	Gyeonggi-do	Yeoju-gun	13.7 (12.4,15.0)	20.1 (16.6,23.6)	13.9 (11.0,16.8)	11.3 (8.7,14.0)	12.3 (9.6,15.0)	11.3 (8.6,13.9)	8.8 (1.2,10.2)	Urban
Men	Gyeonggi-do	Yeoncheon-gun	13.4 (12.1,14.7)	22.6 (18.8,26.4)	14.4 (11.5,17.2)	11.3 (8.9,13.8)	10.4 (7.9,12.9)	9.0 (6.8,11.2)	13.6 (6.1,14.9)	Rural
Men	Gyeonggi-do	Gapyeong-gun	12.5 (11.3,13.7)	21.5 (17.8,25.3)	13.5 (10.7,16.3)	11.5 (9.0,14.0)	8.2 (6.0,10.3)	8.0 (5.8,10.1)	13.5 (7.7,16.3)	Rural
Men	Gyeonggi-do	Yangpyeong-gun	12.9 (11.6,14.1)	18.3 (14.9,21.6)	15.2 (12.2,18.1)	10.2 (7.7,12.6)	12.4 (9.8,15.0)	8.1 (5.9,10.3)	10.2 (6.6,15.2)	Rural
Men	Gangwon-do	Chuncheon-si	11.5 (10.3,12.8)	22.5 (18.3,26.6)	12.6 (9.7,15.5)	8.8 (6.3,11.3)	8.0 (5.7,10.4)	7.1 (4.9,9.3)	15.4 (8.6,17.2)	Urban

Volume: 39, Article ID: e2017011,

https://doi.org/10.4178/epih.e2017011

Men	Gangwon-do	Wonju-si	11.4 (10.2,12.7)	18.4 (14.7,22.1)	12.9 (10.0,15.8)	8.8 (6.3,11.4)	9.9 (7.4,12.5)	7.4 (5.0,9.7)	11.0 (5.8,14.2)	Urban
Men	Gangwon-do	Gangneung-si	13.6 (12.2,14.9)	21.9 (17.9,25.9)	13.4 (10.6,16.3)	12.5 (9.8,15.2)	11.7 (8.9,14.4)	8.9 (6.4,11.3)	13.0 (9.8,18.6)	Urban
Men	Gangwon-do	Donghae-si	12.2 (10.9,13.4)	20.3 (16.6,24.0)	12.4 (9.5,15.3)	10.7 (8.1,13.4)	7.6 (5.4,9.9)	10.0 (7.4,12.7)	10.3 (6.1,14.9)	Urban
Men	Gangwon-do	Taebaek-si	18.7 (17.2,20.2)	26.1 (21.9,30.2)	21.0 (17.4,24.5)	18.0 (14.6,21.4)	14.3 (11.4,17.2)	14.7 (11.7,17.8)	11.4 (6.7,16.5)	Urban
Men	Gangwon-do	Sokcho-si	14.7 (13.2,16.1)	24.3 (20.1,28.5)	15.6 (12.3,18.8)	13.1 (10.2,16.1)	10.2 (7.5,12.8)	10.3 (7.5,13.1)	14.0 (8.8,18.4)	Urban
Men	Gangwon-do	Samcheok-si	15.1 (13.7,16.4)	26.6 (22.4,30.8)	16.2 (13.1,19.3)	12.3 (9.6,14.9)	11.1 (8.5,13.7)	9.9 (7.5,12.3)	16.7 (9.3,18.7)	Urban
Men	Gangwon-do	Hongcheon-gun	12.4 (11.2,13.6)	19.9 (16.4,23.3)	12.2 (9.6,14.8)	11.2 (8.6,13.7)	9.3 (7.1,11.4)	9.4 (7.0,11.8)	10.5 (7.9,16.7)	Rural
Men	Gangwon-do	Hoengseong-gun	14.3 (13.0,15.6)	19.9 (16.5,23.4)	15.6 (12.6,18.5)	13.8 (11.1,16.6)	12.3 (9.7,14.9)	10.1 (7.6,12.7)	9.8 (5.3,14.1)	Rural
Men	Gangwon-do	Yeongwol-gun	15.2 (13.9,16.6)	24.5 (20.5,28.5)	16.3 (13.3,19.3)	12.7 (10.0,15.3)	12.1 (9.5,14.7)	10.9 (8.4,13.4)	13.6 (9.6,18.6)	Rural
Men	Gangwon-do	Pyeongchang-gun	14.7 (13.4,16.1)	23.6 (19.7,27.5)	16.1 (13.0,19.2)	12.6 (10.0,15.3)	11.7 (9.1,14.3)	9.9 (7.5,12.4)	13.7 (7.3,16.3)	Rural
Men	Gangwon-do	Jeongseon-gun	17.8 (16.4,19.3)	25.6 (21.6,29.6)	19.2 (15.9,22.5)	18.0 (14.8,21.1)	14.9 (12.0,17.7)	11.7 (9.0,14.4)	13.9 (11.2,20.6)	Rural
Men	Gangwon-do	Cheorwon-gun	11.9 (10.7,13.1)	22.1 (18.4,25.8)	12.2 (9.6,14.8)	8.9 (6.5,11.3)	8.8 (6.5,11.2)	7.5 (5.3,9.6)	14.6 (9.7,18.3)	Rural
Men	Gangwon-do	Hwacheon-gun	13.7 (12.4,15.1)	21.5 (17.5,25.5)	14.8 (11.8,17.9)	11.3 (8.5,14.1)	12.7 (9.9,15.6)	8.6 (6.2,11.0)	12.9 (8.2,17.2)	Rural
Men	Gangwon-do	Yanggu-gun	13.8 (12.5,15.2)	22.2 (18.2,26.1)	13.6 (10.5,16.7)	13.2 (10.4,16.1)	11.3 (8.5,14.0)	9.1 (6.6,11.5)	13.1 (7.5,16.5)	Rural
Men	Gangwon-do	Inje-gun	13.2 (11.9,14.4)	19.5 (15.9,23.0)	13.7 (10.8,16.5)	13.5 (10.6,16.3)	9.7 (7.3,12.1)	9.5 (7.0,12.0)	10.0 (5.9,14.5)	Rural
Men	Gangwon-do	Goseong-gun	13.7 (12.4,15.0)	20.5 (16.8,24.2)	18.1 (14.6,21.6)	13.0 (10.1,15.9)	9.2 (6.9,11.5)	8.0 (5.8,10.3)	12.5 (8.3,17.1)	Rural
Men	Gangwon-do	Yangyang-gun	13.3 (12.0,14.5)	22.2 (18.4,26.0)	16.1 (13.0,19.2)	10.0 (7.5,12.4)	10.5 (8.0,13.0)	8.0 (5.9,10.1)	14.2 (8.8,17.8)	Rural
Men	Chungcheongbuk-do	Cheongju-si	11.0 (10.3,11.8)	17.4 (15.2,19.6)	12.6 (10.8,14.5)	9.1 (7.6,10.7)	9.1 (7.6,10.5)	7.8 (6.4,9.2)	9.6 (7.1,11.9)	Metropolitan
Men	Chungcheongbuk-do	Chungju-si	15.1 (13.7,16.5)	23.3 (19.4,27.3)	17.9 (14.6,21.2)	11.5 (8.9,14.2)	11.8 (9.1,14.4)	11.2 (8.5,13.9)	12.1 (8.6,17.8)	Urban
Men	Chungcheongbuk-do	Jecheon-si	12.4 (11.1,13.6)	19.7 (16.2,23.3)	12.5 (9.7,15.2)	10.9 (8.3,13.5)	9.7 (7.3,12.2)	9.5 (7.0,12.0)	10.2 (5.9,14.7)	Urban
Men	Chungcheongbuk-do	Boeun-gun	15.0 (13.7,16.3)	24.7 (20.7,28.8)	17.8 (14.6,21.1)	12.1 (9.6,14.5)	9.8 (7.5,12.2)	11.8 (9.1,14.5)	12.9 (9.1,18.5)	Rural
Men	Chungcheongbuk-do	Okcheon-gun	13.3 (12.0,14.6)	19.6 (16.0,23.1)	16.1 (13.0,19.3)	11.1 (8.6,13.6)	11.1 (8.6,13.7)	8.7 (6.3,11.0)	10.9 (5.0,13.8)	Rural
Men	Chungcheongbuk-do	Yeongdong-gun	13.0 (11.8,14.2)	22.0 (18.3,25.7)	12.9 (10.3,15.5)	10.3 (8.0,12.6)	10.3 (7.8,12.7)	9.6 (7.3,12.0)	12.4 (7.2,16.2)	Rural
Men	Chungcheongbuk-do	Jincheon-gun	13.9 (12.5,15.2)	20.7 (16.8,24.5)	11.2 (8.5,13.8)	14.3 (11.2,17.5)	11.6 (8.9,14.3)	12.3 (9.4,15.2)	8.4 (2.0,11.2)	Rural
Men	Chungcheongbuk-do	Goesan-gun	11.3 (10.1,12.5)	16.0 (12.8,19.2)	11.2 (8.6,13.8)	10.9 (8.2,13.6)	9.6 (7.1,12.0)	8.9 (6.5,11.3)	7.1 (2.1,10.3)	Rural
Men	Chungcheongbuk-do	Eumseong-gun	14.1 (12.8,15.3)	22.0 (18.0,25.9)	14.8 (11.9,17.8)	14.0 (11.4,16.6)	10.2 (7.8,12.5)	9.9 (7.5,12.3)	12.1 (7.7,16.5)	Rural
Men	Chungcheongbuk-do	Danyang-gun	14.6 (13.2,15.9)	22.9 (19.2,26.7)	13.3 (10.4,16.2)	13.0 (10.2,15.7)	10.1 (7.6,12.5)	13.3 (10.4,16.2)	9.6 (2.2,11.6)	Rural
Men	Chungcheongbuk-do	Jeungpyeong-gun	12.9 (11.7,14.1)	24.0 (20.1,28.0)	12.4 (9.9,15.0)	11.7 (9.3,14.1)	10.2 (7.8,12.5)	6.6 (4.7,8.5)	17.4 (11.9,20.5)	Rural
Men	Chungcheongnam-do	Cheonan-si	13.8 (12.2,15.3)	19.8 (15.7,23.9)	16.1 (12.4,19.8)	12.3 (9.1,15.5)	12.2 (9.1,15.4)	8.5 (5.9,11.1)	11.3 (8.0,16.6)	Metropolitan
Men	Chungcheongnam-do	Gongju-si	14.1 (12.8,15.3)	24.9 (21.0,28.9)	16.1 (13.0,19.2)	12.3 (9.6,14.9)	9.3 (7.1,11.4)	7.8 (5.7,9.9)	17.1 (13.0,21.8)	Urban
Men	Chungcheongnam-do	Boryeong-si	16.8 (15.4,18.2)	28.3 (23.9,32.7)	18.9 (15.6,22.1)	12.9 (10.1,15.7)	12.7 (9.9,15.4)	12.1 (9.4,14.9)	16.2 (11.4,21.2)	Urban
Men	Chungcheongnam-do	Asan-si	12.5 (11.2,13.8)	21.6 (17.5,25.6)	11.5 (8.7,14.4)	11.1 (8.2,14.0)	10.4 (7.7,13.1)	7.9 (5.6,10.3)	13.7 (7.1,15.3)	Urban
Men	Chungcheongnam-do	Seosan-si	13.4 (12.1,14.7)	20.0 (16.4,23.7)	14.0 (11.0,16.9)	12.4 (9.6,15.1)	11.4 (8.7,14.1)	9.1 (6.7,11.5)	10.9 (7.0,15.4)	Urban
Men	Chungcheongnam-do	Nonsan-si	14.6 (13.3,15.9)	24.3 (20.4,28.3)	16.3 (13.3,19.4)	12.1 (9.3,14.8)	10.6 (8.2,13.0)	9.4 (6.9,11.8)	14.9 (8.8,17.8)	Urban
Men	Chungcheongnam-do	Gyeryong-si	10.3 (8.8,11.7)	15.1 (11.0,19.1)	12.1 (8.6,15.6)	9.8 (6.6,13.0)	8.4 (5.4,11.3)	6.2 (3.7,8.8)	8.9 (5.3,12.7)	Urban
Men	Chungcheongnam-do	Dangjin-si	13.4 (12.1,14.7)	21.2 (17.5,24.8)	11.1 (8.4,13.8)	12.5 (9.8,15.3)	12.5 (9.8,15.3)	9.9 (7.4,12.4)	11.3 (5.0,14.0)	Rural
Men	Chungcheongnam-do	Geumsan-gun	14.1 (12.9,15.4)	21.0 (17.4,24.6)	16.9 (13.6,20.1)	12.7 (10.1,15.3)	10.8 (8.4,13.2)	9.4 (7.1,11.7)	11.6 (6.1,15.1)	Rural

Men	Chungcheongnam-do	Buyeo-gun	13.5 (12.3,14.8)	23.3 (19.2,27.3)	15.2 (12.3,18.2)	10.3 (8.1,12.5)	10.6 (8.2,13.0)	8.8 (6.6,10.9)	14.5 (9.5,18.5)	Rural
Men	Chungcheongnam-do	Seocheon-gun	14.9 (13.5,16.3)	24.0 (19.9,28.1)	16.4 (13.3,19.5)	12.8 (10.1,15.6)	10.0 (7.6,12.4)	11.9 (9.1,14.7)	12.1 (5.8,15.4)	Rural
Men	Chungcheongnam-do	Cheongyang-gun	13.2 (12.0,14.4)	23.0 (19.1,26.9)	13.7 (11.1,16.4)	11.6 (9.2,13.9)	10.1 (7.8,12.5)	8.1 (6.1,10.2)	14.9 (11.0,19.8)	Rural
Men	Chungcheongnam-do	Hongseong-gun	14.7 (13.4,16.1)	23.4 (19.6,27.2)	14.6 (11.7,17.5)	14.0 (11.1,16.9)	11.4 (8.8,14.0)	10.5 (7.9,13.0)	12.9 (8.8,18.0)	Rural
Men	Chungcheongnam-do	Yesan-gun	12.8 (11.6,14.0)	20.9 (17.3,24.6)	12.9 (10.2,15.7)	11.3 (8.9,13.8)	9.1 (6.9,11.3)	10.1 (7.6,12.5)	10.8 (6.6,15.6)	Rural
Men	Chungcheongnam-do	Taean-gun	13.3 (12.0,14.5)	17.8 (14.5,21.1)	14.7 (11.7,17.7)	12.8 (10.2,15.5)	11.1 (8.6,13.7)	10.2 (7.8,12.6)	7.6 (2.2,11.0)	Urban
Men	Jeollabuk-do	Jeonju-si	11.4 (10.1,12.7)	18.8 (15.0,22.6)	12.6 (9.6,15.6)	9.1 (6.6,11.7)	9.0 (6.4,11.6)	8.3 (5.9,10.7)	10.5 (6.3,14.9)	Metropolitan
Men	Jeollabuk-do	Gunsan-si	14.6 (13.2,16.0)	22.1 (18.2,26.0)	12.9 (9.8,15.9)	13.2 (10.3,16.2)	13.1 (10.1,16.1)	12.3 (9.4,15.1)	9.8 (5.6,14.8)	Urban
Men	Jeollabuk-do	Iksan-si	15.2 (13.8,16.6)	25.7 (21.5,29.9)	15.6 (12.5,18.7)	14.3 (11.4,17.3)	10.8 (8.1,13.4)	10.1 (7.6,12.6)	15.6 (12.2,21.6)	Urban
Men	Jeollabuk-do	Jeongeup-si	16.4 (14.9,17.8)	26.2 (22.0,30.5)	18.0 (14.7,21.3)	14.8 (11.9,17.7)	11.5 (8.9,14.1)	11.8 (9.0,14.6)	14.4 (8.2,18.0)	Urban
Men	Jeollabuk-do	Namwon-si	15.2 (13.8,16.5)	23.7 (19.7,27.6)	18.0 (14.7,21.4)	13.7 (10.9,16.6)	9.8 (7.4,12.1)	11.0 (8.5,13.6)	12.7 (9.5,18.7)	Urban
Men	Jeollabuk-do	Gimje-si	15.7 (14.4,17.1)	25.7 (21.6,29.8)	15.9 (12.9,18.9)	14.0 (11.2,16.9)	12.9 (10.2,15.6)	10.6 (8.0,13.2)	15.1 (9.1,18.7)	Urban
Men	Jeollabuk-do	Wanju-gun	13.3 (12.1,14.5)	18.9 (15.5,22.2)	17.8 (14.5,21.0)	11.9 (9.4,14.3)	8.5 (6.3,10.7)	9.6 (7.3,11.9)	9.3 (6.2,14.8)	Rural
Men	Jeollabuk-do	Jinan-gun	13.2 (11.9,14.4)	19.9 (16.4,23.5)	15.6 (12.5,18.6)	11.3 (8.9,13.8)	10.6 (8.2,13.0)	8.2 (6.1,10.3)	11.7 (7.9,17.1)	Rural
Men	Jeollabuk-do	Muju-gun	14.2 (12.8,15.5)	23.4 (19.4,27.4)	15.4 (12.5,18.4)	12.8 (10.1,15.6)	10.3 (7.9,12.7)	8.4 (6.1,10.6)	15.0 (10.2,19.6)	Rural
Men	Jeollabuk-do	Jangsu-gun	15.5 (14.2,16.9)	24.6 (20.4,28.7)	17.0 (13.8,20.2)	13.3 (10.5,16.0)	11.7 (9.2,14.2)	11.6 (9.0,14.2)	13.0 (6.8,16.6)	Rural
Men	Jeollabuk-do	Imsil-gun	15.1 (13.7,16.4)	23.6 (19.4,27.9)	14.8 (12.1,17.5)	15.2 (12.2,18.2)	13.5 (10.8,16.3)	8.1 (6.1,10.2)	15.5 (13.6,22.6)	Rural
Men	Jeollabuk-do	Sunchang-gun	14.9 (13.5,16.3)	22.6 (18.8,26.5)	19.8 (16.2,23.4)	12.4 (9.9,14.9)	11.3 (8.6,14.0)	8.3 (6.1,10.4)	14.3 (11.0,20.4)	Rural
Men	Jeollabuk-do	Gochang-gun	17.9 (16.4,19.4)	29.8 (25.1,34.6)	18.7 (15.3,22.1)	14.8 (11.9,17.7)	16.2 (13.0,19.4)	10.7 (8.2,13.1)	19.1 (13.7,23.5)	Rural
Men	Jeollabuk-do	Buan-gun	17.2 (15.7,18.6)	29.1 (24.7,33.6)	19.1 (15.5,22.7)	15.9 (12.8,18.9)	9.8 (7.4,12.1)	11.5 (8.8,14.1)	17.6 (12.4,22.8)	Rural
Men	Jeollanam-do	Mokpo-si	14.0 (12.6,15.4)	24.2 (20.0,28.4)	15.9 (12.5,19.3)	12.5 (9.6,15.4)	10.4 (7.7,13.1)	7.3 (5.0,9.6)	16.9 (9.8,18.6)	Urban
Men	Jeollanam-do	Yeosu-si	13.2 (11.9,14.5)	21.6 (17.8,25.4)	13.5 (10.7,16.3)	9.8 (7.3,12.4)	10.2 (7.6,12.8)	11.0 (8.2,13.8)	10.6 (5.6,14.6)	Urban
Men	Jeollanam-do	Suncheon-si	12.2 (10.9,13.4)	20.1 (16.4,23.8)	13.9 (10.8,17.0)	8.8 (6.4,11.2)	8.1 (5.7,10.5)	10.0 (7.4,12.6)	10.1 (7.3,16.3)	Urban
Men	Jeollanam-do	Naju-si	14.9 (13.6,16.3)	26.5 (22.4,30.7)	14.5 (11.6,17.4)	13.3 (10.5,16.1)	11.1 (8.6,13.5)	9.2 (6.9,11.5)	17.3 (13.1,22.5)	Urban
Men	Jeollanam-do	Gwangyang-si	11.2 (9.9,12.5)	18.0 (14.2,21.7)	12.5 (9.4,15.5)	9.9 (6.9,12.8)	7.9 (5.4,10.3)	8.2 (5.5,10.8)	9.8 (4.2,12.2)	Urban
Men	Jeollanam-do	Damyang-gun	14.7 (13.4,16.0)	25.7 (21.6,29.9)	15.6 (12.5,18.6)	13.4 (10.8,16.0)	9.5 (7.4,11.7)	9.6 (7.2,12.0)	16.1 (12.1,21.1)	Rural
Men	Jeollanam-do	Gokseong-gun	14.1 (12.8,15.5)	20.9 (17.0,24.8)	14.9 (11.8,17.9)	13.9 (11.0,16.7)	9.1 (6.8,11.3)	12.4 (9.6,15.3)	8.5 (3.9,13.5)	Rural
Men	Jeollanam-do	Gurye-gun	11.7 (10.5,12.9)	23.1 (19.1,27.1)	13.8 (10.9,16.8)	7.4 (5.5,9.3)	9.0 (6.7,11.2)	5.9 (4.1,7.8)	17.2 (11.2,20.2)	Rural
Men	Jeollanam-do	Goheung-gun	14.4 (13.1,15.8)	30.2 (25.3,35.1)	13.3 (10.6,16.0)	11.8 (9.4,14.2)	11.1 (8.6,13.6)	7.0 (5.1,8.8)	23.2 (18.9,28.1)	Rural
Men	Jeollanam-do	Boseong-gun	15.2 (13.8,16.6)	25.5 (21.1,29.8)	19.3 (15.9,22.8)	12.8 (10.2,15.3)	10.4 (8.0,12.8)	7.8 (5.9,9.8)	17.7 (13.7,23.5)	Rural
Men	Jeollanam-do	Hwasun-gun	15.9 (14.5,17.3)	25.5 (21.3,29.6)	16.0 (13.0,19.1)	15.2 (12.2,18.2)	10.8 (8.2,13.4)	12.2 (9.4,15.0)	13.3 (8.1,18.1)	Rural
Men	Jeollanam-do	Jangheung-gun	15.2 (13.8,16.6)	29.8 (25.0,34.5)	15.9 (12.9,18.9)	12.5 (9.8,15.2)	9.4 (7.3,11.5)	9.4 (6.8,11.9)	20.4 (12.9,22.5)	Rural
Men	Jeollanam-do	Gangjin-gun	12.0 (10.8,13.2)	21.0 (17.1,25.0)	14.7 (11.6,17.8)	10.9 (8.4,13.4)	9.2 (6.8,11.5)	4.9 (3.4,6.4)	16.1 (10.8,19.2)	Rural
Men	Jeollanam-do	Haenam-gun	15.6 (14.2,17.0)	26.2 (21.8,30.7)	14.8 (11.8,17.7)	13.6 (10.9,16.3)	12.8 (10.0,15.6)	11.7 (9.0,14.5)	14.5 (7.7,17.3)	Rural
Men	Jeollanam-do	Yeongam-gun	15.0 (13.6,16.3)	24.6 (20.7,28.6)	16.5 (13.4,19.6)	12.2 (9.4,14.9)	11.6 (9.1,14.2)	10.3 (7.8,12.7)	14.3 (10.3,19.5)	Rural
Men	Jeollanam-do	Muan-gun	13.7 (12.4,15.0)	20.5 (16.8,24.1)	14.4 (11.5,17.3)	13.9 (11.1,16.7)	9.9 (7.6,12.2)	10.5 (7.9,13.1)	10.0 (6.3,15.3)	Rural
Men	Jeollanam-do	Hampyeong-gun	15.6 (14.2,17.0)	25.5 (21.3,29.7)	16.1 (13.0,19.2)	13.1 (10.3,16.0)	12.9 (10.2,15.7)	10.2 (7.8,12.6)	15.3 (9.3,18.7)	Rural

Men	Jeollanam-do	Yeonggwang-gun	15.8 (14.5,17.2)	26.3 (22.2,30.4)	18.7 (15.4,22.0)	15.0 (12.0,17.9)	11.2 (8.8,13.7)	8.3 (6.1,10.4)	18.0 (14.7,24.1)	Rural
Men	Jeollanam-do	Jangseong-gun	15.3 (14.0,16.7)	25.8 (21.6,29.9)	15.6 (12.5,18.8)	12.8 (10.1,15.4)	12.1 (9.5,14.8)	10.6 (8.1,13.1)	15.2 (7.2,17.0)	Rural
Men	Jeollanam-do	Wando-gun	15.7 (14.4,17.1)	28.8 (24.4,33.3)	16.7 (13.7,19.7)	12.0 (9.6,14.4)	12.6 (10.0,15.3)	8.9 (6.6,11.1)	19.9 (15.4,24.8)	Rural
Men	Jeollanam-do	Jindo-gun	15.4 (14.1,16.8)	26.8 (22.4,31.1)	17.4 (14.2,20.6)	13.1 (10.6,15.7)	9.9 (7.7,12.1)	9.9 (7.5,12.4)	16.9 (12.3,21.7)	Rural
Men	Jeollanam-do	Sinan-gun	16.7 (15.3,18.1)	24.4 (20.5,28.3)	18.9 (15.7,22.1)	16.3 (13.1,19.5)	12.2 (9.7,14.7)	11.0 (8.4,13.7)	13.4 (10.5,20.1)	Rural
Men	Gyeongsangbuk-do	Nam-gu	13.8 (12.4,15.2)	23.9 (19.8,27.9)	14.8 (11.7,17.9)	12.1 (9.0,15.1)	9.5 (6.7,12.2)	8.7 (6.3,11.2)	15.2 (10.9,19.5)	Metropolitan
Men	Gyeongsangbuk-do	Buk-gu	12.4 (11.1,13.7)	19.0 (15.4,22.7)	12.6 (9.8,15.4)	11.0 (8.2,13.8)	9.8 (7.2,12.4)	9.5 (6.9,12.1)	9.5 (5.9,14.3)	Metropolitan
Men	Gyeongsangbuk-do	Gyeongju-si	15.2 (13.8,16.6)	23.5 (19.6,27.5)	15.0 (11.9,18.1)	14.9 (12.0,17.9)	10.9 (8.3,13.4)	12.0 (9.2,14.8)	11.5 (5.8,15.0)	Urban
Men	Gyeongsangbuk-do	Gimcheon-si	14.8 (13.5,16.2)	22.9 (19.2,26.7)	16.0 (12.9,19.1)	14.2 (11.2,17.1)	10.1 (7.6,12.6)	10.8 (8.2,13.4)	12.1 (6.6,15.8)	Urban
Men	Gyeongsangbuk-do	Andong-si	14.2 (12.9,15.6)	23.8 (19.8,27.7)	15.2 (12.3,18.1)	13.2 (10.4,16.1)	9.9 (7.6,12.3)	9.8 (7.3,12.2)	14.0 (8.2,17.0)	Urban
Men	Gyeongsangbuk-do	Gumi-si	14.6 (13.4,15.8)	22.7 (19.3,26.1)	14.6 (12.0,17.2)	14.3 (11.6,17.0)	11.7 (9.3,14.1)	11.1 (8.9,13.4)	11.6 (6.6,13.2)	Urban
Men	Gyeongsangbuk-do	Yeongju-si	14.8 (13.4,16.1)	26.1 (21.8,30.3)	14.0 (11.2,16.9)	13.9 (11.1,16.8)	11.6 (8.9,14.2)	8.6 (6.4,10.8)	17.5 (11.7,20.9)	Urban
Men	Gyeongsangbuk-do	Yeongcheon-si	16.7 (15.2,18.1)	24.3 (20.3,28.3)	19.6 (16.1,23.1)	14.9 (12.1,17.7)	10.9 (8.4,13.4)	13.6 (10.6,16.5)	10.7 (7.2,17.0)	Urban
Men	Gyeongsangbuk-do	Sangju-si	15.2 (13.8,16.5)	23.4 (19.6,27.1)	14.9 (12.1,17.7)	13.8 (11.0,16.5)	13.3 (10.6,15.9)	10.3 (7.8,12.7)	13.1 (8.6,17.6)	Urban
Men	Gyeongsangbuk-do	Mungyeong-si	15.7 (14.4,17.1)	25.0 (21.0,28.9)	12.5 (9.8,15.2)	16.2 (13.1,19.3)	12.6 (9.9,15.3)	12.6 (9.7,15.5)	12.4 (5.8,15.8)	Urban
Men	Gyeongsangbuk-do	Gyeongsan-si	15.3 (13.9,16.7)	23.6 (19.6,27.7)	15.1 (12.1,18.2)	11.9 (9.0,14.8)	13.6 (10.6,16.7)	12.3 (9.4,15.2)	11.3 (4.1,13.3)	Urban
Men	Gyeongsangbuk-do	Gunwi-gun	14.6 (13.3,15.9)	22.2 (18.1,26.3)	16.6 (13.5,19.7)	13.1 (10.5,15.6)	11.7 (9.1,14.2)	10.1 (7.7,12.5)	12.1 (5.2,14.6)	Rural
Men	Gyeongsangbuk-do	Uiseong-gun	13.6 (12.4,14.9)	18.5 (15.0,22.0)	15.6 (12.6,18.6)	12.0 (9.5,14.5)	11.5 (8.9,14.2)	11.1 (8.4,13.7)	7.4 (3.1,12.3)	Rural
Men	Gyeongsangbuk-do	Cheongsong-gun	16.9 (15.4,18.4)	27.8 (23.2,32.4)	16.1 (13.2,19.0)	14.7 (11.8,17.6)	12.3 (9.7,14.9)	14.0 (10.8,17.3)	13.8 (9.2,19.2)	Rural
Men	Gyeongsangbuk-do	Yeongyang-gun	17.3 (15.8,18.7)	24.5 (20.6,28.4)	21.0 (17.5,24.5)	15.3 (12.4,18.3)	13.6 (10.8,16.3)	11.6 (8.9,14.3)	12.9 (11.0,20.6)	Rural
Men	Gyeongsangbuk-do	Yeongdeok-gun	16.7 (15.2,18.3)	30.3 (25.3,35.3)	20.3 (16.6,23.9)	12.9 (10.1,15.8)	10.8 (8.3,13.3)	9.7 (7.2,12.2)	20.6 (12.2,21.8)	Rural
Men	Gyeongsangbuk-do	Cheongdo-gun	16.1 (14.7,17.6)	27.9 (23.3,32.5)	14.7 (11.8,17.5)	14.8 (11.9,17.7)	13.1 (10.2,15.9)	10.7 (8.1,13.2)	17.2 (9.5,19.5)	Rural
Men	Gyeongsangbuk-do	Goryeong-gun	14.7 (13.3,16.0)	21.1 (17.4,24.8)	14.2 (11.3,17.1)	14.0 (11.1,16.9)	12.0 (9.4,14.6)	12.0 (9.3,14.8)	9.1 (3.7,13.3)	Rural
Men	Gyeongsangbuk-do	Seongju-gun	10.4 (9.3,11.5)	18.8 (15.3,22.3)	10.6 (8.2,13.1)	7.4 (5.3,9.5)	7.5 (5.5,9.5)	7.7 (5.7,9.8)	11.1 (7.8,16.0)	Rural
Men	Gyeongsangbuk-do	Chilgok-gun	14.8 (13.3,16.3)	24.2 (20.0,28.4)	15.5 (12.0,18.9)	13.6 (10.6,16.7)	9.1 (6.5,11.6)	11.7 (8.7,14.7)	12.5 (7.1,16.3)	Rural
Men	Gyeongsangbuk-do	Yecheon-gun	16.1 (14.7,17.6)	31.0 (26.2,35.8)	15.0 (12.1,18.0)	14.7 (11.7,17.6)	11.3 (8.8,13.9)	9.4 (7.0,11.8)	21.6 (12.7,22.7)	Rural
Men	Gyeongsangbuk-do	Bonghwa-gun	15.9 (14.5,17.2)	23.5 (19.7,27.4)	15.7 (12.8,18.6)	17.3 (14.0,20.5)	10.6 (8.3,12.8)	12.0 (9.2,14.8)	11.5 (5.3,14.9)	Rural
Men	Gyeongsangbuk-do	Uljin-gun	15.1 (13.8,16.5)	23.2 (19.3,27.1)	15.8 (12.7,18.9)	11.4 (9.0,13.8)	13.2 (10.3,16.0)	12.5 (9.8,15.2)	10.7 (6.1,16.1)	Rural
Men	Gyeongsangbuk-do	Ulleung-gun	13.1 (11.8,14.4)	21.7 (17.9,25.5)	14.5 (11.4,17.6)	11.3 (8.8,13.9)	9.1 (6.7,11.4)	9.4 (6.9,11.9)	12.3 (8.9,18.3)	Rural
Men	Gyeongsangnam-do	Changwon-si	13.6 (11.9,15.2)	18.6 (14.2,22.9)	15.2 (11.1,19.4)	13.4 (10.0,16.9)	11.3 (8.0,14.7)	9.5 (6.2,12.8)	9.1 (6.0,14.6)	Metropolitan
Men	Gyeongsangnam-do	Masan-si	12.6 (11.3,13.9)	19.9 (16.2,23.6)	11.3 (8.6,13.9)	11.8 (9.0,14.5)	11.6 (8.7,14.5)	8.6 (6.1,11.1)	11.3 (6.2,14.8)	Metropolitan
Men	Gyeongsangnam-do	Jinju-si	12.8 (11.5,14.1)	22.7 (18.6,26.8)	11.4 (8.7,14.2)	12.4 (9.4,15.3)	10.6 (7.9,13.3)	7.1 (4.8,9.4)	15.6 (10.1,18.9)	Urban
Men	Gyeongsangnam-do	Jinhae-si	13.1 (11.7,14.5)	21.5 (17.2,25.7)	13.3 (10.3,16.4)	13.1 (9.9,16.3)	10.4 (7.6,13.1)	8.1 (5.6,10.6)	13.4 (7.7,16.5)	Metropolitan
Men	Gyeongsangnam-do	Tongyeong-si	16.2 (14.7,17.6)	26.7 (22.4,31.1)	18.3 (15.0,21.7)	14.2 (11.1,17.4)	11.7 (8.9,14.5)	10.1 (7.5,12.8)	16.6 (11.3,20.5)	Urban
Men	Gyeongsangnam-do	Sacheon-si	15.1 (13.7,16.5)	25.8 (21.7,30.0)	15.8 (12.4,19.1)	11.0 (8.4,13.5)	12.1 (9.3,14.9)	11.4 (8.6,14.2)	14.4 (8.5,18.1)	Urban
Men	Gyeongsangnam-do	Gimhae-si	13.8 (12.2,15.3)	22.4 (17.9,27.0)	13.9 (10.6,17.2)	11.9 (8.6,15.2)	10.2 (7.4,13.1)	11.5 (8.2,14.8)	10.9 (5.6,14.6)	Urban
Men	Gyeongsangnam-do	Miryang-si	14.8 (13.4,16.1)	27.0 (22.9,31.1)	15.9 (12.8,18.9)	12.0 (9.4,14.6)	8.8 (6.6,11.1)	10.4 (7.9,12.9)	16.6 (13.6,22.8)	Urban

Men	Gyeongsangnam-do	Geoje-si	13.6 (12.1,15.2)	20.3 (16.1,24.4)	12.9 (9.5,16.3)	12.0 (8.7,15.4)	10.8 (7.8,13.7)	12.1 (8.9,15.2)	8.2 (3.9,12.9)	Urban
Men	Gyeongsangnam-do	Yangsan-si	11.4 (10.1,12.7)	17.3 (13.7,20.9)	12.2 (9.1,15.4)	9.0 (6.5,11.4)	9.6 (6.8,12.5)	9.3 (6.6,11.9)	8.0 (3.4,12.0)	Urban
Men	Gyeongsangnam-do	Uiryeong-gun	16.1 (14.6,17.6)	22.7 (18.5,27.0)	18.0 (14.7,21.3)	13.0 (10.2,15.7)	15.1 (11.8,18.3)	12.7 (9.7,15.7)	10.0 (8.7,18.9)	Rural
Men	Gyeongsangnam-do	Haman-gun	13.0 (11.7,14.2)	21.5 (17.8,25.2)	12.8 (10.1,15.6)	11.7 (9.1,14.3)	10.2 (7.8,12.6)	8.9 (6.6,11.2)	12.6 (8.4,17.6)	Rural
Men	Gyeongsangnam-do	Changnyeong-gun	15.0 (13.7,16.4)	23.0 (19.0,27.0)	16.6 (13.5,19.7)	13.5 (10.7,16.3)	11.2 (8.7,13.7)	11.0 (8.4,13.6)	12.0 (7.8,17.4)	Rural
Men	Gyeongsangnam-do	Goseong-gun	13.3 (12.1,14.6)	24.5 (20.4,28.7)	14.6 (11.5,17.6)	11.3 (8.9,13.8)	8.8 (6.4,11.1)	8.2 (5.9,10.4)	16.3 (10.6,19.6)	Rural
Men	Gyeongsangnam-do	Namhae-gun	18.6 (17.0,20.1)	28.3 (23.9,32.8)	21.6 (17.8,25.5)	15.9 (12.8,18.9)	15.5 (12.2,18.9)	11.7 (9.0,14.5)	16.6 (11.5,22.1)	Rural
Men	Gyeongsangnam-do	Hadong-gun	17.7 (16.2,19.2)	25.8 (21.5,30.0)	16.5 (13.4,19.6)	19.3 (15.9,22.8)	14.2 (11.2,17.2)	12.6 (9.7,15.4)	13.2 (5.8,16.0)	Rural
Men	Gyeongsangnam-do	Sancheong-gun	16.9 (15.4,18.3)	28.1 (23.5,32.7)	18.3 (15.0,21.5)	12.9 (10.2,15.6)	13.7 (10.9,16.6)	11.7 (8.9,14.5)	16.4 (11.7,21.9)	Rural
Men	Gyeongsangnam-do	Hamyang-gun	16.8 (15.3,18.3)	22.2 (18.3,26.1)	21.6 (17.9,25.4)	15.1 (12.0,18.2)	13.9 (10.9,16.8)	11.1 (8.6,13.7)	11.1 (10.3,20.5)	Rural
Men	Gyeongsangnam-do	Geochang-gun	15.0 (13.6,16.3)	25.1 (21.0,29.2)	16.7 (13.6,19.9)	12.7 (9.9,15.4)	11.3 (8.7,13.8)	9.5 (7.1,11.9)	15.6 (8.6,18.0)	Rural
Men	Gyeongsangnam-do	Hapcheon-gun	16.1 (14.7,17.5)	26.4 (21.9,30.9)	18.3 (14.9,21.8)	13.5 (10.6,16.3)	10.2 (7.9,12.4)	13.0 (10.0,16.1)	13.4 (7.3,16.9)	Rural
Men	Jeju-do	Jeju-si	14.2 (13.3,15.0)	23.8 (21.4,26.3)	15.3 (13.3,17.3)	12.9 (11.0,14.8)	9.9 (8.3,11.5)	9.8 (8.3,11.3)	14.0 (11.1,16.5)	Urban
Men	Jeju-do	Seogwipo-si	15.1 (14.2,15.9)	22.5 (20.1,24.9)	15.5 (13.6,17.4)	13.7 (11.9,15.4)	12.7 (11.0,14.4)	11.6 (9.9,13.2)	10.9 (6.4,12.0)	Urban
Women	Seoul	Jongno-gu	14.4 (13.2,15.6)	21.7 (18.4,25.1)	14.0 (11.4,16.6)	14.1 (11.4,16.8)	12.3 (9.9,14.8)	10.2 (8.0,12.5)	11.5 (3.8,12.2)	Metropolitan
Women	Seoul	Jung-gu	15.3 (14.1,16.6)	22.7 (19.2,26.3)	16.8 (13.9,19.8)	14.9 (12.2,17.6)	11.3 (8.9,13.7)	11.2 (8.8,13.6)	11.5 (7.4,16.0)	Metropolitan
Women	Seoul	Yongsan-gu	11.1 (10.0,12.1)	16.9 (13.8,19.9)	12.1 (9.6,14.6)	8.6 (6.5,10.6)	9.1 (7.0,11.2)	8.9 (6.8,11.1)	8.0 (4.0,11.6)	Metropolitan
Women	Seoul	Seongdong-gu	14.3 (13.1,15.6)	18.9 (15.6,22.2)	17.1 (14.0,20.2)	13.4 (10.7,16.0)	11.6 (9.0,14.1)	10.6 (8.1,13.0)	8.3 (2.8,10.6)	Metropolitan
Women	Seoul	Gwangjin-gu	14.1 (12.8,15.4)	18.9 (15.5,22.3)	14.6 (11.6,17.6)	13.2 (10.3,16.0)	13.0 (10.3,15.7)	11.1 (8.5,13.8)	7.8 (3.8,12.0)	Metropolitan
Women	Seoul	Dongdaemun-gu	16.1 (14.8,17.4)	19.9 (16.6,23.2)	17.6 (14.6,20.6)	16.5 (13.6,19.4)	15.5 (12.6,18.3)	11.3 (8.9,13.7)	8.6 (5.1,13.3)	Metropolitan
Women	Seoul	Jungnang-gu	14.4 (13.2,15.7)	20.7 (17.3,24.1)	14.9 (12.2,17.6)	13.6 (11.0,16.3)	12.1 (9.5,14.6)	11.1 (8.7,13.6)	9.6 (4.6,12.6)	Metropolitan
Women	Seoul	Seongbuk-gu	13.0 (11.9,14.2)	18.7 (15.5,21.8)	12.0 (9.5,14.5)	13.9 (11.2,16.5)	11.6 (9.1,14.0)	9.3 (7.1,11.5)	9.4 (4.2,12.0)	Metropolitan
Women	Seoul	Gangbuk-gu	15.2 (14.0,16.5)	18.8 (15.7,22.0)	18.5 (15.4,21.5)	13.3 (10.7,15.9)	13.2 (10.7,15.8)	12.4 (9.9,14.9)	6.4 (1.5,9.7)	Metropolitan
Women	Seoul	Dobong-gu	15.5 (14.2,16.7)	20.0 (16.7,23.3)	16.8 (13.9,19.7)	13.9 (11.3,16.5)	12.1 (9.6,14.6)	14.7 (11.9,17.4)	5.3 (1.2,9.6)	Metropolitan
Women	Seoul	Nowon-gu	16.1 (14.8,17.4)	25.4 (21.7,29.1)	18.9 (15.8,22.0)	13.6 (10.9,16.3)	11.6 (9.2,14.0)	11.0 (8.5,13.5)	14.4 (8.1,16.5)	Metropolitan
Women	Seoul	Eunpyeong-gu	15.9 (14.6,17.2)	22.1 (18.7,25.6)	17.5 (14.4,20.6)	15.2 (12.4,17.9)	12.2 (9.7,14.6)	12.8 (10.2,15.4)	9.3 (4.5,12.7)	Metropolitan
Women	Seoul	Seodaemun-gu	15.5 (14.2,16.8)	22.9 (19.4,26.4)	16.4 (13.5,19.4)	12.8 (10.3,15.3)	15.2 (12.4,17.9)	10.7 (8.4,13.0)	12.2 (8.2,16.4)	Metropolitan
Women	Seoul	Mapo-gu	13.8 (12.6,15.0)	19.1 (15.8,22.3)	17.5 (14.3,20.6)	13.0 (10.3,15.7)	11.0 (8.6,13.5)	8.6 (6.4,10.8)	10.5 (8.3,16.1)	Metropolitan
Women	Seoul	Yangcheon-gu	15.2 (13.9,16.5)	21.9 (18.3,25.4)	14.3 (11.4,17.2)	13.8 (11.1,16.6)	13.6 (10.8,16.3)	12.8 (10.2,15.3)	9.1 (3.1,11.3)	Metropolitan
Women	Seoul	Gangseo-gu	14.6 (13.3,15.9)	20.9 (17.4,24.4)	17.4 (14.3,20.6)	14.0 (11.2,16.7)	10.3 (7.9,12.7)	10.9 (8.5,13.4)	10.0 (6.9,15.1)	Metropolitan
Women	Seoul	Guro-gu	16.0 (14.6,17.3)	22.1 (18.4,25.8)	16.5 (13.5,19.6)	16.4 (13.5,19.4)	12.6 (9.9,15.2)	13.0 (10.2,15.7)	9.1 (4.3,12.9)	Metropolitan
Women	Seoul	Geumcheon-gu	16.5 (15.2,17.8)	23.2 (19.6,26.8)	18.5 (15.3,21.7)	15.2 (12.4,18.0)	13.3 (10.6,16.0)	12.6 (10.0,15.3)	10.6 (3.8,12.4)	Metropolitan
Women	Seoul	Yeongdeungpo-gu	15.4 (14.1,16.7)	22.4 (18.8,26.0)	17.0 (13.9,20.0)	13.8 (11.1,16.4)	10.9 (8.5,13.4)	13.0 (10.3,15.8)	9.4 (6.1,14.7)	Metropolitan
Women	Seoul	Dongjak-gu	16.3 (14.9,17.6)	20.1 (16.8,23.5)	17.9 (14.8,21.1)	14.7 (11.8,17.6)	16.8 (13.8,19.7)	12.0 (9.4,14.5)	8.1 (3.3,11.7)	Metropolitan
Women	Seoul	Gwanak-gu	16.1 (14.7,17.5)	20.6 (17.0,24.3)	17.0 (13.8,20.2)	16.9 (13.7,20.0)	13.8 (10.9,16.7)	12.5 (9.8,15.2)	8.1 (3.5,11.7)	Metropolitan
Women	Seoul	Seocho-gu	10.8 (9.7,12.0)	14.4 (11.4,17.4)	13.6 (10.7,16.5)	10.1 (7.7,12.6)	8.4 (6.2,10.5)	8.2 (5.9,10.5)	6.2 (1.9,9.3)	Metropolitan
Women	Seoul	Gangnam-gu	11.1 (9.9,12.3)	16.9 (13.6,20.2)	12.9 (10.1,15.7)	9.6 (7.1,12.0)	9.1 (6.8,11.3)	7.4 (5.3,9.5)	9.5 (6.2,13.6)	Metropolitan

Women	Seoul	Songpa-gu	11.8 (10.6,13.0)	13.5 (10.5,16.4)	10.7 (8.1,13.2)	12.4 (9.7,15.1)	11.4 (8.9,13.9)	11.0 (8.4,13.7)	2.5 (0.9,8.3)	Metropolitan
Women	Seoul	Gangdong-gu	14.5 (13.2,15.8)	17.8 (14.5,21.2)	17.8 (14.6,21.0)	13.7 (10.9,16.4)	12.2 (9.5,15.0)	11.0 (8.4,13.7)	6.8 (3.1,11.1)	Metropolitan
Women	Busan	Jung-gu	16.5 (15.3,17.8)	21.2 (17.9,24.4)	19.1 (16.2,22.0)	16.9 (14.0,19.7)	14.2 (11.7,16.8)	11.2 (9.0,13.5)	10.0 (4.3,12.7)	Metropolitan
Women	Busan	Seo-gu	16.0 (14.8,17.3)	24.7 (21.2,28.3)	15.0 (12.4,17.7)	16.1 (13.4,18.8)	13.8 (11.4,16.2)	11.0 (8.7,13.2)	13.7 (7.4,16.2)	Metropolitan
Women	Busan	Dong-gu	20.4 (19.0,21.8)	31.3 (27.3,35.3)	20.9 (17.8,24.1)	18.9 (16.1,21.8)	16.5 (13.8,19.2)	14.7 (12.1,17.3)	16.6 (10.8,20.0)	Metropolitan
Women	Busan	Yeongdo-gu	19.1 (17.8,20.4)	27.9 (24.1,31.6)	19.8 (16.8,22.8)	18.2 (15.4,21.0)	15.9 (13.2,18.6)	14.2 (11.8,16.6)	13.7 (8.4,17.8)	Metropolitan
Women	Busan	Busanjin-gu	17.5 (16.2,18.8)	25.4 (21.9,28.9)	18.1 (15.1,21.2)	15.5 (12.8,18.3)	14.7 (12.1,17.3)	13.9 (11.3,16.5)	11.5 (8.4,17.2)	Metropolitan
Women	Busan	Dongnae-gu	15.2 (14.0,16.4)	21.9 (18.5,25.2)	16.0 (13.2,18.8)	13.4 (10.8,15.9)	13.9 (11.3,16.5)	10.6 (8.2,13.0)	11.3 (6.6,14.8)	Metropolitan
Women	Busan	Nam-gu	16.8 (15.5,18.1)	22.1 (18.6,25.6)	18.7 (15.7,21.7)	17.1 (14.2,20.0)	15.0 (12.3,17.7)	11.8 (9.3,14.2)	10.3 (5.9,14.5)	Metropolitan
Women	Busan	Buk-gu	17.0 (15.6,18.4)	25.8 (21.9,29.8)	17.8 (14.6,20.9)	17.8 (14.6,21.0)	11.9 (9.4,14.5)	12.0 (9.4,14.7)	13.8 (9.9,18.5)	Metropolitan
Women	Busan	Haeundae-gu	16.7 (15.4,18.0)	28.3 (24.4,32.1)	16.9 (13.9,19.9)	13.9 (11.3,16.5)	14.9 (12.1,17.7)	9.9 (7.6,12.1)	18.4 (14.8,23.6)	Metropolitan
Women	Busan	Saha-gu	15.8 (14.5,17.0)	23.4 (19.7,27.1)	16.1 (13.2,19.0)	14.4 (11.7,17.2)	12.7 (10.1,15.3)	12.6 (10.1,15.2)	10.8 (7.0,15.8)	Metropolitan
Women	Busan	Geumjeong-gu	15.0 (13.8,16.2)	22.5 (19.0,26.0)	16.7 (13.9,19.6)	15.9 (12.9,18.8)	12.7 (10.3,15.2)	8.0 (6.1,10.0)	14.5 (8.7,16.9)	Metropolitan
Women	Busan	Gangseo-gu	18.8 (17.5,20.2)	24.3 (20.7,27.8)	22.0 (18.7,25.2)	16.1 (13.2,18.9)	15.8 (13.0,18.5)	16.1 (13.3,18.9)	8.2 (4.8,13.8)	Metropolitan
Women	Busan	Yeonje-gu	17.0 (15.6,18.3)	26.7 (23.0,30.4)	17.7 (14.6,20.7)	14.2 (11.5,16.9)	13.1 (10.6,15.7)	13.2 (10.5,15.8)	13.5 (9.1,17.9)	Metropolitan
Women	Busan	Suyeong-gu	15.7 (14.5,17.0)	21.7 (18.4,25.0)	17.8 (14.9,20.7)	13.7 (11.1,16.3)	12.9 (10.5,15.3)	12.5 (10.1,15.0)	9.2 (4.3,12.9)	Metropolitan
Women	Busan	Sasang-gu	18.7 (17.2,20.2)	25.4 (21.4,29.4)	20.9 (17.5,24.4)	17.2 (14.1,20.3)	15.3 (12.4,18.3)	15.2 (12.1,18.2)	10.2 (5.4,14.6)	Metropolitan
Women	Busan	Gijang-gun	19.9 (18.5,21.2)	25.1 (21.6,28.7)	23.9 (20.5,27.3)	18.8 (15.8,21.8)	16.6 (13.8,19.4)	14.8 (12.1,17.5)	10.3 (6.7,15.9)	Rural
Women	Daegu	Jung-gu	17.7 (16.4,19.0)	26.5 (22.7,30.2)	17.9 (15.0,20.7)	15.7 (13.0,18.4)	14.7 (12.0,17.3)	14.2 (11.5,16.8)	12.3 (8.5,17.7)	Metropolitan
Women	Daegu	Dong-gu	19.6 (18.2,21.0)	27.2 (23.5,31.0)	18.1 (15.1,21.2)	20.7 (17.5,23.8)	18.0 (15.0,21.0)	14.4 (11.8,17.1)	12.8 (8.3,17.3)	Metropolitan
Women	Daegu	Seo-gu	20.4 (19.0,21.9)	28.6 (24.6,32.5)	19.7 (16.5,22.8)	18.3 (15.3,21.4)	19.9 (16.7,23.1)	16.0 (13.1,18.8)	12.6 (2.3,11.9)	Metropolitan
Women	Daegu	Nam-gu	17.8 (16.5,19.1)	24.0 (20.5,27.5)	20.5 (17.3,23.7)	17.4 (14.6,20.2)	14.9 (12.2,17.7)	12.6 (10.1,15.0)	11.4 (8.8,17.4)	Metropolitan
Women	Daegu	Buk-gu	16.3 (14.9,17.6)	23.5 (19.7,27.2)	16.9 (13.8,20.0)	17.4 (14.2,20.6)	12.4 (9.8,15.1)	11.4 (8.8,13.9)	12.1 (8.9,17.7)	Metropolitan
Women	Daegu	Suseong-gu	17.8 (16.4,19.2)	27.7 (23.7,31.7)	19.1 (15.9,22.3)	17.2 (14.2,20.2)	13.3 (10.6,15.9)	12.4 (9.8,15.0)	15.3 (10.0,19.0)	Metropolitan
Women	Daegu	Dalseo-gu	17.9 (16.5,19.4)	24.6 (20.8,28.5)	19.7 (16.3,23.2)	16.9 (13.7,20.1)	15.3 (12.3,18.2)	13.5 (10.6,16.3)	11.1 (7.7,16.5)	Metropolitan
Women	Daegu	Dalseong-gun	19.5 (18.0,20.9)	27.5 (23.5,31.5)	19.7 (16.5,23.0)	17.1 (14.1,20.1)	14.7 (11.9,17.5)	18.6 (15.4,21.7)	8.9 (3.9,13.5)	Rural
Women	Incheon	Jung-gu	16.5 (15.2,17.8)	23.4 (19.8,27.0)	15.3 (12.5,18.1)	16.5 (13.6,19.4)	13.3 (10.8,15.9)	14.2 (11.5,17.0)	9.2 (4.8,13.8)	Metropolitan
Women	Incheon	Dong-gu	15.5 (14.2,16.7)	20.8 (17.5,24.1)	16.2 (13.4,19.0)	14.9 (12.2,17.6)	13.1 (10.7,15.6)	12.6 (10.1,15.1)	8.2 (3.2,11.8)	Metropolitan
Women	Incheon	Nam-gu	14.1 (12.9,15.3)	20.0 (16.6,23.4)	14.9 (12.1,17.8)	13.1 (10.5,15.8)	12.2 (9.7,14.6)	10.7 (8.3,13.0)	9.3 (5.8,14.0)	Metropolitan
Women	Incheon	Yeonsu-gu	15.6 (14.2,17.0)	24.7 (20.7,28.7)	16.0 (12.8,19.2)	14.6 (11.6,17.6)	13.0 (10.2,15.8)	10.0 (7.5,12.5)	14.7 (9.7,17.9)	Metropolitan
Women	Incheon	Namdong-gu	16.8 (15.4,18.2)	23.3 (19.5,27.0)	17.5 (14.3,20.7)	17.3 (14.2,20.4)	12.4 (9.8,15.1)	13.3 (10.5,16.0)	10.0 (5.3,13.7)	Metropolitan
Women	Incheon	Bupyeong-gu	17.0 (15.6,18.4)	23.9 (20.0,27.7)	17.9 (14.7,21.1)	16.8 (13.7,20.0)	13.9 (11.2,16.5)	13.3 (10.6,16.1)	10.6 (6.3,14.9)	Metropolitan
Women	Incheon	Gyeyang-gu	16.8 (15.4,18.3)	23.3 (19.3,27.3)	19.4 (15.9,22.8)	14.1 (11.1,17.0)	14.1 (11.2,17.0)	13.9 (10.9,16.9)	9.4 (4.5,13.1)	Metropolitan
Women	Incheon	Seo-gu	18.8 (17.3,20.4)	24.1 (20.0,28.1)	19.9 (16.3,23.5)	18.3 (14.8,21.9)	16.7 (13.5,19.9)	15.3 (12.0,18.5)	8.8 (2.4,12.0)	Metropolitan
Women	Incheon	Ganghwa-gun	18.4 (17.1,19.8)	23.3 (19.7,26.9)	19.1 (16.0,22.2)	19.1 (16.1,22.2)	15.2 (12.6,17.8)	15.6 (12.8,18.5)	7.7 (2.6,11.6)	Rural
Women	Incheon	Ongjin-gun	13.8 (12.6,15.0)	16.5 (13.6,19.4)	15.1 (12.4,17.9)	12.0 (9.6,14.5)	12.1 (9.8,14.4)	13.3 (10.6,16.0)	3.2 (0.8,9.8)	Rural
Women	Gwangju	Dong-gu	16.4 (15.1,17.6)	24.9 (21.2,28.5)	17.7 (14.8,20.6)	16.4 (13.6,19.2)	12.5 (10.2,14.8)	11.5 (9.2,13.9)	13.4 (9.4,18.0)	Metropolitan

Women	Gwangju	Seo-gu	15.9 (14.6,17.3)	23.1 (19.4,26.8)	17.5 (14.3,20.6)	12.3 (9.6,15.0)	14.9 (12.0,17.8)	12.4 (9.8,15.1)	10.7 (4.8,13.6)	Metropolitan
Women	Gwangju	Nam-gu	17.1 (15.8,18.5)	24.4 (20.7,28.1)	17.6 (14.5,20.7)	17.2 (14.4,20.0)	14.4 (11.7,17.1)	12.3 (9.9,14.7)	12.1 (8.4,17.0)	Metropolitan
Women	Gwangju	Buk-gu	17.3 (15.9,18.7)	26.1 (22.3,29.9)	16.4 (13.4,19.4)	18.2 (15.1,21.4)	14.7 (11.8,17.5)	11.6 (9.0,14.1)	14.5 (6.9,15.3)	Metropolitan
Women	Gwangju	Gwangsan-gu	17.9 (16.4,19.5)	26.5 (22.2,30.9)	18.8 (15.4,22.2)	17.1 (13.8,20.5)	14.5 (11.4,17.5)	13.2 (10.3,16.2)	13.3 (5.6,14.6)	Metropolitan
Women	Daejeon	Dong-gu	16.4 (15.1,17.7)	25.3 (21.5,29.2)	18.9 (15.8,21.9)	13.9 (11.3,16.6)	13.9 (11.3,16.5)	10.9 (8.6,13.3)	14.4 (9.4,18.0)	Metropolitan
Women	Daejeon	Jung-gu	14.7 (13.4,15.9)	22.2 (18.7,25.8)	14.1 (11.5,16.7)	14.6 (11.8,17.3)	13.0 (10.4,15.5)	10.4 (8.1,12.6)	11.8 (6.9,15.3)	Metropolitan
Women	Daejeon	Seo-gu	16.0 (14.6,17.4)	23.2 (19.2,27.1)	16.3 (13.2,19.5)	14.2 (11.2,17.2)	14.3 (11.3,17.2)	12.3 (9.5,15.2)	10.9 (8.6,17.2)	Metropolitan
Women	Daejeon	Yuseong-gu	13.7 (12.4,15.1)	18.3 (14.7,21.9)	16.9 (13.4,20.3)	10.9 (8.2,13.5)	13.0 (10.1,16.0)	9.4 (6.7,12.1)	8.9 (5.6,13.8)	Metropolitan
Women	Daejeon	Daedeok-gu	16.2 (14.8,17.5)	21.4 (17.8,25.0)	17.5 (14.4,20.6)	14.2 (11.3,17.0)	14.5 (11.6,17.4)	13.0 (10.2,15.8)	8.4 (3.5,11.9)	Metropolitan
Women	Ulsan	Jung-gu	17.1 (15.6,18.5)	22.9 (19.2,26.7)	17.3 (14.1,20.4)	15.6 (12.6,18.7)	14.7 (11.9,17.6)	15.1 (12.0,18.2)	7.8 (2.6,11.4)	Metropolitan
Women	Ulsan	Nam-gu	15.1 (13.7,16.6)	21.2 (17.0,25.4)	15.6 (12.4,18.8)	13.4 (10.2,16.5)	13.3 (10.4,16.2)	13.0 (9.8,16.2)	8.2 (5.2,13.6)	Metropolitan
Women	Ulsan	Dong-gu	14.6 (13.1,16.0)	21.2 (17.1,25.3)	17.4 (13.7,21.0)	13.1 (10.0,16.2)	10.9 (8.1,13.7)	10.4 (7.5,13.3)	10.8 (6.8,15.2)	Metropolitan
Women	Ulsan	Buk-gu	13.8 (12.4,15.3)	16.8 (13.0,20.6)	16.0 (12.6,19.4)	15.4 (12.0,18.8)	12.1 (9.1,15.1)	8.8 (6.2,11.4)	8.0 (5.5,13.3)	Metropolitan
Women	Ulsan	Ulju-gun	19.1 (17.7,20.5)	26.3 (22.4,30.1)	20.4 (17.1,23.6)	17.4 (14.4,20.5)	16.0 (13.2,18.8)	16.1 (13.2,19.0)	10.2 (7.0,16.2)	Rural
Women	Sejong	Sejong	18.3 (16.9,19.6)	23.5 (20.0,27.0)	20.7 (17.5,23.9)	18.1 (15.1,21.0)	15.4 (12.7,18.0)	14.1 (11.5,16.6)	9.4 (5.1,13.9)	Metropolitan
Women	Gyeonggi-do	Jangan-gu	15.6 (14.3,17.0)	18.6 (15.3,21.9)	18.5 (15.2,21.7)	14.0 (11.1,16.9)	14.7 (11.8,17.6)	12.0 (9.3,14.7)	6.6 (2.5,10.9)	Metropolitan
Women	Gyeonggi-do	Gwonseon-gu	16.4 (15.0,17.9)	22.5 (18.6,26.4)	17.5 (14.1,20.8)	15.1 (12.0,18.1)	14.0 (11.0,17.0)	13.3 (10.3,16.3)	9.2 (3.0,12.0)	Metropolitan
Women	Gyeonggi-do	Paldal-gu	16.4 (15.0,17.8)	23.7 (19.9,27.5)	17.7 (14.4,20.9)	14.4 (11.6,17.2)	13.4 (10.7,16.2)	13.4 (10.5,16.3)	10.3 (7.0,16.0)	Metropolitan
Women	Gyeonggi-do	Yeongtong-gu	12.5 (11.0,13.9)	17.3 (13.4,21.3)	11.0 (8.1,13.9)	10.4 (7.4,13.4)	12.5 (9.1,15.8)	11.5 (8.3,14.6)	5.8 (1.0,9.4)	Metropolitan
Women	Gyeonggi-do	Sujeong-gu	17.2 (15.8,18.6)	23.3 (19.6,27.1)	18.7 (15.4,21.9)	15.1 (12.1,18.0)	16.2 (13.2,19.2)	13.0 (10.1,15.8)	10.3 (7.2,16.2)	Metropolitan
Women	Gyeonggi-do	Jungwon-gu	18.9 (17.4,20.4)	24.8 (20.9,28.7)	20.6 (17.2,24.1)	17.1 (13.9,20.3)	16.3 (13.3,19.4)	16.1 (12.9,19.2)	8.7 (5.8,15.2)	Metropolitan
Women	Gyeonggi-do	Bundang-gu	9.6 (8.5,10.6)	12.6 (9.8,15.3)	10.7 (8.2,13.3)	6.9 (4.9,8.9)	9.1 (6.8,11.4)	8.5 (6.2,10.8)	4.1 (0.6,7.4)	Metropolitan
Women	Gyeonggi-do	Uijeongbu-si	16.8 (15.5,18.1)	25.5 (21.7,29.3)	19.9 (16.6,23.1)	14.0 (11.3,16.6)	13.3 (10.7,15.9)	12.3 (9.8,14.8)	13.2 (8.8,17.4)	Urban
Women	Gyeonggi-do	Manan-gu	15.8 (14.4,17.1)	21.6 (18.0,25.2)	15.8 (12.8,18.8)	14.4 (11.6,17.1)	13.9 (11.2,16.7)	13.6 (10.9,16.3)	8.0 (5.4,14.0)	Metropolitan
Women	Gyeonggi-do	Dongan-gu	13.6 (12.3,14.9)	17.4 (14.1,20.7)	14.7 (11.6,17.7)	13.4 (10.6,16.1)	11.2 (8.6,13.8)	11.4 (8.8,14.1)	6.0 (1.2,9.2)	Metropolitan
Women	Gyeonggi-do	Wonmi-gu	16.3 (14.8,17.7)	22.4 (18.5,26.3)	18.2 (14.6,21.9)	14.9 (12.0,17.9)	13.8 (10.8,16.8)	12.6 (9.7,15.4)	9.8 (4.0,12.6)	Metropolitan
Women	Gyeonggi-do	Sosa-gu	16.0 (14.6,17.3)	21.6 (17.9,25.3)	16.4 (13.2,19.5)	15.7 (12.7,18.7)	14.3 (11.5,17.2)	12.1 (9.5,14.7)	9.5 (5.9,14.7)	Metropolitan
Women	Gyeonggi-do	Ojeong-gu	16.9 (15.5,18.3)	25.0 (21.0,28.9)	18.4 (15.2,21.6)	12.9 (10.2,15.6)	13.0 (10.3,15.8)	15.4 (12.4,18.5)	9.6 (5.0,14.0)	Metropolitan
Women	Gyeonggi-do	Gwangmyeong-si	15.8 (14.4,17.2)	22.0 (18.2,25.7)	17.8 (14.7,21.0)	12.5 (9.8,15.3)	11.9 (9.2,14.5)	15.1 (12.1,18.1)	6.9 (5.0,13.8)	Urban
Women	Gyeonggi-do	Pyeongtaek-si	17.0 (16.0,18.0)	24.2 (21.6,26.8)	16.9 (14.8,19.1)	15.9 (13.8,18.0)	14.7 (12.7,16.7)	13.4 (11.6,15.3)	10.8 (8.2,14.6)	Urban
Women	Gyeonggi-do	Dongducheon-si	18.7 (17.3,20.1)	27.1 (23.3,31.0)	19.2 (16.0,22.4)	16.9 (13.9,19.8)	17.2 (14.3,20.2)	13.4 (10.8,16.0)	13.7 (8.4,17.6)	Urban
Women	Gyeonggi-do	Sangnok-gu	15.7 (14.2,17.1)	21.3 (17.6,25.1)	17.6 (14.2,21.1)	15.0 (11.9,18.0)	12.6 (9.8,15.4)	12.0 (9.2,14.8)	9.3 (7.4,15.8)	Metropolitan
Women	Gyeonggi-do	Danwon-gu	17.9 (16.3,19.4)	26.3 (21.9,30.7)	17.5 (14.1,21.0)	16.8 (13.5,20.2)	14.7 (11.5,18.0)	14.4 (11.2,17.5)	11.9 (7.9,17.1)	Metropolitan
Women	Gyeonggi-do	Deogyang-gu	17.2 (15.9,18.6)	24.4 (20.7,28.2)	18.9 (15.8,22.0)	15.9 (12.9,18.9)	13.9 (11.2,16.6)	13.0 (10.3,15.7)	11.4 (4.7,13.3)	Metropolitan
Women	Gyeonggi-do	Ilsandong-gu	14.8 (13.4,16.1)	19.2 (15.7,22.6)	15.8 (12.7,18.8)	14.8 (11.9,17.7)	13.4 (10.7,16.1)	10.8 (8.3,13.4)	8.4 (3.6,11.6)	Metropolitan
Women	Gyeonggi-do	Ilsanseo-gu	13.4 (12.2,14.7)	19.3 (15.9,22.8)	13.2 (10.4,16.0)	10.5 (8.0,13.1)	11.0 (8.5,13.4)	12.9 (10.1,15.7)	6.4 (-0.1,8.1)	Metropolitan
Women	Gyeonggi-do	Gwacheon-si	14.2 (13.0,15.5)	19.5 (16.1,22.9)	14.1 (11.3,16.9)	13.6 (10.9,16.3)	12.3 (9.7,15.0)	11.9 (9.3,14.5)	7.6 (2.8,11.2)	Urban

Women	Gyeonggi-do	Guri-si	16.9 (15.5,18.3)	22.6 (19.0,26.3)	16.8 (13.6,19.9)	17.1 (13.9,20.3)	15.9 (12.8,18.9)	11.8 (9.1,14.5)	10.8 (3.7,12.5)	Urban
Women	Gyeonggi-do	Namyangju-si	16.0 (14.7,17.4)	20.0 (16.6,23.3)	17.6 (14.4,20.7)	14.5 (11.8,17.3)	13.4 (10.7,16.1)	15.3 (12.3,18.2)	4.7 (2.1,10.7)	Urban
Women	Gyeonggi-do	Osan-si	15.8 (14.3,17.3)	18.8 (15.1,22.5)	15.5 (12.0,19.0)	15.4 (12.2,18.5)	15.8 (12.4,19.1)	13.8 (10.5,17.0)	5.0 (-0.3,8.5)	Urban
Women	Gyeonggi-do	Siheung-si	17.4 (15.8,18.9)	20.6 (16.8,24.5)	18.9 (15.5,22.4)	17.1 (13.8,20.4)	17.4 (14.0,20.8)	12.4 (9.4,15.3)	8.2 (2.3,11.1)	Urban
Women	Gyeonggi-do	Gunpo-si	16.5 (15.1,17.9)	24.7 (20.8,28.6)	15.4 (12.4,18.5)	14.1 (11.3,17.0)	14.5 (11.6,17.4)	13.7 (10.7,16.6)	11.0 (6.7,15.5)	Urban
Women	Gyeonggi-do	Uiwang-si	15.0 (13.6,16.3)	21.1 (17.5,24.8)	16.0 (12.9,19.1)	12.6 (9.9,15.3)	12.5 (9.8,15.2)	12.8 (10.1,15.6)	8.3 (4.3,12.9)	Urban
Women	Gyeonggi-do	Hanam-si	16.0 (14.7,17.4)	19.6 (16.2,23.0)	17.5 (14.4,20.7)	15.4 (12.5,18.3)	13.8 (11.1,16.4)	14.4 (11.6,17.2)	5.2 (3.0,11.6)	Urban
Women	Gyeonggi-do	Cheoin-gu	15.5 (14.2,16.8)	21.3 (17.8,24.8)	15.0 (12.2,17.9)	16.8 (13.7,19.9)	13.1 (10.5,15.8)	11.4 (8.9,14.0)	9.9 (1.6,10.0)	Metropolitan
Women	Gyeonggi-do	Giheung-gu	13.1 (11.8,14.3)	18.0 (14.7,21.4)	12.9 (10.1,15.7)	12.4 (9.6,15.1)	11.7 (9.0,14.4)	10.3 (7.8,12.9)	7.7 (4.0,11.8)	Metropolitan
Women	Gyeonggi-do	Suji-gu	11.6 (10.4,12.7)	13.5 (10.5,16.4)	10.5 (8.0,12.9)	13.0 (10.1,15.8)	9.9 (7.4,12.4)	11.6 (9.0,14.2)	1.9 (-2.2,5.2)	Metropolitan
Women	Gyeonggi-do	Paju-si	16.5 (15.1,17.8)	23.5 (19.8,27.2)	17.1 (14.1,20.2)	15.2 (12.4,18.0)	12.1 (9.6,14.6)	14.9 (12.1,17.7)	8.6 (3.2,12.0)	Urban
Women	Gyeonggi-do	Icheon-si	17.6 (16.2,19.0)	25.0 (21.2,28.8)	19.4 (16.2,22.7)	14.3 (11.5,17.1)	15.3 (12.4,18.3)	13.9 (11.1,16.7)	11.1 (6.4,15.4)	Urban
Women	Gyeonggi-do	Anseong-si	17.5 (16.1,18.8)	22.9 (19.5,26.3)	17.9 (14.9,20.9)	16.0 (13.2,18.9)	16.1 (13.3,19.0)	14.4 (11.7,17.2)	8.5 (3.8,12.4)	Urban
Women	Gyeonggi-do	Gimpo-si	13.1 (11.9,14.3)	15.6 (12.6,18.7)	14.3 (11.4,17.1)	12.2 (9.6,14.8)	12.5 (9.9,15.1)	11.2 (8.7,13.7)	4.4 (-0.5,7.3)	Urban
Women	Gyeonggi-do	Hwaseongsi	15.3 (13.9,16.7)	21.3 (17.7,24.9)	16.3 (13.1,19.4)	14.6 (11.6,17.6)	14.8 (11.9,17.8)	9.5 (7.1,12.0)	11.8 (8.1,16.3)	Urban
Women	Gyeonggi-do	Gwangju-si	14.2 (12.9,15.5)	18.5 (15.2,21.9)	15.6 (12.7,18.6)	13.6 (10.8,16.4)	11.0 (8.5,13.4)	12.2 (9.5,14.9)	6.3 (2.3,10.7)	Urban
Women	Gyeonggi-do	Yangju-si	17.4 (16.0,18.8)	22.6 (18.9,26.3)	19.1 (15.8,22.3)	15.6 (12.7,18.4)	16.6 (13.7,19.4)	13.7 (10.9,16.5)	8.9 (5.3,14.3)	Urban
Women	Gyeonggi-do	Pocheon-si	18.6 (17.2,19.9)	24.5 (20.9,28.1)	21.3 (18.0,24.7)	16.5 (13.6,19.4)	16.8 (14.0,19.6)	13.8 (11.2,16.5)	10.7 (4.7,13.7)	Urban
Women	Gyeonggi-do	Yeoju-gun	18.8 (17.5,20.2)	22.4 (19.0,25.9)	22.0 (18.7,25.2)	17.8 (14.9,20.8)	16.0 (13.2,18.8)	15.9 (13.1,18.8)	6.5 (1.6,10.4)	Urban
Women	Gyeonggi-do	Yeoncheon-gun	17.4 (16.1,18.7)	23.0 (19.6,26.5)	18.0 (15.1,20.8)	18.3 (15.4,21.3)	16.0 (13.3,18.7)	11.7 (9.5,14.0)	11.3 (7.7,16.5)	Rural
Women	Gyeonggi-do	Gapyeong-gun	16.7 (15.5,18.0)	24.1 (20.6,27.6)	19.0 (16.0,22.0)	15.3 (12.6,18.0)	12.6 (10.2,14.9)	12.4 (10.0,14.9)	11.7 (8.4,17.2)	Rural
Women	Gyeonggi-do	Yangpyeong-gun	18.2 (16.8,19.5)	21.6 (18.3,24.9)	20.2 (17.1,23.3)	18.0 (15.1,21.0)	16.9 (14.1,19.8)	13.9 (11.3,16.5)	7.7 (2.7,11.5)	Rural
Women	Gangwon-do	Chuncheon-si	15.5 (14.2,16.7)	22.5 (19.0,26.0)	17.5 (14.6,20.5)	13.8 (11.2,16.4)	13.4 (10.9,15.9)	10.7 (8.4,13.0)	11.8 (7.9,16.3)	Urban
Women	Gangwon-do	Wonju-si	17.2 (15.8,18.6)	24.0 (20.3,27.8)	21.2 (17.8,24.5)	13.9 (11.2,16.6)	14.9 (12.1,17.8)	12.2 (9.6,14.8)	11.8 (7.0,16.2)	Urban
Women	Gangwon-do	Gangneung-si	16.5 (15.3,17.8)	22.9 (19.5,26.4)	18.5 (15.6,21.4)	14.3 (11.7,16.9)	14.2 (11.6,16.7)	13.3 (10.6,15.9)	9.6 (6.3,15.1)	Urban
Women	Gangwon-do	Donghae-si	17.5 (16.2,18.8)	24.4 (20.8,28.1)	19.4 (16.4,22.3)	16.5 (13.6,19.4)	14.6 (12.0,17.2)	12.7 (10.2,15.2)	11.7 (8.5,17.3)	Urban
Women	Gangwon-do	Taebaek-si	21.8 (20.4,23.3)	30.2 (26.2,34.1)	23.9 (20.4,27.4)	21.6 (18.5,24.8)	17.3 (14.4,20.2)	16.2 (13.4,19.0)	14.0 (10.4,20.2)	Urban
Women	Gangwon-do	Sokcho-si	19.1 (17.7,20.5)	27.1 (23.4,30.8)	19.3 (16.2,22.3)	16.5 (13.7,19.2)	15.5 (12.8,18.2)	17.4 (14.5,20.4)	9.7 (4.8,14.0)	Urban
Women	Gangwon-do	Samcheok-si	19.2 (17.8,20.6)	28.5 (24.5,32.4)	19.5 (16.5,22.6)	19.9 (16.8,23.0)	15.1 (12.4,17.8)	13.6 (11.0,16.2)	14.9 (9.8,19.2)	Urban
Women	Gangwon-do	Hongcheon-gun	17.3 (16.0,18.6)	24.5 (20.8,28.2)	19.9 (16.9,23.0)	15.2 (12.7,17.8)	13.2 (10.7,15.7)	14.4 (11.7,17.0)	10.1 (5.4,14.8)	Rural
Women	Gangwon-do	Hoengseong-gun	20.1 (18.6,21.6)	27.2 (23.2,31.2)	21.5 (18.3,24.8)	19.7 (16.5,22.8)	17.7 (14.6,20.8)	14.6 (11.8,17.4)	12.6 (7.4,16.8)	Rural
Women	Gangwon-do	Yeongwol-gun	21.6 (20.1,23.0)	30.9 (26.6,35.2)	23.9 (20.6,27.2)	18.4 (15.5,21.2)	18.4 (15.5,21.3)	16.6 (13.8,19.3)	14.3 (9.0,19.2)	Rural
Women	Gangwon-do	Pyeongchang-gun	17.2 (15.9,18.4)	24.1 (20.6,27.6)	18.1 (15.4,20.9)	14.8 (12.2,17.3)	16.5 (13.7,19.3)	12.5 (10.2,14.9)	11.6 (5.8,15.0)	Rural
Women	Gangwon-do	Jeongseon-gun	21.5 (20.0,22.9)	28.7 (24.8,32.6)	21.7 (18.6,24.8)	21.8 (18.5,25.1)	20.6 (17.5,23.7)	14.8 (12.2,17.4)	13.9 (9.3,18.9)	Rural
Women	Gangwon-do	Cheorwon-gun	16.8 (15.5,18.1)	23.8 (20.3,27.4)	17.9 (15.0,20.7)	16.5 (13.7,19.4)	13.5 (11.0,16.1)	12.2 (9.7,14.7)	11.6 (9.5,18.5)	Rural
Women	Gangwon-do	Hwacheon-gun	16.7 (15.4,18.0)	22.7 (19.0,26.3)	18.3 (15.3,21.3)	15.1 (12.4,17.7)	13.9 (11.2,16.5)	13.7 (11.1,16.3)	9.0 (7.1,16.3)	Rural
Women	Gangwon-do	Yanggu-gun	17.7 (16.4,19.0)	23.2 (19.6,26.7)	19.2 (16.1,22.4)	15.8 (13.2,18.4)	14.4 (11.8,17.0)	16.3 (13.5,19.1)	6.9 (2.4,11.6)	Rural

Women	Gangwon-do	Inje-gun	17.8 (16.4,19.1)	26.3 (22.5,30.1)	17.2 (14.2,20.2)	15.7 (12.9,18.4)	16.1 (13.2,19.0)	13.5 (10.8,16.1)	12.8 (8.3,17.5)	Rural
Women	Gangwon-do	Goseong-gun	16.7 (15.4,18.0)	22.1 (18.5,25.7)	17.1 (14.2,20.0)	16.1 (13.3,18.9)	15.8 (12.9,18.6)	12.6 (10.0,15.1)	9.5 (5.2,13.8)	Rural
Women	Gangwon-do	Yangyang-gun	16.2 (15.0,17.5)	22.5 (19.1,25.9)	17.8 (14.9,20.6)	15.8 (13.1,18.5)	14.7 (12.1,17.3)	10.6 (8.4,12.7)	11.9 (7.8,16.4)	Rural
Women	Chungcheongbuk-do	Cheongju-si	15.9 (15.1,16.6)	23.4 (21.2,25.5)	17.8 (15.9,19.7)	14.1 (12.5,15.7)	12.9 (11.4,14.4)	12.0 (10.5,13.4)	11.4 (8.2,13.2)	Metropolitan
Women	Chungcheongbuk-do	Chungju-si	19.3 (17.9,20.7)	26.1 (22.5,29.8)	22.2 (18.9,25.4)	18.8 (15.7,21.9)	15.0 (12.3,17.7)	14.8 (12.1,17.6)	11.3 (7.0,16.0)	Urban
Women	Chungcheongbuk-do	Jecheon-si	17.1 (15.8,18.4)	23.5 (20.0,27.0)	17.3 (14.5,20.2)	17.5 (14.6,20.3)	14.6 (12.0,17.2)	13.0 (10.4,15.6)	10.5 (6.8,15.8)	Urban
Women	Chungcheongbuk-do	Boeun-gun	20.2 (18.8,21.6)	28.7 (24.4,33.0)	22.6 (19.3,26.0)	17.9 (15.1,20.7)	18.2 (15.2,21.3)	14.6 (11.9,17.3)	14.1 (10.3,19.9)	Rural
Women	Chungcheongbuk-do	Okcheon-gun	18.3 (17.0,19.7)	22.2 (18.8,25.6)	19.9 (16.9,22.8)	18.6 (15.6,21.5)	16.1 (13.5,18.7)	14.9 (12.2,17.6)	7.3 (3.3,12.3)	Rural
Women	Chungcheongbuk-do	Yeongdong-gun	18.8 (17.5,20.2)	27.1 (23.3,31.0)	20.2 (17.1,23.2)	17.2 (14.4,19.9)	15.0 (12.5,17.5)	14.8 (12.0,17.5)	12.3 (6.9,16.1)	Rural
Women	Chungcheongbuk-do	Jincheon-gun	19.0 (17.6,20.4)	26.2 (22.5,30.0)	18.5 (15.3,21.6)	18.7 (15.7,21.8)	14.9 (12.2,17.6)	16.9 (14.0,19.8)	9.3 (4.6,14.4)	Rural
Women	Chungcheongbuk-do	Goesan-gun	18.9 (17.5,20.3)	25.2 (21.5,28.9)	19.0 (15.9,22.1)	17.9 (14.9,20.8)	17.3 (14.5,20.2)	15.3 (12.5,18.1)	9.9 (8.4,17.6)	Rural
Women	Chungcheongbuk-do	Eumseong-gun	20.1 (18.6,21.5)	27.7 (23.8,31.6)	23.6 (19.9,27.2)	19.2 (16.2,22.2)	15.6 (13.1,18.1)	14.5 (11.8,17.2)	13.2 (9.4,19.0)	Rural
Women	Chungcheongbuk-do	Danyang-gun	20.1 (18.7,21.6)	30.2 (26.2,34.3)	19.3 (16.1,22.4)	18.0 (15.0,21.0)	17.2 (14.5,20.0)	16.8 (13.9,19.7)	13.4 (7.6,17.2)	Rural
Women	Chungcheongbuk-do	Jeungpyeong-gun	16.4 (15.2,17.6)	20.4 (17.3,23.6)	16.8 (14.2,19.4)	16.0 (13.4,18.5)	15.3 (12.7,18.0)	13.2 (10.7,15.6)	7.2 (4.7,13.3)	Rural
Women	Chungcheongnam-do	Cheonan-si	17.6 (16.1,19.1)	25.3 (21.1,29.5)	19.1 (15.6,22.5)	16.3 (13.2,19.4)	14.1 (11.1,17.0)	14.2 (11.2,17.2)	11.1 (5.9,15.3)	Metropolitan
Women	Chungcheongnam-do	Gongju-si	19.7 (18.3,21.0)	25.5 (21.8,29.2)	19.0 (16.0,21.9)	20.0 (17.0,23.1)	18.5 (15.6,21.5)	15.3 (12.5,18.1)	10.2 (5.2,14.6)	Urban
Women	Chungcheongnam-do	Boryeong-si	22.0 (20.5,23.5)	31.3 (27.1,35.5)	25.6 (21.9,29.3)	17.6 (14.9,20.4)	20.0 (16.9,23.1)	15.9 (13.2,18.6)	15.4 (10.1,19.9)	Urban
Women	Chungcheongnam-do	Asan-si	18.4 (17.0,19.9)	26.5 (22.4,30.6)	20.3 (17.0,23.6)	16.5 (13.5,19.6)	14.4 (11.5,17.2)	14.5 (11.6,17.3)	12.0 (8.6,17.8)	Urban
Women	Chungcheongnam-do	Seosan-si	18.1 (16.8,19.5)	22.9 (19.4,26.3)	20.1 (16.9,23.3)	16.2 (13.4,19.1)	16.6 (13.7,19.6)	15.1 (12.4,17.8)	7.8 (3.1,12.3)	Urban
Women	Chungcheongnam-do	Nonsan-si	18.7 (17.4,20.0)	27.2 (23.4,31.0)	18.2 (15.6,20.9)	17.1 (14.4,19.8)	16.9 (14.1,19.7)	13.9 (11.4,16.3)	13.3 (8.2,17.2)	Urban
Women	Chungcheongnam-do	Gyeryong-si	15.9 (14.4,17.3)	21.0 (17.4,24.7)	18.2 (14.8,21.6)	12.7 (9.9,15.6)	14.8 (11.8,17.8)	12.6 (9.7,15.4)	8.4 (2.3,11.1)	Urban
Women	Chungcheongnam-do	Dangjin-si	18.9 (17.5,20.3)	25.7 (21.9,29.5)	20.8 (17.5,24.0)	19.4 (16.2,22.6)	14.7 (12.0,17.3)	14.3 (11.6,17.1)	11.4 (4.9,14.1)	Rural
Women	Chungcheongnam-do	Geumsan-gun	18.6 (17.3,19.9)	24.3 (20.7,28.0)	21.2 (18.2,24.2)	17.1 (14.5,19.7)	16.4 (13.7,19.1)	13.8 (11.5,16.2)	10.5 (5.4,14.4)	Rural
Women	Chungcheongnam-do	Buyeo-gun	17.4 (16.2,18.7)	22.0 (18.5,25.4)	19.5 (16.5,22.5)	16.3 (13.7,18.9)	16.5 (13.8,19.2)	13.2 (10.7,15.7)	8.8 (3.0,11.4)	Rural
Women	Chungcheongnam-do	Seocheon-gun	18.4 (17.0,19.7)	25.7 (21.9,29.5)	17.4 (14.5,20.2)	16.6 (13.9,19.3)	19.0 (15.9,22.0)	13.4 (11.0,15.8)	12.3 (6.6,15.4)	Rural
Women	Chungcheongnam-do	Cheongyang-gun	16.9 (15.6,18.2)	22.3 (18.8,25.8)	18.8 (15.8,21.8)	18.8 (15.7,21.8)	13.2 (10.7,15.7)	11.1 (9.0,13.3)	11.2 (7.0,15.6)	Rural
Women	Chungcheongnam-do	Hongseong-gun	18.3 (17.0,19.6)	22.0 (18.7,25.3)	20.9 (17.8,24.1)	16.7 (14.0,19.4)	17.0 (14.2,19.8)	14.9 (12.3,17.6)	7.1 (5.0,14.0)	Rural
Women	Chungcheongnam-do	Yesan-gun	17.9 (16.6,19.3)	26.3 (22.5,30.2)	19.9 (16.9,23.0)	14.6 (12.2,17.1)	15.2 (12.6,17.8)	14.3 (11.5,17.0)	12.0 (8.7,18.1)	Rural
Women	Chungcheongnam-do	Taean-gun	17.9 (16.6,19.2)	23.0 (19.6,26.5)	20.4 (17.4,23.5)	17.9 (15.0,20.9)	15.3 (12.6,18.1)	12.9 (10.4,15.4)	10.1 (5.5,14.3)	Urban
Women	Jeollabuk-do	Jeonju-si	15.8 (14.5,17.1)	23.8 (20.1,27.5)	16.6 (13.7,19.6)	14.7 (11.9,17.6)	13.0 (10.4,15.6)	11.7 (9.2,14.2)	12.1 (9.1,17.5)	Metropolitan
Women	Jeollabuk-do	Gunsan-si	20.1 (18.6,21.5)	29.4 (25.4,33.5)	21.4 (18.1,24.6)	17.3 (14.3,20.2)	18.1 (15.0,21.1)	14.6 (11.9,17.2)	14.8 (9.0,18.4)	Urban
Women	Jeollabuk-do	Iksan-si	20.8 (19.4,22.3)	31.6 (27.6,35.6)	20.8 (17.6,24.0)	19.1 (16.0,22.1)	17.3 (14.4,20.2)	15.7 (13.0,18.4)	15.9 (11.0,20.6)	Urban
Women	Jeollabuk-do	Jeongeup-si	20.6 (19.2,22.0)	31.9 (27.7,36.0)	19.1 (16.2,22.0)	20.0 (17.0,22.9)	15.5 (12.8,18.1)	17.0 (14.2,19.8)	14.9 (10.0,19.2)	Urban
Women	Jeollabuk-do	Namwon-si	20.7 (19.3,22.1)	27.1 (23.4,30.7)	23.0 (19.7,26.3)	19.4 (16.4,22.4)	16.4 (13.8,19.1)	17.6 (14.8,20.3)	9.5 (6.9,16.1)	Urban
Women	Jeollabuk-do	Gimje-si	19.0 (17.7,20.4)	26.9 (23.0,30.8)	18.9 (16.0,21.8)	16.7 (14.0,19.3)	17.8 (14.8,20.8)	14.9 (12.3,17.5)	12.0 (6.4,15.8)	Urban
Women	Jeollabuk-do	Wanju-gun	19.3 (18.0,20.7)	29.1 (25.1,33.1)	20.2 (17.2,23.2)	18.2 (15.4,21.1)	15.1 (12.5,17.8)	14.6 (12.1,17.1)	14.5 (7.7,17.1)	Rural
Women	Jeollabuk-do	Jinan-gun	18.4 (17.0,19.8)	25.4 (21.4,29.5)	22.5 (19.1,26.0)	15.0 (12.7,17.4)	17.4 (14.3,20.4)	11.8 (9.5,14.2)	13.6 (8.1,17.1)	Rural

Women	Jeollabuk-do	Muju-gun	21.4 (19.9,22.8)	26.8 (23.0,30.7)	25.1 (21.4,28.8)	19.1 (16.3,22.0)	18.4 (15.4,21.3)	17.1 (14.1,20.1)	9.7 (3.2,12.8)	Rural
Women	Jeollabuk-do	Jangsu-gun	19.8 (18.4,21.2)	24.3 (20.6,28.1)	21.5 (18.3,24.7)	18.4 (15.5,21.3)	15.8 (13.1,18.4)	19.4 (16.2,22.6)	4.9 (2.5,11.9)	Rural
Women	Jeollabuk-do	Imsil-gun	19.5 (18.1,20.9)	23.9 (20.0,27.8)	19.0 (16.1,21.9)	27.3 (23.5,31.1)	13.4 (10.9,16.0)	12.6 (10.3,14.9)	11.3 (7.9,16.7)	Rural
Women	Jeollabuk-do	Sunchang-gun	19.6 (18.2,21.0)	27.5 (23.6,31.5)	21.7 (18.3,25.0)	17.9 (15.2,20.6)	15.5 (12.9,18.2)	14.8 (12.1,17.5)	12.7 (6.3,15.5)	Rural
Women	Jeollabuk-do	Gochang-gun	24.4 (22.9,26.0)	31.4 (27.2,35.6)	23.8 (20.6,27.1)	23.1 (19.8,26.5)	23.8 (20.4,27.2)	20.6 (17.3,23.8)	10.8 (5.8,15.4)	Rural
Women	Jeollabuk-do	Buan-gun	21.1 (19.6,22.5)	32.0 (27.6,36.4)	20.4 (17.5,23.3)	19.9 (16.8,23.1)	15.2 (12.6,17.8)	18.3 (15.2,21.3)	13.7 (8.6,18.2)	Rural
Women	Jeollanam-do	Mokpo-si	19.5 (18.1,21.0)	26.5 (22.7,30.3)	22.0 (18.6,25.4)	19.8 (16.6,23.0)	15.9 (13.1,18.7)	13.4 (10.8,16.1)	13.1 (6.0,15.0)	Urban
Women	Jeollanam-do	Yeosu-si	17.9 (16.6,19.3)	23.3 (19.9,26.8)	17.4 (14.5,20.2)	17.9 (15.0,20.7)	14.1 (11.5,16.6)	17.3 (14.4,20.3)	6.0 (1.9,10.9)	Urban
Women	Jeollanam-do	Suncheon-si	16.7 (15.4,18.0)	22.6 (19.0,26.2)	19.2 (16.1,22.2)	15.2 (12.4,18.1)	15.8 (13.0,18.7)	11.2 (8.9,13.6)	11.4 (9.2,17.8)	Urban
Women	Jeollanam-do	Naju-si	19.1 (17.8,20.4)	28.8 (25.0,32.7)	21.3 (18.1,24.4)	16.6 (14.0,19.2)	14.9 (12.5,17.3)	13.8 (11.4,16.2)	15.0 (11.9,21.1)	Urban
Women	Jeollanam-do	Gwangyang-si	16.8 (15.4,18.1)	24.1 (20.2,27.9)	18.0 (14.8,21.2)	15.5 (12.6,18.3)	14.1 (11.2,17.0)	12.6 (9.9,15.2)	11.5 (6.9,16.1)	Urban
Women	Jeollanam-do	Damyang-gun	19.2 (17.8,20.5)	24.8 (21.1,28.5)	21.1 (18.0,24.3)	16.4 (13.8,19.0)	17.2 (14.5,20.0)	16.6 (13.7,19.5)	8.2 (6.6,15.2)	Rural
Women	Jeollanam-do	Gokseong-gun	18.3 (17.0,19.6)	23.1 (19.6,26.6)	18.5 (15.8,21.2)	19.4 (16.4,22.4)	16.1 (13.5,18.7)	14.5 (12.2,16.9)	8.6 (5.7,14.3)	Rural
Women	Jeollanam-do	Gurye-gun	14.3 (13.2,15.5)	21.1 (17.8,24.5)	16.7 (14.0,19.3)	12.3 (10.2,14.4)	10.6 (8.5,12.6)	11.5 (9.2,13.8)	9.6 (7.2,15.6)	Rural
Women	Jeollanam-do	Goheung-gun	16.2 (14.9,17.4)	26.0 (21.8,30.2)	16.6 (14.0,19.1)	17.4 (14.4,20.4)	11.9 (9.9,13.9)	8.9 (7.2,10.6)	17.1 (14.2,22.2)	Rural
Women	Jeollanam-do	Boseong-gun	20.1 (18.7,21.5)	29.1 (24.9,33.3)	21.7 (18.5,25.0)	20.8 (17.6,24.0)	16.1 (13.5,18.7)	13.3 (11.2,15.4)	15.8 (11.8,21.0)	Rural
Women	Jeollanam-do	Hwasun-gun	20.7 (19.4,22.1)	31.6 (27.6,35.7)	19.9 (17.0,22.8)	20.4 (17.3,23.5)	16.6 (14.0,19.2)	15.8 (13.3,18.4)	15.8 (11.6,21.0)	Rural
Women	Jeollanam-do	Jangheung-gun	16.0 (14.8,17.1)	22.4 (18.9,25.9)	16.1 (13.5,18.7)	14.3 (11.8,16.7)	12.5 (10.4,14.6)	14.9 (12.2,17.5)	7.5 (2.6,11.0)	Rural
Women	Jeollanam-do	Gangjin-gun	15.7 (14.5,16.9)	21.7 (18.4,25.1)	14.6 (12.1,17.0)	14.1 (11.5,16.6)	14.7 (12.0,17.3)	13.6 (11.0,16.2)	8.1 (1.5,9.7)	Rural
Women	Jeollanam-do	Haenam-gun	19.1 (17.8,20.4)	24.6 (21.0,28.2)	19.7 (16.8,22.5)	18.2 (15.5,20.8)	17.1 (14.3,20.0)	16.1 (13.6,18.7)	8.5 (4.9,14.1)	Rural
Women	Jeollanam-do	Yeongam-gun	19.3 (18.0,20.7)	26.5 (22.8,30.3)	21.0 (17.9,24.2)	19.2 (16.2,22.3)	17.4 (14.6,20.2)	12.4 (10.1,14.7)	14.1 (8.7,17.7)	Rural
Women	Jeollanam-do	Muan-gun	16.1 (14.9,17.3)	19.6 (16.5,22.6)	16.4 (13.7,19.1)	17.6 (14.8,20.3)	13.3 (11.0,15.7)	13.8 (11.2,16.4)	5.8 (3.1,11.5)	Rural
Women	Jeollanam-do	Hampyeong-gun	21.7 (20.2,23.2)	30.7 (26.4,34.9)	22.2 (18.9,25.5)	19.7 (16.5,22.9)	18.5 (15.5,21.6)	17.3 (14.3,20.3)	13.4 (11.2,20.8)	Rural
Women	Jeollanam-do	Yeonggwang-gun	21.7 (20.3,23.1)	30.7 (26.8,34.7)	23.5 (20.3,26.8)	19.6 (16.6,22.6)	17.8 (15.2,20.4)	17.4 (14.4,20.4)	13.3 (10.5,20.3)	Rural
Women	Jeollanam-do	Jangseong-gun	18.3 (17.0,19.6)	25.9 (22.0,29.7)	19.3 (16.3,22.2)	16.5 (13.8,19.1)	17.0 (14.2,19.7)	13.3 (10.9,15.7)	12.6 (3.1,12.1)	Rural
Women	Jeollanam-do	Wando-gun	18.4 (17.1,19.7)	25.7 (22.0,29.4)	21.2 (18.3,24.2)	16.2 (13.6,18.9)	15.8 (13.4,18.2)	13.0 (10.7,15.3)	12.7 (9.5,18.3)	Rural
Women	Jeollanam-do	Jindo-gun	20.7 (19.3,22.1)	29.2 (25.1,33.3)	21.6 (18.4,24.7)	19.6 (16.7,22.6)	18.0 (15.2,20.8)	15.3 (12.6,18.0)	13.9 (10.4,19.6)	Rural
Women	Jeollanam-do	Sinan-gun	22.0 (20.5,23.6)	26.5 (22.8,30.3)	27.7 (23.6,31.8)	17.0 (14.3,19.7)	22.2 (18.8,25.7)	16.9 (13.9,19.9)	9.6 (8.8,18.6)	Rural
Women	Gyeongsangbuk-do	Nam-gu	19.7 (18.3,21.2)	28.6 (24.7,32.6)	20.1 (16.9,23.3)	18.7 (15.5,21.8)	15.2 (12.3,18.0)	16.0 (13.1,18.9)	12.6 (7.2,16.8)	Metropolitan
Women	Gyeongsangbuk-do	Buk-gu	17.9 (16.6,19.3)	26.6 (22.7,30.4)	18.4 (15.3,21.5)	16.4 (13.5,19.2)	15.1 (12.3,17.9)	13.5 (10.9,16.2)	13.1 (7.6,16.6)	Metropolitan
Women	Gyeongsangbuk-do	Gyeongju-si	17.8 (16.6,19.1)	24.8 (21.3,28.4)	19.6 (16.6,22.5)	16.8 (14.0,19.5)	15.6 (13.1,18.0)	12.8 (10.5,15.2)	12.0 (8.6,17.4)	Urban
Women	Gyeongsangbuk-do	Gimcheon-si	17.1 (15.8,18.3)	21.6 (18.4,24.8)	18.0 (15.2,20.8)	17.3 (14.5,20.2)	14.7 (12.0,17.3)	13.5 (11.0,16.0)	8.1 (3.5,12.3)	Urban
Women	Gyeongsangbuk-do	Andong-si	18.9 (17.5,20.2)	26.7 (23.0,30.3)	21.8 (18.5,25.1)	17.5 (14.8,20.3)	14.8 (12.2,17.5)	14.0 (11.5,16.5)	12.7 (7.1,16.3)	Urban
Women	Gyeongsangbuk-do	Gumi-si	18.7 (17.6,19.9)	27.7 (24.4,30.9)	20.8 (18.0,23.6)	15.7 (13.3,18.0)	16.1 (13.7,18.5)	14.7 (12.6,16.8)	13.0 (8.1,14.7)	Urban
Women	Gyeongsangbuk-do	Yeongju-si	19.5 (18.1,20.8)	26.3 (22.6,30.0)	21.0 (17.9,24.0)	18.8 (15.9,21.7)	16.0 (13.4,18.6)	15.7 (13.1,18.4)	10.6 (5.8,15.2)	Urban
Women	Gyeongsangbuk-do	Yeongcheon-si	21.0 (19.6,22.4)	25.6 (22.0,29.1)	23.6 (20.4,26.8)	19.7 (16.6,22.7)	20.6 (17.5,23.7)	15.7 (12.9,18.4)	9.9 (3.6,12.8)	Urban
Women	Gyeongsangbuk-do	Sangju-si	18.7 (17.3,20.0)	26.3 (22.6,30.1)	17.8 (15.0,20.7)	17.4 (14.6,20.2)	15.8 (13.2,18.4)	15.9 (13.0,18.7)	10.4 (3.8,12.8)	Urban

Women	Gyeongsangbuk-do	Mungyeong-si	18.7 (17.4,20.0)	22.7 (19.3,26.1)	20.4 (17.5,23.4)	18.8 (16.0,21.6)	17.8 (14.9,20.7)	13.6 (11.3,15.9)	9.1 (3.5,12.3)	Urban
Women	Gyeongsangbuk-do	Gyeongsan-si	18.5 (17.1,19.9)	24.7 (21.0,28.4)	20.7 (17.5,24.0)	15.7 (12.9,18.5)	17.3 (14.3,20.2)	14.3 (11.6,16.9)	10.4 (5.8,14.8)	Urban
Women	Gyeongsangbuk-do	Gunwi-gun	18.8 (17.5,20.2)	25.9 (22.0,29.9)	18.9 (16.1,21.7)	17.6 (14.7,20.4)	17.1 (14.2,19.9)	14.9 (12.2,17.6)	11.0 (4.3,13.7)	Rural
Women	Gyeongsangbuk-do	Uiseong-gun	18.4 (17.0,19.8)	25.3 (21.4,29.3)	18.2 (15.3,21.2)	16.1 (13.3,18.9)	17.9 (14.9,20.8)	14.8 (12.0,17.6)	10.5 (3.5,12.5)	Rural
Women	Gyeongsangbuk-do	Cheongsong-gun	20.0 (18.6,21.4)	27.3 (23.2,31.3)	21.4 (18.3,24.6)	17.5 (14.8,20.2)	18.1 (15.2,21.0)	15.9 (13.3,18.4)	11.4 (7.1,16.5)	Rural
Women	Gyeongsangbuk-do	Yeongyang-gun	23.2 (21.6,24.8)	34.2 (29.4,39.1)	23.9 (20.4,27.3)	22.1 (18.8,25.4)	19.9 (16.7,23.0)	16.5 (13.8,19.1)	17.7 (9.3,19.3)	Rural
Women	Gyeongsangbuk-do	Yeongdeok-gun	17.6 (16.3,18.9)	25.1 (21.4,28.8)	20.9 (17.6,24.2)	15.2 (13.0,17.4)	14.3 (11.9,16.8)	12.2 (10.0,14.5)	12.9 (9.1,17.5)	Rural
Women	Gyeongsangbuk-do	Cheongdo-gun	19.3 (18.0,20.7)	22.0 (18.8,25.2)	19.6 (16.6,22.7)	16.4 (13.9,18.8)	19.5 (16.3,22.6)	19.6 (16.3,22.8)	2.4 (-2.3,6.7)	Rural
Women	Gyeongsangbuk-do	Goryeong-gun	20.1 (18.7,21.5)	25.6 (21.9,29.3)	19.6 (16.6,22.5)	19.7 (16.5,22.8)	17.5 (14.8,20.3)	18.2 (15.1,21.3)	7.4 (1.8,11.0)	Rural
Women	Gyeongsangbuk-do	Seongju-gun	13.3 (12.2,14.4)	20.4 (17.0,23.7)	14.9 (12.2,17.5)	11.5 (9.3,13.6)	10.0 (8.0,12.1)	9.8 (7.7,12.0)	10.6 (5.0,13.0)	Rural
Women	Gyeongsangbuk-do	Chilgok-gun	18.5 (17.1,19.9)	23.7 (20.0,27.5)	18.6 (15.4,21.9)	19.6 (16.3,22.9)	15.6 (12.7,18.5)	15.6 (12.7,18.5)	8.1 (3.0,12.2)	Rural
Women	Gyeongsangbuk-do	Yecheon-gun	19.8 (18.4,21.2)	28.5 (24.4,32.7)	21.3 (18.1,24.4)	20.4 (17.0,23.8)	15.3 (12.8,17.8)	13.4 (11.1,15.7)	15.1 (7.7,16.9)	Rural
Women	Gyeongsangbuk-do	Bonghwa-gun	23.0 (21.4,24.6)	32.2 (27.5,37.0)	23.5 (20.0,27.0)	20.9 (17.7,24.0)	23.0 (19.4,26.6)	15.9 (13.2,18.5)	16.3 (10.9,20.7)	Rural
Women	Gyeongsangbuk-do	Uljin-gun	20.6 (19.2,21.9)	30.5 (26.4,34.5)	22.3 (19.2,25.4)	20.2 (17.3,23.2)	16.9 (14.2,19.7)	13.4 (11.2,15.6)	17.1 (12.0,21.2)	Rural
Women	Gyeongsangbuk-do	Ulleung-gun	17.7 (16.3,19.1)	22.4 (18.9,25.9)	20.1 (17.0,23.3)	17.1 (14.0,20.2)	15.1 (12.3,18.0)	13.4 (10.7,16.2)	9.0 (6.1,15.5)	Rural
Women	Gyeongsangnam-do	Changwon-si	20.3 (18.6,21.9)	26.5 (22.1,31.0)	20.8 (17.1,24.5)	19.6 (16.1,23.2)	19.1 (15.5,22.6)	16.0 (12.7,19.3)	10.5 (4.8,14.2)	Metropolitan
Women	Gyeongsangnam-do	Masan-si	15.9 (14.6,17.1)	23.4 (20.0,26.9)	15.8 (13.0,18.6)	14.5 (11.8,17.2)	14.9 (12.2,17.6)	11.2 (8.8,13.5)	12.2 (6.5,15.1)	Metropolitan
Women	Gyeongsangnam-do	Jinju-si	17.5 (16.2,18.8)	24.7 (21.1,28.2)	17.8 (14.8,20.7)	16.7 (13.9,19.5)	14.6 (12.0,17.2)	14.3 (11.6,17.0)	10.4 (5.9,14.9)	Urban
Women	Gyeongsangnam-do	Jinhae-si	17.6 (16.2,19.0)	22.3 (18.7,25.9)	19.4 (16.1,22.6)	16.1 (13.1,19.1)	15.7 (12.8,18.6)	14.8 (12.0,17.7)	7.5 (3.4,12.6)	Metropolitan
Women	Gyeongsangnam-do	Tongyeong-si	21.9 (20.4,23.3)	29.0 (25.1,32.9)	22.1 (18.8,25.4)	20.3 (17.2,23.5)	17.9 (15.0,20.8)	20.7 (17.4,23.9)	8.3 (3.9,13.7)	Urban
Women	Gyeongsangnam-do	Sacheon-si	17.8 (16.5,19.1)	25.4 (21.7,29.1)	20.3 (17.2,23.4)	15.7 (13.1,18.4)	14.6 (12.1,17.2)	13.5 (11.0,15.9)	11.9 (6.4,15.2)	Urban
Women	Gyeongsangnam-do	Gimhae-si	18.5 (17.0,20.0)	23.4 (19.5,27.4)	18.4 (15.1,21.7)	17.4 (14.2,20.6)	16.0 (13.0,19.1)	17.6 (14.4,20.8)	5.8 (1.4,10.6)	Urban
Women	Gyeongsangnam-do	Miryang-si	19.1 (17.8,20.4)	25.7 (22.3,29.2)	21.2 (18.1,24.3)	20.3 (17.2,23.3)	15.4 (12.9,17.8)	13.1 (10.8,15.4)	12.6 (9.3,18.1)	Urban
Women	Gyeongsangnam-do	Geoje-si	18.5 (16.9,20.0)	21.6 (17.9,25.4)	20.5 (16.9,24.2)	19.0 (15.5,22.5)	16.0 (12.7,19.2)	15.2 (12.1,18.2)	6.4 (1.6,10.8)	Urban
Women	Gyeongsangnam-do	Yangsan-si	15.2 (13.9,16.5)	23.1 (19.5,26.6)	15.5 (12.4,18.5)	13.8 (11.1,16.6)	12.1 (9.5,14.6)	11.8 (9.3,14.4)	11.3 (4.2,13.0)	Urban
Women	Gyeongsangnam-do	Uiryeong-gun	18.0 (16.7,19.3)	22.5 (19.1,25.9)	20.4 (17.1,23.7)	16.2 (13.5,18.8)	15.9 (13.3,18.5)	15.6 (12.7,18.5)	6.9 (5.6,14.6)	Rural
Women	Gyeongsangnam-do	Haman-gun	17.6 (16.4,18.8)	22.1 (18.9,25.3)	17.9 (15.2,20.7)	17.5 (14.8,20.2)	14.7 (12.2,17.2)	16.0 (13.3,18.7)	6.1 (2.6,11.6)	Rural
Women	Gyeongsangnam-do	Changnyeong-gun	20.7 (19.3,22.1)	25.8 (22.1,29.4)	21.9 (18.8,24.9)	18.7 (15.8,21.5)	18.1 (15.5,20.8)	19.3 (16.2,22.5)	6.5 (-0.3,9.3)	Rural
Women	Gyeongsangnam-do	Goseong-gun	17.3 (16.1,18.6)	20.5 (17.3,23.7)	18.2 (15.5,21.0)	17.9 (15.1,20.8)	17.2 (14.3,20.0)	12.9 (10.5,15.2)	7.6 (3.3,11.9)	Rural
Women	Gyeongsangnam-do	Namhae-gun	23.5 (22.0,25.1)	32.8 (28.3,37.4)	25.8 (22.2,29.4)	22.8 (19.4,26.1)	19.2 (16.3,22.1)	17.7 (14.6,20.8)	15.1 (9.8,19.4)	Rural
Women	Gyeongsangnam-do	Hadong-gun	23.5 (22.0,25.0)	30.7 (26.4,34.9)	27.1 (23.5,30.8)	20.6 (17.5,23.6)	18.8 (16.1,21.6)	20.4 (17.1,23.7)	10.3 (2.6,12.4)	Rural
Women	Gyeongsangnam-do	Sancheong-gun	20.8 (19.4,22.2)	27.1 (23.1,31.1)	23.6 (20.2,27.0)	19.5 (16.7,22.2)	18.2 (15.5,21.0)	15.6 (12.9,18.3)	11.5 (6.8,16.0)	Rural
Women	Gyeongsangnam-do	Hamyang-gun	20.6 (19.2,22.0)	23.7 (20.3,27.2)	22.3 (19.0,25.5)	19.4 (16.5,22.3)	19.2 (16.2,22.2)	18.5 (15.4,21.7)	5.2 (0.9,10.3)	Rural
Women	Gyeongsangnam-do	Geochang-gun	18.1 (16.8,19.3)	25.2 (21.7,28.7)	17.2 (14.5,19.8)	19.1 (16.2,22.1)	15.1 (12.6,17.6)	13.9 (11.5,16.4)	11.3 (6.2,15.2)	Rural
Women	Gyeongsangnam-do	Hapcheon-gun	21.0 (19.6,22.4)	25.7 (21.9,29.6)	24.5 (20.9,28.0)	21.8 (18.6,25.0)	18.5 (15.6,21.5)	14.7 (12.3,17.1)	11.0 (3.8,13.0)	Rural
Women	Jeju-do	Jeju-si	16.6 (15.9,17.4)	24.3 (22.1,26.5)	19.6 (17.6,21.6)	14.3 (12.6,16.0)	12.7 (11.2,14.2)	12.9 (11.4,14.4)	11.4 (9.1,14.3)	Urban
Women	Jeju-do	Seogwipo-si	16.4 (15.6,17.1)	23.3 (21.2,25.4)	17.3 (15.6,19.1)	14.9 (13.3,16.6)	13.0 (11.5,14.4)	13.8 (12.2,15.4)	9.5 (6.1,11.5)	Urban

Volume: 39, Article ID: e2017011,

https://doi.org/10.4178/epih.e2017011

Appendix 5. Mean equivalized household income and its relative ratios and absolute differences among metropolitan, urban, and rural areas: Findings for total study population, those with the lowest and highest income quintiles using the Community Health Survey in South Korea, 2008-2014

Subjects	Measures	Metropolitan	Urban	Rural
Total study population	Mean equivalized house income	2119.2	1831.9	1407.1
	Standard deviation	1971.4	1826.9	1830.1
	Relative ratio	1.5	1.3	Reference
	Absolute difference	712.1	424.8	Reference
Those with Lowest income quintile	Mean equivalized house income	818.7	674.7	444.8
	Standard deviation	410.5	391.8	292.2
	Relative ratio	1.8	1.5	Reference
	Absolute difference	373.9	229.9	Reference
Those with Highest income quintile	Mean equivalized house income	4088.0	3638.5	3036.4
	Standard deviation	3496.9	3245.4	3453.5
	Relative ratio	1.3	1.2	Reference
	Absolute difference	1051.6	602.1	Reference
