# Supplementary material for: Income gaps in self-rated poor health and its association with life expectancy in 245 districts of Korea
Source: Epidemiol Health. 2017 Mar 15;39:e2017011. doi: 10.4178/epih.e2017011 (PMC5543297; doi:10.4178/epih.e2017011)
Supplement: Supplementary file 2 [file epih-39-e2017011-app1.pdf]

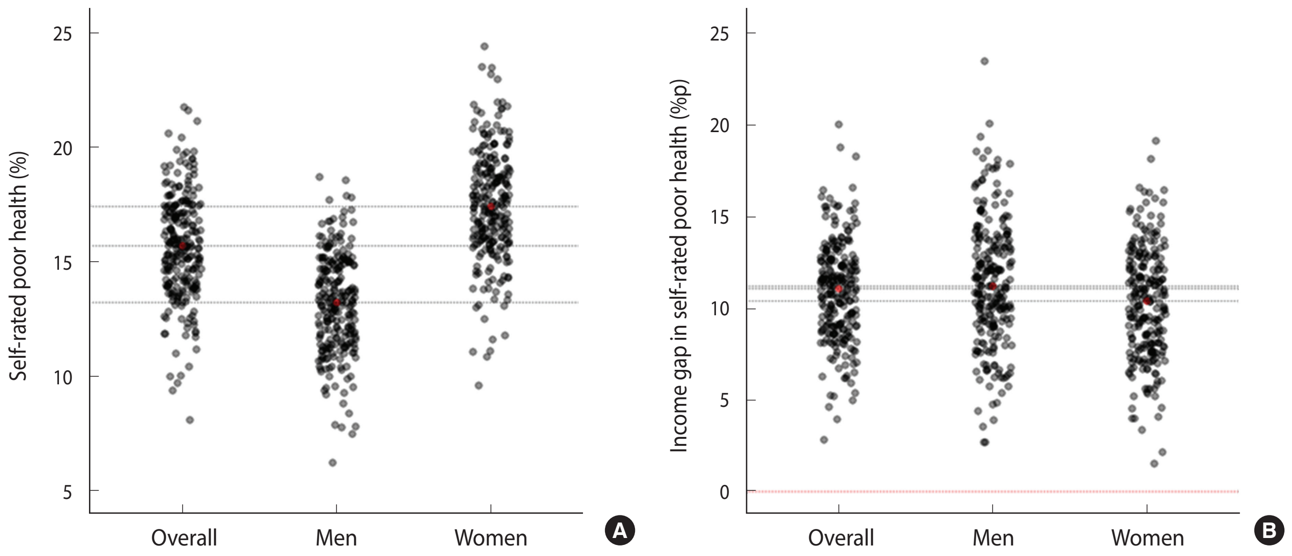

Appendix 1. Distribution of (A) age-standardized prevalences of self-rated poor health, and of (B) interquintile income gaps in self-rated poor health in the 245 local districts in South Korea, findings from the Korea Community Health Survey, 2008-2014.
