# Supplementary material for: Income gaps in self-rated poor health and its association with life expectancy in 245 districts of Korea
Source: Epidemiol Health. 2017 Mar 15;39:e2017011. doi: 10.4178/epih.e2017011 (PMC5543297; doi:10.4178/epih.e2017011)
Supplement: Supplementary file 3 [file epih-39-e2017011-app2.pdf]

Appendix 2. Ten top and bottom districts in terms of age-standardized prevalences of self-rated poor health in Korea: findings from the Korea Community Health Survey, 2008-2014

	Top 10			Bottom 10		
	Province	District	Self-rated poor health (%)	Province	District	Self-rated poor health (%)
Total	Jeollabuk-do	Gochang-gun	21.8	Gyeonggi-do	Bundang-gu	8.1
	Gyeongsangnam-do	Namhae-gun	21.6	Seoul	Seocho-gu	9.4
	Gyeongsangnam-do	Hadong-gun	21.2	Seoul	Gangnam-gu	9.7
	Gyeongsangbuk-do	Yeongyang-gun	20.6	Seoul	Songpa-gu	10.0
	Gangwon-do	Taebaek-si	20.4	Gyeonggi-do	Suji-gu	10.0
	Gangwon-do	Jeongseon-gun	19.9	Seoul	Yongsan-gu	10.4
	Chungcheongnam-do	Boryeong-si	19.8	Gyeonggi-do	Giheung-gu	11.0
	Jeollanam-do	Sinan-gun	19.8	Gyeonggi-do	Yeongtong-gu	11.2
	Gyeongsangbuk-do	Bonghwa-gun	19.7	Gyeonggi-do	Ilsanseo-gu	11.7
	Jeollabuk-do	Buan-gun	19.5	Gyeonggi-do	Dongan-gu	11.8
Men	Gangwon-do	Taebaek-si	18.7	Gyeonggi-do	Bundang-gu	6.2
	Gyeongsangnam-do	Namhae-gun	18.6	Seoul	Seocho-gu	7.5
	Jeollabuk-do	Gochang-gun	17.9	Seoul	Gangnam-gu	7.8
	Gangwon-do	Jeongseon-gun	17.8	Seoul	Songpa-gu	7.8
	Gyeongsangnam-do	Hadong-gun	17.7	Gyeonggi-do	Suji-gu	7.9
	Gyeongsangbuk-do	Yeongyang-gun	17.3	Gyeonggi-do	Giheung-gu	8.4
	Jeollabuk-do	Buan-gun	17.2	Gyeonggi-do	Gwacheon-si	8.8
	Gyeongsangbuk-do	Cheongsong-gun	16.9	Gyeonggi-do	Dongan-gu	9.2
	Gyeongsangnam-do	Sancheong-gun	16.9	Seoul	Mapo-gu	9.3
	Chungcheongnam-do	Boryeong-si	16.8	Gyeonggi-do	Ilsandong-gu	9.4
Women	Jeollabuk-do	Gochang-gun	24.4	Gyeonggi-do	Bundang-gu	9.6
	Gyeongsangnam-do	Namhae-gun	23.5	Seoul	Seocho-gu	10.8
	Gyeongsangnam-do	Hadong-gun	23.5	Seoul	Yongsan-gu	11.1
	Gyeongsangbuk-do	Yeongyang-gun	23.2	Seoul	Gangnam-gu	11.1
	Gyeongsangbuk-do	Bonghwa-gun	23.0	Gyeonggi-do	Suji-gu	11.6
	Chungcheongnam-do	Boryeong-si	22.0	Seoul	Songpa-gu	11.8
	Jeollanam-do	Sinan-gun	22.0	Gyeonggi-do	Yeongtong-gu	12.5
	Gyeongsangnam-do	Tongyeong-si	21.9	Seoul	Seongbuk-gu	13.0
	Gangwon-do	Taebaek-si	21.8	Gyeonggi-do	Giheung-gu	13.1
	Jeollanam-do	Hampyeong-gun	21.7	Gyeonggi-do	Gimpo-si	13.1
