# Supplementary material for: Income gaps in self-rated poor health and its association with life expectancy in 245 districts of Korea
Source: Epidemiol Health. 2017 Mar 15;39:e2017011. doi: 10.4178/epih.e2017011 (PMC5543297; doi:10.4178/epih.e2017011)
Supplement: Supplementary file 4 [file epih-39-e2017011-app3.pdf]

Appendix 3. Ten top and bottom districts in terms of income gaps in self-rated poor health between the lowest and highest income quintiles in South Korea: findings from the Korea Community Health Survey, 2008-2014

	Top 10			Bottom 10		
	Province	District	Interquintile income gap in self-rated poor health (%p)	Province	District	Interquintile income gap in self-rated poor health (%p)
Total	Jeollanam-do	Goheung-gun	20.1	Gyeonggi-do	Suji-gu	2.8
	Busan	Haeundae-gu	18.8	Gyeonggi-do	Bundang-gu	4.0
	Gyeongsangbuk-do	Yecheon-gun	18.3	Gyeonggi-do	Yeongtong-gu	4.6
	Jeollanam-do	Boseong-gun	16.6	Incheon	Ongjin-gun	5.0
	Gyeongsangbuk-do	Yeongdeok-gun	16.5	Seoul	Songpa-gu	5.2
	Jeollanam-do	Naju-si	16.1	Gyeonggi-do	Dongan-gu	5.2
	Jeollanam-do	Wando-gun	16.0	Gyeonggi-do	Gwangmyeong-si	5.4
	Gangwon-do	Samcheok-si	15.8	Seoul	Seocho-gu	5.9
	Gyeongsangnam-do	Namhae-gun	15.7	Gyeonggi-do	Gimpo-si	6.2
	Chungcheongnam-do	Boryeong-si	15.6	Gyeonggi-do	Osan-si	6.2
Men	Jeollanam-do	Goheung-gun	23.5	Gyeonggi-do	Yeongtong-gu	2.7
	Jeollanam-do	Wando-gun	20.1	Gyeonggi-do	Bundang-gu	2.7
	Jeollanam-do	Yeonggwang-gun	19.4	Seoul	Seongdong-gu	3.6
	Jeollabuk-do	Gochang-gun	18.6	Gyeonggi-do	Dongan-gu	3.9
	Jeollanam-do	Boseong-gun	18.6	Gyeonggi-do	Cheoin-gu	4.4
	Gyeongsangnam-do	Miryang-si	18.2	Incheon	Yeonsu-gu	4.8
	Jeollabuk-do	Imsil-gun	18.1	Gyeonggi-do	Suji-gu	4.8
	Busan	Haeundae-gu	17.9	Busan	Sasang-gu	5.4
	Jeollanam-do	Naju-si	17.8	Gyeonggi-do	Yeoju-gun	5.7
	Gyeongsangbuk-do	Yecheon-gun	17.7	Gyeonggi-do	Gunpo-si	5.7
Women	Busan	Haeundae-gu	19.2	Gyeonggi-do	Suji-gu	1.5
	Jeollanam-do	Goheung-gun	18.2	Gyeongsangbuk-do	Cheongdo-gun	2.2
	Gyeongsangbuk-do	Uljin-gun	16.6	Gyeonggi-do	Gimpo-si	3.4
	Jeollanam-do	Naju-si	16.5	Gyeonggi-do	Ilsanseo-gu	4.0
	Jeollanam-do	Boseong-gun	16.4	Gyeonggi-do	Bundang-gu	4.0
	Jeollanam-do	Hwasun-gun	16.3	Gyeonggi-do	Osan-si	4.1
	Jeollanam-do	Hampyeong-gun	16.0	Gyeongsangnam-do	Changnyeong-gun	4.5
	Gyeongsangbuk-do	Bonghwa-gun	15.8	Seoul	Songpa-gu	4.6
	Jeollabuk-do	Iksan-si	15.8	Gyeonggi-do	Dongan-gu	5.2
	Jeollanam-do	Yeonggwang-gun	15.4	Gyeonggi-do	Yeongtong-gu	5.2
