# Supplementary material for: Income gaps in self-rated poor health and its association with life expectancy in 245 districts of Korea
Source: Epidemiol Health. 2017 Mar 15;39:e2017011. doi: 10.4178/epih.e2017011 (PMC5543297; doi:10.4178/epih.e2017011)
Supplement: Supplementary file 5 [file epih-39-e2017011-app4.pdf]

Appendix 4. Age-standardized prevalences of self-rated poor health, self-rated poor health prevalences according to income quintiles, and income gaps in self-rated poor health (between the highest [Q5] and the lowest [Q1] income quintiles) in 245 local districts of South Korea: findings from the Korea Community Health Survey, 2008-2014

Gender	Provinces	Districts	Overall	Q1 (lowest)	Q2	Q3	Q4	Q5 (highest)	Q1-Q5	Urbanity
Total	Seoul	Jongno-gu	12.5 (11.7,13.4)	20.3 (17.8,22.7)	12.6 (10.8,14.5)	12.2 (10.3,14.1)	9.8 (8.1,11.4)	8.2 (6.7,9.7)	12.1 (7.1,13.1)	Metropolitan
	Seoul	Jung-gu	13.5 (12.6,14.4)	21.7 (19.1,24.2)	14.5 (12.5,16.6)	12.7 (10.8,14.5)	10.2 (8.5,11.9)	9.2 (7.5,10.8)	12.5 (9.2,15.4)	Metropolitan
	Seoul	Yongsan-gu	10.4 (9.7,11.2)	16.9 (14.6,19.2)	9.8 (8.1,11.5)	8.8 (7.2,10.4)	8.7 (7.1,10.3)	8.1 (6.6,9.6)	8.8 (8.2,14.4)	Metropolitan
	Seoul	Seongdong-gu	12.8 (11.9,13.8)	16.3 (14.0,18.6)	14.3 (12.2,16.3)	12.9 (10.8,14.9)	11.2 (9.3,13.0)	9.6 (7.8,11.4)	6.7 (2.4,7.8)	Metropolitan
	Seoul	Gwangjin-gu	12.3 (11.3,13.2)	16.4 (14.0,18.8)	13.5 (11.3,15.6)	11.5 (9.5,13.5)	11.2 (9.3,13.0)	9.0 (7.2,10.8)	7.4 (5.8,11.4)	Metropolitan
	Seoul	Dongdaemun-gu	14.0 (13.1,14.9)	18.9 (16.5,21.4)	15.0 (13.0,17.1)	13.3 (11.3,15.2)	12.5 (10.5,14.4)	10.3 (8.5,12.1)	8.6 (5.5,11.3)	Metropolitan
	Seoul	Jungnang-gu	13.1 (12.2,14.0)	19.8 (17.3,22.4)	14.2 (12.1,16.2)	11.6 (9.7,13.4)	11.3 (9.4,13.2)	8.8 (7.1,10.5)	11.0 (8.1,13.9)	Metropolitan
	Seoul	Seongbuk-gu	11.9 (11.1,12.8)	18.8 (16.4,21.2)	11.1 (9.3,12.9)	11.0 (9.2,12.7)	10.4 (8.6,12.2)	8.5 (6.9,10.1)	10.3 (7.1,12.9)	Metropolitan
	Seoul	Gangbuk-gu	13.9 (13.0,14.8)	18.4 (16.0,20.8)	16.4 (14.2,18.5)	12.9 (11.0,14.8)	11.2 (9.4,12.9)	10.4 (8.7,12.1)	8.0 (4.7,10.7)	Metropolitan
	Seoul	Dobong-gu	13.8 (12.9,14.7)	19.4 (17.0,21.9)	14.5 (12.5,16.6)	13.0 (11.0,14.9)	10.8 (9.0,12.5)	11.7 (9.8,13.6)	7.7 (4.9,11.1)	Metropolitan
	Seoul	Nowon-gu	14.2 (13.2,15.1)	23.8 (21.0,26.5)	15.8 (13.7,18.0)	12.3 (10.4,14.3)	10.1 (8.3,11.8)	8.8 (7.1,10.5)	15.0 (10.0,16.0)	Metropolitan
	Seoul	Eunpyeong-gu	14.3 (13.3,15.2)	20.7 (18.2,23.3)	15.7 (13.5,17.9)	13.6 (11.6,15.6)	11.4 (9.6,13.2)	10.1 (8.3,11.8)	10.6 (7.1,13.1)	Metropolitan
	Seoul	Seodaemun-gu	13.7 (12.8,14.6)	20.2 (17.7,22.7)	14.9 (12.8,17.0)	12.0 (10.1,13.9)	12.8 (10.9,14.7)	8.8 (7.2,10.4)	11.4 (8.1,13.9)	Metropolitan
	Seoul	Mapo-gu	12.0 (11.1,12.9)	17.0 (14.7,19.4)	15.0 (12.8,17.2)	11.8 (9.9,13.8)	9.1 (7.4,10.8)	7.2 (5.7,8.8)	9.8 (6.9,13.1)	Metropolitan
	Seoul	Yangcheon-gu	13.5 (12.6,14.5)	21.5 (18.7,24.2)	13.5 (11.4,15.6)	11.6 (9.6,13.5)	11.0 (9.0,12.9)	10.7 (8.8,12.6)	10.8 (6.0,12.4)	Metropolitan
	Seoul	Gangseo-gu	13.7 (12.7,14.6)	20.7 (18.1,23.4)	15.8 (13.6,18.0)	12.6 (10.6,14.6)	10.1 (8.3,11.9)	9.4 (7.7,11.2)	11.3 (7.5,13.3)	Metropolitan
	Seoul	Guro-gu	14.2 (13.3,15.2)	22.0 (19.2,24.7)	13.5 (11.4,15.6)	14.1 (12.1,16.2)	11.2 (9.3,13.1)	10.7 (8.8,12.6)	11.3 (7.6,13.8)	Metropolitan
	Seoul	Geumcheon-gu	14.4 (13.5,15.3)	21.0 (18.4,23.6)	15.3 (13.1,17.4)	13.8 (11.7,15.8)	10.8 (9.0,12.6)	11.4 (9.5,13.3)	9.6 (4.5,10.5)	Metropolitan
	Seoul	Yeongdeungpo-gu	13.9 (13.0,14.9)	21.1 (18.5,23.7)	14.8 (12.7,17.0)	12.7 (10.7,14.6)	10.1 (8.3,11.8)	11.0 (9.1,12.9)	10.1 (7.4,13.4)	Metropolitan
	Seoul	Dongjak-gu	14.3 (13.3,15.2)	19.0 (16.5,21.5)	15.1 (12.9,17.3)	12.6 (10.6,14.6)	14.0 (12.0,16.1)	10.9 (9.1,12.7)	8.1 (3.9,9.9)	Metropolitan
	Seoul	Gwanak-gu	14.2 (13.3,15.2)	18.7 (16.1,21.3)	15.7 (13.4,18.0)	14.3 (12.1,16.4)	11.4 (9.4,13.4)	11.4 (9.4,13.4)	7.3 (5.4,11.2)	Metropolitan
	Seoul	Seocho-gu	9.4 (8.6,10.2)	12.9 (10.8,15.0)	11.6 (9.6,13.6)	8.8 (7.1,10.5)	7.1 (5.6,8.6)	7.0 (5.4,8.6)	5.9 (3.7,8.9)	Metropolitan
	Seoul	Gangnam-gu	9.7 (8.8,10.5)	14.6 (12.3,17.0)	10.5 (8.5,12.4)	8.8 (7.1,10.6)	8.0 (6.4,9.7)	6.7 (5.2,8.3)	7.9 (5.8,11.2)	Metropolitan
	Seoul	Songpa-gu	10.0 (9.2,10.9)	13.2 (11.0,15.4)	9.4 (7.6,11.2)	10.0 (8.1,11.9)	9.6 (7.8,11.3)	8.0 (6.3,9.7)	5.2 (4.0,9.2)	Metropolitan
	Seoul	Gangdong-gu	13.3 (12.3,14.2)	17.6 (15.1,20.0)	16.0 (13.8,18.3)	12.1 (10.1,14.1)	10.7 (8.8,12.7)	9.9 (8.0,11.9)	7.7 (5.2,11.6)	Metropolitan
	Busan	Jung-gu	15.0 (14.1,15.9)	21.4 (18.9,23.9)	16.8 (14.7,18.8)	15.5 (13.4,17.5)	12.0 (10.3,13.8)	9.8 (8.2,11.4)	11.6 (7.9,14.1)	Metropolitan
	Busan	Seo-gu	15.1 (14.2,16.0)	24.2 (21.6,26.8)	14.9 (13.0,16.9)	13.6 (11.7,15.5)	12.3 (10.6,14.1)	10.6 (8.9,12.3)	13.6 (8.8,15.2)	Metropolitan
	Busan	Dong-gu	18.7 (17.7,19.7)	29.4 (26.5,32.3)	18.6 (16.3,20.8)	16.6 (14.5,18.6)	15.2 (13.2,17.2)	13.8 (11.9,15.7)	15.6 (11.3,18.1)	Metropolitan
	Busan	Yeongdo-gu	18.0 (17.0,18.9)	27.9 (25.1,30.8)	18.8 (16.6,21.1)	16.4 (14.3,18.4)	14.1 (12.2,16.0)	13.1 (11.3,14.9)	14.8 (10.6,17.4)	Metropolitan
	Busan	Busanjin-gu	15.2 (14.2,16.1)	23.2 (20.6,25.8)	16.4 (14.2,18.6)	13.5 (11.5,15.5)	12.2 (10.4,14.0)	10.6 (8.8,12.3)	12.6 (10.0,16.2)	Metropolitan
	Busan	Dongnae-gu	13.5 (12.6,14.4)	19.2 (16.9,21.5)	14.2 (12.1,16.2)	11.8 (10.0,13.6)	12.0 (10.2,13.8)	10.2 (8.4,12.0)	9.0 (6.9,12.7)	Metropolitan
	Busan	Nam-gu	14.4 (13.5,15.3)	19.9 (17.4,22.3)	15.7 (13.6,17.8)	14.2 (12.2,16.2)	12.5 (10.7,14.4)	10.1 (8.4,11.9)	9.8 (7.5,13.5)	Metropolitan
	Busan	Buk-gu	14.9 (13.9,15.9)	23.6 (20.8,26.4)	14.8 (12.6,17.0)	14.8 (12.6,17.0)	10.4 (8.6,12.2)	11.2 (9.2,13.2)	12.4 (10.9,17.1)	Metropolitan
	Busan	Haeundae-gu	14.7 (13.8,15.7)	26.9 (24.1,29.8)	14.4 (12.3,16.5)	12.3 (10.4,14.2)	12.3 (10.3,14.2)	8.1 (6.5,9.6)	18.8 (15.3,21.5)	Metropolitan
	Busan	Saha-gu	14.6 (13.6,15.5)	22.8 (20.1,25.5)	14.7 (12.6,16.8)	12.5 (10.5,14.4)	11.5 (9.6,13.4)	11.7 (9.8,13.6)	11.1 (8.1,14.5)	Metropolitan
	Busan	Geumjeong-gu	13.3 (12.4,14.2)	21.5 (18.9,24.1)	14.5 (12.5,16.5)	12.5 (10.6,14.4)	10.5 (8.8,12.3)	8.2 (6.6,9.7)	13.3 (10.6,16.4)	Metropolitan
	Busan	Gangseo-gu	16.8 (15.9,17.8)	23.0 (20.4,25.5)	18.1 (15.9,20.3)	15.3 (13.2,17.3)	14.2 (12.2,16.1)	13.8 (11.8,15.7)	9.2 (5.7,12.1)	Metropolitan
	Busan	Yeonje-gu	15.1 (14.1,16.0)	24.7 (21.9,27.4)	14.7 (12.7,16.8)	12.6 (10.6,14.5)	11.2 (9.5,13.0)	12.4 (10.4,14.3)	12.3 (8.6,15.0)	Metropolitan
	Busan	Suyeong-gu	13.7 (12.9,14.6)	20.0 (17.6,22.3)	15.0 (12.9,17.0)	11.2 (9.5,13.0)	11.9 (10.1,13.7)	10.6 (8.9,12.3)	9.4 (6.2,12.4)	Metropolitan
	Busan	Sasang-gu	16.4 (15.4,17.4)	22.3 (19.6,25.1)	17.0 (14.7,19.4)	15.1 (12.9,17.3)	14.0 (11.9,16.2)	13.7 (11.5,15.9)	8.6 (4.8,11.2)	Metropolitan
	Busan	Gijang-gun	17.9 (16.9,18.9)	23.8 (21.1,26.4)	20.6 (18.2,23.1)	17.3 (15.1,19.4)	15.6 (13.5,17.6)	12.5 (10.6,14.3)	11.3 (7.7,14.3)	Rural
	Daegu	Jung-gu	17.0 (16.0,18.0)	25.5 (22.8,28.3)	17.9 (15.8,20.1)	15.4 (13.4,17.5)	13.4 (11.6,15.3)	12.8 (10.9,14.7)	12.7 (9.2,15.8)	Metropolitan
	Daegu	Dong-gu	18.1 (17.1,19.1)	25.6 (22.8,28.4)	18.3 (16.0,20.6)	17.8 (15.6,20.0)	16.2 (14.0,18.4)	12.9 (10.9,14.8)	12.7 (9.4,15.8)	Metropolitan
	Daegu	Seo-gu	18.2 (17.2,19.2)	25.3 (22.5,28.1)	18.4 (16.1,20.8)	16.1 (14.0,18.3)	17.3 (15.1,19.6)	13.7 (11.7,15.7)	11.6 (5.6,12.2)	Metropolitan
	Daegu	Nam-gu	15.8 (14.9,16.8)	22.8 (20.2,25.4)	17.5 (15.3,19.8)	15.0 (13.0,17.0)	12.9 (11.0,14.8)	11.1 (9.3,12.9)	11.7 (9.5,15.7)	Metropolitan
	Daegu	Buk-gu	14.7 (13.7,15.7)	22.5 (19.7,25.3)	15.6 (13.3,17.8)	14.9 (12.7,17.1)	10.8 (9.0,12.7)	10.1 (8.3,12.0)	12.4 (10.5,16.7)	Metropolitan
	Daegu	Suseong-gu	15.1 (14.2,16.1)	23.7 (21.0,26.5)	16.1 (13.8,18.3)	14.5 (12.4,16.5)	11.5 (9.6,13.4)	10.3 (8.5,12.1)	13.4 (9.1,15.3)	Metropolitan
	Daegu	Dalseo-gu	16.0 (14.9,17.0)	23.6 (20.8,26.3)	18.3 (15.8,20.9)	13.9 (11.8,16.1)	12.9 (10.9,15.0)	11.4 (9.4,13.4)	12.2 (8.9,15.1)	Metropolitan
	Daegu	Dalseong-gun	17.2 (16.2,18.2)	23.2 (20.5,25.9)	18.3 (15.9,20.6)	16.1 (13.9,18.3)	13.8 (11.7,15.8)	14.9 (12.7,17.0)	8.3 (6.9,13.3)	Rural
	Incheon	Jung-gu	14.7 (13.8,15.6)	21.2 (18.7,23.8)	14.5 (12.5,16.5)	14.7 (12.6,16.7)	11.7 (9.9,13.5)	11.5 (9.6,13.3)	9.7 (7.4,14.6)	Metropolitan
	Incheon	Dong-gu	14.1 (13.3,15.0)	19.9 (17.4,22.3)	14.9 (12.9,17.0)	14.3 (12.3,16.3)	11.1 (9.4,12.8)	10.9 (9.1,12.6)	9.0 (5.3,11.3)	Metropolitan
	Incheon	Nam-gu	12.6 (11.7,13.4)	19.0 (16.5,21.5)	12.4 (10.4,14.3)	11.2 (9.3,13.0)	10.9 (9.1,12.7)	9.9 (8.2,11.7)	9.1 (5.6,11.4)	Metropolitan
	Incheon	Yeonsu-gu	13.9 (12.9,15.0)	21.1 (18.2,23.9)	14.5 (12.1,16.8)	12.8 (10.6,14.9)	11.6 (9.6,13.7)	10.3 (8.3,12.3)	10.8 (6.9,13.3)	Metropolitan
	Incheon	Namdong-gu	15.0 (14.0,16.0)	21.5 (18.7,24.3)	15.4 (13.1,17.8)	15.1 (12.8,17.4)	10.6 (8.7,12.5)	12.3 (10.3,14.4)	9.2 (7.7,14.3)	Metropolitan
	Incheon	Bupyeong-gu	16.0 (14.9,17.0)	24.3 (21.4,27.3)	16.0 (13.7,18.4)	15.5 (13.2,17.7)	12.9 (10.9,14.9)	11.8 (9.8,13.8)	12.5 (9.2,16.2)	Metropolitan
	Incheon	Gyeyang-gu	15.7 (14.6,16.8)	22.3 (19.3,25.3)	17.5 (14.8,20.1)	13.7 (11.5,15.9)	12.9 (10.7,15.1)	12.6 (10.4,14.8)	9.7 (6.3,13.1)	Metropolitan
	Incheon	Seo-gu	16.2 (15.1,17.3)	21.9 (19.0,24.9)	16.2 (13.7,18.6)	15.5 (13.0,17.9)	14.3 (12.0,16.6)	13.5 (11.2,15.8)	8.4 (5.5,12.7)	Metropolitan
	Incheon	Ganghwa-gun	16.3 (15.3,17.2)	22.0 (19.5,24.6)	17.7 (15.5,19.9)	15.4 (13.5,17.4)	12.7 (10.9,14.4)	13.4 (11.5,15.4)	8.6 (4.3,10.7)	Rural

(continued to the next page)

Appendix 4. Continued

Gender	Provinces	Districts	Overall	Q1 (lowest)	Q2	Q3	Q4	Q5 (highest)	Q1-Q5	Urbanity
	Incheon	Ongjin-gun	12.7 (11.9,13.6)	16.5 (14.3,18.7)	12.7 (10.9,14.6)	11.7 (9.9,13.5)	11.2 (9.5,12.9)	11.5 (9.7,13.3)	5.0 (3.0,9.8)	Rural
	Gwangju	Dong-gu	15.1 (14.2,16.0)	23.4 (20.7,26.0)	16.9 (14.8,19.1)	13.5 (11.6,15.4)	11.8 (10.1,13.6)	10.5 (8.8,12.2)	12.9 (10.3,16.5)	Metropolitan
	Gwangju	Seo-gu	13.8 (12.8,14.7)	19.7 (17.2,22.3)	15.5 (13.2,17.8)	11.9 (9.9,13.9)	11.7 (9.8,13.7)	10.5 (8.6,12.3)	9.2 (6.4,12.2)	Metropolitan
	Gwangju	Nam-gu	15.3 (14.4,16.3)	23.0 (20.4,25.7)	16.5 (14.3,18.7)	14.2 (12.3,16.2)	12.1 (10.2,14.0)	11.4 (9.6,13.2)	11.6 (8.9,15.1)	Metropolitan
	Gwangju	Buk-gu	15.7 (14.7,16.7)	23.1 (20.4,25.8)	15.3 (13.1,17.5)	15.3 (13.1,17.5)	13.5 (11.5,15.6)	11.5 (9.5,13.4)	11.6 (7.2,13.2)	Metropolitan
	Gwangju	Gwangsan-gu	15.8 (14.7,16.8)	23.9 (20.9,27.0)	17.2 (14.7,19.7)	14.7 (12.4,17.0)	11.7 (9.6,13.8)	11.6 (9.5,13.8)	12.3 (8.7,15.5)	Metropolitan
	Daejeon	Dong-gu	14.9 (14.0,15.8)	25.4 (22.6,28.3)	16.0 (13.9,18.1)	12.0 (10.1,13.8)	11.5 (9.7,13.4)	10.1 (8.4,11.8)	15.3 (10.7,16.9)	Metropolitan
	Daejeon	Jung-gu	13.3 (12.4,14.2)	20.7 (18.2,23.3)	13.3 (11.4,15.3)	12.6 (10.7,14.6)	11.5 (9.7,13.3)	8.9 (7.3,10.5)	11.8 (7.8,13.8)	Metropolitan
	Daejeon	Seo-gu	14.0 (13.0,15.0)	21.2 (18.3,24.0)	15.1 (12.8,17.3)	12.8 (10.6,15.0)	11.7 (9.7,13.7)	9.7 (7.8,11.6)	11.5 (9.8,15.8)	Metropolitan
	Daejeon	Yuseong-gu	12.4 (11.4,13.4)	17.3 (14.7,19.8)	15.0 (12.6,17.4)	9.9 (8.0,11.8)	10.8 (8.7,12.9)	8.5 (6.5,10.4)	8.8 (5.9,11.7)	Metropolitan
	Daejeon	Daedeok-gu	13.9 (13.0,14.9)	19.4 (16.8,22.0)	15.7 (13.5,17.9)	12.0 (10.0,14.0)	12.3 (10.2,14.3)	10.4 (8.5,12.3)	9.0 (5.7,13.1)	Metropolitan
	Ulsan	Jung-gu	15.7 (14.7,16.8)	22.0 (19.3,24.7)	15.4 (13.1,17.6)	13.9 (11.7,16.2)	13.7 (11.6,15.8)	13.7 (11.5,15.9)	8.3 (5.7,12.1)	Metropolitan
	Ulsan	Nam-gu	13.1 (12.1,14.1)	17.8 (14.9,20.7)	13.6 (11.3,15.9)	12.3 (10.0,14.5)	11.5 (9.4,13.6)	10.8 (8.7,13.0)	7.0 (4.9,10.7)	Metropolitan
	Ulsan	Dong-gu	12.5 (11.5,13.5)	19.6 (16.7,22.5)	14.4 (11.9,16.9)	11.0 (8.9,13.1)	8.9 (7.0,10.8)	9.3 (7.2,11.4)	10.3 (7.8,13.4)	Metropolitan
	Ulsan	Buk-gu	12.3 (11.3,13.4)	15.8 (13.1,18.5)	14.2 (11.8,16.6)	12.6 (10.3,15.0)	10.1 (8.0,12.2)	8.8 (6.8,10.9)	7.0 (4.3,10.3)	Metropolitan
	Ulsan	Ulju-gun	17.4 (16.4,18.5)	25.4 (22.6,28.3)	18.7 (16.3,21.1)	15.3 (13.2,17.5)	13.8 (11.8,15.8)	14.6 (12.5,16.7)	10.8 (7.8,14.4)	Rural
	Sejong	Sejong	17.0 (16.1,18.0)	24.0 (21.4,26.7)	18.2 (16.0,20.4)	16.9 (14.8,19.1)	14.0 (12.1,15.9)	12.4 (10.6,14.3)	11.6 (8.1,14.5)	Metropolitan
	Gyeonggi-do	Jangan-gu	13.6 (12.7,14.6)	18.5 (16.0,21.0)	15.8 (13.5,18.0)	12.4 (10.3,14.4)	11.7 (9.7,13.7)	9.7 (7.8,11.5)	8.8 (6.1,12.1)	Metropolitan
	Gyeonggi-do	Gwonseon-gu	14.0 (12.9,15.0)	20.2 (17.4,22.9)	14.1 (11.8,16.4)	13.1 (11.0,15.3)	11.8 (9.7,13.9)	10.7 (8.7,12.8)	9.5 (5.7,11.7)	Metropolitan
	Gyeonggi-do	Paldal-gu	14.1 (13.2,15.1)	20.4 (17.7,23.0)	14.7 (12.5,16.9)	12.1 (10.1,14.1)	11.9 (9.9,13.9)	11.8 (9.7,13.8)	8.6 (5.5,12.5)	Metropolitan
	Gyeonggi-do	Yeongtong-gu	11.2 (10.1,12.2)	14.9 (12.1,17.6)	10.5 (8.2,12.7)	9.6 (7.4,11.7)	10.8 (8.5,13.2)	10.3 (8.0,12.7)	4.6 (2.3,10.1)	Metropolitan
	Gyeonggi-do	Sujeong-gu	15.7 (14.6,16.7)	22.0 (19.3,24.8)	16.8 (14.5,19.1)	14.1 (11.9,16.3)	13.6 (11.6,15.7)	11.8 (9.8,13.9)	10.2 (7.8,14.6)	Metropolitan
	Gyeonggi-do	Jungwon-gu	16.6 (15.5,17.6)	24.1 (21.2,26.9)	17.3 (15.0,19.7)	14.6 (12.4,16.8)	13.8 (11.6,15.9)	13.3 (11.2,15.4)	10.8 (7.7,14.3)	Metropolitan
	Gyeonggi-do	Bundang-gu	8.1 (7.3,8.8)	11.0 (9.1,13.0)	8.2 (6.5,9.8)	6.7 (5.2,8.1)	7.5 (5.9,9.1)	7.0 (5.4,8.6)	4.0 (0.9,6.1)	Metropolitan
	Gyeonggi-do	Uijeongbu-si	15.2 (14.2,16.1)	23.4 (20.6,26.1)	17.0 (14.7,19.3)	12.7 (10.7,14.7)	11.9 (10.0,13.8)	11.5 (9.6,13.3)	11.9 (8.2,14.6)	Urban
	Gyeonggi-do	Manan-gu	13.6 (12.7,14.6)	20.1 (17.5,22.7)	14.8 (12.6,17.0)	12.0 (10.1,13.9)	11.2 (9.3,13.1)	10.6 (8.7,12.4)	9.5 (7.4,13.4)	Metropolitan
	Gyeonggi-do	Dongan-gu	11.8 (10.9,12.7)	15.8 (13.4,18.3)	12.1 (10.0,14.3)	11.3 (9.4,13.2)	9.2 (7.4,11.0)	10.6 (8.7,12.6)	5.2 (0.6,6.4)	Metropolitan
	Gyeonggi-do	Wonmi-gu	14.1 (13.0,15.1)	19.9 (17.1,22.6)	15.1 (12.7,17.6)	12.2 (10.2,14.3)	12.0 (9.9,14.1)	11.6 (9.5,13.6)	8.3 (4.6,10.8)	Metropolitan
	Gyeonggi-do	Sosa-gu	14.2 (13.2,15.2)	20.0 (17.3,22.7)	14.8 (12.6,17.0)	13.3 (11.2,15.4)	12.7 (10.6,14.7)	10.8 (8.9,12.7)	9.2 (7.4,13.6)	Metropolitan
	Gyeonggi-do	Ojeong-gu	15.2 (14.2,16.2)	23.7 (20.8,26.5)	15.6 (13.3,17.8)	11.7 (9.7,13.6)	12.1 (10.1,14.1)	13.1 (10.9,15.2)	10.6 (6.2,12.8)	Metropolitan
	Gyeonggi-do	Gwangmyeong-si	13.9 (12.9,14.8)	18.4 (15.8,20.9)	15.2 (13.0,17.4)	12.2 (10.2,14.3)	10.7 (8.8,12.6)	13.0 (10.8,15.1)	5.4 (5.3,13.1)	Urban
	Gyeonggi-do	Pyeongtaek-si	14.9 (14.2,15.5)	22.0 (20.1,23.8)	15.1 (13.5,16.6)	13.4 (11.9,14.8)	12.6 (11.2,14.0)	11.5 (10.2,12.8)	10.5 (9.2,13.6)	Urban
	Gyeonggi-do	Dongducheon-si	16.3 (15.4,17.3)	23.8 (21.1,26.5)	17.3 (15.0,19.6)	15.4 (13.3,17.5)	14.0 (12.0,16.0)	11.5 (9.7,13.4)	12.3 (9.0,15.4)	Urban
	Gyeonggi-do	Sangnok-gu	13.8 (12.7,14.8)	19.9 (17.2,22.7)	14.5 (12.1,16.8)	12.5 (10.3,14.7)	11.4 (9.4,13.4)	10.7 (8.7,12.7)	9.2 (8.6,14.6)	Metropolitan
	Gyeonggi-do	Danwon-gu	15.3 (14.2,16.4)	22.6 (19.5,25.7)	15.6 (13.1,18.0)	14.0 (11.6,16.3)	12.6 (10.3,14.9)	12.1 (9.8,14.3)	10.5 (7.0,13.4)	Metropolitan
	Gyeonggi-do	Deogyang-gu	15.4 (14.4,16.4)	23.7 (20.9,26.4)	16.4 (14.2,18.7)	13.1 (11.0,15.1)	12.0 (10.1,13.9)	11.9 (9.9,13.9)	11.8 (6.3,12.5)	Metropolitan
	Gyeonggi-do	Ilsandong-gu	12.5 (11.6,13.5)	16.9 (14.4,19.4)	13.4 (11.2,15.5)	12.9 (10.8,14.9)	11.1 (9.2,13.0)	8.7 (6.9,10.4)	8.2 (5.6,11.8)	Metropolitan
	Gyeonggi-do	Ilsanseo-gu	11.7 (10.8,12.6)	17.7 (15.2,20.2)	11.2 (9.3,13.2)	10.1 (8.3,12.0)	9.4 (7.7,11.1)	10.5 (8.6,12.4)	7.2 (2.3,8.1)	Metropolitan
	Gyeonggi-do	Gwacheon-si	11.9 (11.0,12.8)	16.7 (14.3,19.1)	11.6 (9.7,13.5)	11.1 (9.2,13.0)	10.9 (9.0,12.8)	9.4 (7.6,11.1)	7.3 (3.9,9.7)	Urban
	Gyeonggi-do	Guri-si	15.3 (14.3,16.4)	21.4 (18.7,24.2)	15.6 (13.3,17.9)	14.5 (12.3,16.8)	14.5 (12.3,16.6)	10.6 (8.7,12.6)	10.8 (5.4,11.6)	Urban
	Gyeonggi-do	Namyangju-si	14.8 (13.8,15.8)	19.6 (17.0,22.1)	15.6 (13.4,17.9)	13.3 (11.3,15.3)	12.5 (10.5,14.5)	13.3 (11.2,15.4)	6.3 (3.7,10.1)	Urban
	Gyeonggi-do	Osan-si	13.9 (12.9,15.0)	17.6 (14.9,20.3)	13.2 (10.9,15.6)	13.3 (11.1,15.5)	14.3 (11.9,16.7)	11.4 (9.2,13.6)	6.2 (1.9,8.3)	Urban
	Gyeonggi-do	Siheung-si	14.7 (13.6,15.8)	19.7 (16.9,22.5)	15.8 (13.3,18.2)	12.8 (10.6,14.9)	13.8 (11.5,16.1)	11.5 (9.3,13.6)	8.2 (5.7,13.3)	Urban
	Gyeonggi-do	Gunpo-si	14.5 (13.5,15.5)	21.9 (19.1,24.7)	13.5 (11.4,15.6)	12.2 (10.2,14.3)	13.4 (11.2,15.5)	11.9 (9.8,13.9)	10.0 (5.6,11.8)	Urban
	Gyeonggi-do	Uiwang-si	12.8 (11.9,13.7)	18.2 (15.7,20.6)	13.8 (11.6,16.0)	10.4 (8.6,12.3)	11.2 (9.4,13.1)	10.7 (8.8,12.6)	7.5 (5.1,11.1)	Urban
	Gyeonggi-do	Hanam-si	14.6 (13.7,15.6)	18.9 (16.4,21.4)	15.8 (13.6,18.1)	13.6 (11.5,15.7)	12.9 (11.0,14.9)	12.4 (10.4,14.4)	6.5 (3.7,10.3)	Urban
	Gyeonggi-do	Cheoin-gu	13.4 (12.5,14.3)	18.2 (15.8,20.6)	14.4 (12.3,16.5)	12.6 (10.7,14.5)	12.2 (10.2,14.1)	9.8 (8.0,11.5)	8.4 (1.7,8.9)	Metropolitan
	Gyeonggi-do	Giheung-gu	11.0 (10.2,11.9)	16.2 (13.8,18.6)	10.9 (9.0,12.8)	10.2 (8.4,12.1)	10.0 (8.1,11.8)	8.1 (6.4,9.7)	8.1 (4.5,10.1)	Metropolitan
	Gyeonggi-do	Suji-gu	10.0 (9.1,10.8)	12.0 (9.9,14.0)	10.1 (8.2,11.9)	10.4 (8.5,12.2)	8.4 (6.7,10.0)	9.2 (7.5,11.0)	2.8 (-0.1,5.5)	Metropolitan
	Gyeonggi-do	Paju-si	14.7 (13.8,15.7)	21.7 (19.0,24.4)	15.4 (13.3,17.6)	13.4 (11.4,15.4)	11.6 (9.7,13.4)	12.0 (10.1,13.9)	9.7 (5.4,11.6)	Urban
	Gyeonggi-do	Icheon-si	15.4 (14.4,16.3)	21.3 (18.7,23.9)	16.1 (13.9,18.3)	13.4 (11.4,15.5)	13.5 (11.4,15.5)	12.5 (10.5,14.5)	8.8 (6.4,12.6)	Urban
	Gyeonggi-do	Anseong-si	15.3 (14.3,16.2)	20.9 (18.4,23.3)	15.7 (13.6,17.9)	13.5 (11.5,15.5)	13.8 (11.8,15.8)	12.2 (10.4,14.1)	8.7 (4.0,11.4)	Urban
	Gyeonggi-do	Gimpo-si	11.9 (11.1,12.8)	15.7 (13.4,17.9)	12.6 (10.6,14.6)	11.2 (9.4,13.1)	10.8 (9.0,12.6)	9.5 (7.8,11.3)	6.2 (2.9,8.5)	Urban
	Gyeonggi-do	Hwaseongsi	13.6 (12.7,14.6)	19.5 (16.9,22.1)	14.8 (12.5,17.1)	12.8 (10.7,14.9)	11.9 (9.9,13.9)	9.4 (7.5,11.2)	10.1 (8.0,13.8)	Urban
	Gyeonggi-do	Gwangju-si	13.2 (12.3,14.1)	17.4 (15.0,19.8)	14.3 (12.2,16.4)	12.5 (10.5,14.4)	10.6 (8.7,12.4)	11.1 (9.2,13.0)	6.3 (4.2,11.2)	Urban
	Gyeonggi-do	Yangju-si	15.5 (14.5,16.5)	21.6 (19.0,24.3)	15.8 (13.6,18.0)	13.3 (11.3,15.3)	14.7 (12.6,16.7)	12.5 (10.5,14.5)	9.1 (6.1,12.3)	Urban
	Gyeonggi-do	Pocheon-si	16.6 (15.6,17.6)	23.8 (21.2,26.5)	17.7 (15.5,19.9)	15.2 (13.2,17.3)	14.1 (12.2,16.0)	12.4 (10.6,14.3)	11.4 (6.9,13.3)	Urban
	Gyeonggi-do	Yeoju-gun	16.6 (15.6,17.5)	21.4 (19.0,23.9)	18.4 (16.2,20.6)	15.1 (13.1,17.1)	14.4 (12.5,16.4)	13.8 (11.8,15.7)	7.6 (2.9,9.3)	Urban
	Gyeonggi-do	Yeoncheon-gun	15.7 (14.8,16.6)	22.9 (20.4,25.5)	16.5 (14.5,18.5)	15.1 (13.1,17.0)	13.5 (11.6,15.3)	10.6 (9.0,12.2)	12.3 (8.2,14.4)	Rural
	Gyeonggi-do	Gapyeong-gun	14.9 (14.0,15.8)	23.2 (20.6,25.8)	16.6 (14.6,18.7)	13.5 (11.7,15.4)	10.8 (9.2,12.5)	10.5 (8.8,12.1)	12.7 (9.4,15.6)	Rural

(continued to the next page)

Appendix 4. Continued

Gender	Provinces	Districts	Overall	Q1 (lowest)	Q2	Q3	Q4	Q5 (highest)	Q1-Q5	Urbanity
	Gyeonggi-do	Yangpyeong-gun	15.9 (15.0,16.8)	20.4 (18.0,22.8)	18.0 (15.8,20.1)	14.7 (12.7,16.6)	15.0 (13.1,17.0)	11.4 (9.6,13.1)	9.0 (5.8,12.0)	Rural
	Gangwon-do	Chuncheon-si	13.9 (13.0,14.8)	22.4 (19.8,25.0)	15.4 (13.3,17.5)	11.7 (9.9,13.5)	11.3 (9.5,13.1)	9.2 (7.6,10.8)	13.2 (9.0,15.8)	Urban
	Gangwon-do	Wonju-si	14.6 (13.7,15.6)	21.7 (19.1,24.4)	17.4 (15.1,19.7)	11.5 (9.7,13.4)	12.7 (10.7,14.6)	10.0 (8.2,11.8)	11.7 (7.9,14.1)	Urban
	Gangwon-do	Gangneung-si	15.3 (14.4,16.2)	22.6 (20.0,25.2)	16.3 (14.2,18.3)	13.6 (11.7,15.4)	13.2 (11.3,15.1)	11.3 (9.5,13.1)	11.3 (9.3,15.5)	Urban
	Gangwon-do	Donghae-si	15.3 (14.3,16.2)	22.6 (20.0,25.2)	16.5 (14.4,18.6)	14.1 (12.1,16.1)	11.8 (10.0,13.6)	11.6 (9.7,13.4)	11.0 (8.6,14.8)	Urban
	Gangwon-do	Taebaek-si	20.4 (19.4,21.5)	28.4 (25.5,31.2)	22.4 (20.0,24.9)	19.9 (17.7,22.2)	15.9 (13.9,18.0)	15.6 (13.5,17.6)	12.8 (10.0,17.0)	Urban
	Gangwon-do	Sokcho-si	17.3 (16.3,18.3)	26.1 (23.3,28.9)	17.8 (15.6,20.0)	15.2 (13.1,17.2)	13.3 (11.4,15.2)	14.5 (12.4,16.6)	11.6 (8.0,14.6)	Urban
	Gangwon-do	Samcheok-si	17.5 (16.5,18.4)	27.7 (24.8,30.6)	18.3 (16.1,20.5)	16.5 (14.5,18.5)	13.5 (11.6,15.4)	11.9 (10.2,13.7)	15.8 (10.8,17.4)	Urban
	Gangwon-do	Hongcheon-gun	15.1 (14.2,16.0)	22.4 (19.9,24.9)	16.5 (14.4,18.5)	13.6 (11.8,15.4)	11.3 (9.7,13.0)	12.2 (10.4,14.0)	10.2 (7.9,14.5)	Rural
	Gangwon-do	Hoengseong-gun	17.4 (16.5,18.4)	23.7 (21.0,26.3)	18.8 (16.6,21.0)	17.0 (14.9,19.1)	15.1 (13.1,17.1)	12.6 (10.7,14.5)	11.1 (7.6,14.0)	Rural
	Gangwon-do	Yeongwol-gun	18.8 (17.8,19.8)	28.1 (25.1,31.0)	20.6 (18.3,22.9)	15.9 (13.9,17.8)	15.6 (13.7,17.6)	14.2 (12.3,16.1)	13.9 (10.3,17.1)	Rural
	Gangwon-do	Pyeongchang-gun	16.3 (15.3,17.2)	24.2 (21.6,26.8)	17.4 (15.4,19.5)	13.9 (12.1,15.8)	14.4 (12.5,16.3)	11.6 (9.8,13.3)	12.6 (7.9,14.3)	Rural
	Gangwon-do	Jeongseon-gun	19.9 (18.9,20.9)	27.4 (24.6,30.2)	20.7 (18.4,22.9)	20.0 (17.7,22.2)	18.0 (15.9,20.2)	13.7 (11.8,15.6)	13.7 (11.7,18.5)	Rural
	Gangwon-do	Cheorwon-gun	14.7 (13.8,15.6)	23.5 (20.9,26.1)	15.3 (13.4,17.3)	13.3 (11.4,15.1)	11.5 (9.7,13.2)	10.1 (8.4,11.7)	13.4 (11.3,17.7)	Rural
	Gangwon-do	Hwacheon-gun	15.6 (14.6,16.5)	22.5 (19.8,25.2)	17.0 (14.8,19.2)	13.6 (11.7,15.5)	13.5 (11.5,15.4)	11.6 (9.9,13.4)	10.9 (8.9,15.3)	Rural
	Gangwon-do	Yanggu-gun	16.1 (15.2,17.1)	22.9 (20.2,25.5)	16.8 (14.6,19.0)	14.7 (12.8,16.7)	13.2 (11.3,15.1)	13.3 (11.4,15.2)	9.6 (5.9,12.5)	Rural
	Gangwon-do	Inje-gun	15.6 (14.7,16.6)	23.1 (20.5,25.7)	15.5 (13.5,17.6)	14.6 (12.7,16.6)	13.1 (11.2,15.0)	11.6 (9.8,13.5)	11.5 (8.6,15.0)	Rural
	Gangwon-do	Goseong-gun	15.4 (14.5,16.3)	21.3 (18.7,23.8)	17.8 (15.5,20.1)	14.8 (12.8,16.8)	12.7 (10.9,14.5)	10.6 (8.9,12.3)	10.7 (7.9,14.1)	Rural
	Gangwon-do	Yangyang-gun	15.1 (14.2,16.0)	22.6 (20.0,25.2)	17.2 (15.1,19.3)	13.3 (11.4,15.2)	12.9 (11.1,14.7)	9.5 (8.0,11.0)	13.1 (9.7,15.9)	Rural
	Chungcheongbuk-do	Cheongju-si	13.8 (13.2,14.3)	20.7 (19.1,22.2)	15.4 (14.1,16.8)	12.1 (11.0,13.2)	11.2 (10.2,12.3)	10.2 (9.2,11.2)	10.5 (8.2,11.8)	Metropolitan
	Chungcheongbuk-do	Chungju-si	17.5 (16.5,18.5)	25.0 (22.3,27.7)	20.3 (18.0,22.6)	15.4 (13.4,17.5)	13.8 (11.9,15.7)	13.2 (11.3,15.2)	11.8 (9.1,15.5)	Urban
	Chungcheongbuk-do	Jecheon-si	15.1 (14.2,16.0)	21.9 (19.4,24.4)	15.2 (13.2,17.1)	14.6 (12.7,16.6)	12.6 (10.8,14.4)	11.5 (9.7,13.3)	10.4 (7.7,14.1)	Urban
	Chungcheongbuk-do	Boeun-gun	17.9 (17.0,18.9)	26.8 (23.9,29.8)	20.6 (18.3,23.0)	15.2 (13.4,17.1)	14.5 (12.5,16.4)	13.6 (11.7,15.5)	13.2 (10.6,17.4)	Rural
	Chungcheongbuk-do	Okcheon-gun	16.3 (15.4,17.2)	21.2 (18.8,23.7)	18.5 (16.4,20.7)	15.3 (13.4,17.3)	14.3 (12.4,16.1)	12.2 (10.3,14.0)	9.0 (5.4,11.8)	Rural
	Chungcheongbuk-do	Yeongdong-gun	16.3 (15.4,17.2)	24.9 (22.2,27.5)	16.9 (14.8,18.9)	14.3 (12.5,16.1)	13.1 (11.4,14.9)	12.5 (10.7,14.3)	12.4 (8.4,14.8)	Rural
	Chungcheongbuk-do	Jincheon-gun	16.8 (15.9,17.8)	23.8 (21.1,26.5)	15.1 (13.0,17.2)	16.8 (14.6,18.9)	13.5 (11.6,15.4)	15.2 (13.1,17.2)	8.6 (4.6,11.4)	Rural
	Chungcheongbuk-do	Goesan-gun	15.6 (14.7,16.5)	21.2 (18.7,23.6)	15.4 (13.4,17.5)	14.9 (12.9,16.9)	14.1 (12.1,16.0)	12.6 (10.8,14.5)	8.6 (6.7,12.9)	Rural
	Chungcheongbuk-do	Eumseong-gun	17.5 (16.5,18.4)	25.2 (22.4,28.0)	19.5 (17.1,21.8)	16.8 (14.8,18.8)	13.5 (11.7,15.2)	12.6 (10.8,14.4)	12.6 (9.8,16.4)	Rural
	Chungcheongbuk-do	Danyang-gun	17.7 (16.7,18.6)	26.9 (24.1,29.7)	16.6 (14.4,18.7)	15.7 (13.7,17.7)	14.3 (12.5,16.2)	15.4 (13.3,17.5)	11.5 (6.2,13.0)	Rural
	Chungcheongbuk-do	Jeungpyeong-gun	14.9 (14.1,15.8)	22.4 (19.9,24.9)	14.8 (13.0,16.6)	14.1 (12.3,15.8)	13.1 (11.3,14.8)	10.4 (8.8,12.0)	12.0 (9.3,15.5)	Rural
	Chungcheongnam-do	Cheonan-si	16.0 (14.9,17.0)	23.0 (20.0,26.0)	17.9 (15.4,20.4)	14.5 (12.3,16.8)	13.5 (11.3,15.7)	11.6 (9.6,13.7)	11.4 (8.3,14.7)	Metropolitan
	Chungcheongnam-do	Gongju-si	17.2 (16.2,18.1)	25.3 (22.6,28.0)	18.0 (15.8,20.1)	16.6 (14.5,18.6)	14.3 (12.4,16.1)	11.9 (10.1,13.7)	13.4 (10.3,16.7)	Urban
	Chungcheongnam-do	Boryeong-si	19.8 (18.8,20.8)	30.1 (27.1,33.1)	22.5 (20.0,24.9)	15.7 (13.8,17.6)	16.9 (14.8,19.0)	14.5 (12.5,16.4)	15.6 (11.9,18.9)	Urban
	Chungcheongnam-do	Asan-si	15.9 (14.9,16.9)	24.3 (21.4,27.2)	16.7 (14.4,18.9)	14.2 (12.1,16.3)	12.8 (10.8,14.8)	11.7 (9.8,13.6)	12.6 (9.3,15.7)	Urban
	Chungcheongnam-do	Seosan-si	16.0 (15.1,17.0)	21.8 (19.3,24.3)	17.2 (15.1,19.4)	14.5 (12.5,16.5)	14.3 (12.3,16.3)	12.5 (10.6,14.3)	9.3 (6.2,12.4)	Urban
	Chungcheongnam-do	Nonsan-si	17.1 (16.1,18.0)	26.1 (23.3,28.8)	17.6 (15.6,19.7)	15.2 (13.3,17.2)	14.1 (12.2,16.0)	12.2 (10.4,14.0)	13.9 (10.9,17.7)	Urban
	Chungcheongnam-do	Gyeryong-si	13.5 (12.5,14.5)	18.7 (16.0,21.4)	15.7 (13.3,18.1)	11.3 (9.2,13.4)	12.0 (9.9,14.1)	10.0 (8.0,11.9)	8.7 (4.7,10.7)	Urban
	Chungcheongnam-do	Dangjin-si	16.4 (15.4,17.3)	23.6 (21.0,26.2)	16.4 (14.3,18.5)	16.2 (14.1,18.4)	13.8 (11.9,15.7)	12.3 (10.4,14.1)	11.3 (6.3,12.7)	Rural
	Chungcheongnam-do	Geumsan-gun	16.8 (15.9,17.7)	22.9 (20.4,25.5)	19.7 (17.5,21.9)	15.2 (13.4,17.1)	13.9 (12.1,15.7)	12.1 (10.4,13.7)	10.8 (6.9,13.3)	Rural
	Chungcheongnam-do	Buyeo-gun	15.8 (14.9,16.7)	22.9 (20.3,25.6)	17.7 (15.6,19.8)	13.6 (11.9,15.3)	14.0 (12.2,15.9)	11.2 (9.6,12.9)	11.7 (7.6,13.8)	Rural
	Chungcheongnam-do	Seocheon-gun	17.0 (16.1,18.0)	25.2 (22.4,28.0)	17.0 (14.9,19.1)	15.2 (13.3,17.1)	15.1 (13.1,17.0)	13.1 (11.2,15.0)	12.1 (7.4,14.0)	Rural
	Chungcheongnam-do	Cheongyang-gun	15.2 (14.4,16.1)	22.8 (20.1,25.4)	16.6 (14.6,18.6)	15.3 (13.4,17.2)	11.9 (10.1,13.6)	9.9 (8.4,11.3)	12.9 (10.8,17.4)	Rural
	Chungcheongnam-do	Hongseong-gun	16.8 (15.9,17.7)	22.8 (20.3,25.4)	18.1 (15.9,20.2)	15.6 (13.6,17.5)	14.5 (12.6,16.5)	13.1 (11.3,14.9)	9.7 (8.0,14.4)	Rural
	Chungcheongnam-do	Yesan-gun	15.6 (14.8,16.5)	23.9 (21.3,26.6)	16.9 (14.8,18.9)	13.3 (11.5,15.0)	12.5 (10.8,14.2)	12.3 (10.5,14.1)	11.6 (9.0,15.6)	Rural
	Chungcheongnam-do	Taean-gun	15.9 (15.0,16.8)	20.8 (18.4,23.2)	17.9 (15.8,20.1)	15.6 (13.7,17.6)	13.4 (11.6,15.3)	11.7 (10.0,13.5)	9.1 (5.4,11.6)	Urban
	Jeollabuk-do	Jeonju-si	14.0 (13.1,15.0)	21.6 (19.0,24.3)	14.9 (12.8,17.1)	12.5 (10.5,14.4)	11.5 (9.7,13.4)	10.3 (8.5,12.1)	11.3 (9.9,16.7)	Metropolitan
	Jeollabuk-do	Gunsan-si	17.7 (16.7,18.7)	26.2 (23.4,29.0)	17.5 (15.3,19.7)	15.6 (13.5,17.7)	15.9 (13.8,18.1)	13.7 (11.8,15.7)	12.5 (10.6,18.6)	Urban
	Jeollabuk-do	Iksan-si	18.5 (17.4,19.5)	29.0 (26.1,31.9)	18.4 (16.2,20.7)	17.1 (14.9,19.2)	14.5 (12.6,16.5)	13.4 (11.5,15.3)	15.6 (12.9,19.7)	Urban
	Jeollabuk-do	Jeongeup-si	19.0 (18.0,20.0)	29.6 (26.6,32.5)	19.0 (16.8,21.2)	17.9 (15.8,20.0)	13.9 (12.0,15.7)	15.0 (13.0,17.0)	14.6 (10.5,17.3)	Urban
	Jeollabuk-do	Namwon-si	18.4 (17.5,19.4)	25.8 (23.1,28.5)	20.9 (18.6,23.2)	17.1 (15.0,19.2)	13.5 (11.8,15.3)	15.1 (13.2,17.0)	10.7 (10.5,18.1)	Urban
	Jeollabuk-do	Gimje-si	17.7 (16.7,18.7)	26.4 (23.6,29.2)	17.9 (15.8,20.0)	15.7 (13.7,17.6)	15.7 (13.7,17.7)	13.2 (11.3,15.1)	13.2 (6.3,14.7)	Urban
	Jeollabuk-do	Wanju-gun	16.7 (15.8,17.6)	24.3 (21.7,27.0)	19.4 (17.2,21.6)	15.4 (13.5,17.3)	12.2 (10.4,13.9)	12.5 (10.8,14.2)	11.8 (8.7,17.3)	Rural
	Jeollabuk-do	Jinan-gun	16.2 (15.2,17.1)	23.0 (20.3,25.8)	19.6 (17.3,21.9)	13.7 (12.0,15.4)	14.3 (12.4,16.2)	10.4 (8.8,12.0)	12.6 (11.8,21.6)	Rural
	Jeollabuk-do	Muju-gun	18.3 (17.3,19.3)	25.6 (22.8,28.3)	20.7 (18.3,23.0)	16.7 (14.7,18.7)	14.9 (13.0,16.9)	13.4 (11.5,15.3)	12.2 (7.9,14.7)	Rural
	Jeollabuk-do	Jangsu-gun	18.0 (17.0,19.0)	24.6 (21.8,27.4)	19.7 (17.4,21.9)	16.2 (14.3,18.2)	13.9 (12.1,15.8)	16.0 (13.9,18.0)	8.6 (5.7,13.5)	Rural
	Jeollabuk-do	Imsil-gun	17.7 (16.7,18.7)	24.2 (21.3,27.1)	17.2 (15.2,19.2)	22.2 (19.7,24.6)	13.8 (11.9,15.7)	11.0 (9.5,12.6)	13.2 (11.1,18.3)	Rural
	Jeollabuk-do	Sunchang-gun	17.7 (16.7,18.7)	25.6 (22.8,28.3)	21.3 (18.8,23.8)	15.6 (13.8,17.5)	13.9 (12.0,15.9)	12.0 (10.3,13.8)	13.6 (9.7,16.3)	Rural
	Jeollabuk-do	Gochang-gun	21.8 (20.7,22.9)	31.2 (28.0,34.3)	21.9 (19.6,24.2)	19.5 (17.2,21.7)	20.8 (18.4,23.2)	16.2 (14.2,18.3)	15.0 (11.3,18.3)	Rural
	Jeollabuk-do	Buan-gun	19.5 (18.5,20.5)	30.8 (27.7,33.9)	20.2 (17.9,22.5)	18.3 (16.1,20.5)	12.9 (11.2,14.7)	15.4 (13.3,17.4)	15.4 (11.7,19.1)	Rural
	Jeollanam-do	Mokpo-si	17.2 (16.2,18.2)	25.6 (22.8,28.4)	19.5 (17.1,21.9)	16.6 (14.4,18.7)	13.7 (11.7,15.6)	10.9 (9.1,12.7)	14.7 (8.9,15.3)	Urban

(continued to the next page)

Appendix 4. Continued

Gender	Provinces	Districts	Overall	Q1 (lowest)	Q2	Q3	Q4	Q5 (highest)	Q1-Q5	Urbanity
	Jeollanam-do	Yeosu-si	16.0 (15.1,16.9)	22.7 (20.1,25.2)	15.7 (13.7,17.7)	14.7 (12.7,16.7)	12.5 (10.6,14.3)	14.6 (12.6,16.6)	8.1 (4.8,11.2)	Urban
	Jeollanam-do	Suncheon-si	14.8 (13.9,15.8)	21.7 (19.1,24.3)	17.0 (14.9,19.2)	12.4 (10.5,14.3)	12.7 (10.8,14.6)	10.7 (9.0,12.4)	11.0 (10.1,16.3)	Urban
	Jeollanam-do	Naju-si	17.6 (16.6,18.5)	28.2 (25.4,31.1)	18.4 (16.3,20.6)	15.4 (13.5,17.4)	13.6 (11.8,15.3)	12.1 (10.4,13.7)	16.1 (13.6,20.2)	Urban
	Jeollanam-do	Gwangyang-si	14.3 (13.3,15.3)	21.3 (18.6,24.0)	15.6 (13.4,17.8)	13.0 (11.0,15.1)	11.2 (9.3,13.1)	10.7 (8.8,12.5)	10.6 (6.8,13.0)	Urban
	Jeollanam-do	Damyang-gun	17.4 (16.4,18.3)	25.6 (22.9,28.4)	18.8 (16.6,21.0)	15.2 (13.4,17.0)	13.9 (12.1,15.7)	13.6 (11.7,15.5)	12.0 (10.2,16.6)	Rural
	Jeollanam-do	Gokseong-gun	16.8 (15.9,17.7)	22.4 (19.8,25.0)	17.3 (15.3,19.3)	17.1 (15.0,19.2)	13.3 (11.6,15.0)	14.0 (12.1,15.9)	8.4 (5.9,12.5)	Rural
	Jeollanam-do	Gurye-gun	13.4 (12.6,14.2)	22.1 (19.6,24.7)	15.7 (13.7,17.7)	10.4 (9.0,11.9)	10.1 (8.6,11.6)	9.1 (7.6,10.7)	13.0 (10.3,16.5)	Rural
	Jeollanam-do	Goheung-gun	15.6 (14.7,16.5)	28.3 (25.1,31.6)	15.4 (13.5,17.3)	14.8 (12.9,16.6)	11.8 (10.2,13.5)	8.2 (7.0,9.5)	20.1 (18.0,24.0)	Rural
	Jeollanam-do	Boseong-gun	18.2 (17.2,19.1)	27.8 (24.7,30.8)	21.0 (18.6,23.3)	17.3 (15.2,19.3)	13.7 (11.9,15.5)	11.2 (9.8,12.7)	16.6 (14.1,20.9)	Rural
	Jeollanam-do	Hwasun-gun	18.9 (17.9,19.9)	29.2 (26.3,32.1)	18.4 (16.3,20.5)	18.1 (16.0,20.3)	14.3 (12.5,16.2)	14.7 (12.8,16.7)	14.5 (11.0,18.0)	Rural
	Jeollanam-do	Jangheung-gun	15.9 (15.0,16.8)	26.4 (23.5,29.3)	16.0 (14.1,18.0)	13.7 (11.8,15.5)	11.3 (9.8,12.8)	12.7 (10.9,14.6)	13.7 (9.8,18.0)	Rural
	Jeollanam-do	Gangjin-gun	14.2 (13.3,15.1)	21.7 (19.1,24.3)	15.1 (13.1,17.0)	12.6 (10.8,14.4)	12.2 (10.4,13.9)	9.8 (8.2,11.3)	11.9 (10.8,18.2)	Rural
	Jeollanam-do	Haenam-gun	17.9 (16.9,18.8)	25.8 (23.0,28.7)	17.8 (15.7,19.8)	16.4 (14.5,18.3)	15.4 (13.4,17.4)	14.6 (12.7,16.5)	11.2 (8.0,15.4)	Rural
	Jeollanam-do	Yeongam-gun	17.5 (16.6,18.5)	25.9 (23.2,28.6)	19.2 (17.0,21.4)	16.1 (14.1,18.1)	15.0 (13.0,16.9)	11.7 (10.1,13.4)	14.2 (10.8,17.2)	Rural
	Jeollanam-do	Muan-gun	15.2 (14.3,16.1)	20.3 (17.9,22.7)	15.7 (13.7,17.6)	16.0 (14.0,18.0)	11.8 (10.2,13.5)	12.5 (10.6,14.3)	7.8 (6.0,12.2)	Rural
	Jeollanam-do	Hampyeong-gun	19.2 (18.2,20.2)	28.6 (25.6,31.6)	19.7 (17.5,22.0)	17.1 (15.0,19.3)	16.3 (14.3,18.4)	14.3 (12.3,16.2)	14.3 (11.6,18.4)	Rural
	Jeollanam-do	Yeonggwang-gun	19.3 (18.3,20.3)	29.0 (26.1,31.8)	21.7 (19.4,24.0)	17.7 (15.6,19.8)	15.1 (13.3,16.9)	13.4 (11.6,15.3)	15.6 (13.8,20.6)	Rural
	Jeollanam-do	Jangseong-gun	17.2 (16.3,18.2)	26.0 (23.2,28.8)	18.2 (16.1,20.4)	15.0 (13.1,16.9)	15.0 (13.1,16.9)	12.3 (10.6,14.1)	13.7 (6.3,12.9)	Rural
	Jeollanam-do	Wando-gun	17.4 (16.5,18.4)	27.4 (24.5,30.2)	19.3 (17.2,21.4)	14.3 (12.5,16.2)	14.8 (13.0,16.6)	11.4 (9.7,13.0)	16.0 (13.8,20.2)	Rural
	Jeollanam-do	Jindo-gun	18.5 (17.6,19.5)	28.4 (25.4,31.4)	20.1 (17.8,22.3)	16.7 (14.8,18.7)	14.6 (12.8,16.4)	13.0 (11.2,14.9)	15.4 (12.7,19.3)	Rural
	Jeollanam-do	Sinan-gun	19.8 (18.7,20.8)	25.9 (23.2,28.6)	23.3 (20.8,25.9)	17.2 (15.1,19.3)	17.5 (15.4,19.5)	14.7 (12.7,16.6)	11.2 (11.8,19.4)	Rural
	Gyeongsangbuk-do	Nam-gu	17.1 (16.1,18.1)	26.5 (23.7,29.3)	17.7 (15.4,19.9)	15.9 (13.7,18.1)	12.6 (10.7,14.6)	12.8 (10.8,14.7)	13.7 (10.1,16.7)	Metropolitan
	Gyeongsangbuk-do	Buk-gu	15.5 (14.6,16.5)	23.3 (20.6,26.0)	15.7 (13.6,17.8)	14.2 (12.2,16.2)	12.7 (10.8,14.7)	11.8 (9.9,13.7)	11.5 (8.1,14.5)	Metropolitan
	Gyeongsangbuk-do	Gyeongju-si	16.9 (16.0,17.9)	24.4 (21.8,27.0)	17.8 (15.6,20.0)	16.3 (14.2,18.3)	13.9 (12.1,15.8)	12.7 (10.9,14.5)	11.7 (8.5,14.9)	Urban
	Gyeongsangbuk-do	Gimcheon-si	16.2 (15.3,17.2)	22.4 (19.9,24.8)	17.3 (15.3,19.4)	16.1 (14.0,18.1)	12.7 (10.9,14.6)	12.5 (10.6,14.3)	9.9 (6.2,12.6)	Urban
	Gyeongsangbuk-do	Andong-si	16.9 (15.9,17.8)	25.6 (22.9,28.3)	18.7 (16.5,20.9)	15.8 (13.8,17.7)	12.6 (10.8,14.4)	12.2 (10.5,14.0)	13.4 (8.9,15.3)	Urban
	Gyeongsangbuk-do	Gumi-si	17.1 (16.2,17.9)	25.7 (23.3,28.0)	18.2 (16.2,20.1)	15.2 (13.4,17.0)	14.3 (12.6,16.1)	13.3 (11.8,14.9)	12.4 (8.5,13.1)	Urban
	Gyeongsangbuk-do	Yeongju-si	17.5 (16.6,18.5)	26.5 (23.7,29.3)	18.0 (15.9,20.1)	16.9 (14.9,19.0)	14.2 (12.3,16.0)	12.7 (10.9,14.4)	13.8 (9.9,16.5)	Urban
	Gyeongsangbuk-do	Yeongcheon-si	19.3 (18.3,20.3)	25.4 (22.7,28.1)	22.3 (19.9,24.6)	17.6 (15.5,19.7)	16.3 (14.3,18.4)	15.0 (13.0,17.0)	10.4 (6.7,13.5)	Urban
	Gyeongsangbuk-do	Sangju-si	17.1 (16.2,18.0)	24.9 (22.3,27.6)	16.7 (14.7,18.6)	15.9 (13.9,17.8)	14.7 (12.8,16.5)	13.3 (11.5,15.2)	11.6 (7.5,13.9)	Urban
	Gyeongsangbuk-do	Mungyeong-si	17.5 (16.6,18.5)	23.7 (21.2,26.3)	17.3 (15.3,19.4)	17.9 (15.8,19.9)	15.5 (13.6,17.5)	13.4 (11.5,15.2)	10.3 (5.3,12.7)	Urban
	Gyeongsangbuk-do	Gyeongsan-si	17.1 (16.1,18.1)	24.2 (21.5,27.0)	18.2 (15.9,20.4)	14.1 (12.1,16.1)	15.8 (13.7,18.0)	13.5 (11.5,15.4)	10.7 (6.4,12.8)	Urban
	Gyeongsangbuk-do	Gunwi-gun	17.1 (16.1,18.0)	24.4 (21.6,27.3)	18.1 (16.1,20.2)	15.6 (13.7,17.5)	14.8 (12.8,16.7)	12.9 (11.1,14.7)	11.5 (5.9,12.7)	Rural
	Gyeongsangbuk-do	Uiseong-gun	16.4 (15.4,17.3)	22.2 (19.6,24.9)	17.1 (15.0,19.1)	14.2 (12.4,16.1)	15.3 (13.2,17.3)	13.3 (11.4,15.3)	8.9 (4.4,10.8)	Rural
	Gyeongsangbuk-do	Cheongsong-gun	18.9 (17.9,19.9)	28.0 (24.9,31.0)	19.1 (17.0,21.2)	16.5 (14.5,18.5)	15.5 (13.6,17.5)	15.6 (13.5,17.7)	12.4 (9.6,16.4)	Rural
	Gyeongsangbuk-do	Yeongyang-gun	20.6 (19.5,21.6)	29.5 (26.4,32.5)	22.9 (20.4,25.4)	19.1 (16.8,21.3)	17.1 (15.0,19.2)	14.6 (12.7,16.6)	14.9 (11.2,18.2)	Rural
	Gyeongsangbuk-do	Yeongdeok-gun	17.6 (16.6,18.6)	27.9 (24.8,31.0)	20.8 (18.4,23.3)	14.8 (13.0,16.6)	13.0 (11.2,14.8)	11.4 (9.7,13.1)	16.5 (11.9,18.3)	Rural
	Gyeongsangbuk-do	Cheongdo-gun	18.2 (17.3,19.2)	25.3 (22.5,28.0)	17.6 (15.5,19.7)	16.0 (14.1,17.9)	16.9 (14.8,19.0)	15.7 (13.7,17.8)	9.6 (4.4,11.2)	Rural
	Gyeongsangbuk-do	Goryeong-gun	17.9 (16.9,18.8)	23.8 (21.1,26.4)	17.4 (15.3,19.4)	17.2 (15.1,19.4)	15.3 (13.3,17.2)	15.7 (13.6,17.8)	8.1 (4.1,10.9)	Rural
	Gyeongsangbuk-do	Seongju-gun	12.1 (11.3,12.9)	19.8 (17.4,22.2)	13.0 (11.2,14.8)	9.9 (8.3,11.4)	9.0 (7.5,10.4)	8.9 (7.4,10.4)	10.9 (7.5,13.3)	Rural
	Gyeongsangbuk-do	Chilgok-gun	17.1 (16.1,18.1)	24.3 (21.5,27.2)	17.4 (15.0,19.8)	17.2 (14.9,19.5)	13.1 (11.1,15.1)	13.9 (11.9,16.0)	10.4 (6.5,13.1)	Rural
	Gyeongsangbuk-do	Yecheon-gun	18.4 (17.3,19.4)	30.1 (26.9,33.3)	18.6 (16.4,20.7)	17.7 (15.4,19.9)	13.8 (12.0,15.6)	11.8 (10.1,13.5)	18.3 (11.4,18.2)	Rural
	Gyeongsangbuk-do	Bonghwa-gun	19.7 (18.7,20.8)	28.0 (25.0,31.0)	19.8 (17.5,22.1)	19.6 (17.3,21.9)	17.0 (15.0,19.1)	14.4 (12.4,16.4)	13.6 (9.4,16.4)	Rural
	Gyeongsangbuk-do	Uljin-gun	18.3 (17.3,19.3)	27.2 (24.4,30.0)	19.6 (17.5,21.8)	16.4 (14.4,18.3)	15.5 (13.5,17.5)	13.3 (11.5,15.0)	13.9 (10.3,17.1)	Rural
	Gyeongsangbuk-do	Ulleung-gun	15.7 (14.8,16.7)	22.6 (19.9,25.2)	17.7 (15.5,19.9)	14.3 (12.3,16.4)	12.3 (10.4,14.1)	11.7 (9.8,13.6)	10.9 (8.8,15.6)	Rural
	Gyeongsangnam-do	Changwon-si	17.5 (16.3,18.7)	23.3 (20.1,26.4)	18.6 (15.9,21.4)	16.9 (14.4,19.4)	15.9 (13.4,18.4)	13.2 (10.9,15.5)	10.1 (6.7,13.3)	Metropolitan
	Gyeongsangnam-do	Masan-si	14.6 (13.7,15.5)	22.1 (19.6,24.7)	14.0 (12.0,15.9)	13.4 (11.4,15.3)	13.7 (11.7,15.7)	10.1 (8.4,11.8)	12.0 (7.4,13.6)	Metropolitan
	Gyeongsangnam-do	Jinju-si	15.7 (14.7,16.6)	24.1 (21.4,26.7)	15.1 (13.0,17.1)	15.1 (13.0,17.1)	13.0 (11.1,14.9)	11.4 (9.6,13.2)	12.7 (8.8,15.2)	Urban
	Gyeongsangnam-do	Jinhae-si	15.8 (14.8,16.8)	22.0 (19.3,24.8)	16.8 (14.5,19.0)	14.9 (12.7,17.1)	13.4 (11.4,15.5)	12.1 (10.2,14.1)	9.9 (6.4,12.8)	Metropolitan
	Gyeongsangnam-do	Tongyeong-si	19.5 (18.5,20.6)	28.1 (25.2,31.0)	20.8 (18.4,23.2)	17.7 (15.5,20.0)	15.3 (13.3,17.4)	16.0 (13.9,18.2)	12.1 (8.5,15.3)	Urban
	Gyeongsangnam-do	Sacheon-si	16.8 (15.9,17.8)	25.7 (22.9,28.4)	18.6 (16.4,20.9)	13.7 (11.8,15.5)	13.6 (11.8,15.5)	12.9 (11.1,14.8)	12.8 (8.5,15.1)	Urban
	Gyeongsangnam-do	Gimhae-si	16.6 (15.5,17.7)	23.1 (20.1,26.1)	16.6 (14.3,19.0)	15.2 (12.9,17.5)	13.8 (11.6,16.0)	15.0 (12.7,17.2)	8.1 (4.3,10.9)	Urban
	Gyeongsangnam-do	Miryang-si	17.4 (16.5,18.4)	26.6 (23.9,29.2)	19.0 (16.8,21.2)	16.8 (14.8,18.8)	12.9 (11.2,14.6)	12.1 (10.4,13.8)	14.5 (12.7,19.1)	Urban
	Gyeongsangnam-do	Geoje-si	16.4 (15.3,17.5)	21.0 (18.3,23.8)	17.3 (14.8,19.8)	15.8 (13.4,18.2)	13.9 (11.7,16.1)	14.1 (11.9,16.4)	6.9 (3.3,9.7)	Urban
	Gyeongsangnam-do	Yangsan-si	13.6 (12.7,14.6)	20.7 (18.1,23.2)	14.1 (11.9,16.3)	11.9 (10.0,13.8)	11.0 (9.1,12.8)	10.9 (9.0,12.7)	9.8 (5.1,11.3)	Urban
	Gyeongsangnam-do	Uiryeong-gun	17.5 (16.5,18.5)	23.1 (20.4,25.8)	19.4 (17.1,21.7)	15.0 (13.1,16.8)	15.9 (13.8,18.0)	14.6 (12.5,16.6)	8.5 (7.6,14.8)	Rural
	Gyeongsangnam-do	Haman-gun	15.8 (14.9,16.7)	22.2 (19.7,24.6)	15.9 (13.9,17.9)	15.0 (13.2,16.9)	12.9 (11.1,14.6)	13.1 (11.3,14.9)	9.1 (6.3,12.9)	Rural
	Gyeongsangnam-do	Changnyeong-gun	18.4 (17.5,19.4)	24.8 (22.1,27.5)	19.7 (17.5,21.9)	16.7 (14.7,18.7)	15.4 (13.5,17.3)	15.8 (13.7,17.8)	9.0 (5.2,12.0)	Rural
	Gyeongsangnam-do	Goseong-gun	15.9 (15.0,16.8)	22.8 (20.2,25.4)	17.0 (15.0,19.1)	15.1 (13.2,17.0)	13.7 (11.8,15.5)	11.1 (9.5,12.8)	11.7 (8.0,14.2)	Rural
	Gyeongsangnam-do	Namhae-gun	21.6 (20.5,22.7)	31.0 (27.8,34.2)	24.2 (21.6,26.8)	19.9 (17.6,22.2)	18.1 (15.9,20.3)	15.3 (13.2,17.4)	15.7 (11.7,18.9)	Rural

(continued to the next page)

Appendix 4. Continued

Gender	Provinces	Districts	Overall	Q1 (lowest)	Q2	Q3	Q4	Q5 (highest)	Q1-Q5	Urbanity
	Gyeongsangnam-do	Hadong-gun	21.2 (20.1,22.3)	28.8 (25.8,31.8)	22.7 (20.3,25.2)	20.4 (18.1,22.7)	17.3 (15.2,19.3)	17.2 (15.0,19.4)	11.6 (5.5,12.7)	Rural
	Gyeongsangnam-do	Sancheong-gun	19.4 (18.3,20.4)	27.9 (24.9,30.9)	21.2 (18.9,23.6)	17.1 (15.1,19.0)	16.7 (14.6,18.7)	14.2 (12.3,16.2)	13.7 (10.2,17.0)	Rural
	Gyeongsangnam-do	Hamyang-gun	19.2 (18.2,20.2)	23.5 (20.9,26.2)	22.3 (19.8,24.8)	18.0 (15.8,20.1)	17.1 (15.0,19.2)	15.3 (13.3,17.3)	8.2 (5.5,13.7)	Rural
	Gyeongsangnam-do	Geochang-gun	17.0 (16.0,17.9)	25.5 (22.8,28.1)	17.3 (15.3,19.4)	16.3 (14.3,18.4)	13.6 (11.8,15.4)	12.3 (10.5,14.0)	13.2 (8.4,15.0)	Rural
	Gyeongsangnam-do	Hapcheon-gun	19.2 (18.1,20.2)	26.5 (23.6,29.5)	21.8 (19.3,24.3)	18.6 (16.5,20.7)	14.8 (13.0,16.7)	14.7 (12.7,16.7)	11.8 (5.9,13.3)	Rural
	Jeju-do	Jeju-si	15.7 (15.1,16.2)	24.4 (22.7,26.0)	17.6 (16.2,19.1)	13.7 (12.5,15.0)	11.7 (10.6,12.8)	11.6 (10.6,12.7)	12.8 (10.9,14.7)	Urban
	Jeju-do	Seogwipo-si	16.0 (15.5,16.6)	23.2 (21.6,24.8)	16.7 (15.4,18.0)	14.5 (13.3,15.7)	13.2 (12.1,14.3)	13.0 (11.9,14.2)	10.2 (7.0,11.0)	Urban
Men	Seoul	Jongno-gu	10.0 (8.9,11.2)	18.1 (14.6,21.5)	10.7 (8.1,13.4)	9.6 (7.0,12.1)	6.4 (4.4,8.5)	5.7 (3.8,7.6)	12.4 (8.5,16.5)	Metropolitan
	Seoul	Jung-gu	11.1 (9.9,12.3)	20.1 (16.3,23.8)	11.2 (8.5,13.9)	9.7 (7.2,12.2)	8.6 (6.3,10.9)	6.3 (4.3,8.3)	13.8 (8.8,17.4)	Metropolitan
	Seoul	Yongsan-gu	9.5 (8.3,10.7)	16.8 (13.3,20.2)	6.8 (4.6,8.9)	9.1 (6.6,11.6)	7.9 (5.5,10.3)	6.7 (4.6,8.9)	10.1 (8.1,16.7)	Metropolitan
	Seoul	Seongdong-gu	10.7 (9.4,12.0)	12.7 (9.6,15.8)	10.6 (7.9,13.2)	11.6 (8.5,14.7)	10.3 (7.7,13.0)	8.2 (5.7,10.6)	4.5 (-0.1,7.3)	Metropolitan
	Seoul	Gwangjin-gu	9.9 (8.6,11.1)	13.0 (9.7,16.2)	12.1 (9.1,15.1)	9.3 (6.5,12.0)	8.8 (6.2,11.4)	6.3 (4.0,8.5)	6.7 (5.9,12.9)	Metropolitan
	Seoul	Dongdaemun-gu	11.0 (9.8,12.3)	17.4 (13.8,20.9)	11.9 (9.1,14.7)	9.0 (6.5,11.5)	8.1 (5.7,10.6)	8.7 (6.1,11.3)	8.7 (3.7,11.9)	Metropolitan
	Seoul	Jungnang-gu	11.1 (9.9,12.4)	18.8 (15.0,22.5)	13.2 (10.0,16.3)	8.6 (6.1,11.1)	9.9 (7.2,12.7)	5.5 (3.3,7.6)	13.3 (10.3,18.7)	Metropolitan
	Seoul	Seongbuk-gu	10.4 (9.2,11.6)	19.0 (15.3,22.7)	9.8 (7.2,12.4)	7.3 (5.1,9.5)	9.0 (6.4,11.6)	7.5 (5.2,9.8)	11.5 (8.1,16.7)	Metropolitan
	Seoul	Gangbuk-gu	12.0 (10.8,13.3)	17.7 (14.2,21.3)	13.8 (10.7,16.8)	12.4 (9.5,15.3)	8.4 (6.0,10.7)	7.7 (5.4,10.0)	10.0 (6.2,15.0)	Metropolitan
	Seoul	Dobong-gu	11.5 (10.2,12.8)	18.4 (14.8,22.1)	11.2 (8.4,14.1)	11.7 (8.8,14.5)	9.0 (6.5,11.4)	7.7 (5.3,10.0)	10.7 (6.7,15.7)	Metropolitan
	Seoul	Nowon-gu	11.3 (9.9,12.6)	21.2 (17.1,25.4)	11.5 (8.6,14.3)	10.6 (7.6,13.6)	7.9 (5.4,10.5)	5.4 (3.4,7.5)	15.8 (9.6,18.0)	Metropolitan
	Seoul	Eunpyeong-gu	12.0 (10.7,13.3)	18.8 (15.1,22.6)	13.4 (10.3,16.4)	11.4 (8.6,14.1)	10.3 (7.6,13.0)	6.0 (3.9,8.1)	12.8 (7.8,16.2)	Metropolitan
	Seoul	Seodaemun-gu	11.2 (9.9,12.4)	16.6 (13.2,20.1)	12.9 (9.9,15.9)	10.6 (7.8,13.4)	9.6 (7.1,12.1)	6.1 (4.0,8.2)	10.5 (4.9,13.1)	Metropolitan
	Seoul	Mapo-gu	9.3 (8.1,10.5)	13.7 (10.4,17.0)	11.8 (8.8,14.9)	10.2 (7.5,13.0)	6.3 (4.1,8.4)	4.9 (3.0,6.9)	8.8 (2.6,10.8)	Metropolitan
	Seoul	Yangcheon-gu	11.0 (9.6,12.3)	20.6 (16.4,24.8)	12.3 (9.3,15.3)	8.0 (5.3,10.6)	7.2 (4.7,9.7)	7.2 (4.7,9.7)	13.4 (6.6,15.4)	Metropolitan
	Seoul	Gangseo-gu	12.4 (11.0,13.8)	20.4 (16.3,24.5)	14.0 (10.8,17.2)	10.5 (7.6,13.4)	9.9 (7.1,12.7)	7.5 (5.1,10.0)	12.9 (5.1,13.5)	Metropolitan
	Seoul	Guro-gu	11.9 (10.5,13.2)	21.6 (17.6,25.7)	9.9 (7.2,12.6)	11.0 (8.1,13.9)	9.3 (6.7,11.8)	7.7 (5.2,10.2)	13.9 (9.1,17.7)	Metropolitan
	Seoul	Geumcheon-gu	11.6 (10.3,12.9)	18.0 (14.4,21.6)	11.4 (8.6,14.2)	11.5 (8.7,14.3)	7.7 (5.4,10.0)	9.7 (7.1,12.4)	8.3 (2.7,11.1)	Metropolitan
	Seoul	Yeongdeungpo-gu	11.9 (10.6,13.2)	19.3 (15.6,23.1)	12.0 (9.0,14.9)	11.0 (8.2,13.8)	8.9 (6.4,11.5)	8.3 (5.8,10.8)	11.0 (5.9,14.3)	Metropolitan
	Seoul	Dongjak-gu	11.6 (10.3,12.9)	17.4 (13.7,21.0)	11.1 (8.2,14.0)	10.1 (7.5,12.7)	10.4 (7.7,13.1)	9.4 (6.8,12.1)	8.0 (2.0,10.4)	Metropolitan
	Seoul	Gwanak-gu	11.7 (10.3,13.0)	16.3 (12.7,19.9)	13.4 (10.1,16.7)	11.1 (8.3,13.9)	8.4 (5.8,11.0)	9.8 (6.9,12.6)	6.5 (5.0,13.2)	Metropolitan
	Seoul	Seocho-gu	7.5 (6.4,8.6)	10.9 (8.0,13.9)	9.2 (6.5,11.8)	7.0 (4.7,9.3)	5.1 (3.2,7.1)	5.3 (3.1,7.5)	5.6 (4.2,11.2)	Metropolitan
	Seoul	Gangnam-gu	7.8 (6.6,8.9)	11.6 (8.3,15.0)	7.4 (4.8,9.9)	7.7 (5.2,10.2)	6.5 (4.1,8.9)	5.9 (3.5,8.3)	5.7 (3.0,10.6)	Metropolitan
	Seoul	Songpa-gu	7.8 (6.6,8.9)	12.9 (9.6,16.3)	8.0 (5.3,10.7)	6.8 (4.3,9.2)	7.2 (4.8,9.6)	4.0 (2.2,5.7)	8.9 (5.9,13.1)	Metropolitan
	Seoul	Gangdong-gu	11.5 (10.1,12.9)	17.1 (13.4,20.8)	13.5 (10.4,16.7)	9.8 (6.9,12.6)	8.8 (6.1,11.5)	8.2 (5.4,11.0)	8.9 (5.9,14.3)	Metropolitan
	Busan	Jung-gu	12.8 (11.5,14.1)	21.5 (17.7,25.4)	13.3 (10.5,16.2)	13.2 (10.2,16.1)	8.8 (6.4,11.1)	7.7 (5.5,9.9)	13.8 (10.0,19.0)	Metropolitan
	Busan	Seo-gu	13.5 (12.2,14.8)	23.0 (19.1,26.8)	14.5 (11.5,17.4)	10.2 (7.7,12.7)	9.8 (7.4,12.3)	10.0 (7.5,12.5)	13.0 (7.6,16.8)	Metropolitan
	Busan	Dong-gu	16.0 (14.6,17.5)	26.7 (22.4,30.9)	15.3 (12.1,18.5)	13.2 (10.3,16.1)	13.1 (10.2,16.0)	12.2 (9.4,15.1)	14.5 (8.8,18.6)	Metropolitan
	Busan	Yeongdo-gu	16.1 (14.7,17.6)	27.8 (23.5,32.1)	17.3 (14.1,20.5)	13.6 (10.6,16.5)	11.6 (9.0,14.2)	11.1 (8.5,13.7)	16.7 (10.4,20.2)	Metropolitan
	Busan	Busanjin-gu	11.9 (10.6,13.1)	20.0 (16.3,23.7)	14.2 (11.2,17.3)	10.4 (7.6,13.2)	8.6 (6.1,11.0)	5.8 (3.8,7.9)	14.2 (9.6,17.8)	Metropolitan
	Busan	Dongnae-gu	11.3 (10.0,12.5)	15.8 (12.6,19.0)	11.6 (8.6,14.5)	9.7 (7.2,12.3)	9.3 (6.8,11.8)	9.7 (7.0,12.3)	6.1 (4.7,12.7)	Metropolitan
	Busan	Nam-gu	11.2 (9.9,12.4)	17.0 (13.6,20.4)	11.8 (9.0,14.6)	10.2 (7.5,12.8)	9.0 (6.6,11.5)	7.9 (5.5,10.3)	9.1 (6.7,14.7)	Metropolitan
	Busan	Buk-gu	12.1 (10.7,13.5)	20.8 (16.9,24.8)	10.6 (7.5,13.6)	10.9 (8.0,13.7)	8.2 (5.7,10.7)	10.1 (7.1,13.0)	10.7 (9.0,17.8)	Metropolitan
	Busan	Haeundae-gu	11.8 (10.5,13.1)	24.7 (20.4,29.0)	10.9 (8.1,13.7)	10.1 (7.4,12.8)	8.6 (6.1,11.2)	5.3 (3.2,7.3)	19.4 (13.6,22.2)	Metropolitan
	Busan	Saha-gu	12.7 (11.4,14.1)	21.5 (17.5,25.5)	12.7 (9.6,15.8)	9.7 (7.1,12.4)	9.8 (7.1,12.5)	10.1 (7.3,13.0)	11.4 (6.7,15.7)	Metropolitan
	Busan	Geumjeong-gu	10.9 (9.6,12.1)	20.0 (16.2,23.9)	11.5 (8.6,14.3)	8.4 (6.1,10.7)	7.4 (5.2,9.7)	7.9 (5.5,10.3)	12.1 (10.2,18.4)	Metropolitan
	Busan	Gangseo-gu	14.0 (12.7,15.3)	21.1 (17.4,24.8)	13.4 (10.5,16.2)	13.7 (10.7,16.6)	11.4 (8.7,14.2)	10.5 (7.9,13.2)	10.6 (4.5,13.5)	Metropolitan
	Busan	Yeonje-gu	12.5 (11.1,13.8)	21.7 (17.8,25.6)	11.1 (8.4,13.8)	10.3 (7.6,13.0)	8.5 (6.2,10.9)	11.1 (8.2,14.0)	10.6 (5.2,14.2)	Metropolitan
	Busan	Suyeong-gu	10.9 (9.8,12.1)	17.5 (14.1,20.8)	11.1 (8.4,13.9)	8.2 (6.0,10.5)	10.2 (7.6,12.8)	7.8 (5.4,10.1)	9.7 (5.9,14.3)	Metropolitan
	Busan	Sasang-gu	13.5 (12.0,14.9)	18.8 (15.1,22.6)	12.2 (9.2,15.3)	12.5 (9.4,15.6)	12.2 (9.2,15.2)	11.9 (8.8,15.0)	6.9 (1.0,9.8)	Metropolitan
	Busan	Gijang-gun	15.0 (13.5,16.4)	21.3 (17.4,25.1)	15.7 (12.3,19.1)	15.2 (12.0,18.3)	13.9 (10.8,17.0)	9.2 (6.6,11.7)	12.1 (5.2,14.2)	Rural
	Daegu	Jung-gu	15.8 (14.4,17.3)	24.0 (19.8,28.1)	17.7 (14.5,21.0)	14.8 (11.7,18.0)	12.0 (9.2,14.7)	10.8 (8.0,13.5)	13.2 (7.1,16.3)	Metropolitan
	Daegu	Dong-gu	15.7 (14.3,17.2)	22.8 (18.7,26.9)	18.2 (14.8,21.6)	13.9 (10.9,16.8)	13.5 (10.5,16.6)	10.6 (7.9,13.3)	12.2 (7.9,16.9)	Metropolitan
	Daegu	Seo-gu	15.1 (13.7,16.6)	21.0 (17.1,24.8)	16.6 (13.0,20.1)	13.6 (10.5,16.6)	14.0 (10.8,17.2)	10.4 (7.6,13.1)	10.6 (7.0,15.6)	Metropolitan
	Daegu	Nam-gu	13.1 (11.7,14.4)	21.1 (17.2,25.0)	13.7 (10.7,16.8)	11.6 (8.8,14.4)	10.4 (7.8,12.9)	9.0 (6.5,11.5)	12.1 (7.7,16.5)	Metropolitan
	Daegu	Buk-gu	12.5 (11.2,13.9)	20.8 (16.7,24.9)	13.6 (10.3,16.8)	11.8 (8.9,14.7)	8.7 (6.2,11.3)	8.4 (5.8,10.9)	12.4 (10.1,18.5)	Metropolitan
	Daegu	Suseong-gu	11.7 (10.4,13.0)	19.1 (15.4,22.8)	12.1 (9.2,15.1)	11.0 (8.3,13.7)	9.0 (6.5,11.6)	7.3 (5.0,9.7)	11.8 (6.1,14.3)	Metropolitan
	Daegu	Dalseo-gu	13.1 (11.7,14.5)	21.7 (17.6,25.7)	16.0 (12.3,19.7)	10.1 (7.4,12.9)	9.4 (6.7,12.2)	8.4 (5.8,10.9)	13.3 (7.7,16.5)	Metropolitan
	Daegu	Dalseong-gun	14.2 (12.8,15.6)	18.1 (14.4,21.7)	16.1 (12.6,19.6)	14.7 (11.6,17.8)	12.2 (9.1,15.2)	9.9 (7.2,12.7)	8.2 (7.9,16.3)	Rural
	Incheon	Jung-gu	12.2 (10.9,13.5)	18.3 (14.7,22.0)	13.5 (10.6,16.4)	12.0 (9.1,14.9)	9.6 (7.1,12.2)	7.7 (5.4,10.1)	10.6 (5.7,14.1)	Metropolitan
	Incheon	Dong-gu	12.1 (10.9,13.4)	18.6 (15.0,22.2)	12.9 (9.9,16.0)	13.2 (10.3,16.0)	8.2 (5.9,10.5)	8.2 (5.8,10.5)	10.4 (5.9,14.1)	Metropolitan
	Incheon	Nam-gu	10.3 (9.1,11.5)	17.6 (13.9,21.2)	8.7 (6.1,11.2)	8.7 (6.3,11.1)	8.4 (5.9,10.8)	8.7 (6.2,11.2)	8.9 (2.4,10.6)	Metropolitan
	Incheon	Yeonsu-gu	11.9 (10.4,13.4)	15.8 (12.0,19.7)	12.4 (8.9,15.9)	10.5 (7.4,13.5)	10.0 (6.9,13.1)	11.0 (7.6,14.3)	4.8 (0.4,9.2)	Metropolitan

(continued to the next page)

Appendix 4. Continued

Gender	Provinces	Districts	Overall	Q1 (lowest)	Q2	Q3	Q4	Q5 (highest)	Q1-Q5	Urbanity
	Incheon	Namdong-gu	12.2 (10.7,13.7)	18.9 (14.8,23.1)	12.3 (8.8,15.7)	11.7 (8.4,14.9)	7.5 (4.9,10.1)	10.7 (7.6,13.8)	8.2 (7.8,16.8)	Metropolitan
	Incheon	Bupyeong-gu	14.3 (12.8,15.9)	24.2 (19.7,28.8)	13.8 (10.4,17.2)	13.7 (10.5,17.0)	10.7 (7.7,13.6)	9.8 (6.8,12.7)	14.4 (10.1,19.5)	Metropolitan
	Incheon	Gyeyang-gu	14.1 (12.4,15.8)	21.2 (16.5,25.9)	14.6 (10.7,18.6)	13.4 (9.9,16.9)	11.1 (7.7,14.5)	10.8 (7.4,14.1)	10.4 (5.2,14.8)	Metropolitan
	Incheon	Seo-gu	12.8 (11.3,14.4)	18.8 (14.5,23.0)	11.4 (8.2,14.5)	12.1 (8.8,15.4)	11.6 (8.3,14.9)	10.5 (7.4,13.6)	8.3 (5.7,14.3)	Metropolitan
	Incheon	Ganghwa-gun	13.5 (12.2,14.8)	20.5 (16.8,24.2)	15.9 (12.8,18.9)	10.9 (8.5,13.3)	9.4 (7.1,11.7)	10.9 (8.3,13.4)	9.6 (3.0,12.2)	Rural
	Incheon	Ongjin-gun	10.8 (9.7,12.0)	15.5 (12.3,18.8)	9.8 (7.4,12.2)	10.2 (7.6,12.8)	9.5 (7.1,12.0)	9.0 (6.6,11.4)	6.5 (3.6,11.8)	Rural
	Gwangju	Dong-gu	13.0 (11.7,14.3)	21.1 (17.3,24.9)	15.2 (12.1,18.4)	9.9 (7.5,12.3)	10.4 (7.8,13.0)	8.8 (6.3,11.2)	12.3 (8.5,17.3)	Metropolitan
	Gwangju	Seo-gu	10.8 (9.4,12.1)	15.3 (11.9,18.8)	12.6 (9.2,15.9)	11.1 (8.1,14.0)	7.6 (5.2,10.0)	7.8 (5.2,10.4)	7.5 (5.6,13.0)	Metropolitan
	Gwangju	Nam-gu	12.9 (11.6,14.2)	21.3 (17.4,25.1)	15.1 (12.0,18.2)	9.7 (7.1,12.3)	9.0 (6.6,11.4)	9.8 (7.3,12.4)	11.5 (7.0,15.8)	Metropolitan
	Gwangju	Buk-gu	13.5 (12.1,14.9)	19.3 (15.4,23.1)	13.7 (10.6,16.9)	11.6 (8.6,14.6)	12.0 (8.9,15.0)	10.9 (8.0,13.8)	8.4 (5.0,13.6)	Metropolitan
	Gwangju	Gwangsan-gu	12.8 (11.2,14.3)	21.1 (16.6,25.5)	14.9 (11.2,18.6)	11.0 (7.8,14.2)	8.2 (5.4,11.0)	8.8 (5.8,11.8)	12.3 (9.1,17.7)	Metropolitan
	Daejeon	Dong-gu	12.7 (11.3,14.0)	25.1 (20.9,29.3)	12.2 (9.3,15.0)	9.2 (6.7,11.7)	8.1 (5.6,10.5)	8.9 (6.4,11.4)	16.2 (9.2,18.2)	Metropolitan
	Daejeon	Jung-gu	11.3 (10.0,12.5)	18.6 (14.9,22.3)	12.0 (9.2,14.9)	10.1 (7.4,12.9)	9.5 (7.0,11.9)	6.5 (4.4,8.7)	12.1 (6.5,15.1)	Metropolitan
	Daejeon	Seo-gu	11.4 (10.0,12.8)	18.7 (14.5,22.8)	13.4 (10.3,16.5)	10.8 (7.7,13.9)	8.5 (5.9,11.2)	6.5 (4.2,8.8)	12.2 (8.9,16.9)	Metropolitan
	Daejeon	Yuseong-gu	10.4 (9.0,11.8)	15.5 (11.9,19.1)	12.4 (8.9,15.8)	8.9 (6.1,11.7)	7.2 (4.5,9.8)	7.1 (4.3,9.8)	8.4 (4.0,12.0)	Metropolitan
	Daejeon	Daedeok-gu	11.0 (9.7,12.4)	16.7 (13.0,20.5)	13.0 (9.9,16.1)	8.8 (6.1,11.6)	9.4 (6.5,12.2)	7.2 (4.6,9.8)	9.5 (5.9,14.1)	Metropolitan
	Ulsan	Jung-gu	13.9 (12.4,15.4)	20.8 (16.8,24.7)	13.0 (9.7,16.4)	11.7 (8.5,14.9)	11.9 (8.9,14.8)	11.9 (8.8,15.0)	8.9 (7.1,16.1)	Metropolitan
	Ulsan	Nam-gu	10.4 (9.0,11.8)	13.9 (10.1,17.8)	10.8 (7.6,13.9)	11.1 (7.9,14.2)	8.9 (5.9,11.9)	7.9 (5.3,10.4)	6.0 (2.5,9.9)	Metropolitan
	Ulsan	Dong-gu	10.0 (8.6,11.4)	17.3 (13.1,21.4)	10.5 (7.2,13.8)	8.8 (6.0,11.5)	6.6 (4.1,9.0)	7.6 (4.7,10.5)	9.7 (6.6,14.0)	Metropolitan
	Ulsan	Buk-gu	10.3 (8.8,11.8)	14.4 (10.5,18.4)	11.9 (8.3,15.4)	9.3 (6.0,12.5)	7.5 (4.6,10.4)	8.8 (5.5,12.0)	5.6 (2.6,10.2)	Metropolitan
	Ulsan	Ulju-gun	14.9 (13.4,16.3)	23.9 (19.6,28.2)	16.1 (12.7,19.6)	12.6 (9.6,15.6)	10.0 (7.3,12.8)	12.2 (9.2,15.2)	11.7 (5.5,14.9)	Rural
	Sejong	Sejong	15.1 (13.7,16.5)	24.3 (20.2,28.4)	14.8 (11.8,17.8)	15.0 (11.9,18.1)	11.6 (8.9,14.2)	9.9 (7.3,12.5)	14.4 (9.3,18.5)	Metropolitan
	Gyeonggi-do	Jangan-gu	10.8 (9.5,12.2)	18.2 (14.2,22.2)	12.5 (9.3,15.7)	10.0 (7.3,12.6)	7.2 (4.8,9.7)	6.3 (3.9,8.6)	11.9 (8.1,16.5)	Metropolitan
	Gyeonggi-do	Gwonseon-gu	10.7 (9.3,12.1)	17.4 (13.4,21.5)	9.9 (6.9,13.0)	10.7 (7.7,13.7)	8.6 (5.9,11.3)	6.9 (4.3,9.5)	10.5 (7.0,14.8)	Metropolitan
	Gyeonggi-do	Paldal-gu	11.0 (9.7,12.4)	16.2 (12.6,19.9)	11.3 (8.4,14.2)	8.5 (5.9,11.2)	9.6 (6.9,12.3)	9.6 (6.8,12.3)	6.6 (1.2,11.0)	Metropolitan
	Gyeonggi-do	Yeongtong-gu	9.6 (8.1,11.2)	11.9 (8.1,15.7)	9.9 (6.3,13.5)	8.2 (5.2,11.1)	8.9 (5.6,12.1)	9.0 (5.3,12.6)	2.9 (-1.1,6.5)	Metropolitan
	Gyeonggi-do	Sujeong-gu	13.5 (12.0,14.9)	20.4 (16.4,24.3)	14.3 (11.0,17.5)	12.7 (9.6,15.9)	10.0 (7.3,12.6)	10.1 (7.3,12.9)	10.3 (5.9,14.5)	Metropolitan
	Gyeonggi-do	Jungwon-gu	13.5 (12.0,14.9)	22.9 (18.7,27.0)	13.2 (10.1,16.4)	11.7 (8.7,14.8)	10.1 (7.3,12.9)	9.7 (7.0,12.4)	13.2 (7.1,16.1)	Metropolitan
	Gyeonggi-do	Bundang-gu	6.2 (5.2,7.2)	9.1 (6.4,11.8)	5.1 (3.1,7.1)	6.4 (4.2,8.6)	5.3 (3.3,7.4)	5.2 (3.1,7.3)	3.9 (-0.3,5.7)	Metropolitan
	Gyeonggi-do	Uijeongbu-si	12.6 (11.2,14.0)	20.0 (16.0,24.0)	13.0 (9.8,16.2)	10.9 (8.0,13.7)	9.7 (7.1,12.4)	9.7 (7.0,12.5)	10.3 (4.6,13.6)	Urban
	Gyeonggi-do	Manan-gu	10.7 (9.4,11.9)	17.6 (14.0,21.3)	13.2 (10.1,16.3)	9.1 (6.5,11.7)	7.2 (4.8,9.5)	6.3 (4.1,8.6)	11.3 (7.3,15.3)	Metropolitan
	Gyeonggi-do	Dongan-gu	9.2 (7.9,10.5)	13.7 (10.0,17.4)	8.3 (5.4,11.1)	8.6 (6.0,11.3)	6.2 (3.9,8.5)	9.4 (6.5,12.3)	4.3 (0.1,7.7)	Metropolitan
	Gyeonggi-do	Wonmi-gu	11.2 (9.8,12.7)	16.2 (12.3,20.0)	11.5 (8.3,14.7)	8.5 (5.7,11.4)	9.8 (6.9,12.7)	10.3 (7.1,13.4)	5.9 (2.2,10.8)	Metropolitan
	Gyeonggi-do	Sosa-gu	11.7 (10.3,13.1)	17.2 (13.3,21.1)	12.7 (9.6,15.8)	9.6 (6.9,12.4)	10.2 (7.4,13.1)	9.2 (6.4,12.1)	8.0 (5.8,14.4)	Metropolitan
	Gyeonggi-do	Ojeong-gu	12.5 (11.1,13.9)	21.8 (17.7,26.0)	11.4 (8.4,14.5)	9.7 (6.9,12.4)	9.8 (7.0,12.6)	9.8 (6.8,12.8)	12.0 (6.5,15.3)	Metropolitan
	Gyeonggi-do	Gwangmyeong-si	11.2 (9.9,12.5)	13.9 (10.4,17.3)	11.9 (8.9,14.9)	11.7 (8.6,14.7)	8.9 (6.2,11.6)	10.0 (7.1,12.9)	3.9 (2.0,10.4)	Urban
	Gyeonggi-do	Pyeongtaek-si	12.0 (11.1,12.9)	19.2 (16.6,21.8)	12.5 (10.3,14.6)	10.0 (8.1,11.9)	9.8 (7.9,11.7)	8.7 (6.9,10.4)	10.5 (8.5,14.5)	Urban
	Gyeonggi-do	Dongducheon-si	13.2 (11.8,14.5)	19.5 (15.8,23.2)	15.0 (11.8,18.2)	13.6 (10.7,16.5)	9.2 (6.7,11.8)	8.8 (6.4,11.2)	10.7 (6.9,15.7)	Urban
	Gyeonggi-do	Sangnok-gu	11.1 (9.7,12.5)	18.2 (14.1,22.2)	10.0 (6.9,13.1)	9.0 (6.0,11.9)	9.5 (6.6,12.3)	8.6 (5.8,11.4)	9.6 (7.9,16.5)	Metropolitan
	Gyeonggi-do	Danwon-gu	11.7 (10.2,13.2)	17.9 (13.6,22.2)	12.4 (9.1,15.8)	10.2 (7.1,13.3)	9.6 (6.5,12.6)	8.7 (5.7,11.6)	9.2 (3.5,11.7)	Metropolitan
	Gyeonggi-do	Deogyang-gu	12.8 (11.4,14.1)	21.8 (17.7,25.9)	13.2 (10.2,16.3)	9.5 (6.9,12.2)	9.2 (6.6,11.8)	10.3 (7.5,13.2)	11.5 (5.1,13.9)	Metropolitan
	Gyeonggi-do	Ilsandong-gu	9.4 (8.2,10.7)	13.5 (10.0,17.1)	10.1 (7.3,13.0)	10.1 (7.3,12.9)	8.0 (5.5,10.5)	5.8 (3.5,8.2)	7.7 (4.3,12.1)	Metropolitan
	Gyeonggi-do	Ilsanseo-gu	9.5 (8.3,10.8)	15.5 (11.9,19.1)	8.7 (5.9,11.4)	9.5 (6.9,12.1)	7.5 (5.1,9.9)	6.8 (4.5,9.1)	8.7 (2.6,10.6)	Metropolitan
	Gyeonggi-do	Gwacheon-si	8.8 (7.6,9.9)	13.3 (9.9,16.7)	8.4 (5.9,10.9)	7.6 (5.1,10.0)	8.9 (6.3,11.4)	5.8 (3.7,7.9)	7.5 (3.2,10.6)	Urban
	Gyeonggi-do	Guri-si	13.2 (11.7,14.7)	19.9 (15.8,23.9)	13.8 (10.5,17.1)	10.7 (7.7,13.6)	12.5 (9.4,15.7)	9.0 (6.2,11.8)	10.9 (4.4,13.4)	Urban
	Gyeonggi-do	Namyangju-si	13.1 (11.7,14.5)	18.7 (14.9,22.5)	13.0 (9.9,16.1)	11.8 (8.8,14.8)	11.6 (8.5,14.6)	10.5 (7.5,13.4)	8.2 (4.0,12.8)	Urban
	Gyeonggi-do	Osan-si	11.4 (9.9,12.9)	15.8 (11.8,19.8)	10.4 (7.4,13.5)	10.5 (7.4,13.6)	12.5 (9.0,16.0)	8.2 (5.4,10.9)	7.6 (4.1,12.1)	Urban
	Gyeonggi-do	Siheung-si	11.4 (9.9,12.9)	18.6 (14.4,22.8)	11.9 (8.6,15.2)	7.6 (4.9,10.3)	9.2 (6.1,12.4)	9.8 (6.7,12.8)	8.8 (4.8,13.2)	Urban
	Gyeonggi-do	Gunpo-si	11.8 (10.4,13.2)	18.3 (14.3,22.3)	11.0 (8.1,13.9)	9.2 (6.3,12.0)	11.4 (8.4,14.5)	9.5 (6.7,12.3)	8.8 (1.4,10.0)	Urban
	Gyeonggi-do	Uiwang-si	10.2 (8.9,11.4)	14.7 (11.3,18.1)	11.6 (8.5,14.8)	7.2 (4.8,9.6)	9.6 (6.9,12.2)	8.3 (5.8,10.9)	6.4 (3.5,11.5)	Urban
	Gyeonggi-do	Hanam-si	12.7 (11.3,14.1)	17.9 (14.2,21.6)	13.5 (10.2,16.8)	11.2 (8.3,14.1)	11.4 (8.5,14.3)	10.1 (7.2,13.0)	7.8 (3.7,12.3)	Urban
	Gyeonggi-do	Cheoin-gu	10.7 (9.5,12.0)	14.6 (11.3,17.8)	13.7 (10.5,16.8)	7.8 (5.5,10.0)	10.7 (7.9,13.5)	7.5 (5.2,9.8)	7.1 (0.5,8.3)	Metropolitan
	Gyeonggi-do	Giheung-gu	8.4 (7.3,9.6)	13.7 (10.3,17.0)	8.1 (5.6,10.6)	7.7 (5.3,10.0)	7.8 (5.3,10.4)	5.1 (3.2,7.0)	8.6 (3.4,10.4)	Metropolitan
	Gyeonggi-do	Suji-gu	7.9 (6.8,8.9)	10.1 (7.3,12.8)	9.1 (6.5,11.8)	7.3 (5.1,9.6)	6.7 (4.5,8.9)	6.1 (3.9,8.2)	4.0 (1.3,8.3)	Metropolitan
	Gyeonggi-do	Paju-si	12.3 (11.0,13.7)	19.3 (15.5,23.1)	13.2 (10.1,16.2)	10.6 (7.9,13.4)	11.0 (8.2,13.8)	8.1 (5.7,10.5)	11.2 (5.1,13.7)	Urban
	Gyeonggi-do	Icheon-si	12.5 (11.2,13.9)	16.8 (13.3,20.2)	12.0 (9.0,15.0)	12.4 (9.4,15.3)	11.0 (8.3,13.7)	10.5 (7.7,13.3)	6.3 (3.4,11.8)	Urban
	Gyeonggi-do	Anseong-si	12.2 (10.9,13.4)	18.1 (14.7,21.5)	12.7 (9.8,15.7)	10.0 (7.4,12.6)	10.3 (7.7,12.9)	9.4 (6.9,11.9)	8.7 (5.0,13.4)	Urban
	Gyeonggi-do	Gimpo-si	10.3 (9.1,11.5)	15.4 (12.1,18.8)	10.3 (7.7,13.0)	10.0 (7.3,12.6)	8.6 (6.2,11.0)	7.6 (5.2,9.9)	7.8 (4.3,11.9)	Urban
	Gyeonggi-do	Hwaseongsi	11.4 (10.0,12.7)	16.6 (13.0,20.2)	13.1 (9.8,16.5)	10.3 (7.4,13.1)	8.2 (5.6,10.7)	9.0 (6.2,11.9)	7.6 (5.2,13.2)	Urban
	Gyeonggi-do	Gwangju-si	11.8 (10.5,13.1)	16.0 (12.7,19.4)	12.3 (9.3,15.2)	10.9 (8.2,13.6)	10.0 (7.3,12.7)	9.6 (7.0,12.3)	6.4 (2.5,10.7)	Urban

(continued to the next page)

Appendix 4. Continued

Gender	Provinces	Districts	Overall	Q1 (lowest)	Q2	Q3	Q4	Q5 (highest)	Q1-Q5	Urbanity
	Gyeonggi-do	Yangju-si	13.1 (11.7,14.4)	20.1 (16.3,24.0)	12.0 (9.1,14.9)	10.3 (7.5,13.0)	12.0 (9.2,14.9)	11.3 (8.3,14.2)	8.8 (3.6,12.2)	Urban
	Gyeonggi-do	Pocheon-si	14.0 (12.7,15.3)	23.0 (18.9,27.1)	13.3 (10.5,16.1)	13.2 (10.3,16.1)	10.5 (7.9,13.1)	10.6 (8.0,13.3)	12.4 (6.3,15.3)	Urban
	Gyeonggi-do	Yeoju-gun	13.7 (12.4,15.0)	20.1 (16.6,23.6)	13.9 (11.0,16.8)	11.3 (8.7,14.0)	12.3 (9.6,15.0)	11.3 (8.6,13.9)	8.8 (1.2,10.2)	Urban
	Gyeonggi-do	Yeoncheon-gun	13.4 (12.1,14.7)	22.6 (18.8,26.4)	14.4 (11.5,17.2)	11.3 (8.9,13.8)	10.4 (7.9,12.9)	9.0 (6.8,11.2)	13.6 (6.1,14.9)	Rural
	Gyeonggi-do	Gapyeong-gun	12.5 (11.3,13.7)	21.5 (17.8,25.3)	13.5 (10.7,16.3)	11.5 (9.0,14.0)	8.2 (6.0,10.3)	8.0 (5.8,10.1)	13.5 (7.7,16.3)	Rural
	Gyeonggi-do	Yangpyeong-gun	12.9 (11.6,14.1)	18.3 (14.9,21.6)	15.2 (12.2,18.1)	10.2 (7.7,12.6)	12.4 (9.8,15.0)	8.1 (5.9,10.3)	10.2 (6.6,15.2)	Rural
	Gangwon-do	Chuncheon-si	11.5 (10.3,12.8)	22.5 (18.3,26.6)	12.6 (9.7,15.5)	8.8 (6.3,11.3)	8.0 (5.7,10.4)	7.1 (4.9,9.3)	15.4 (8.6,17.2)	Urban
	Gangwon-do	Wonju-si	11.4 (10.2,12.7)	18.4 (14.7,22.1)	12.9 (10.0,15.8)	8.8 (6.3,11.4)	9.9 (7.4,12.5)	7.4 (5.0,9.7)	11.0 (5.8,14.2)	Urban
	Gangwon-do	Gangneung-si	13.6 (12.2,14.9)	21.9 (17.9,25.9)	13.4 (10.6,16.3)	12.5 (9.8,15.2)	11.7 (8.9,14.4)	8.9 (6.4,11.3)	13.0 (9.8,18.6)	Urban
	Gangwon-do	Donghae-si	12.2 (10.9,13.4)	20.3 (16.6,24.0)	12.4 (9.5,15.3)	10.7 (8.1,13.4)	7.6 (5.4,9.9)	10.0 (7.4,12.7)	10.3 (6.1,14.9)	Urban
	Gangwon-do	Taebaek-si	18.7 (17.2,20.2)	26.1 (21.9,30.2)	21.0 (17.4,24.5)	18.0 (14.6,21.4)	14.3 (11.4,17.2)	14.7 (11.7,17.8)	11.4 (6.7,16.5)	Urban
	Gangwon-do	Sokcho-si	14.7 (13.2,16.1)	24.3 (20.1,28.5)	15.6 (12.3,18.8)	13.1 (10.2,16.1)	10.2 (7.5,12.8)	10.3 (7.5,13.1)	14.0 (8.8,18.4)	Urban
	Gangwon-do	Samcheok-si	15.1 (13.7,16.4)	26.6 (22.4,30.8)	16.2 (13.1,19.3)	12.3 (9.6,14.9)	11.1 (8.5,13.7)	9.9 (7.5,12.3)	16.7 (9.3,18.7)	Urban
	Gangwon-do	Hongcheon-gun	12.4 (11.2,13.6)	19.9 (16.4,23.3)	12.2 (9.6,14.8)	11.2 (8.6,13.7)	9.3 (7.1,11.4)	9.4 (7.0,11.8)	10.5 (7.9,16.7)	Rural
	Gangwon-do	Hoengseong-gun	14.3 (13.0,15.6)	19.9 (16.5,23.4)	15.6 (12.6,18.5)	13.8 (11.1,16.6)	12.3 (9.7,14.9)	10.1 (7.6,12.7)	9.8 (5.3,14.1)	Rural
	Gangwon-do	Yeongwol-gun	15.2 (13.9,16.6)	24.5 (20.5,28.5)	16.3 (13.3,19.3)	12.7 (10.0,15.3)	12.1 (9.5,14.7)	10.9 (8.4,13.4)	13.6 (9.6,18.6)	Rural
	Gangwon-do	Pyeongchang-gun	14.7 (13.4,16.1)	23.6 (19.7,27.5)	16.1 (13.0,19.2)	12.6 (10.0,15.3)	11.7 (9.1,14.3)	9.9 (7.5,12.4)	13.7 (7.3,16.3)	Rural
	Gangwon-do	Jeongseon-gun	17.8 (16.4,19.3)	25.6 (21.6,29.6)	19.2 (15.9,22.5)	18.0 (14.8,21.1)	14.9 (12.0,17.7)	11.7 (9.0,14.4)	13.9 (11.2,20.6)	Rural
	Gangwon-do	Cheorwon-gun	11.9 (10.7,13.1)	22.1 (18.4,25.8)	12.2 (9.6,14.8)	8.9 (6.5,11.3)	8.8 (6.5,11.2)	7.5 (5.3,9.6)	14.6 (9.7,18.3)	Rural
	Gangwon-do	Hwacheon-gun	13.7 (12.4,15.1)	21.5 (17.5,25.5)	14.8 (11.8,17.9)	11.3 (8.5,14.1)	12.7 (9.9,15.6)	8.6 (6.2,11.0)	12.9 (8.2,17.2)	Rural
	Gangwon-do	Yanggu-gun	13.8 (12.5,15.2)	22.2 (18.2,26.1)	13.6 (10.5,16.7)	13.2 (10.4,16.1)	11.3 (8.5,14.0)	9.1 (6.6,11.5)	13.1 (7.5,16.5)	Rural
	Gangwon-do	Inje-gun	13.2 (11.9,14.4)	19.5 (15.9,23.0)	13.7 (10.8,16.5)	13.5 (10.6,16.3)	9.7 (7.3,12.1)	9.5 (7.0,12.0)	10.0 (5.9,14.5)	Rural
	Gangwon-do	Goseong-gun	13.7 (12.4,15.0)	20.5 (16.8,24.2)	18.1 (14.6,21.6)	13.0 (10.1,15.9)	9.2 (6.9,11.5)	8.0 (5.8,10.3)	12.5 (8.3,17.1)	Rural
	Gangwon-do	Yangyang-gun	13.3 (12.0,14.5)	22.2 (18.4,26.0)	16.1 (13.0,19.2)	10.0 (7.5,12.4)	10.5 (8.0,13.0)	8.0 (5.9,10.1)	14.2 (8.8,17.8)	Rural
	Chungcheongbuk-do	Cheongju-si	11.0 (10.3,11.8)	17.4 (15.2,19.6)	12.6 (10.8,14.5)	9.1 (7.6,10.7)	9.1 (7.6,10.5)	7.8 (6.4,9.2)	9.6 (7.1,11.9)	Metropolitan
	Chungcheongbuk-do	Chungju-si	15.1 (13.7,16.5)	23.3 (19.4,27.3)	17.9 (14.6,21.2)	11.5 (8.9,14.2)	11.8 (9.1,14.4)	11.2 (8.5,13.9)	12.1 (8.6,17.8)	Urban
	Chungcheongbuk-do	Jecheon-si	12.4 (11.1,13.6)	19.7 (16.2,23.3)	12.5 (9.7,15.2)	10.9 (8.3,13.5)	9.7 (7.3,12.2)	9.5 (7.0,12.0)	10.2 (5.9,14.7)	Urban
	Chungcheongbuk-do	Boeun-gun	15.0 (13.7,16.3)	24.7 (20.7,28.8)	17.8 (14.6,21.1)	12.1 (9.6,14.5)	9.8 (7.5,12.2)	11.8 (9.1,14.5)	12.9 (9.1,18.5)	Rural
	Chungcheongbuk-do	Okcheon-gun	13.3 (12.0,14.6)	19.6 (16.0,23.1)	16.1 (13.0,19.3)	11.1 (8.6,13.6)	11.1 (8.6,13.7)	8.7 (6.3,11.0)	10.9 (5.0,13.8)	Rural
	Chungcheongbuk-do	Yeongdong-gun	13.0 (11.8,14.2)	22.0 (18.3,25.7)	12.9 (10.3,15.5)	10.3 (8.0,12.6)	10.3 (7.8,12.7)	9.6 (7.3,12.0)	12.4 (7.2,16.2)	Rural
	Chungcheongbuk-do	Jincheon-gun	13.9 (12.5,15.2)	20.7 (16.8,24.5)	11.2 (8.5,13.8)	14.3 (11.2,17.5)	11.6 (8.9,14.3)	12.3 (9.4,15.2)	8.4 (2.0,11.2)	Rural
	Chungcheongbuk-do	Goesan-gun	11.3 (10.1,12.5)	16.0 (12.8,19.2)	11.2 (8.6,13.8)	10.9 (8.2,13.6)	9.6 (7.1,12.0)	8.9 (6.5,11.3)	7.1 (2.1,10.3)	Rural
	Chungcheongbuk-do	Eumseong-gun	14.1 (12.8,15.3)	22.0 (18.0,25.9)	14.8 (11.9,17.8)	14.0 (11.4,16.6)	10.2 (7.8,12.5)	9.9 (7.5,12.3)	12.1 (7.7,16.5)	Rural
	Chungcheongbuk-do	Danyang-gun	14.6 (13.2,15.9)	22.9 (19.2,26.7)	13.3 (10.4,16.2)	13.0 (10.2,15.7)	10.1 (7.6,12.5)	13.3 (10.4,16.2)	9.6 (2.2,11.6)	Rural
	Chungcheongbuk-do	Jeungpyeong-gun	12.9 (11.7,14.1)	24.0 (20.1,28.0)	12.4 (9.9,15.0)	11.7 (9.3,14.1)	10.2 (7.8,12.5)	6.6 (4.7,8.5)	17.4 (11.9,20.5)	Rural
	Chungcheongnam-do	Cheonan-si	13.8 (12.2,15.3)	19.8 (15.7,23.9)	16.1 (12.4,19.8)	12.3 (9.1,15.5)	12.2 (9.1,15.4)	8.5 (5.9,11.1)	11.3 (8.0,16.6)	Metropolitan
	Chungcheongnam-do	Gongju-si	14.1 (12.8,15.3)	24.9 (21.0,28.9)	16.1 (13.0,19.2)	12.3 (9.6,14.9)	9.3 (7.1,11.4)	7.8 (5.7,9.9)	17.1 (13.0,21.8)	Urban
	Chungcheongnam-do	Boryeong-si	16.8 (15.4,18.2)	28.3 (23.9,32.7)	18.9 (15.6,22.1)	12.9 (10.1,15.7)	12.7 (9.9,15.4)	12.1 (9.4,14.9)	16.2 (11.4,21.2)	Urban
	Chungcheongnam-do	Asan-si	12.5 (11.2,13.8)	21.6 (17.5,25.6)	11.5 (8.7,14.4)	11.1 (8.2,14.0)	10.4 (7.7,13.1)	7.9 (5.6,10.3)	13.7 (7.1,15.3)	Urban
	Chungcheongnam-do	Seosan-si	13.4 (12.1,14.7)	20.0 (16.4,23.7)	14.0 (11.0,16.9)	12.4 (9.6,15.1)	11.4 (8.7,14.1)	9.1 (6.7,11.5)	10.9 (7.0,15.4)	Urban
	Chungcheongnam-do	Nonsan-si	14.6 (13.3,15.9)	24.3 (20.4,28.3)	16.3 (13.3,19.4)	12.1 (9.3,14.8)	10.6 (8.2,13.0)	9.4 (6.9,11.8)	14.9 (8.8,17.8)	Urban
	Chungcheongnam-do	Gyeryong-si	10.3 (8.8,11.7)	15.1 (11.0,19.1)	12.1 (8.6,15.6)	9.8 (6.6,13.0)	8.4 (5.4,11.3)	6.2 (3.7,8.8)	8.9 (5.3,12.7)	Urban
	Chungcheongnam-do	Dangjin-si	13.4 (12.1,14.7)	21.2 (17.5,24.8)	11.1 (8.4,13.8)	12.5 (9.8,15.3)	12.5 (9.8,15.3)	9.9 (7.4,12.4)	11.3 (5.0,14.0)	Rural
	Chungcheongnam-do	Geumsan-gun	14.1 (12.9,15.4)	21.0 (17.4,24.6)	16.9 (13.6,20.1)	12.7 (10.1,15.3)	10.8 (8.4,13.2)	9.4 (7.1,11.7)	11.6 (6.1,15.1)	Rural
	Chungcheongnam-do	Buyeo-gun	13.5 (12.3,14.8)	23.3 (19.2,27.3)	15.2 (12.3,18.2)	10.3 (8.1,12.5)	10.6 (8.2,13.0)	8.8 (6.6,10.9)	14.5 (9.5,18.5)	Rural
	Chungcheongnam-do	Seocheon-gun	14.9 (13.5,16.3)	24.0 (19.9,28.1)	16.4 (13.3,19.5)	12.8 (10.1,15.6)	10.0 (7.6,12.4)	11.9 (9.1,14.7)	12.1 (5.8,15.4)	Rural
	Chungcheongnam-do	Cheongyang-gun	13.2 (12.0,14.4)	23.0 (19.1,26.9)	13.7 (11.1,16.4)	11.6 (9.2,13.9)	10.1 (7.8,12.5)	8.1 (6.1,10.2)	14.9 (11.0,19.8)	Rural
	Chungcheongnam-do	Hongseong-gun	14.7 (13.4,16.1)	23.4 (19.6,27.2)	14.6 (11.7,17.5)	14.0 (11.1,16.9)	11.4 (8.8,14.0)	10.5 (7.9,13.0)	12.9 (8.8,18.0)	Rural
	Chungcheongnam-do	Yesan-gun	12.8 (11.6,14.0)	20.9 (17.3,24.6)	12.9 (10.2,15.7)	11.3 (8.9,13.8)	9.1 (6.9,11.3)	10.1 (7.6,12.5)	10.8 (6.6,15.6)	Rural
	Chungcheongnam-do	Taean-gun	13.3 (12.0,14.5)	17.8 (14.5,21.1)	14.7 (11.7,17.7)	12.8 (10.2,15.5)	11.1 (8.6,13.7)	10.2 (7.8,12.6)	7.6 (2.2,11.0)	Urban
	Jeollabuk-do	Jeonju-si	11.4 (10.1,12.7)	18.8 (15.0,22.6)	12.6 (9.6,15.6)	9.1 (6.6,11.7)	9.0 (6.4,11.6)	8.3 (5.9,10.7)	10.5 (6.3,14.9)	Metropolitan
	Jeollabuk-do	Gunsan-si	14.6 (13.2,16.0)	22.1 (18.2,26.0)	12.9 (9.8,15.9)	13.2 (10.3,16.2)	13.1 (10.1,16.1)	12.3 (9.4,15.1)	9.8 (5.6,14.8)	Urban
	Jeollabuk-do	Iksan-si	15.2 (13.8,16.6)	25.7 (21.5,29.9)	15.6 (12.5,18.7)	14.3 (11.4,17.3)	10.8 (8.1,13.4)	10.1 (7.6,12.6)	15.6 (12.2,21.6)	Urban
	Jeollabuk-do	Jeongeup-si	16.4 (14.9,17.8)	26.2 (22.0,30.5)	18.0 (14.7,21.3)	14.8 (11.9,17.7)	11.5 (8.9,14.1)	11.8 (9.0,14.6)	14.4 (8.2,18.0)	Urban
	Jeollabuk-do	Namwon-si	15.2 (13.8,16.5)	23.7 (19.7,27.6)	18.0 (14.7,21.4)	13.7 (10.9,16.6)	9.8 (7.4,12.1)	11.0 (8.5,13.6)	12.7 (9.5,18.7)	Urban
	Jeollabuk-do	Gimje-si	15.7 (14.4,17.1)	25.7 (21.6,29.8)	15.9 (12.9,18.9)	14.0 (11.2,16.9)	12.9 (10.2,15.6)	10.6 (8.0,13.2)	15.1 (9.1,18.7)	Urban
	Jeollabuk-do	Wanju-gun	13.3 (12.1,14.5)	18.9 (15.5,22.2)	17.8 (14.5,21.0)	11.9 (9.4,14.3)	8.5 (6.3,10.7)	9.6 (7.3,11.9)	9.3 (6.2,14.8)	Rural
	Jeollabuk-do	Jinan-gun	13.2 (11.9,14.4)	19.9 (16.4,23.5)	15.6 (12.5,18.6)	11.3 (8.9,13.8)	10.6 (8.2,13.0)	8.2 (6.1,10.3)	11.7 (7.9,17.1)	Rural
	Jeollabuk-do	Muju-gun	14.2 (12.8,15.5)	23.4 (19.4,27.4)	15.4 (12.5,18.4)	12.8 (10.1,15.6)	10.3 (7.9,12.7)	8.4 (6.1,10.6)	15.0 (10.2,19.6)	Rural
	Jeollabuk-do	Jangsu-gun	15.5 (14.2,16.9)	24.6 (20.4,28.7)	17.0 (13.8,20.2)	13.3 (10.5,16.0)	11.7 (9.2,14.2)	11.6 (9.0,14.2)	13.0 (6.8,16.6)	Rural

(continued to the next page)

Appendix 4. Continued

Gender	Provinces	Districts	Overall	Q1 (lowest)	Q2	Q3	Q4	Q5 (highest)	Q1-Q5	Urbanity
	Jeollabuk-do	Imsil-gun	15.1 (13.7,16.4)	23.6 (19.4,27.9)	14.8 (12.1,17.5)	15.2 (12.2,18.2)	13.5 (10.8,16.3)	8.1 (6.1,10.2)	15.5 (13.6,22.6)	Rural
	Jeollabuk-do	Sunchang-gun	14.9 (13.5,16.3)	22.6 (18.8,26.5)	19.8 (16.2,23.4)	12.4 (9.9,14.9)	11.3 (8.6,14.0)	8.3 (6.1,10.4)	14.3 (11.0,20.4)	Rural
	Jeollabuk-do	Gochang-gun	17.9 (16.4,19.4)	29.8 (25.1,34.6)	18.7 (15.3,22.1)	14.8 (11.9,17.7)	16.2 (13.0,19.4)	10.7 (8.2,13.1)	19.1 (13.7,23.5)	Rural
	Jeollabuk-do	Buan-gun	17.2 (15.7,18.6)	29.1 (24.7,33.6)	19.1 (15.5,22.7)	15.9 (12.8,18.9)	9.8 (7.4,12.1)	11.5 (8.8,14.1)	17.6 (12.4,22.8)	Rural
	Jeollanam-do	Mokpo-si	14.0 (12.6,15.4)	24.2 (20.0,28.4)	15.9 (12.5,19.3)	12.5 (9.6,15.4)	10.4 (7.7,13.1)	7.3 (5.0,9.6)	16.9 (9.8,18.6)	Urban
	Jeollanam-do	Yeosu-si	13.2 (11.9,14.5)	21.6 (17.8,25.4)	13.5 (10.7,16.3)	9.8 (7.3,12.4)	10.2 (7.6,12.8)	11.0 (8.2,13.8)	10.6 (5.6,14.6)	Urban
	Jeollanam-do	Suncheon-si	12.2 (10.9,13.4)	20.1 (16.4,23.8)	13.9 (10.8,17.0)	8.8 (6.4,11.2)	8.1 (5.7,10.5)	10.0 (7.4,12.6)	10.1 (7.3,16.3)	Urban
	Jeollanam-do	Naju-si	14.9 (13.6,16.3)	26.5 (22.4,30.7)	14.5 (11.6,17.4)	13.3 (10.5,16.1)	11.1 (8.6,13.5)	9.2 (6.9,11.5)	17.3 (13.1,22.5)	Urban
	Jeollanam-do	Gwangyang-si	11.2 (9.9,12.5)	18.0 (14.2,21.7)	12.5 (9.4,15.5)	9.9 (6.9,12.8)	7.9 (5.4,10.3)	8.2 (5.5,10.8)	9.8 (4.2,12.2)	Urban
	Jeollanam-do	Damyang-gun	14.7 (13.4,16.0)	25.7 (21.6,29.9)	15.6 (12.5,18.6)	13.4 (10.8,16.0)	9.5 (7.4,11.7)	9.6 (7.2,12.0)	16.1 (12.1,21.1)	Rural
	Jeollanam-do	Gokseong-gun	14.1 (12.8,15.5)	20.9 (17.0,24.8)	14.9 (11.8,17.9)	13.9 (11.0,16.7)	9.1 (6.8,11.3)	12.4 (9.6,15.3)	8.5 (3.9,13.5)	Rural
	Jeollanam-do	Gurye-gun	11.7 (10.5,12.9)	23.1 (19.1,27.1)	13.8 (10.9,16.8)	7.4 (5.5,9.3)	9.0 (6.7,11.2)	5.9 (4.1,7.8)	17.2 (11.2,20.2)	Rural
	Jeollanam-do	Goheung-gun	14.4 (13.1,15.8)	30.2 (25.3,35.1)	13.3 (10.6,16.0)	11.8 (9.4,14.2)	11.1 (8.6,13.6)	7.0 (5.1,8.8)	23.2 (18.9,28.1)	Rural
	Jeollanam-do	Boseong-gun	15.2 (13.8,16.6)	25.5 (21.1,29.8)	19.3 (15.9,22.8)	12.8 (10.2,15.3)	10.4 (8.0,12.8)	7.8 (5.9,9.8)	17.7 (13.7,23.5)	Rural
	Jeollanam-do	Hwasun-gun	15.9 (14.5,17.3)	25.5 (21.3,29.6)	16.0 (13.0,19.1)	15.2 (12.2,18.2)	10.8 (8.2,13.4)	12.2 (9.4,15.0)	13.3 (8.1,18.1)	Rural
	Jeollanam-do	Jangheung-gun	15.2 (13.8,16.6)	29.8 (25.0,34.5)	15.9 (12.9,18.9)	12.5 (9.8,15.2)	9.4 (7.3,11.5)	9.4 (6.8,11.9)	20.4 (12.9,22.5)	Rural
	Jeollanam-do	Gangjin-gun	12.0 (10.8,13.2)	21.0 (17.1,25.0)	14.7 (11.6,17.8)	10.9 (8.4,13.4)	9.2 (6.8,11.5)	4.9 (3.4,6.4)	16.1 (10.8,19.2)	Rural
	Jeollanam-do	Haenam-gun	15.6 (14.2,17.0)	26.2 (21.8,30.7)	14.8 (11.8,17.7)	13.6 (10.9,16.3)	12.8 (10.0,15.6)	11.7 (9.0,14.5)	14.5 (7.7,17.3)	Rural
	Jeollanam-do	Yeongam-gun	15.0 (13.6,16.3)	24.6 (20.7,28.6)	16.5 (13.4,19.6)	12.2 (9.4,14.9)	11.6 (9.1,14.2)	10.3 (7.8,12.7)	14.3 (10.3,19.5)	Rural
	Jeollanam-do	Muan-gun	13.7 (12.4,15.0)	20.5 (16.8,24.1)	14.4 (11.5,17.3)	13.9 (11.1,16.7)	9.9 (7.6,12.2)	10.5 (7.9,13.1)	10.0 (6.3,15.3)	Rural
	Jeollanam-do	Hampyeong-gun	15.6 (14.2,17.0)	25.5 (21.3,29.7)	16.1 (13.0,19.2)	13.1 (10.3,16.0)	12.9 (10.2,15.7)	10.2 (7.8,12.6)	15.3 (9.3,18.7)	Rural
	Jeollanam-do	Yeonggwang-gun	15.8 (14.5,17.2)	26.3 (22.2,30.4)	18.7 (15.4,22.0)	15.0 (12.0,17.9)	11.2 (8.8,13.7)	8.3 (6.1,10.4)	18.0 (14.7,24.1)	Rural
	Jeollanam-do	Jangseong-gun	15.3 (14.0,16.7)	25.8 (21.6,29.9)	15.6 (12.5,18.8)	12.8 (10.1,15.4)	12.1 (9.5,14.8)	10.6 (8.1,13.1)	15.2 (7.2,17.0)	Rural
	Jeollanam-do	Wando-gun	15.7 (14.4,17.1)	28.8 (24.4,33.3)	16.7 (13.7,19.7)	12.0 (9.6,14.4)	12.6 (10.0,15.3)	8.9 (6.6,11.1)	19.9 (15.4,24.8)	Rural
	Jeollanam-do	Jindo-gun	15.4 (14.1,16.8)	26.8 (22.4,31.1)	17.4 (14.2,20.6)	13.1 (10.6,15.7)	9.9 (7.7,12.1)	9.9 (7.5,12.4)	16.9 (12.3,21.7)	Rural
	Jeollanam-do	Sinan-gun	16.7 (15.3,18.1)	24.4 (20.5,28.3)	18.9 (15.7,22.1)	16.3 (13.1,19.5)	12.2 (9.7,14.7)	11.0 (8.4,13.7)	13.4 (10.5,20.1)	Rural
	Gyeongsangbuk-do	Nam-gu	13.8 (12.4,15.2)	23.9 (19.8,27.9)	14.8 (11.7,17.9)	12.1 (9.0,15.1)	9.5 (6.7,12.2)	8.7 (6.3,11.2)	15.2 (10.9,19.5)	Metropolitan
	Gyeongsangbuk-do	Buk-gu	12.4 (11.1,13.7)	19.0 (15.4,22.7)	12.6 (9.8,15.4)	11.0 (8.2,13.8)	9.8 (7.2,12.4)	9.5 (6.9,12.1)	9.5 (5.9,14.3)	Metropolitan
	Gyeongsangbuk-do	Gyeongju-si	15.2 (13.8,16.6)	23.5 (19.6,27.5)	15.0 (11.9,18.1)	14.9 (12.0,17.9)	10.9 (8.3,13.4)	12.0 (9.2,14.8)	11.5 (5.8,15.0)	Urban
	Gyeongsangbuk-do	Gimcheon-si	14.8 (13.5,16.2)	22.9 (19.2,26.7)	16.0 (12.9,19.1)	14.2 (11.2,17.1)	10.1 (7.6,12.6)	10.8 (8.2,13.4)	12.1 (6.6,15.8)	Urban
	Gyeongsangbuk-do	Andong-si	14.2 (12.9,15.6)	23.8 (19.8,27.7)	15.2 (12.3,18.1)	13.2 (10.4,16.1)	9.9 (7.6,12.3)	9.8 (7.3,12.2)	14.0 (8.2,17.0)	Urban
	Gyeongsangbuk-do	Gumi-si	14.6 (13.4,15.8)	22.7 (19.3,26.1)	14.6 (12.0,17.2)	14.3 (11.6,17.0)	11.7 (9.3,14.1)	11.1 (8.9,13.4)	11.6 (6.6,13.2)	Urban
	Gyeongsangbuk-do	Yeongju-si	14.8 (13.4,16.1)	26.1 (21.8,30.3)	14.0 (11.2,16.9)	13.9 (11.1,16.8)	11.6 (8.9,14.2)	8.6 (6.4,10.8)	17.5 (11.7,20.9)	Urban
	Gyeongsangbuk-do	Yeongcheon-si	16.7 (15.2,18.1)	24.3 (20.3,28.3)	19.6 (16.1,23.1)	14.9 (12.1,17.7)	10.9 (8.4,13.4)	13.6 (10.6,16.5)	10.7 (7.2,17.0)	Urban
	Gyeongsangbuk-do	Sangju-si	15.2 (13.8,16.5)	23.4 (19.6,27.1)	14.9 (12.1,17.7)	13.8 (11.0,16.5)	13.3 (10.6,15.9)	10.3 (7.8,12.7)	13.1 (8.6,17.6)	Urban
	Gyeongsangbuk-do	Mungyeong-si	15.7 (14.4,17.1)	25.0 (21.0,28.9)	12.5 (9.8,15.2)	16.2 (13.1,19.3)	12.6 (9.9,15.3)	12.6 (9.7,15.5)	12.4 (5.8,15.8)	Urban
	Gyeongsangbuk-do	Gyeongsan-si	15.3 (13.9,16.7)	23.6 (19.6,27.7)	15.1 (12.1,18.2)	11.9 (9.0,14.8)	13.6 (10.6,16.7)	12.3 (9.4,15.2)	11.3 (4.1,13.3)	Urban
	Gyeongsangbuk-do	Gunwi-gun	14.6 (13.3,15.9)	22.2 (18.1,26.3)	16.6 (13.5,19.7)	13.1 (10.5,15.6)	11.7 (9.1,14.2)	10.1 (7.7,12.5)	12.1 (5.2,14.6)	Rural
	Gyeongsangbuk-do	Uiseong-gun	13.6 (12.4,14.9)	18.5 (15.0,22.0)	15.6 (12.6,18.6)	12.0 (9.5,14.5)	11.5 (8.9,14.2)	11.1 (8.4,13.7)	7.4 (3.1,12.3)	Rural
	Gyeongsangbuk-do	Cheongsong-gun	16.9 (15.4,18.4)	27.8 (23.2,32.4)	16.1 (13.2,19.0)	14.7 (11.8,17.6)	12.3 (9.7,14.9)	14.0 (10.8,17.3)	13.8 (9.2,19.2)	Rural
	Gyeongsangbuk-do	Yeongyang-gun	17.3 (15.8,18.7)	24.5 (20.6,28.4)	21.0 (17.5,24.5)	15.3 (12.4,18.3)	13.6 (10.8,16.3)	11.6 (8.9,14.3)	12.9 (11.0,20.6)	Rural
	Gyeongsangbuk-do	Yeongdeok-gun	16.7 (15.2,18.3)	30.3 (25.3,35.3)	20.3 (16.6,23.9)	12.9 (10.1,15.8)	10.8 (8.3,13.3)	9.7 (7.2,12.2)	20.6 (12.2,21.8)	Rural
	Gyeongsangbuk-do	Cheongdo-gun	16.1 (14.7,17.6)	27.9 (23.3,32.5)	14.7 (11.8,17.5)	14.8 (11.9,17.7)	13.1 (10.2,15.9)	10.7 (8.1,13.2)	17.2 (9.5,19.5)	Rural
	Gyeongsangbuk-do	Goryeong-gun	14.7 (13.3,16.0)	21.1 (17.4,24.8)	14.2 (11.3,17.1)	14.0 (11.1,16.9)	12.0 (9.4,14.6)	12.0 (9.3,14.8)	9.1 (3.7,13.3)	Rural
	Gyeongsangbuk-do	Seongju-gun	10.4 (9.3,11.5)	18.8 (15.3,22.3)	10.6 (8.2,13.1)	7.4 (5.3,9.5)	7.5 (5.5,9.5)	7.7 (5.7,9.8)	11.1 (7.8,16.0)	Rural
	Gyeongsangbuk-do	Chilgok-gun	14.8 (13.3,16.3)	24.2 (20.0,28.4)	15.5 (12.0,18.9)	13.6 (10.6,16.7)	9.1 (6.5,11.6)	11.7 (8.7,14.7)	12.5 (7.1,16.3)	Rural
	Gyeongsangbuk-do	Yecheon-gun	16.1 (14.7,17.6)	31.0 (26.2,35.8)	15.0 (12.1,18.0)	14.7 (11.7,17.6)	11.3 (8.8,13.9)	9.4 (7.0,11.8)	21.6 (12.7,22.7)	Rural
	Gyeongsangbuk-do	Bonghwa-gun	15.9 (14.5,17.2)	23.5 (19.7,27.4)	15.7 (12.8,18.6)	17.3 (14.0,20.5)	10.6 (8.3,12.8)	12.0 (9.2,14.8)	11.5 (5.3,14.9)	Rural
	Gyeongsangbuk-do	Uljin-gun	15.1 (13.8,16.5)	23.2 (19.3,27.1)	15.8 (12.7,18.9)	11.4 (9.0,13.8)	13.2 (10.3,16.0)	12.5 (9.8,15.2)	10.7 (6.1,16.1)	Rural
	Gyeongsangbuk-do	Ulleung-gun	13.1 (11.8,14.4)	21.7 (17.9,25.5)	14.5 (11.4,17.6)	11.3 (8.8,13.9)	9.1 (6.7,11.4)	9.4 (6.9,11.9)	12.3 (8.9,18.3)	Rural
	Gyeongsangnam-do	Changwon-si	13.6 (11.9,15.2)	18.6 (14.2,22.9)	15.2 (11.1,19.4)	13.4 (10.0,16.9)	11.3 (8.0,14.7)	9.5 (6.2,12.8)	9.1 (6.0,14.6)	Metropolitan
	Gyeongsangnam-do	Masan-si	12.6 (11.3,13.9)	19.9 (16.2,23.6)	11.3 (8.6,13.9)	11.8 (9.0,14.5)	11.6 (8.7,14.5)	8.6 (6.1,11.1)	11.3 (6.2,14.8)	Metropolitan
	Gyeongsangnam-do	Jinju-si	12.8 (11.5,14.1)	22.7 (18.6,26.8)	11.4 (8.7,14.2)	12.4 (9.4,15.3)	10.6 (7.9,13.3)	7.1 (4.8,9.4)	15.6 (10.1,18.9)	Urban
	Gyeongsangnam-do	Jinhae-si	13.1 (11.7,14.5)	21.5 (17.2,25.7)	13.3 (10.3,16.4)	13.1 (9.9,16.3)	10.4 (7.6,13.1)	8.1 (5.6,10.6)	13.4 (7.7,16.5)	Metropolitan
	Gyeongsangnam-do	Tongyeong-si	16.2 (14.7,17.6)	26.7 (22.4,31.1)	18.3 (15.0,21.7)	14.2 (11.1,17.4)	11.7 (8.9,14.5)	10.1 (7.5,12.8)	16.6 (11.3,20.5)	Urban
	Gyeongsangnam-do	Sacheon-si	15.1 (13.7,16.5)	25.8 (21.7,30.0)	15.8 (12.4,19.1)	11.0 (8.4,13.5)	12.1 (9.3,14.9)	11.4 (8.6,14.2)	14.4 (8.5,18.1)	Urban
	Gyeongsangnam-do	Gimhae-si	13.8 (12.2,15.3)	22.4 (17.9,27.0)	13.9 (10.6,17.2)	11.9 (8.6,15.2)	10.2 (7.4,13.1)	11.5 (8.2,14.8)	10.9 (5.6,14.6)	Urban
	Gyeongsangnam-do	Miryang-si	14.8 (13.4,16.1)	27.0 (22.9,31.1)	15.9 (12.8,18.9)	12.0 (9.4,14.6)	8.8 (6.6,11.1)	10.4 (7.9,12.9)	16.6 (13.6,22.8)	Urban
	Gyeongsangnam-do	Geoje-si	13.6 (12.1,15.2)	20.3 (16.1,24.4)	12.9 (9.5,16.3)	12.0 (8.7,15.4)	10.8 (7.8,13.7)	12.1 (8.9,15.2)	8.2 (3.9,12.9)	Urban
	Gyeongsangnam-do	Yangsan-si	11.4 (10.1,12.7)	17.3 (13.7,20.9)	12.2 (9.1,15.4)	9.0 (6.5,11.4)	9.6 (6.8,12.5)	9.3 (6.6,11.9)	8.0 (3.4,12.0)	Urban

(continued to the next page)

Appendix 4. Continued

Gender	Provinces	Districts	Overall	Q1 (lowest)	Q2	Q3	Q4	Q5 (highest)	Q1-Q5	Urbanity
	Gyeongsangnam-do	Uiryeong-gun	16.1 (14.6,17.6)	22.7 (18.5,27.0)	18.0 (14.7,21.3)	13.0 (10.2,15.7)	15.1 (11.8,18.3)	12.7 (9.7,15.7)	10.0 (8.7,18.9)	Rural
	Gyeongsangnam-do	Haman-gun	13.0 (11.7,14.2)	21.5 (17.8,25.2)	12.8 (10.1,15.6)	11.7 (9.1,14.3)	10.2 (7.8,12.6)	8.9 (6.6,11.2)	12.6 (8.4,17.6)	Rural
	Gyeongsangnam-do	Changnyeong-gun	15.0 (13.7,16.4)	23.0 (19.0,27.0)	16.6 (13.5,19.7)	13.5 (10.7,16.3)	11.2 (8.7,13.7)	11.0 (8.4,13.6)	12.0 (7.8,17.4)	Rural
	Gyeongsangnam-do	Goseong-gun	13.3 (12.1,14.6)	24.5 (20.4,28.7)	14.6 (11.5,17.6)	11.3 (8.9,13.8)	8.8 (6.4,11.1)	8.2 (5.9,10.4)	16.3 (10.6,19.6)	Rural
	Gyeongsangnam-do	Namhae-gun	18.6 (17.0,20.1)	28.3 (23.9,32.8)	21.6 (17.8,25.5)	15.9 (12.8,18.9)	15.5 (12.2,18.9)	11.7 (9.0,14.5)	16.6 (11.5,22.1)	Rural
	Gyeongsangnam-do	Hadong-gun	17.7 (16.2,19.2)	25.8 (21.5,30.0)	16.5 (13.4,19.6)	19.3 (15.9,22.8)	14.2 (11.2,17.2)	12.6 (9.7,15.4)	13.2 (5.8,16.0)	Rural
	Gyeongsangnam-do	Sancheong-gun	16.9 (15.4,18.3)	28.1 (23.5,32.7)	18.3 (15.0,21.5)	12.9 (10.2,15.6)	13.7 (10.9,16.6)	11.7 (8.9,14.5)	16.4 (11.7,21.9)	Rural
	Gyeongsangnam-do	Hamyang-gun	16.8 (15.3,18.3)	22.2 (18.3,26.1)	21.6 (17.9,25.4)	15.1 (12.0,18.2)	13.9 (10.9,16.8)	11.1 (8.6,13.7)	11.1 (10.3,20.5)	Rural
	Gyeongsangnam-do	Geochang-gun	15.0 (13.6,16.3)	25.1 (21.0,29.2)	16.7 (13.6,19.9)	12.7 (9.9,15.4)	11.3 (8.7,13.8)	9.5 (7.1,11.9)	15.6 (8.6,18.0)	Rural
	Gyeongsangnam-do	Hapcheon-gun	16.1 (14.7,17.5)	26.4 (21.9,30.9)	18.3 (14.9,21.8)	13.5 (10.6,16.3)	10.2 (7.9,12.4)	13.0 (10.0,16.1)	13.4 (7.3,16.9)	Rural
	Jeju-do	Jeju-si	14.2 (13.3,15.0)	23.8 (21.4,26.3)	15.3 (13.3,17.3)	12.9 (11.0,14.8)	9.9 (8.3,11.5)	9.8 (8.3,11.3)	14.0 (11.1,16.5)	Urban
	Jeju-do	Seogwipo-si	15.1 (14.2,15.9)	22.5 (20.1,24.9)	15.5 (13.6,17.4)	13.7 (11.9,15.4)	12.7 (11.0,14.4)	11.6 (9.9,13.2)	10.9 (6.4,12.0)	Urban
Women	Seoul	Jongno-gu	14.4 (13.2,15.6)	21.7 (18.4,25.1)	14.0 (11.4,16.6)	14.1 (11.4,16.8)	12.3 (9.9,14.8)	10.2 (8.0,12.5)	11.5 (3.8,12.2)	Metropolitan
	Seoul	Jung-gu	15.3 (14.1,16.6)	22.7 (19.2,26.3)	16.8 (13.9,19.8)	14.9 (12.2,17.6)	11.3 (8.9,13.7)	11.2 (8.8,13.6)	11.5 (7.4,16.0)	Metropolitan
	Seoul	Yongsan-gu	11.1 (10.0,12.1)	16.9 (13.8,19.9)	12.1 (9.6,14.6)	8.6 (6.5,10.6)	9.1 (7.0,11.2)	8.9 (6.8,11.1)	8.0 (4.0,11.6)	Metropolitan
	Seoul	Seongdong-gu	14.3 (13.1,15.6)	18.9 (15.6,22.2)	17.1 (14.0,20.2)	13.4 (10.7,16.0)	11.6 (9.0,14.1)	10.6 (8.1,13.0)	8.3 (2.8,10.6)	Metropolitan
	Seoul	Gwangjin-gu	14.1 (12.8,15.4)	18.9 (15.5,22.3)	14.6 (11.6,17.6)	13.2 (10.3,16.0)	13.0 (10.3,15.7)	11.1 (8.5,13.8)	7.8 (3.8,12.0)	Metropolitan
	Seoul	Dongdaemun-gu	16.1 (14.8,17.4)	19.9 (16.6,23.2)	17.6 (14.6,20.6)	16.5 (13.6,19.4)	15.5 (12.6,18.3)	11.3 (8.9,13.7)	8.6 (5.1,13.3)	Metropolitan
	Seoul	Jungnang-gu	14.4 (13.2,15.7)	20.7 (17.3,24.1)	14.9 (12.2,17.6)	13.6 (11.0,16.3)	12.1 (9.5,14.6)	11.1 (8.7,13.6)	9.6 (4.6,12.6)	Metropolitan
	Seoul	Seongbuk-gu	13.0 (11.9,14.2)	18.7 (15.5,21.8)	12.0 (9.5,14.5)	13.9 (11.2,16.5)	11.6 (9.1,14.0)	9.3 (7.1,11.5)	9.4 (4.2,12.0)	Metropolitan
	Seoul	Gangbuk-gu	15.2 (14.0,16.5)	18.8 (15.7,22.0)	18.5 (15.4,21.5)	13.3 (10.7,15.9)	13.2 (10.7,15.8)	12.4 (9.9,14.9)	6.4 (1.5,9.7)	Metropolitan
	Seoul	Dobong-gu	15.5 (14.2,16.7)	20.0 (16.7,23.3)	16.8 (13.9,19.7)	13.9 (11.3,16.5)	12.1 (9.6,14.6)	14.7 (11.9,17.4)	5.3 (1.2,9.6)	Metropolitan
	Seoul	Nowon-gu	16.1 (14.8,17.4)	25.4 (21.7,29.1)	18.9 (15.8,22.0)	13.6 (10.9,16.3)	11.6 (9.2,14.0)	11.0 (8.5,13.5)	14.4 (8.1,16.5)	Metropolitan
	Seoul	Eunpyeong-gu	15.9 (14.6,17.2)	22.1 (18.7,25.6)	17.5 (14.4,20.6)	15.2 (12.4,17.9)	12.2 (9.7,14.6)	12.8 (10.2,15.4)	9.3 (4.5,12.7)	Metropolitan
	Seoul	Seodaemun-gu	15.5 (14.2,16.8)	22.9 (19.4,26.4)	16.4 (13.5,19.4)	12.8 (10.3,15.3)	15.2 (12.4,17.9)	10.7 (8.4,13.0)	12.2 (8.2,16.4)	Metropolitan
	Seoul	Mapo-gu	13.8 (12.6,15.0)	19.1 (15.8,22.3)	17.5 (14.3,20.6)	13.0 (10.3,15.7)	11.0 (8.6,13.5)	8.6 (6.4,10.8)	10.5 (8.3,16.1)	Metropolitan
	Seoul	Yangcheon-gu	15.2 (13.9,16.5)	21.9 (18.3,25.4)	14.3 (11.4,17.2)	13.8 (11.1,16.6)	13.6 (10.8,16.3)	12.8 (10.2,15.3)	9.1 (3.1,11.3)	Metropolitan
	Seoul	Gangseo-gu	14.6 (13.3,15.9)	20.9 (17.4,24.4)	17.4 (14.3,20.6)	14.0 (11.2,16.7)	10.3 (7.9,12.7)	10.9 (8.5,13.4)	10.0 (6.9,15.1)	Metropolitan
	Seoul	Guro-gu	16.0 (14.6,17.3)	22.1 (18.4,25.8)	16.5 (13.5,19.6)	16.4 (13.5,19.4)	12.6 (9.9,15.2)	13.0 (10.2,15.7)	9.1 (4.3,12.9)	Metropolitan
	Seoul	Geumcheon-gu	16.5 (15.2,17.8)	23.2 (19.6,26.8)	18.5 (15.3,21.7)	15.2 (12.4,18.0)	13.3 (10.6,16.0)	12.6 (10.0,15.3)	10.6 (3.8,12.4)	Metropolitan
	Seoul	Yeongdeungpo-gu	15.4 (14.1,16.7)	22.4 (18.8,26.0)	17.0 (13.9,20.0)	13.8 (11.1,16.4)	10.9 (8.5,13.4)	13.0 (10.3,15.8)	9.4 (6.1,14.7)	Metropolitan
	Seoul	Dongjak-gu	16.3 (14.9,17.6)	20.1 (16.8,23.5)	17.9 (14.8,21.1)	14.7 (11.8,17.6)	16.8 (13.8,19.7)	12.0 (9.4,14.5)	8.1 (3.3,11.7)	Metropolitan
	Seoul	Gwanak-gu	16.1 (14.7,17.5)	20.6 (17.0,24.3)	17.0 (13.8,20.2)	16.9 (13.7,20.0)	13.8 (10.9,16.7)	12.5 (9.8,15.2)	8.1 (3.5,11.7)	Metropolitan
	Seoul	Seocho-gu	10.8 (9.7,12.0)	14.4 (11.4,17.4)	13.6 (10.7,16.5)	10.1 (7.7,12.6)	8.4 (6.2,10.5)	8.2 (5.9,10.5)	6.2 (1.9,9.3)	Metropolitan
	Seoul	Gangnam-gu	11.1 (9.9,12.3)	16.9 (13.6,20.2)	12.9 (10.1,15.7)	9.6 (7.1,12.0)	9.1 (6.8,11.3)	7.4 (5.3,9.5)	9.5 (6.2,13.6)	Metropolitan
	Seoul	Songpa-gu	11.8 (10.6,13.0)	13.5 (10.5,16.4)	10.7 (8.1,13.2)	12.4 (9.7,15.1)	11.4 (8.9,13.9)	11.0 (8.4,13.7)	2.5 (0.9,8.3)	Metropolitan
	Seoul	Gangdong-gu	14.5 (13.2,15.8)	17.8 (14.5,21.2)	17.8 (14.6,21.0)	13.7 (10.9,16.4)	12.2 (9.5,15.0)	11.0 (8.4,13.7)	6.8 (3.1,11.1)	Metropolitan
	Busan	Jung-gu	16.5 (15.3,17.8)	21.2 (17.9,24.4)	19.1 (16.2,22.0)	16.9 (14.0,19.7)	14.2 (11.7,16.8)	11.2 (9.0,13.5)	10.0 (4.3,12.7)	Metropolitan
	Busan	Seo-gu	16.0 (14.8,17.3)	24.7 (21.2,28.3)	15.0 (12.4,17.7)	16.1 (13.4,18.8)	13.8 (11.4,16.2)	11.0 (8.7,13.2)	13.7 (7.4,16.2)	Metropolitan
	Busan	Dong-gu	20.4 (19.0,21.8)	31.3 (27.3,35.3)	20.9 (17.8,24.1)	18.9 (16.1,21.8)	16.5 (13.8,19.2)	14.7 (12.1,17.3)	16.6 (10.8,20.0)	Metropolitan
	Busan	Yeongdo-gu	19.1 (17.8,20.4)	27.9 (24.1,31.6)	19.8 (16.8,22.8)	18.2 (15.4,21.0)	15.9 (13.2,18.6)	14.2 (11.8,16.6)	13.7 (8.4,17.8)	Metropolitan
	Busan	Busanjin-gu	17.5 (16.2,18.8)	25.4 (21.9,28.9)	18.1 (15.1,21.2)	15.5 (12.8,18.3)	14.7 (12.1,17.3)	13.9 (11.3,16.5)	11.5 (8.4,17.2)	Metropolitan
	Busan	Dongnae-gu	15.2 (14.0,16.4)	21.9 (18.5,25.2)	16.0 (13.2,18.8)	13.4 (10.8,15.9)	13.9 (11.3,16.5)	10.6 (8.2,13.0)	11.3 (6.6,14.8)	Metropolitan
	Busan	Nam-gu	16.8 (15.5,18.1)	22.1 (18.6,25.6)	18.7 (15.7,21.7)	17.1 (14.2,20.0)	15.0 (12.3,17.7)	11.8 (9.3,14.2)	10.3 (5.9,14.5)	Metropolitan
	Busan	Buk-gu	17.0 (15.6,18.4)	25.8 (21.9,29.8)	17.8 (14.6,20.9)	17.8 (14.6,21.0)	11.9 (9.4,14.5)	12.0 (9.4,14.7)	13.8 (9.9,18.5)	Metropolitan
	Busan	Haeundae-gu	16.7 (15.4,18.0)	28.3 (24.4,32.1)	16.9 (13.9,19.9)	13.9 (11.3,16.5)	14.9 (12.1,17.7)	9.9 (7.6,12.1)	18.4 (14.8,23.6)	Metropolitan
	Busan	Saha-gu	15.8 (14.5,17.0)	23.4 (19.7,27.1)	16.1 (13.2,19.0)	14.4 (11.7,17.2)	12.7 (10.1,15.3)	12.6 (10.1,15.2)	10.8 (7.0,15.8)	Metropolitan
	Busan	Geumjeong-gu	15.0 (13.8,16.2)	22.5 (19.0,26.0)	16.7 (13.9,19.6)	15.9 (12.9,18.8)	12.7 (10.3,15.2)	8.0 (6.1,10.0)	14.5 (8.7,16.9)	Metropolitan
	Busan	Gangseo-gu	18.8 (17.5,20.2)	24.3 (20.7,27.8)	22.0 (18.7,25.2)	16.1 (13.2,18.9)	15.8 (13.0,18.5)	16.1 (13.3,18.9)	8.2 (4.8,13.8)	Metropolitan
	Busan	Yeonje-gu	17.0 (15.6,18.3)	26.7 (23.0,30.4)	17.7 (14.6,20.7)	14.2 (11.5,16.9)	13.1 (10.6,15.7)	13.2 (10.5,15.8)	13.5 (9.1,17.9)	Metropolitan
	Busan	Suyeong-gu	15.7 (14.5,17.0)	21.7 (18.4,25.0)	17.8 (14.9,20.7)	13.7 (11.1,16.3)	12.9 (10.5,15.3)	12.5 (10.1,15.0)	9.2 (4.3,12.9)	Metropolitan
	Busan	Sasang-gu	18.7 (17.2,20.2)	25.4 (21.4,29.4)	20.9 (17.5,24.4)	17.2 (14.1,20.3)	15.3 (12.4,18.3)	15.2 (12.1,18.2)	10.2 (5.4,14.6)	Metropolitan
	Busan	Gijang-gun	19.9 (18.5,21.2)	25.1 (21.6,28.7)	23.9 (20.5,27.3)	18.8 (15.8,21.8)	16.6 (13.8,19.4)	14.8 (12.1,17.5)	10.3 (6.7,15.9)	Rural
	Daegu	Jung-gu	17.7 (16.4,19.0)	26.5 (22.7,30.2)	17.9 (15.0,20.7)	15.7 (13.0,18.4)	14.7 (12.0,17.3)	14.2 (11.5,16.8)	12.3 (8.5,17.7)	Metropolitan
	Daegu	Dong-gu	19.6 (18.2,21.0)	27.2 (23.5,31.0)	18.1 (15.1,21.2)	20.7 (17.5,23.8)	18.0 (15.0,21.0)	14.4 (11.8,17.1)	12.8 (8.3,17.3)	Metropolitan
	Daegu	Seo-gu	20.4 (19.0,21.9)	28.6 (24.6,32.5)	19.7 (16.5,22.8)	18.3 (15.3,21.4)	19.9 (16.7,23.1)	16.0 (13.1,18.8)	12.6 (2.3,11.9)	Metropolitan
	Daegu	Nam-gu	17.8 (16.5,19.1)	24.0 (20.5,27.5)	20.5 (17.3,23.7)	17.4 (14.6,20.2)	14.9 (12.2,17.7)	12.6 (10.1,15.0)	11.4 (8.8,17.4)	Metropolitan
	Daegu	Buk-gu	16.3 (14.9,17.6)	23.5 (19.7,27.2)	16.9 (13.8,20.0)	17.4 (14.2,20.6)	12.4 (9.8,15.1)	11.4 (8.8,13.9)	12.1 (8.9,17.7)	Metropolitan
	Daegu	Suseong-gu	17.8 (16.4,19.2)	27.7 (23.7,31.7)	19.1 (15.9,22.3)	17.2 (14.2,20.2)	13.3 (10.6,15.9)	12.4 (9.8,15.0)	15.3 (10.0,19.0)	Metropolitan
	Daegu	Dalseo-gu	17.9 (16.5,19.4)	24.6 (20.8,28.5)	19.7 (16.3,23.2)	16.9 (13.7,20.1)	15.3 (12.3,18.2)	13.5 (10.6,16.3)	11.1 (7.7,16.5)	Metropolitan

(continued to the next page)

Appendix 4. Continued

Gender	Provinces	Districts	Overall	Q1 (lowest)	Q2	Q3	Q4	Q5 (highest)	Q1-Q5	Urbanity
	Daegu	Dalseong-gun	19.5 (18.0,20.9)	27.5 (23.5,31.5)	19.7 (16.5,23.0)	17.1 (14.1,20.1)	14.7 (11.9,17.5)	18.6 (15.4,21.7)	8.9 (3.9,13.5)	Rural
	Incheon	Jung-gu	16.5 (15.2,17.8)	23.4 (19.8,27.0)	15.3 (12.5,18.1)	16.5 (13.6,19.4)	13.3 (10.8,15.9)	14.2 (11.5,17.0)	9.2 (4.8,13.8)	Metropolitan
	Incheon	Dong-gu	15.5 (14.2,16.7)	20.8 (17.5,24.1)	16.2 (13.4,19.0)	14.9 (12.2,17.6)	13.1 (10.7,15.6)	12.6 (10.1,15.1)	8.2 (3.2,11.8)	Metropolitan
	Incheon	Nam-gu	14.1 (12.9,15.3)	20.0 (16.6,23.4)	14.9 (12.1,17.8)	13.1 (10.5,15.8)	12.2 (9.7,14.6)	10.7 (8.3,13.0)	9.3 (5.8,14.0)	Metropolitan
	Incheon	Yeonsu-gu	15.6 (14.2,17.0)	24.7 (20.7,28.7)	16.0 (12.8,19.2)	14.6 (11.6,17.6)	13.0 (10.2,15.8)	10.0 (7.5,12.5)	14.7 (9.7,17.9)	Metropolitan
	Incheon	Namdong-gu	16.8 (15.4,18.2)	23.3 (19.5,27.0)	17.5 (14.3,20.7)	17.3 (14.2,20.4)	12.4 (9.8,15.1)	13.3 (10.5,16.0)	10.0 (5.3,13.7)	Metropolitan
	Incheon	Bupyeong-gu	17.0 (15.6,18.4)	23.9 (20.0,27.7)	17.9 (14.7,21.1)	16.8 (13.7,20.0)	13.9 (11.2,16.5)	13.3 (10.6,16.1)	10.6 (6.3,14.9)	Metropolitan
	Incheon	Gyeyang-gu	16.8 (15.4,18.3)	23.3 (19.3,27.3)	19.4 (15.9,22.8)	14.1 (11.1,17.0)	14.1 (11.2,17.0)	13.9 (10.9,16.9)	9.4 (4.5,13.1)	Metropolitan
	Incheon	Seo-gu	18.8 (17.3,20.4)	24.1 (20.0,28.1)	19.9 (16.3,23.5)	18.3 (14.8,21.9)	16.7 (13.5,19.9)	15.3 (12.0,18.5)	8.8 (2.4,12.0)	Metropolitan
	Incheon	Ganghwa-gun	18.4 (17.1,19.8)	23.3 (19.7,26.9)	19.1 (16.0,22.2)	19.1 (16.1,22.2)	15.2 (12.6,17.8)	15.6 (12.8,18.5)	7.7 (2.6,11.6)	Rural
	Incheon	Ongjin-gun	13.8 (12.6,15.0)	16.5 (13.6,19.4)	15.1 (12.4,17.9)	12.0 (9.6,14.5)	12.1 (9.8,14.4)	13.3 (10.6,16.0)	3.2 (0.8,9.8)	Rural
	Gwangju	Dong-gu	16.4 (15.1,17.6)	24.9 (21.2,28.5)	17.7 (14.8,20.6)	16.4 (13.6,19.2)	12.5 (10.2,14.8)	11.5 (9.2,13.9)	13.4 (9.4,18.0)	Metropolitan
	Gwangju	Seo-gu	15.9 (14.6,17.3)	23.1 (19.4,26.8)	17.5 (14.3,20.6)	12.3 (9.6,15.0)	14.9 (12.0,17.8)	12.4 (9.8,15.1)	10.7 (4.8,13.6)	Metropolitan
	Gwangju	Nam-gu	17.1 (15.8,18.5)	24.4 (20.7,28.1)	17.6 (14.5,20.7)	17.2 (14.4,20.0)	14.4 (11.7,17.1)	12.3 (9.9,14.7)	12.1 (8.4,17.0)	Metropolitan
	Gwangju	Buk-gu	17.3 (15.9,18.7)	26.1 (22.3,29.9)	16.4 (13.4,19.4)	18.2 (15.1,21.4)	14.7 (11.8,17.5)	11.6 (9.0,14.1)	14.5 (6.9,15.3)	Metropolitan
	Gwangju	Gwangsan-gu	17.9 (16.4,19.5)	26.5 (22.2,30.9)	18.8 (15.4,22.2)	17.1 (13.8,20.5)	14.5 (11.4,17.5)	13.2 (10.3,16.2)	13.3 (5.6,14.6)	Metropolitan
	Daejeon	Dong-gu	16.4 (15.1,17.7)	25.3 (21.5,29.2)	18.9 (15.8,21.9)	13.9 (11.3,16.6)	13.9 (11.3,16.5)	10.9 (8.6,13.3)	14.4 (9.4,18.0)	Metropolitan
	Daejeon	Jung-gu	14.7 (13.4,15.9)	22.2 (18.7,25.8)	14.1 (11.5,16.7)	14.6 (11.8,17.3)	13.0 (10.4,15.5)	10.4 (8.1,12.6)	11.8 (6.9,15.3)	Metropolitan
	Daejeon	Seo-gu	16.0 (14.6,17.4)	23.2 (19.2,27.1)	16.3 (13.2,19.5)	14.2 (11.2,17.2)	14.3 (11.3,17.2)	12.3 (9.5,15.2)	10.9 (8.6,17.2)	Metropolitan
	Daejeon	Yuseong-gu	13.7 (12.4,15.1)	18.3 (14.7,21.9)	16.9 (13.4,20.3)	10.9 (8.2,13.5)	13.0 (10.1,16.0)	9.4 (6.7,12.1)	8.9 (5.6,13.8)	Metropolitan
	Daejeon	Daedeok-gu	16.2 (14.8,17.5)	21.4 (17.8,25.0)	17.5 (14.4,20.6)	14.2 (11.3,17.0)	14.5 (11.6,17.4)	13.0 (10.2,15.8)	8.4 (3.5,11.9)	Metropolitan
	Ulsan	Jung-gu	17.1 (15.6,18.5)	22.9 (19.2,26.7)	17.3 (14.1,20.4)	15.6 (12.6,18.7)	14.7 (11.9,17.6)	15.1 (12.0,18.2)	7.8 (2.6,11.4)	Metropolitan
	Ulsan	Nam-gu	15.1 (13.7,16.6)	21.2 (17.0,25.4)	15.6 (12.4,18.8)	13.4 (10.2,16.5)	13.3 (10.4,16.2)	13.0 (9.8,16.2)	8.2 (5.2,13.6)	Metropolitan
	Ulsan	Dong-gu	14.6 (13.1,16.0)	21.2 (17.1,25.3)	17.4 (13.7,21.0)	13.1 (10.0,16.2)	10.9 (8.1,13.7)	10.4 (7.5,13.3)	10.8 (6.8,15.2)	Metropolitan
	Ulsan	Buk-gu	13.8 (12.4,15.3)	16.8 (13.0,20.6)	16.0 (12.6,19.4)	15.4 (12.0,18.8)	12.1 (9.1,15.1)	8.8 (6.2,11.4)	8.0 (5.5,13.3)	Metropolitan
	Ulsan	Ulju-gun	19.1 (17.7,20.5)	26.3 (22.4,30.1)	20.4 (17.1,23.6)	17.4 (14.4,20.5)	16.0 (13.2,18.8)	16.1 (13.2,19.0)	10.2 (7.0,16.2)	Rural
	Sejong	Sejong	18.3 (16.9,19.6)	23.5 (20.0,27.0)	20.7 (17.5,23.9)	18.1 (15.1,21.0)	15.4 (12.7,18.0)	14.1 (11.5,16.6)	9.4 (5.1,13.9)	Metropolitan
	Gyeonggi-do	Jangan-gu	15.6 (14.3,17.0)	18.6 (15.3,21.9)	18.5 (15.2,21.7)	14.0 (11.1,16.9)	14.7 (11.8,17.6)	12.0 (9.3,14.7)	6.6 (2.5,10.9)	Metropolitan
	Gyeonggi-do	Gwonseon-gu	16.4 (15.0,17.9)	22.5 (18.6,26.4)	17.5 (14.1,20.8)	15.1 (12.0,18.1)	14.0 (11.0,17.0)	13.3 (10.3,16.3)	9.2 (3.0,12.0)	Metropolitan
	Gyeonggi-do	Paldal-gu	16.4 (15.0,17.8)	23.7 (19.9,27.5)	17.7 (14.4,20.9)	14.4 (11.6,17.2)	13.4 (10.7,16.2)	13.4 (10.5,16.3)	10.3 (7.0,16.0)	Metropolitan
	Gyeonggi-do	Yeongtong-gu	12.5 (11.0,13.9)	17.3 (13.4,21.3)	11.0 (8.1,13.9)	10.4 (7.4,13.4)	12.5 (9.1,15.8)	11.5 (8.3,14.6)	5.8 (1.0,9.4)	Metropolitan
	Gyeonggi-do	Sujeong-gu	17.2 (15.8,18.6)	23.3 (19.6,27.1)	18.7 (15.4,21.9)	15.1 (12.1,18.0)	16.2 (13.2,19.2)	13.0 (10.1,15.8)	10.3 (7.2,16.2)	Metropolitan
	Gyeonggi-do	Jungwon-gu	18.9 (17.4,20.4)	24.8 (20.9,28.7)	20.6 (17.2,24.1)	17.1 (13.9,20.3)	16.3 (13.3,19.4)	16.1 (12.9,19.2)	8.7 (5.8,15.2)	Metropolitan
	Gyeonggi-do	Bundang-gu	9.6 (8.5,10.6)	12.6 (9.8,15.3)	10.7 (8.2,13.3)	6.9 (4.9,8.9)	9.1 (6.8,11.4)	8.5 (6.2,10.8)	4.1 (0.6,7.4)	Metropolitan
	Gyeonggi-do	Uijeongbu-si	16.8 (15.5,18.1)	25.5 (21.7,29.3)	19.9 (16.6,23.1)	14.0 (11.3,16.6)	13.3 (10.7,15.9)	12.3 (9.8,14.8)	13.2 (8.8,17.4)	Urban
	Gyeonggi-do	Manan-gu	15.8 (14.4,17.1)	21.6 (18.0,25.2)	15.8 (12.8,18.8)	14.4 (11.6,17.1)	13.9 (11.2,16.7)	13.6 (10.9,16.3)	8.0 (5.4,14.0)	Metropolitan
	Gyeonggi-do	Dongan-gu	13.6 (12.3,14.9)	17.4 (14.1,20.7)	14.7 (11.6,17.7)	13.4 (10.6,16.1)	11.2 (8.6,13.8)	11.4 (8.8,14.1)	6.0 (1.2,9.2)	Metropolitan
	Gyeonggi-do	Wonmi-gu	16.3 (14.8,17.7)	22.4 (18.5,26.3)	18.2 (14.6,21.9)	14.9 (12.0,17.9)	13.8 (10.8,16.8)	12.6 (9.7,15.4)	9.8 (4.0,12.6)	Metropolitan
	Gyeonggi-do	Sosa-gu	16.0 (14.6,17.3)	21.6 (17.9,25.3)	16.4 (13.2,19.5)	15.7 (12.7,18.7)	14.3 (11.5,17.2)	12.1 (9.5,14.7)	9.5 (5.9,14.7)	Metropolitan
	Gyeonggi-do	Ojeong-gu	16.9 (15.5,18.3)	25.0 (21.0,28.9)	18.4 (15.2,21.6)	12.9 (10.2,15.6)	13.0 (10.3,15.8)	15.4 (12.4,18.5)	9.6 (5.0,14.0)	Metropolitan
	Gyeonggi-do	Gwangmyeong-si	15.8 (14.4,17.2)	22.0 (18.2,25.7)	17.8 (14.7,21.0)	12.5 (9.8,15.3)	11.9 (9.2,14.5)	15.1 (12.1,18.1)	6.9 (5.0,13.8)	Urban
	Gyeonggi-do	Pyeongtaek-si	17.0 (16.0,18.0)	24.2 (21.6,26.8)	16.9 (14.8,19.1)	15.9 (13.8,18.0)	14.7 (12.7,16.7)	13.4 (11.6,15.3)	10.8 (8.2,14.6)	Urban
	Gyeonggi-do	Dongducheon-si	18.7 (17.3,20.1)	27.1 (23.3,31.0)	19.2 (16.0,22.4)	16.9 (13.9,19.8)	17.2 (14.3,20.2)	13.4 (10.8,16.0)	13.7 (8.4,17.6)	Urban
	Gyeonggi-do	Sangnok-gu	15.7 (14.2,17.1)	21.3 (17.6,25.1)	17.6 (14.2,21.1)	15.0 (11.9,18.0)	12.6 (9.8,15.4)	12.0 (9.2,14.8)	9.3 (7.4,15.8)	Metropolitan
	Gyeonggi-do	Danwon-gu	17.9 (16.3,19.4)	26.3 (21.9,30.7)	17.5 (14.1,21.0)	16.8 (13.5,20.2)	14.7 (11.5,18.0)	14.4 (11.2,17.5)	11.9 (7.9,17.1)	Metropolitan
	Gyeonggi-do	Deogyang-gu	17.2 (15.9,18.6)	24.4 (20.7,28.2)	18.9 (15.8,22.0)	15.9 (12.9,18.9)	13.9 (11.2,16.6)	13.0 (10.3,15.7)	11.4 (4.7,13.3)	Metropolitan
	Gyeonggi-do	Ilsandong-gu	14.8 (13.4,16.1)	19.2 (15.7,22.6)	15.8 (12.7,18.8)	14.8 (11.9,17.7)	13.4 (10.7,16.1)	10.8 (8.3,13.4)	8.4 (3.6,11.6)	Metropolitan
	Gyeonggi-do	Ilsanseo-gu	13.4 (12.2,14.7)	19.3 (15.9,22.8)	13.2 (10.4,16.0)	10.5 (8.0,13.1)	11.0 (8.5,13.4)	12.9 (10.1,15.7)	6.4 (-0.1,8.1)	Metropolitan
	Gyeonggi-do	Gwacheon-si	14.2 (13.0,15.5)	19.5 (16.1,22.9)	14.1 (11.3,16.9)	13.6 (10.9,16.3)	12.3 (9.7,15.0)	11.9 (9.3,14.5)	7.6 (2.8,11.2)	Urban
	Gyeonggi-do	Guri-si	16.9 (15.5,18.3)	22.6 (19.0,26.3)	16.8 (13.6,19.9)	17.1 (13.9,20.3)	15.9 (12.8,18.9)	11.8 (9.1,14.5)	10.8 (3.7,12.5)	Urban
	Gyeonggi-do	Namyangju-si	16.0 (14.7,17.4)	20.0 (16.6,23.3)	17.6 (14.4,20.7)	14.5 (11.8,17.3)	13.4 (10.7,16.1)	15.3 (12.3,18.2)	4.7 (2.1,10.7)	Urban
	Gyeonggi-do	Osan-si	15.8 (14.3,17.3)	18.8 (15.1,22.5)	15.5 (12.0,19.0)	15.4 (12.2,18.5)	15.8 (12.4,19.1)	13.8 (10.5,17.0)	5.0 (-0.3,8.5)	Urban
	Gyeonggi-do	Siheung-si	17.4 (15.8,18.9)	20.6 (16.8,24.5)	18.9 (15.5,22.4)	17.1 (13.8,20.4)	17.4 (14.0,20.8)	12.4 (9.4,15.3)	8.2 (2.3,11.1)	Urban
	Gyeonggi-do	Gunpo-si	16.5 (15.1,17.9)	24.7 (20.8,28.6)	15.4 (12.4,18.5)	14.1 (11.3,17.0)	14.5 (11.6,17.4)	13.7 (10.7,16.6)	11.0 (6.7,15.5)	Urban
	Gyeonggi-do	Uiwang-si	15.0 (13.6,16.3)	21.1 (17.5,24.8)	16.0 (12.9,19.1)	12.6 (9.9,15.3)	12.5 (9.8,15.2)	12.8 (10.1,15.6)	8.3 (4.3,12.9)	Urban
	Gyeonggi-do	Hanam-si	16.0 (14.7,17.4)	19.6 (16.2,23.0)	17.5 (14.4,20.7)	15.4 (12.5,18.3)	13.8 (11.1,16.4)	14.4 (11.6,17.2)	5.2 (3.0,11.6)	Urban
	Gyeonggi-do	Cheoin-gu	15.5 (14.2,16.8)	21.3 (17.8,24.8)	15.0 (12.2,17.9)	16.8 (13.7,19.9)	13.1 (10.5,15.8)	11.4 (8.9,14.0)	9.9 (1.6,10.0)	Metropolitan
	Gyeonggi-do	Giheung-gu	13.1 (11.8,14.3)	18.0 (14.7,21.4)	12.9 (10.1,15.7)	12.4 (9.6,15.1)	11.7 (9.0,14.4)	10.3 (7.8,12.9)	7.7 (4.0,11.8)	Metropolitan
	Gyeonggi-do	Suji-gu	11.6 (10.4,12.7)	13.5 (10.5,16.4)	10.5 (8.0,12.9)	13.0 (10.1,15.8)	9.9 (7.4,12.4)	11.6 (9.0,14.2)	1.9 (-2.2,5.2)	Metropolitan
	Gyeonggi-do	Paju-si	16.5 (15.1,17.8)	23.5 (19.8,27.2)	17.1 (14.1,20.2)	15.2 (12.4,18.0)	12.1 (9.6,14.6)	14.9 (12.1,17.7)	8.6 (3.2,12.0)	Urban

(continued to the next page)

Appendix 4. Continued

Gender	Provinces	Districts	Overall	Q1 (lowest)	Q2	Q3	Q4	Q5 (highest)	Q1-Q5	Urbanity
	Gyeonggi-do	Icheon-si	17.6 (16.2,19.0)	25.0 (21.2,28.8)	19.4 (16.2,22.7)	14.3 (11.5,17.1)	15.3 (12.4,18.3)	13.9 (11.1,16.7)	11.1 (6.4,15.4)	Urban
	Gyeonggi-do	Anseong-si	17.5 (16.1,18.8)	22.9 (19.5,26.3)	17.9 (14.9,20.9)	16.0 (13.2,18.9)	16.1 (13.3,19.0)	14.4 (11.7,17.2)	8.5 (3.8,12.4)	Urban
	Gyeonggi-do	Gimpo-si	13.1 (11.9,14.3)	15.6 (12.6,18.7)	14.3 (11.4,17.1)	12.2 (9.6,14.8)	12.5 (9.9,15.1)	11.2 (8.7,13.7)	4.4 (-0.5,7.3)	Urban
	Gyeonggi-do	Hwaseongsi	15.3 (13.9,16.7)	21.3 (17.7,24.9)	16.3 (13.1,19.4)	14.6 (11.6,17.6)	14.8 (11.9,17.8)	9.5 (7.1,12.0)	11.8 (8.1,16.3)	Urban
	Gyeonggi-do	Gwangju-si	14.2 (12.9,15.5)	18.5 (15.2,21.9)	15.6 (12.7,18.6)	13.6 (10.8,16.4)	11.0 (8.5,13.4)	12.2 (9.5,14.9)	6.3 (2.3,10.7)	Urban
	Gyeonggi-do	Yangju-si	17.4 (16.0,18.8)	22.6 (18.9,26.3)	19.1 (15.8,22.3)	15.6 (12.7,18.4)	16.6 (13.7,19.4)	13.7 (10.9,16.5)	8.9 (5.3,14.3)	Urban
	Gyeonggi-do	Pocheon-si	18.6 (17.2,19.9)	24.5 (20.9,28.1)	21.3 (18.0,24.7)	16.5 (13.6,19.4)	16.8 (14.0,19.6)	13.8 (11.2,16.5)	10.7 (4.7,13.7)	Urban
	Gyeonggi-do	Yeoju-gun	18.8 (17.5,20.2)	22.4 (19.0,25.9)	22.0 (18.7,25.2)	17.8 (14.9,20.8)	16.0 (13.2,18.8)	15.9 (13.1,18.8)	6.5 (1.6,10.4)	Urban
	Gyeonggi-do	Yeoncheon-gun	17.4 (16.1,18.7)	23.0 (19.6,26.5)	18.0 (15.1,20.8)	18.3 (15.4,21.3)	16.0 (13.3,18.7)	11.7 (9.5,14.0)	11.3 (7.7,16.5)	Rural
	Gyeonggi-do	Gapyeong-gun	16.7 (15.5,18.0)	24.1 (20.6,27.6)	19.0 (16.0,22.0)	15.3 (12.6,18.0)	12.6 (10.2,14.9)	12.4 (10.0,14.9)	11.7 (8.4,17.2)	Rural
	Gyeonggi-do	Yangpyeong-gun	18.2 (16.8,19.5)	21.6 (18.3,24.9)	20.2 (17.1,23.3)	18.0 (15.1,21.0)	16.9 (14.1,19.8)	13.9 (11.3,16.5)	7.7 (2.7,11.5)	Rural
	Gangwon-do	Chuncheon-si	15.5 (14.2,16.7)	22.5 (19.0,26.0)	17.5 (14.6,20.5)	13.8 (11.2,16.4)	13.4 (10.9,15.9)	10.7 (8.4,13.0)	11.8 (7.9,16.3)	Urban
	Gangwon-do	Wonju-si	17.2 (15.8,18.6)	24.0 (20.3,27.8)	21.2 (17.8,24.5)	13.9 (11.2,16.6)	14.9 (12.1,17.8)	12.2 (9.6,14.8)	11.8 (7.0,16.2)	Urban
	Gangwon-do	Gangneung-si	16.5 (15.3,17.8)	22.9 (19.5,26.4)	18.5 (15.6,21.4)	14.3 (11.7,16.9)	14.2 (11.6,16.7)	13.3 (10.6,15.9)	9.6 (6.3,15.1)	Urban
	Gangwon-do	Donghae-si	17.5 (16.2,18.8)	24.4 (20.8,28.1)	19.4 (16.4,22.3)	16.5 (13.6,19.4)	14.6 (12.0,17.2)	12.7 (10.2,15.2)	11.7 (8.5,17.3)	Urban
	Gangwon-do	Taebaek-si	21.8 (20.4,23.3)	30.2 (26.2,34.1)	23.9 (20.4,27.4)	21.6 (18.5,24.8)	17.3 (14.4,20.2)	16.2 (13.4,19.0)	14.0 (10.4,20.2)	Urban
	Gangwon-do	Sokcho-si	19.1 (17.7,20.5)	27.1 (23.4,30.8)	19.3 (16.2,22.3)	16.5 (13.7,19.2)	15.5 (12.8,18.2)	17.4 (14.5,20.4)	9.7 (4.8,14.0)	Urban
	Gangwon-do	Samcheok-si	19.2 (17.8,20.6)	28.5 (24.5,32.4)	19.5 (16.5,22.6)	19.9 (16.8,23.0)	15.1 (12.4,17.8)	13.6 (11.0,16.2)	14.9 (9.8,19.2)	Urban
	Gangwon-do	Hongcheon-gun	17.3 (16.0,18.6)	24.5 (20.8,28.2)	19.9 (16.9,23.0)	15.2 (12.7,17.8)	13.2 (10.7,15.7)	14.4 (11.7,17.0)	10.1 (5.4,14.8)	Rural
	Gangwon-do	Hoengseong-gun	20.1 (18.6,21.6)	27.2 (23.2,31.2)	21.5 (18.3,24.8)	19.7 (16.5,22.8)	17.7 (14.6,20.8)	14.6 (11.8,17.4)	12.6 (7.4,16.8)	Rural
	Gangwon-do	Yeongwol-gun	21.6 (20.1,23.0)	30.9 (26.6,35.2)	23.9 (20.6,27.2)	18.4 (15.5,21.2)	18.4 (15.5,21.3)	16.6 (13.8,19.3)	14.3 (9.0,19.2)	Rural
	Gangwon-do	Pyeongchang-gun	17.2 (15.9,18.4)	24.1 (20.6,27.6)	18.1 (15.4,20.9)	14.8 (12.2,17.3)	16.5 (13.7,19.3)	12.5 (10.2,14.9)	11.6 (5.8,15.0)	Rural
	Gangwon-do	Jeongseon-gun	21.5 (20.0,22.9)	28.7 (24.8,32.6)	21.7 (18.6,24.8)	21.8 (18.5,25.1)	20.6 (17.5,23.7)	14.8 (12.2,17.4)	13.9 (9.3,18.9)	Rural
	Gangwon-do	Cheorwon-gun	16.8 (15.5,18.1)	23.8 (20.3,27.4)	17.9 (15.0,20.7)	16.5 (13.7,19.4)	13.5 (11.0,16.1)	12.2 (9.7,14.7)	11.6 (9.5,18.5)	Rural
	Gangwon-do	Hwacheon-gun	16.7 (15.4,18.0)	22.7 (19.0,26.3)	18.3 (15.3,21.3)	15.1 (12.4,17.7)	13.9 (11.2,16.5)	13.7 (11.1,16.3)	9.0 (7.1,16.3)	Rural
	Gangwon-do	Yanggu-gun	17.7 (16.4,19.0)	23.2 (19.6,26.7)	19.2 (16.1,22.4)	15.8 (13.2,18.4)	14.4 (11.8,17.0)	16.3 (13.5,19.1)	6.9 (2.4,11.6)	Rural
	Gangwon-do	Inje-gun	17.8 (16.4,19.1)	26.3 (22.5,30.1)	17.2 (14.2,20.2)	15.7 (12.9,18.4)	16.1 (13.2,19.0)	13.5 (10.8,16.1)	12.8 (8.3,17.5)	Rural
	Gangwon-do	Goseong-gun	16.7 (15.4,18.0)	22.1 (18.5,25.7)	17.1 (14.2,20.0)	16.1 (13.3,18.9)	15.8 (12.9,18.6)	12.6 (10.0,15.1)	9.5 (5.2,13.8)	Rural
	Gangwon-do	Yangyang-gun	16.2 (15.0,17.5)	22.5 (19.1,25.9)	17.8 (14.9,20.6)	15.8 (13.1,18.5)	14.7 (12.1,17.3)	10.6 (8.4,12.7)	11.9 (7.8,16.4)	Rural
	Chungcheongbuk-do	Cheongju-si	15.9 (15.1,16.6)	23.4 (21.2,25.5)	17.8 (15.9,19.7)	14.1 (12.5,15.7)	12.9 (11.4,14.4)	12.0 (10.5,13.4)	11.4 (8.2,13.2)	Metropolitan
	Chungcheongbuk-do	Chungju-si	19.3 (17.9,20.7)	26.1 (22.5,29.8)	22.2 (18.9,25.4)	18.8 (15.7,21.9)	15.0 (12.3,17.7)	14.8 (12.1,17.6)	11.3 (7.0,16.0)	Urban
	Chungcheongbuk-do	Jecheon-si	17.1 (15.8,18.4)	23.5 (20.0,27.0)	17.3 (14.5,20.2)	17.5 (14.6,20.3)	14.6 (12.0,17.2)	13.0 (10.4,15.6)	10.5 (6.8,15.8)	Urban
	Chungcheongbuk-do	Boeun-gun	20.2 (18.8,21.6)	28.7 (24.4,33.0)	22.6 (19.3,26.0)	17.9 (15.1,20.7)	18.2 (15.2,21.3)	14.6 (11.9,17.3)	14.1 (10.3,19.9)	Rural
	Chungcheongbuk-do	Okcheon-gun	18.3 (17.0,19.7)	22.2 (18.8,25.6)	19.9 (16.9,22.8)	18.6 (15.6,21.5)	16.1 (13.5,18.7)	14.9 (12.2,17.6)	7.3 (3.3,12.3)	Rural
	Chungcheongbuk-do	Yeongdong-gun	18.8 (17.5,20.2)	27.1 (23.3,31.0)	20.2 (17.1,23.2)	17.2 (14.4,19.9)	15.0 (12.5,17.5)	14.8 (12.0,17.5)	12.3 (6.9,16.1)	Rural
	Chungcheongbuk-do	Jincheon-gun	19.0 (17.6,20.4)	26.2 (22.5,30.0)	18.5 (15.3,21.6)	18.7 (15.7,21.8)	14.9 (12.2,17.6)	16.9 (14.0,19.8)	9.3 (4.6,14.4)	Rural
	Chungcheongbuk-do	Goesan-gun	18.9 (17.5,20.3)	25.2 (21.5,28.9)	19.0 (15.9,22.1)	17.9 (14.9,20.8)	17.3 (14.5,20.2)	15.3 (12.5,18.1)	9.9 (8.4,17.6)	Rural
	Chungcheongbuk-do	Eumseong-gun	20.1 (18.6,21.5)	27.7 (23.8,31.6)	23.6 (19.9,27.2)	19.2 (16.2,22.2)	15.6 (13.1,18.1)	14.5 (11.8,17.2)	13.2 (9.4,19.0)	Rural
	Chungcheongbuk-do	Danyang-gun	20.1 (18.7,21.6)	30.2 (26.2,34.3)	19.3 (16.1,22.4)	18.0 (15.0,21.0)	17.2 (14.5,20.0)	16.8 (13.9,19.7)	13.4 (7.6,17.2)	Rural
	Chungcheongbuk-do	Jeungpyeong-gun	16.4 (15.2,17.6)	20.4 (17.3,23.6)	16.8 (14.2,19.4)	16.0 (13.4,18.5)	15.3 (12.7,18.0)	13.2 (10.7,15.6)	7.2 (4.7,13.3)	Rural
	Chungcheongnam-do	Cheonan-si	17.6 (16.1,19.1)	25.3 (21.1,29.5)	19.1 (15.6,22.5)	16.3 (13.2,19.4)	14.1 (11.1,17.0)	14.2 (11.2,17.2)	11.1 (5.9,15.3)	Metropolitan
	Chungcheongnam-do	Gongju-si	19.7 (18.3,21.0)	25.5 (21.8,29.2)	19.0 (16.0,21.9)	20.0 (17.0,23.1)	18.5 (15.6,21.5)	15.3 (12.5,18.1)	10.2 (5.2,14.6)	Urban
	Chungcheongnam-do	Boryeong-si	22.0 (20.5,23.5)	31.3 (27.1,35.5)	25.6 (21.9,29.3)	17.6 (14.9,20.4)	20.0 (16.9,23.1)	15.9 (13.2,18.6)	15.4 (10.1,19.9)	Urban
	Chungcheongnam-do	Asan-si	18.4 (17.0,19.9)	26.5 (22.4,30.6)	20.3 (17.0,23.6)	16.5 (13.5,19.6)	14.4 (11.5,17.2)	14.5 (11.6,17.3)	12.0 (8.6,17.8)	Urban
	Chungcheongnam-do	Seosan-si	18.1 (16.8,19.5)	22.9 (19.4,26.3)	20.1 (16.9,23.3)	16.2 (13.4,19.1)	16.6 (13.7,19.6)	15.1 (12.4,17.8)	7.8 (3.1,12.3)	Urban
	Chungcheongnam-do	Nonsan-si	18.7 (17.4,20.0)	27.2 (23.4,31.0)	18.2 (15.6,20.9)	17.1 (14.4,19.8)	16.9 (14.1,19.7)	13.9 (11.4,16.3)	13.3 (8.2,17.2)	Urban
	Chungcheongnam-do	Gyeryong-si	15.9 (14.4,17.3)	21.0 (17.4,24.7)	18.2 (14.8,21.6)	12.7 (9.9,15.6)	14.8 (11.8,17.8)	12.6 (9.7,15.4)	8.4 (2.3,11.1)	Urban
	Chungcheongnam-do	Dangjin-si	18.9 (17.5,20.3)	25.7 (21.9,29.5)	20.8 (17.5,24.0)	19.4 (16.2,22.6)	14.7 (12.0,17.3)	14.3 (11.6,17.1)	11.4 (4.9,14.1)	Rural
	Chungcheongnam-do	Geumsan-gun	18.6 (17.3,19.9)	24.3 (20.7,28.0)	21.2 (18.2,24.2)	17.1 (14.5,19.7)	16.4 (13.7,19.1)	13.8 (11.5,16.2)	10.5 (5.4,14.4)	Rural
	Chungcheongnam-do	Buyeo-gun	17.4 (16.2,18.7)	22.0 (18.5,25.4)	19.5 (16.5,22.5)	16.3 (13.7,18.9)	16.5 (13.8,19.2)	13.2 (10.7,15.7)	8.8 (3.0,11.4)	Rural
	Chungcheongnam-do	Seocheon-gun	18.4 (17.0,19.7)	25.7 (21.9,29.5)	17.4 (14.5,20.2)	16.6 (13.9,19.3)	19.0 (15.9,22.0)	13.4 (11.0,15.8)	12.3 (6.6,15.4)	Rural
	Chungcheongnam-do	Cheongyang-gun	16.9 (15.6,18.2)	22.3 (18.8,25.8)	18.8 (15.8,21.8)	18.8 (15.7,21.8)	13.2 (10.7,15.7)	11.1 (9.0,13.3)	11.2 (7.0,15.6)	Rural
	Chungcheongnam-do	Hongseong-gun	18.3 (17.0,19.6)	22.0 (18.7,25.3)	20.9 (17.8,24.1)	16.7 (14.0,19.4)	17.0 (14.2,19.8)	14.9 (12.3,17.6)	7.1 (5.0,14.0)	Rural
	Chungcheongnam-do	Yesan-gun	17.9 (16.6,19.3)	26.3 (22.5,30.2)	19.9 (16.9,23.0)	14.6 (12.2,17.1)	15.2 (12.6,17.8)	14.3 (11.5,17.0)	12.0 (8.7,18.1)	Rural
	Chungcheongnam-do	Taean-gun	17.9 (16.6,19.2)	23.0 (19.6,26.5)	20.4 (17.4,23.5)	17.9 (15.0,20.9)	15.3 (12.6,18.1)	12.9 (10.4,15.4)	10.1 (5.5,14.3)	Urban
	Jeollabuk-do	Jeonju-si	15.8 (14.5,17.1)	23.8 (20.1,27.5)	16.6 (13.7,19.6)	14.7 (11.9,17.6)	13.0 (10.4,15.6)	11.7 (9.2,14.2)	12.1 (9.1,17.5)	Metropolitan
	Jeollabuk-do	Gunsan-si	20.1 (18.6,21.5)	29.4 (25.4,33.5)	21.4 (18.1,24.6)	17.3 (14.3,20.2)	18.1 (15.0,21.1)	14.6 (11.9,17.2)	14.8 (9.0,18.4)	Urban
	Jeollabuk-do	Iksan-si	20.8 (19.4,22.3)	31.6 (27.6,35.6)	20.8 (17.6,24.0)	19.1 (16.0,22.1)	17.3 (14.4,20.2)	15.7 (13.0,18.4)	15.9 (11.0,20.6)	Urban
	Jeollabuk-do	Jeongeup-si	20.6 (19.2,22.0)	31.9 (27.7,36.0)	19.1 (16.2,22.0)	20.0 (17.0,22.9)	15.5 (12.8,18.1)	17.0 (14.2,19.8)	14.9 (10.0,19.2)	Urban
	Jeollabuk-do	Namwon-si	20.7 (19.3,22.1)	27.1 (23.4,30.7)	23.0 (19.7,26.3)	19.4 (16.4,22.4)	16.4 (13.8,19.1)	17.6 (14.8,20.3)	9.5 (6.9,16.1)	Urban

(continued to the next page)

Appendix 4. Continued

Gender	Provinces	Districts	Overall	Q1 (lowest)	Q2	Q3	Q4	Q5 (highest)	Q1-Q5	Urbanity
	Jeollabuk-do	Gimje-si	19.0 (17.7,20.4)	26.9 (23.0,30.8)	18.9 (16.0,21.8)	16.7 (14.0,19.3)	17.8 (14.8,20.8)	14.9 (12.3,17.5)	12.0 (6.4,15.8)	Urban
	Jeollabuk-do	Wanju-gun	19.3 (18.0,20.7)	29.1 (25.1,33.1)	20.2 (17.2,23.2)	18.2 (15.4,21.1)	15.1 (12.5,17.8)	14.6 (12.1,17.1)	14.5 (7.7,17.1)	Rural
	Jeollabuk-do	Jinan-gun	18.4 (17.0,19.8)	25.4 (21.4,29.5)	22.5 (19.1,26.0)	15.0 (12.7,17.4)	17.4 (14.3,20.4)	11.8 (9.5,14.2)	13.6 (8.1,17.1)	Rural
	Jeollabuk-do	Muju-gun	21.4 (19.9,22.8)	26.8 (23.0,30.7)	25.1 (21.4,28.8)	19.1 (16.3,22.0)	18.4 (15.4,21.3)	17.1 (14.1,20.1)	9.7 (3.2,12.8)	Rural
	Jeollabuk-do	Jangsu-gun	19.8 (18.4,21.2)	24.3 (20.6,28.1)	21.5 (18.3,24.7)	18.4 (15.5,21.3)	15.8 (13.1,18.4)	19.4 (16.2,22.6)	4.9 (2.5,11.9)	Rural
	Jeollabuk-do	Imsil-gun	19.5 (18.1,20.9)	23.9 (20.0,27.8)	19.0 (16.1,21.9)	27.3 (23.5,31.1)	13.4 (10.9,16.0)	12.6 (10.3,14.9)	11.3 (7.9,16.7)	Rural
	Jeollabuk-do	Sunchang-gun	19.6 (18.2,21.0)	27.5 (23.6,31.5)	21.7 (18.3,25.0)	17.9 (15.2,20.6)	15.5 (12.9,18.2)	14.8 (12.1,17.5)	12.7 (6.3,15.5)	Rural
	Jeollabuk-do	Gochang-gun	24.4 (22.9,26.0)	31.4 (27.2,35.6)	23.8 (20.6,27.1)	23.1 (19.8,26.5)	23.8 (20.4,27.2)	20.6 (17.3,23.8)	10.8 (5.8,15.4)	Rural
	Jeollabuk-do	Buan-gun	21.1 (19.6,22.5)	32.0 (27.6,36.4)	20.4 (17.5,23.3)	19.9 (16.8,23.1)	15.2 (12.6,17.8)	18.3 (15.2,21.3)	13.7 (8.6,18.2)	Rural
	Jeollanam-do	Mokpo-si	19.5 (18.1,21.0)	26.5 (22.7,30.3)	22.0 (18.6,25.4)	19.8 (16.6,23.0)	15.9 (13.1,18.7)	13.4 (10.8,16.1)	13.1 (6.0,15.0)	Urban
	Jeollanam-do	Yeosu-si	17.9 (16.6,19.3)	23.3 (19.9,26.8)	17.4 (14.5,20.2)	17.9 (15.0,20.7)	14.1 (11.5,16.6)	17.3 (14.4,20.3)	6.0 (1.9,10.9)	Urban
	Jeollanam-do	Suncheon-si	16.7 (15.4,18.0)	22.6 (19.0,26.2)	19.2 (16.1,22.2)	15.2 (12.4,18.1)	15.8 (13.0,18.7)	11.2 (8.9,13.6)	11.4 (9.2,17.8)	Urban
	Jeollanam-do	Naju-si	19.1 (17.8,20.4)	28.8 (25.0,32.7)	21.3 (18.1,24.4)	16.6 (14.0,19.2)	14.9 (12.5,17.3)	13.8 (11.4,16.2)	15.0 (11.9,21.1)	Urban
	Jeollanam-do	Gwangyang-si	16.8 (15.4,18.1)	24.1 (20.2,27.9)	18.0 (14.8,21.2)	15.5 (12.6,18.3)	14.1 (11.2,17.0)	12.6 (9.9,15.2)	11.5 (6.9,16.1)	Urban
	Jeollanam-do	Damyang-gun	19.2 (17.8,20.5)	24.8 (21.1,28.5)	21.1 (18.0,24.3)	16.4 (13.8,19.0)	17.2 (14.5,20.0)	16.6 (13.7,19.5)	8.2 (6.6,15.2)	Rural
	Jeollanam-do	Gokseong-gun	18.3 (17.0,19.6)	23.1 (19.6,26.6)	18.5 (15.8,21.2)	19.4 (16.4,22.4)	16.1 (13.5,18.7)	14.5 (12.2,16.9)	8.6 (5.7,14.3)	Rural
	Jeollanam-do	Gurye-gun	14.3 (13.2,15.5)	21.1 (17.8,24.5)	16.7 (14.0,19.3)	12.3 (10.2,14.4)	10.6 (8.5,12.6)	11.5 (9.2,13.8)	9.6 (7.2,15.6)	Rural
	Jeollanam-do	Goheung-gun	16.2 (14.9,17.4)	26.0 (21.8,30.2)	16.6 (14.0,19.1)	17.4 (14.4,20.4)	11.9 (9.9,13.9)	8.9 (7.2,10.6)	17.1 (14.2,22.2)	Rural
	Jeollanam-do	Boseong-gun	20.1 (18.7,21.5)	29.1 (24.9,33.3)	21.7 (18.5,25.0)	20.8 (17.6,24.0)	16.1 (13.5,18.7)	13.3 (11.2,15.4)	15.8 (11.8,21.0)	Rural
	Jeollanam-do	Hwasun-gun	20.7 (19.4,22.1)	31.6 (27.6,35.7)	19.9 (17.0,22.8)	20.4 (17.3,23.5)	16.6 (14.0,19.2)	15.8 (13.3,18.4)	15.8 (11.6,21.0)	Rural
	Jeollanam-do	Jangheung-gun	16.0 (14.8,17.1)	22.4 (18.9,25.9)	16.1 (13.5,18.7)	14.3 (11.8,16.7)	12.5 (10.4,14.6)	14.9 (12.2,17.5)	7.5 (2.6,11.0)	Rural
	Jeollanam-do	Gangjin-gun	15.7 (14.5,16.9)	21.7 (18.4,25.1)	14.6 (12.1,17.0)	14.1 (11.5,16.6)	14.7 (12.0,17.3)	13.6 (11.0,16.2)	8.1 (1.5,9.7)	Rural
	Jeollanam-do	Haenam-gun	19.1 (17.8,20.4)	24.6 (21.0,28.2)	19.7 (16.8,22.5)	18.2 (15.5,20.8)	17.1 (14.3,20.0)	16.1 (13.6,18.7)	8.5 (4.9,14.1)	Rural
	Jeollanam-do	Yeongam-gun	19.3 (18.0,20.7)	26.5 (22.8,30.3)	21.0 (17.9,24.2)	19.2 (16.2,22.3)	17.4 (14.6,20.2)	12.4 (10.1,14.7)	14.1 (8.7,17.7)	Rural
	Jeollanam-do	Muan-gun	16.1 (14.9,17.3)	19.6 (16.5,22.6)	16.4 (13.7,19.1)	17.6 (14.8,20.3)	13.3 (11.0,15.7)	13.8 (11.2,16.4)	5.8 (3.1,11.5)	Rural
	Jeollanam-do	Hampyeong-gun	21.7 (20.2,23.2)	30.7 (26.4,34.9)	22.2 (18.9,25.5)	19.7 (16.5,22.9)	18.5 (15.5,21.6)	17.3 (14.3,20.3)	13.4 (11.2,20.8)	Rural
	Jeollanam-do	Yeonggwang-gun	21.7 (20.3,23.1)	30.7 (26.8,34.7)	23.5 (20.3,26.8)	19.6 (16.6,22.6)	17.8 (15.2,20.4)	17.4 (14.4,20.4)	13.3 (10.5,20.3)	Rural
	Jeollanam-do	Jangseong-gun	18.3 (17.0,19.6)	25.9 (22.0,29.7)	19.3 (16.3,22.2)	16.5 (13.8,19.1)	17.0 (14.2,19.7)	13.3 (10.9,15.7)	12.6 (3.1,12.1)	Rural
	Jeollanam-do	Wando-gun	18.4 (17.1,19.7)	25.7 (22.0,29.4)	21.2 (18.3,24.2)	16.2 (13.6,18.9)	15.8 (13.4,18.2)	13.0 (10.7,15.3)	12.7 (9.5,18.3)	Rural
	Jeollanam-do	Jindo-gun	20.7 (19.3,22.1)	29.2 (25.1,33.3)	21.6 (18.4,24.7)	19.6 (16.7,22.6)	18.0 (15.2,20.8)	15.3 (12.6,18.0)	13.9 (10.4,19.6)	Rural
	Jeollanam-do	Sinan-gun	22.0 (20.5,23.6)	26.5 (22.8,30.3)	27.7 (23.6,31.8)	17.0 (14.3,19.7)	22.2 (18.8,25.7)	16.9 (13.9,19.9)	9.6 (8.8,18.6)	Rural
	Gyeongsangbuk-do	Nam-gu	19.7 (18.3,21.2)	28.6 (24.7,32.6)	20.1 (16.9,23.3)	18.7 (15.5,21.8)	15.2 (12.3,18.0)	16.0 (13.1,18.9)	12.6 (7.2,16.8)	Metropolitan
	Gyeongsangbuk-do	Buk-gu	17.9 (16.6,19.3)	26.6 (22.7,30.4)	18.4 (15.3,21.5)	16.4 (13.5,19.2)	15.1 (12.3,17.9)	13.5 (10.9,16.2)	13.1 (7.6,16.6)	Metropolitan
	Gyeongsangbuk-do	Gyeongju-si	17.8 (16.6,19.1)	24.8 (21.3,28.4)	19.6 (16.6,22.5)	16.8 (14.0,19.5)	15.6 (13.1,18.0)	12.8 (10.5,15.2)	12.0 (8.6,17.4)	Urban
	Gyeongsangbuk-do	Gimcheon-si	17.1 (15.8,18.3)	21.6 (18.4,24.8)	18.0 (15.2,20.8)	17.3 (14.5,20.2)	14.7 (12.0,17.3)	13.5 (11.0,16.0)	8.1 (3.5,12.3)	Urban
	Gyeongsangbuk-do	Andong-si	18.9 (17.5,20.2)	26.7 (23.0,30.3)	21.8 (18.5,25.1)	17.5 (14.8,20.3)	14.8 (12.2,17.5)	14.0 (11.5,16.5)	12.7 (7.1,16.3)	Urban
	Gyeongsangbuk-do	Gumi-si	18.7 (17.6,19.9)	27.7 (24.4,30.9)	20.8 (18.0,23.6)	15.7 (13.3,18.0)	16.1 (13.7,18.5)	14.7 (12.6,16.8)	13.0 (8.1,14.7)	Urban
	Gyeongsangbuk-do	Yeongju-si	19.5 (18.1,20.8)	26.3 (22.6,30.0)	21.0 (17.9,24.0)	18.8 (15.9,21.7)	16.0 (13.4,18.6)	15.7 (13.1,18.4)	10.6 (5.8,15.2)	Urban
	Gyeongsangbuk-do	Yeongcheon-si	21.0 (19.6,22.4)	25.6 (22.0,29.1)	23.6 (20.4,26.8)	19.7 (16.6,22.7)	20.6 (17.5,23.7)	15.7 (12.9,18.4)	9.9 (3.6,12.8)	Urban
	Gyeongsangbuk-do	Sangju-si	18.7 (17.3,20.0)	26.3 (22.6,30.1)	17.8 (15.0,20.7)	17.4 (14.6,20.2)	15.8 (13.2,18.4)	15.9 (13.0,18.7)	10.4 (3.8,12.8)	Urban
	Gyeongsangbuk-do	Mungyeong-si	18.7 (17.4,20.0)	22.7 (19.3,26.1)	20.4 (17.5,23.4)	18.8 (16.0,21.6)	17.8 (14.9,20.7)	13.6 (11.3,15.9)	9.1 (3.5,12.3)	Urban
	Gyeongsangbuk-do	Gyeongsan-si	18.5 (17.1,19.9)	24.7 (21.0,28.4)	20.7 (17.5,24.0)	15.7 (12.9,18.5)	17.3 (14.3,20.2)	14.3 (11.6,16.9)	10.4 (5.8,14.8)	Urban
	Gyeongsangbuk-do	Gunwi-gun	18.8 (17.5,20.2)	25.9 (22.0,29.9)	18.9 (16.1,21.7)	17.6 (14.7,20.4)	17.1 (14.2,19.9)	14.9 (12.2,17.6)	11.0 (4.3,13.7)	Rural
	Gyeongsangbuk-do	Uiseong-gun	18.4 (17.0,19.8)	25.3 (21.4,29.3)	18.2 (15.3,21.2)	16.1 (13.3,18.9)	17.9 (14.9,20.8)	14.8 (12.0,17.6)	10.5 (3.5,12.5)	Rural
	Gyeongsangbuk-do	Cheongsong-gun	20.0 (18.6,21.4)	27.3 (23.2,31.3)	21.4 (18.3,24.6)	17.5 (14.8,20.2)	18.1 (15.2,21.0)	15.9 (13.3,18.4)	11.4 (7.1,16.5)	Rural
	Gyeongsangbuk-do	Yeongyang-gun	23.2 (21.6,24.8)	34.2 (29.4,39.1)	23.9 (20.4,27.3)	22.1 (18.8,25.4)	19.9 (16.7,23.0)	16.5 (13.8,19.1)	17.7 (9.3,19.3)	Rural
	Gyeongsangbuk-do	Yeongdeok-gun	17.6 (16.3,18.9)	25.1 (21.4,28.8)	20.9 (17.6,24.2)	15.2 (13.0,17.4)	14.3 (11.9,16.8)	12.2 (10.0,14.5)	12.9 (9.1,17.5)	Rural
	Gyeongsangbuk-do	Cheongdo-gun	19.3 (18.0,20.7)	22.0 (18.8,25.2)	19.6 (16.6,22.7)	16.4 (13.9,18.8)	19.5 (16.3,22.6)	19.6 (16.3,22.8)	2.4 (-2.3,6.7)	Rural
	Gyeongsangbuk-do	Goryeong-gun	20.1 (18.7,21.5)	25.6 (21.9,29.3)	19.6 (16.6,22.5)	19.7 (16.5,22.8)	17.5 (14.8,20.3)	18.2 (15.1,21.3)	7.4 (1.8,11.0)	Rural
	Gyeongsangbuk-do	Seongju-gun	13.3 (12.2,14.4)	20.4 (17.0,23.7)	14.9 (12.2,17.5)	11.5 (9.3,13.6)	10.0 (8.0,12.1)	9.8 (7.7,12.0)	10.6 (5.0,13.0)	Rural
	Gyeongsangbuk-do	Chilgok-gun	18.5 (17.1,19.9)	23.7 (20.0,27.5)	18.6 (15.4,21.9)	19.6 (16.3,22.9)	15.6 (12.7,18.5)	15.6 (12.7,18.5)	8.1 (3.0,12.2)	Rural
	Gyeongsangbuk-do	Yecheon-gun	19.8 (18.4,21.2)	28.5 (24.4,32.7)	21.3 (18.1,24.4)	20.4 (17.0,23.8)	15.3 (12.8,17.8)	13.4 (11.1,15.7)	15.1 (7.7,16.9)	Rural
	Gyeongsangbuk-do	Bonghwa-gun	23.0 (21.4,24.6)	32.2 (27.5,37.0)	23.5 (20.0,27.0)	20.9 (17.7,24.0)	23.0 (19.4,26.6)	15.9 (13.2,18.5)	16.3 (10.9,20.7)	Rural
	Gyeongsangbuk-do	Uljin-gun	20.6 (19.2,21.9)	30.5 (26.4,34.5)	22.3 (19.2,25.4)	20.2 (17.3,23.2)	16.9 (14.2,19.7)	13.4 (11.2,15.6)	17.1 (12.0,21.2)	Rural
	Gyeongsangbuk-do	Ulleung-gun	17.7 (16.3,19.1)	22.4 (18.9,25.9)	20.1 (17.0,23.3)	17.1 (14.0,20.2)	15.1 (12.3,18.0)	13.4 (10.7,16.2)	9.0 (6.1,15.5)	Rural
	Gyeongsangnam-do	Changwon-si	20.3 (18.6,21.9)	26.5 (22.1,31.0)	20.8 (17.1,24.5)	19.6 (16.1,23.2)	19.1 (15.5,22.6)	16.0 (12.7,19.3)	10.5 (4.8,14.2)	Metropolitan
	Gyeongsangnam-do	Masan-si	15.9 (14.6,17.1)	23.4 (20.0,26.9)	15.8 (13.0,18.6)	14.5 (11.8,17.2)	14.9 (12.2,17.6)	11.2 (8.8,13.5)	12.2 (6.5,15.1)	Metropolitan
	Gyeongsangnam-do	Jinju-si	17.5 (16.2,18.8)	24.7 (21.1,28.2)	17.8 (14.8,20.7)	16.7 (13.9,19.5)	14.6 (12.0,17.2)	14.3 (11.6,17.0)	10.4 (5.9,14.9)	Urban
	Gyeongsangnam-do	Jinhae-si	17.6 (16.2,19.0)	22.3 (18.7,25.9)	19.4 (16.1,22.6)	16.1 (13.1,19.1)	15.7 (12.8,18.6)	14.8 (12.0,17.7)	7.5 (3.4,12.6)	Metropolitan
	Gyeongsangnam-do	Tongyeong-si	21.9 (20.4,23.3)	29.0 (25.1,32.9)	22.1 (18.8,25.4)	20.3 (17.2,23.5)	17.9 (15.0,20.8)	20.7 (17.4,23.9)	8.3 (3.9,13.7)	Urban

(continued to the next page)

Appendix 4. Continued

Gender	Provinces	Districts	Overall	Q1 (lowest)	Q2	Q3	Q4	Q5 (highest)	Q1-Q5	Urbanity
	Gyeongsangnam-do	Sacheon-si	17.8 (16.5,19.1)	25.4 (21.7,29.1)	20.3 (17.2,23.4)	15.7 (13.1,18.4)	14.6 (12.1,17.2)	13.5 (11.0,15.9)	11.9 (6.4,15.2)	Urban
	Gyeongsangnam-do	Gimhae-si	18.5 (17.0,20.0)	23.4 (19.5,27.4)	18.4 (15.1,21.7)	17.4 (14.2,20.6)	16.0 (13.0,19.1)	17.6 (14.4,20.8)	5.8 (1.4,10.6)	Urban
	Gyeongsangnam-do	Miryang-si	19.1 (17.8,20.4)	25.7 (22.3,29.2)	21.2 (18.1,24.3)	20.3 (17.2,23.3)	15.4 (12.9,17.8)	13.1 (10.8,15.4)	12.6 (9.3,18.1)	Urban
	Gyeongsangnam-do	Geoje-si	18.5 (16.9,20.0)	21.6 (17.9,25.4)	20.5 (16.9,24.2)	19.0 (15.5,22.5)	16.0 (12.7,19.2)	15.2 (12.1,18.2)	6.4 (1.6,10.8)	Urban
	Gyeongsangnam-do	Yangsan-si	15.2 (13.9,16.5)	23.1 (19.5,26.6)	15.5 (12.4,18.5)	13.8 (11.1,16.6)	12.1 (9.5,14.6)	11.8 (9.3,14.4)	11.3 (4.2,13.0)	Urban
	Gyeongsangnam-do	Uiryeong-gun	18.0 (16.7,19.3)	22.5 (19.1,25.9)	20.4 (17.1,23.7)	16.2 (13.5,18.8)	15.9 (13.3,18.5)	15.6 (12.7,18.5)	6.9 (5.6,14.6)	Rural
	Gyeongsangnam-do	Haman-gun	17.6 (16.4,18.8)	22.1 (18.9,25.3)	17.9 (15.2,20.7)	17.5 (14.8,20.2)	14.7 (12.2,17.2)	16.0 (13.3,18.7)	6.1 (2.6,11.6)	Rural
	Gyeongsangnam-do	Changnyeong-gun	20.7 (19.3,22.1)	25.8 (22.1,29.4)	21.9 (18.8,24.9)	18.7 (15.8,21.5)	18.1 (15.5,20.8)	19.3 (16.2,22.5)	6.5 (-0.3,9.3)	Rural
	Gyeongsangnam-do	Goseong-gun	17.3 (16.1,18.6)	20.5 (17.3,23.7)	18.2 (15.5,21.0)	17.9 (15.1,20.8)	17.2 (14.3,20.0)	12.9 (10.5,15.2)	7.6 (3.3,11.9)	Rural
	Gyeongsangnam-do	Namhae-gun	23.5 (22.0,25.1)	32.8 (28.3,37.4)	25.8 (22.2,29.4)	22.8 (19.4,26.1)	19.2 (16.3,22.1)	17.7 (14.6,20.8)	15.1 (9.8,19.4)	Rural
	Gyeongsangnam-do	Hadong-gun	23.5 (22.0,25.0)	30.7 (26.4,34.9)	27.1 (23.5,30.8)	20.6 (17.5,23.6)	18.8 (16.1,21.6)	20.4 (17.1,23.7)	10.3 (2.6,12.4)	Rural
	Gyeongsangnam-do	Sancheong-gun	20.8 (19.4,22.2)	27.1 (23.1,31.1)	23.6 (20.2,27.0)	19.5 (16.7,22.2)	18.2 (15.5,21.0)	15.6 (12.9,18.3)	11.5 (6.8,16.0)	Rural
	Gyeongsangnam-do	Hamyang-gun	20.6 (19.2,22.0)	23.7 (20.3,27.2)	22.3 (19.0,25.5)	19.4 (16.5,22.3)	19.2 (16.2,22.2)	18.5 (15.4,21.7)	5.2 (0.9,10.3)	Rural
	Gyeongsangnam-do	Geochang-gun	18.1 (16.8,19.3)	25.2 (21.7,28.7)	17.2 (14.5,19.8)	19.1 (16.2,22.1)	15.1 (12.6,17.6)	13.9 (11.5,16.4)	11.3 (6.2,15.2)	Rural
	Gyeongsangnam-do	Hapcheon-gun	21.0 (19.6,22.4)	25.7 (21.9,29.6)	24.5 (20.9,28.0)	21.8 (18.6,25.0)	18.5 (15.6,21.5)	14.7 (12.3,17.1)	11.0 (3.8,13.0)	Rural
	Jeju-do	Jeju-si	16.6 (15.9,17.4)	24.3 (22.1,26.5)	19.6 (17.6,21.6)	14.3 (12.6,16.0)	12.7 (11.2,14.2)	12.9 (11.4,14.4)	11.4 (9.1,14.3)	Urban
	Jeju-do	Seogwipo-si	16.4 (15.6,17.1)	23.3 (21.2,25.4)	17.3 (15.6,19.1)	14.9 (13.3,16.6)	13.0 (11.5,14.4)	13.8 (12.2,15.4)	9.5 (6.1,11.5)	Urban
