# Supplementary material for: Income gaps in self-rated poor health and its association with life expectancy in 245 districts of Korea
Source: Epidemiol Health. 2017 Mar 15;39:e2017011. doi: 10.4178/epih.e2017011 (PMC5543297; doi:10.4178/epih.e2017011)
Supplement: Supplementary file 6 [file epih-39-e2017011-app5.pdf]

Appendix 5. Mean equivalized household income and its relative ratios and absolute differences among metropolitan, urban, and rural areas: findings for total study population, those with the lowest and highest income quintiles using the Community Health Survey in South Korea, 2008-2014

Subjects	Measures	Metropolitan	Urban	Rural
Total study population	Mean equivalized house income	2,119.2	1,831.9	1,407.1
	Standard deviation	1,971.4	1,826.9	1,830.1
	Relative ratio	1.5	1.3	Reference
	Absolute difference	712.1	424.8	Reference
Those with Lowest income quintile	Mean equivalized house income	818.7	674.7	444.8
	Standard deviation	410.5	391.8	292.2
	Relative ratio	1.8	1.5	Reference
	Absolute difference	373.9	229.9	Reference
Those with Highest income quintile	Mean equivalized house income	4,088.0	3,638.5	3,036.4
	Standard deviation	3,496.9	3,245.4	3,453.5
	Relative ratio	1.3	1.2	Reference
	Absolute difference	1,051.6	602.1	Reference
